# A Careful Look at Binding Site Reorganization in the *even-skipped* Enhancers of *Drosophila* and Sepsids

**DOI:** 10.1371/journal.pgen.1000268

**Published:** 2008-11-28

**Authors:** Emily E. Hare, Brant K. Peterson, Michael B. Eisen

**Affiliations:** 1 Department of Molecular and Cell Biology, University of California Berkeley, Berkeley, California, United States of America; 2 Howard Hughes Medical Institute, University of California Berkeley, Berkeley, California, United States of America; Harvard Medical School, Howard Hughes Medical Institute, United States of America

In our recent *PLoS Genetics* paper [1] on the organization and activity of enhancers in the *even-skipped* gene of sepsid flies, we described, illustrated, analyzed, and discussed a series of small sequence blocks conserved between sepsid and *Drosophila* enhancers. We are thus rather surprised that Crocker and Erives [2] have announced the “discovery” of these conserved blocks in their Perspective on our paper. Nonetheless, we were happy to see that their reanalysis of our data reproduced our principle findings. Specifically, in their analyses, they confirm that:

- Sepsid and *Drosophila even-skipped* enhancers are highly diverged.
- Each of the *even-skipped* enhancers contains one or more small sequence blocks that are nearly identical between sepsids and drosophilids.
- These conserved blocks are modestly enriched for biochemically validated *D. melanogaster* sites and strongly enriched for paired *D. melanogaster* sites.
- Despite the presence of conserved blocks and this site enrichment, at least 70% of the functional binding sites in *D. melanogaster* are not detectably conserved with sepsids.

These results constitute the bulk of the sequence analysis reported in our paper, so we are in substantial agreement with Crocker and Erives about the nature of conservation between these two families. However their conclusion about the conservation of global organization of transcriptional information within these enhancers is based on several flawed assumptions and is inconsistent with information published by ourselves and others.

## Complex Organization of Conserved Blocks in Sepsid and *Drosophila* Enhancers

Crocker and Erives [2] base their analysis on the examination of what they call “two-dimensional homology plots” that reveal regions of similarity between two sequences. These plots place diagonal lines wherever BLAST finds a short stretch of sequence similarity (forward- and reverse-strand hits are distinguished by the direction of the line). The name “homology plot” is a misnomer, as most of the hits they display have BLAST scores too low to confirm the evolutionary relationship implied by the term homology—so we will use the more appropriate term “two-dimensional similarity plot.” Nonetheless, these plots, and the more commonly used dot plots [3], are an excellent tool for identifying **potential** regions of homology between distantly related species. Indeed, our initial discovery of the sepsid-*Drosophila*–conserved noncoding blocks was based on dot plots we generated to compare our newly identified enhancers to their *D. melanogaster* orthologs. These dot plots, as well as two-dimensional similarity plots for each enhancer, are shown in Figure S1.

Crocker and Erives cite the presence of multiple hits along a single diagonal in similarity plots of the *D. melanogaster* and *Themira putris* stripe 2 enhancers to argue that there is conservation of the global organization of this enhancer. However, they overemphasize the significance of this observation by displaying only a portion of the enhancer, placing reverse-strand hits in a separate panel, and augmenting on-diagonal hits with blue lines that are longer than the hits themselves.

**Figure 1:**
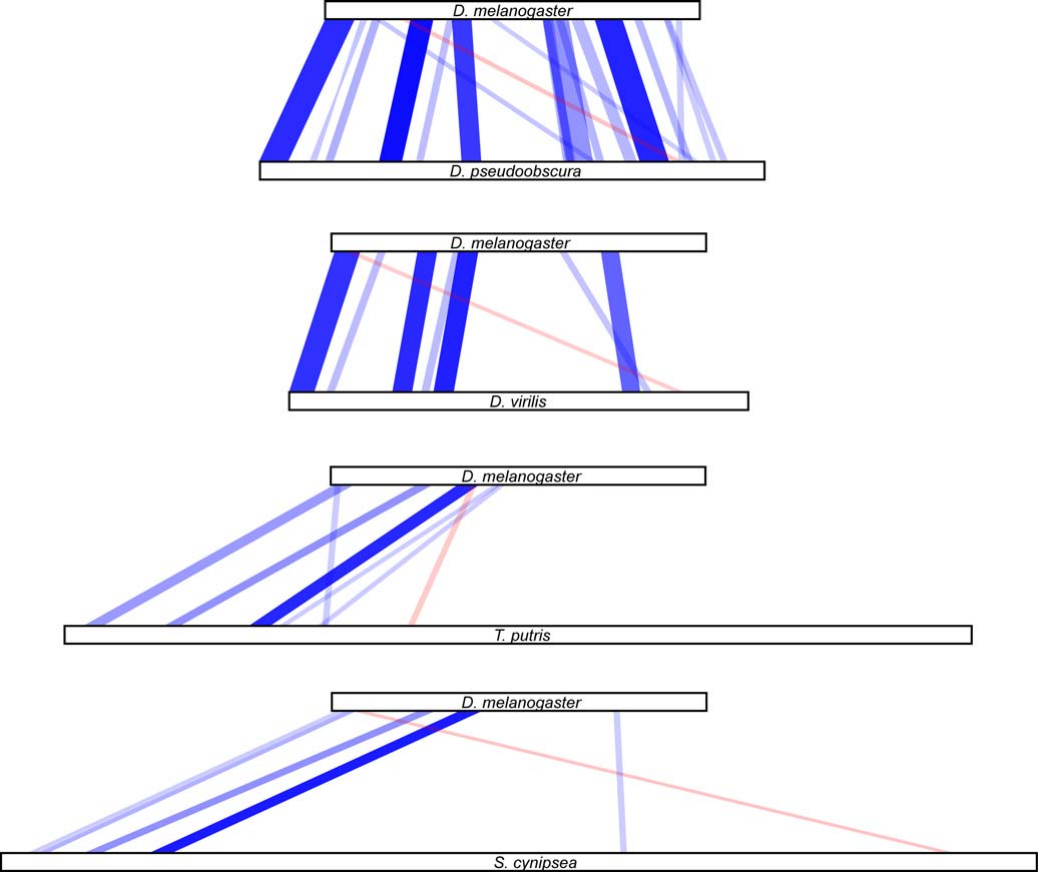
BLAST Similarity Maps of *D. melanogaster even-skipped* Stripe 2 Enhancer and Orthologous Enhancers from *Drosophila* and Sepsid Species. We aligned the *D. melanogaster even-skipped* stripe 2 enhancer against the orthologous enhancers of *D. pseudoobscura*, *D. virilis*, *T. putris*, and *Sepsis cynipsea* (sequences as described in [1]) using NBCI BLAST bl2seq v2.2.17, with default parameters except –W (wordsize) = 9. For each species pair, high-scoring pairs (HSPs) above the default E-value cutoff of 10 were mapped by drawing a box connecting the start and end of the hit in the query and target sequence. Blue boxes represent forward strand hits, red boxes indicate reverse strand hits. The opacity of the color was scaled so that the highest scoring BLAST hits had maximal opacity of 1.0 and the lowest scoring hit had opacity of 0.1.

**Figure 2:**
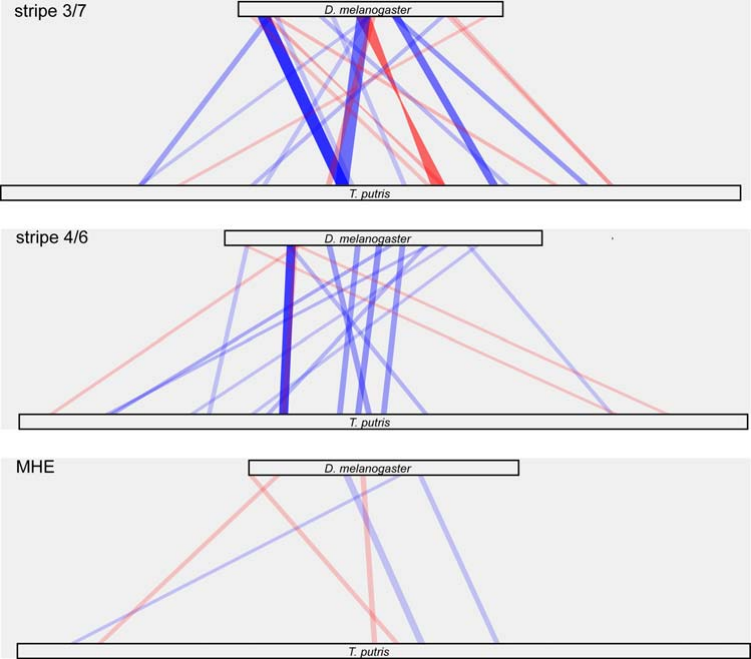
BLAST Similarity Maps of *D. melanogaster even-skipped* Stripe 2 Enhancer and Orthologous Enhancers. We aligned the *D. melanogaster even-skipped* stripe 3/7, stripe 4/6 and MHE enhancers against the orthologous enhancers of *T. putris* (sequences as described in [1]) using NBCI BLAST bl2seq v2.2.17, with default parameters except –W (wordsize) = 9. For each species pair, HSPs above the default E-value cutoff of 10 were mapped by drawing a box connecting the start and end of the hit in the query and target sequence. Blue boxes represent forward strand hits, red boxes indicate reverse strand hits. The opacity of the color was scaled so that the highest scoring BLAST hits had maximal opacity of 1.0 and the lowest scoring hit had opacity of 0.1.

A more straightforward way to visualize these data is to plot each enhancer in parallel, with regions of similarity connected by bars whose width represents the size of the matched region and whose color represents the degree of similarity. Such plots for all of the enhancers discussed in our paper [1] place the collinearity highlighted by Crocker and Erives [2] in context (Figure 1 and Figure 2) (additional maps using different similarity detection methods and different cutoffs are shown in Figure S2). First, the colinear blocks span a region that is less than half of the length of the minimal *D. melanogaster* enhancer. There is little or no conservation in the other half, which has been repeatedly shown to be required for proper functioning of the enhancer [4]–[7]. Crocker and Ervies left this nonconserved region out of their plots. Second, the blocks themselves cover only a small fraction of the bases in the enhancer. And finally, outside of the one very strongly conserved block (which was discussed extensively in our original paper), the similarity in the blocks is weak and often below or near the BLAST threshold for statistical significance. The relative weakness of the colinear conservation between families is particularly evident when viewed in the context of comparisons within *Drosophila*. The same general features are observed for stripe 4/6, where there is a single highly conserved block flanked by weakly conserved colinear blocks that span a fraction of the enhancer, interspersed with similarly conserved non-colinear blocks. No weak evidence for colinearity in stripe 3/7 or the muscle-heart enhancer (MHE) exists.

**Figure 3:**
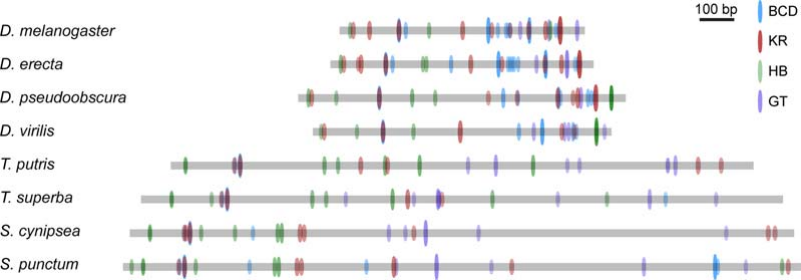
Predicted Binding Sites in the *even-skipped* Stripe 2 Enhancer of *Drosophila* and Sepsid Species. Predicted binding sites for HB, BCD, GT, and KR in the *even-skipped* stripe 2 enhancers of four *Drosophila* and four sepsid species. Sites were predicted using PATSER [14] using position-weight matrixes and cutoffs for each factor as described in [1]. The height of the oval representing each predicted binding site, and the intensity of the color inside the oval, are proportional to the score of the hit.

We agree with Crocker and Erives that it is possible for there to be a conserved binding site organization in the absence of detectable sequence conservation, as they and others have shown [8]–[11]. But, since binding sites can be destroyed or created by small numbers of substitutions, weak sequence conservation does not imply binding site conservation. For both these reasons, in our paper we focused our analyses of the *Drosophila* and sepsid enhancers on the organization of binding sites they contain (see Figure 3). These analyses strongly support the conclusion that there has been substantial reorganization of the regulatory information contained in these enhancers.

## Binding Sites in Conserved Blocks Are Often Not Conserved

**Figure 4:**
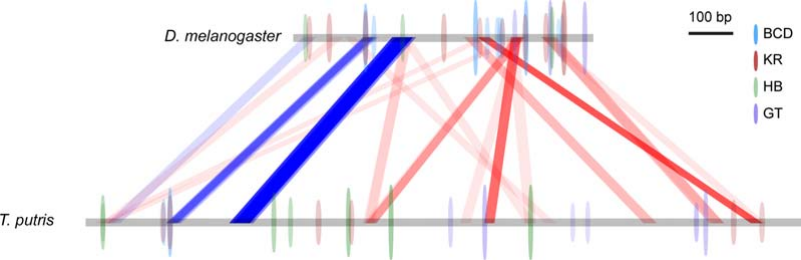
Similarity Map of *D. melanogaster even-skipped* Stripe 2 Enhancer and Predicted Binding Sites in *D. melanogaster* and *T. putris*. We compared all 20-bp windows in the *D. melanogaster* and *T. putris even-skipped* stripe 2 enhancers (sequences as described in [1]) and mapped regions with at least 14 identical base pairs. We have found that simple percent-identity plot gives a more reliable and robust measure of similarity that BLAST. We then predicted binding sites for HB, BCD, GT, and KR in both sequences using PATSER [14] with position-weight matrixes and cutoffs for each factor as described in [1]. The height of the oval representing each predicted binding site, and the intensity of the color inside the oval, are proportional to the score of the hit.

Many of the differences between our and Crocker and Erives' views of enhancer evolution arise from a serious logical flaw in the analyses they present: they repeatedly and mistakenly equate the presence of a *D. melanogaster* binding site in a conserved block with the conservation of that binding site. They identified ten biochemically validated *D. melanogaster* binding sites in their conserved blocks (Figure 3 in [2]). However, when they searched for binding sites in the *T. putris* version of each of these sequences (Figure 4 in [2]), they found only four of these ten sites. The interspecies differences in these imperfectly conserved blocks have transformed six of the *D. melanogaster* binding sites into *T. putris* sequences no longer recognized by the same factors. (This lack of correspondence between the weak sequence conservation in their blocks and binding site conservation is illustrated in Figure 4.)

By their own analysis, KR-5 and KR-6 are the only conserved Krüppel binding sites. Nonetheless, in their discussion of these data, they continue to treat all five Krüppel binding sites in conserved blocks as being conserved:

Specifically, two high-affinity Kruppel repressor binding sites, KR-6 and KR-5, occur in conserved blocks A and B, respectively, while one and two low-affinity Kruppel binding sites (KRW sites) are present in conserved blocks E and F, respectively (Figures 3 and 4). Thus, this organized array of conserved Kruppel repressor binding sites spans ∼300 bp.

But the KRW sites are not conserved. Without them, only two closely spaced conserved sites remain, and we do not see how these can be said to constitute an “organized array.” Rather, the lack of conservation of these Krüppel sites supports the opposite conclusion—the one we made in our paper [1]—that the organization of binding sites within these enhancers is highly flexible.

Crocker and Erives suggest that they may not detect sites in *T. putris* because “*Themira* binding preferences may have diverged since their latest common ancestor, resulting in an artifactual phylogenetic decay of detection.” This argument, however, ignores another major finding of our paper—that these sepsid enhancers function normally in transgenic *D. melanogaster* embryos. If the binding specificity of Krüppel had diverged significantly between the two families, we would not expect *D. melanogaster* Krüppel to repress expression from the *T. putris* stripe 2 enhancer, as the proper expression of sepsid stripe 2 in *D. melanogaster* embryos strongly suggests it does.

## Organization of *D. melanogaster even-skipped* Stripe 2 Enhancer Is Not Conserved in Sepsids

The major point of our paper was that the extensive divergence between sepsid and *Drosophila* enhancers—both in terms of raw sequence and the overall composition and organization of binding sites within the enhancers—is inconsistent with the idea that there is only one fixed organization of sites capable of generating the *even-skipped* stripe and MHE expression patterns.

Detailed experimental dissections of the *even-skipped* stripe 2 enhancer have identified 24 sites that are bound by the factors that regulate the enhancer (HB, BCD, GT, KR, SLP1), and many of these sites have been shown to contribute significantly to the enhancer's activity [4]–[7],[12],[13]. By any measure, only a small fraction of these sites are detectably conserved between the families (of the 17 *D. melanogaster* HB, BCD, GT, and KR sites Crocker and Erives analyzed, they found only five in *T. putris*). Based on earlier experimental work, we do not believe that this vestige of *D. melanogaster* binding site organization is sufficient to explain the conserved activity of these enhancers. For example, *T. putris* does not contain an ortholog of the *D. melanogaster* BCD-1 site, yet deletion of BCD-1 from the *D. melanogaster* stripe 2 element destroys its activity [4].

We looked extensively for evidence of a conserved global organization of transcription factor binding sites between sepsid and *Drosophila* enhancers, and we have been unable to find any. We have also looked at the pattern of gain and loss of binding sites within families—where accurate alignments can be readily computed. If the “skeleton key” model favored by Crocker and Erives is correct, binding site loss at one location must be accompanied by the gain of a site nearby, otherwise the global organization of sites within the enhancer would be disrupted. However, we again find no evidence for such an effect.

The example of the essential BCD-1/KR-3 pair in *D. melanogaster* stripe 2 is particularly illustrative. As shown in our original Figure 6 (in [1]), this pair is conserved in closely related *Drosophila* species, degraded in the more distant *Drosophila* species, and absent from the sepsids. There are no equivalent KR or BCD sites in that region of the sepsid enhancers. However, there is an overlapping pair of KR/BCD sites in a distal region of the enhancer. Although we have not yet assayed the function of these paired sites experimentally, they are conserved throughout the sepsids, suggesting that they are important to the enhancer's activity (and highlighting the value of examining multiple species within each family). This pattern of evolution is inconsistent with strict conservation of global enhancer organization.

## Conclusion

A careful analysis of sepsid and *Drosophila even-skipped* enhancers reveals changes in the organization of transcription factor binding sites that are not compatible with a model in which conserved expression patterns are generated by a single conserved binding site organization. More sophisticated analyses and additional data are needed to define what is required—at the sequence level—to produce a specific pattern of expression. Comparisons of divergent sequences with conserved function provide an especially powerful window into the molecular logic of gene regulation, and we are glad that our exploration of the genetic diversity of fly enhancers has inspired others to begin thinking about this problem.

## Supporting Information

Figure S1 Dot plots and BLAST-based two-dimensional similarity plots for four *even-skipped* enhancers in multiple *Drosophila* and sepsid species. Dot plots based on percent identity in windows of 14 and 20 bp comparing the *D. melanogaster even-skipped* stripe 2, stripe 3/7, stripe 4/6, and muscle-heart enhancers to their orthologs in *D. pseudoobscura*, *D. virilis*, *T. putris*, and *S. cynipsea* (sequences as described in [1]). Blocks with identities greater than 60% are shown, with the shading of the black box proportional to the strength of the match. BLAST-based two-dimensional similarity plots were computed by aligning the *D. melanogaster even-skipped* stripe 2, stripe 3/7, stripe 4/6, and muscle-heart enhancers to their orthologs in *D. pseudoobscura*, *D. virilis*, *T. putris*, and *S. cynipsea* (sequences as described in [1]) using NBCI BLAST bl2seq v2.2.17, with default parameters except –set 1, W (wordsize) = 9, E-value cutoff of 10; set 2 W = 7, E-value cutoff of 20. HSPs above E-value cutoff are shown, with the shading of the black box proportional to the strength of the match.
(1.01 MB PDF).

Figure S2 Similarity maps (derived from dot plots and BLAST-based similarity plots) for four *even-skipped* enhancers in multiple *Drosophila* and sepsid species. Similarity maps were computed for dotplots and BLAST based 2D similarity plots shown in Figure S1. For each set of dot plots, three maps are shown, each with a different threshold on which dotplot hits are shown: cutoffs of 0.50, 0.60, and 0.70 representing the position of the score for the hit between the highest and lowest scores (a cutoff of 0.60, for example, means that only hits in the top 40% of the range shown in the dot plot are mapped). For BLAST-based similarity maps, all HSPs in the similarity plots are shown. Blue boxes represent forward strand hits, red boxes indicate reverse strand hits. The opacity of the color was scaled so that the highest scoring hits had maximal opacity of 1.0 and the lowest scoring hit had opacity of 0.1.
(1.37 MB PDF).
