# Supplementary material for: A Careful Look at Binding Site Reorganization in the even-skipped Enhancers of Drosophila and Sepsids
Source: PLoS Genet. 2008 Nov 28;4(11):e1000268. doi: 10.1371/journal.pgen.1000268 (PMC2582681; doi:10.1371/journal.pgen.1000268)
Supplement: Figure S1 — Dot plots and BLAST-based two-dimensional similarity plots for four even-skipped enhancers in multiple Drosophila and sepsid species. Dot plots based on percent identity in windows of 14 and 20 bp comparing the D. melanogaster even-skipped stripe 2, stripe 3/7, stripe 4/6, and muscle-heart enhancers to their orthologs in D. pseudoobscura, D. virilis, T. putris, and S. cynipsea (sequences as described in [1]). Blocks with identities greater than 60% are shown, with the shading of the black box proportional to the strength of the match. BLAST-based two-dimensional similarity plots were computed by aligning the D. melanogaster even-skipped stripe 2, stripe 3/7, stripe 4/6, and muscle-heart enhancers to their orthologs in D. pseudoobscura, D. virilis, T. putris, and S. cynipsea (sequences as described in [1]) using NBCI BLAST bl2seq v2.2.17, with default parameters except –set 1, W (wordsize) = 9, E-value cutoff of 10; set 2 W = 7, E-value cutoff of 20. HSPs above E-value cutoff are shown, with the shading of the black box proportional to the strength of the match. (1.01 MB PDF). [file pgen.1000268.s001.pdf]

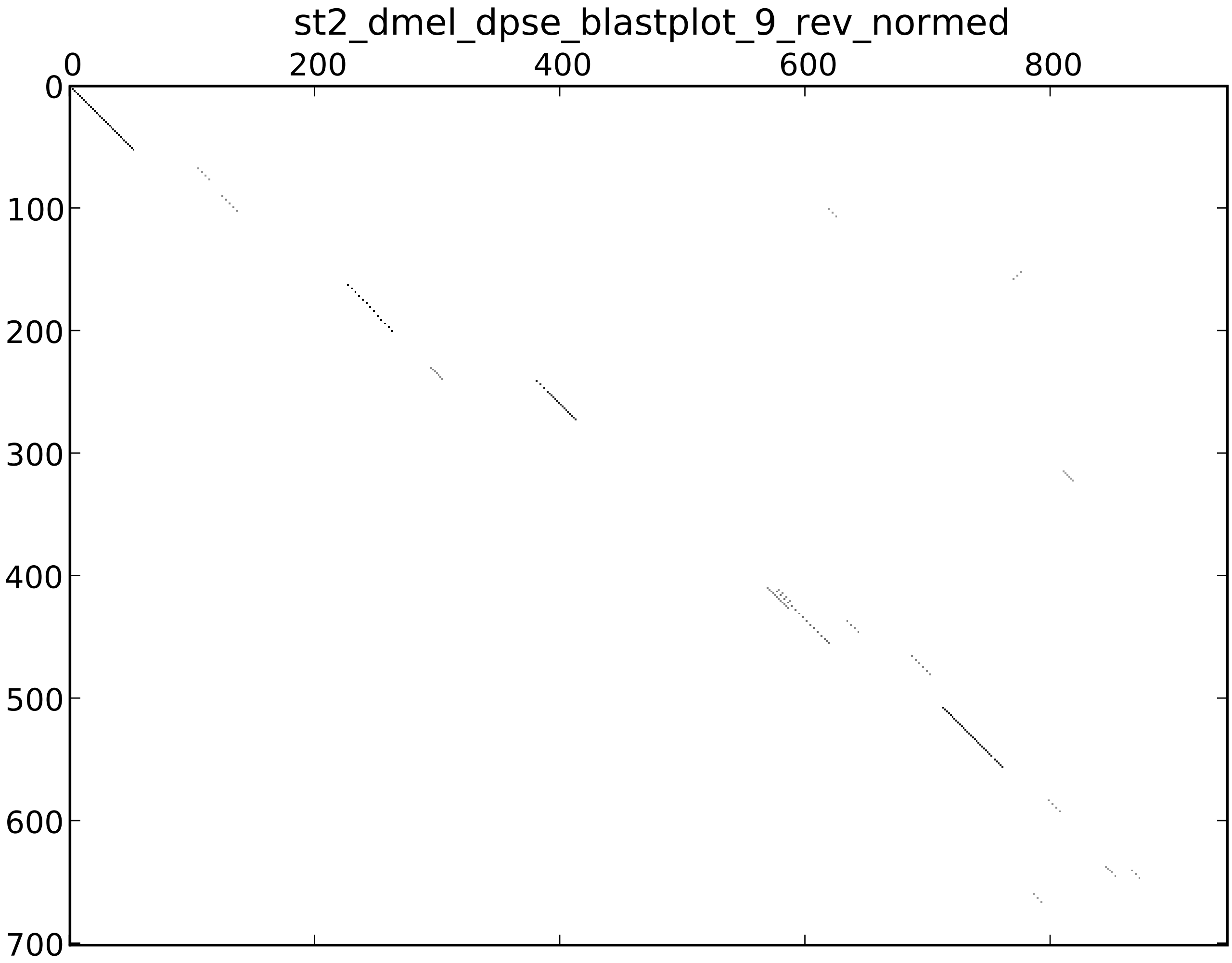

st2_dmel_dpse_blastplot_9_rev_normed
0
200
400
600
800
0
100
200
300
400
500
600
700

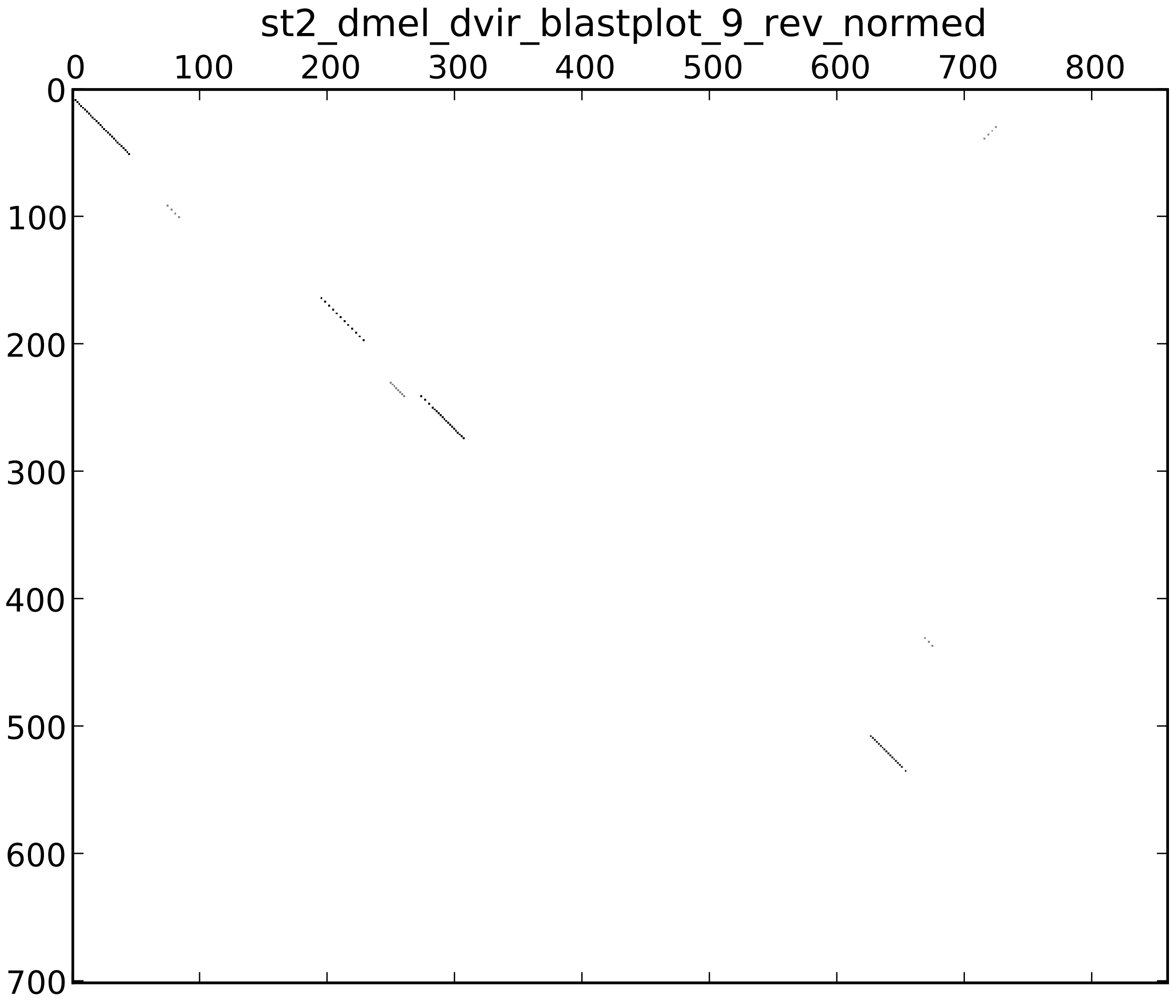
st2_dmel_dvir_blastplot_9_rev_normed
0
100
200
300
400
500
600
700
800
0
100
200
300
400
500
600
700

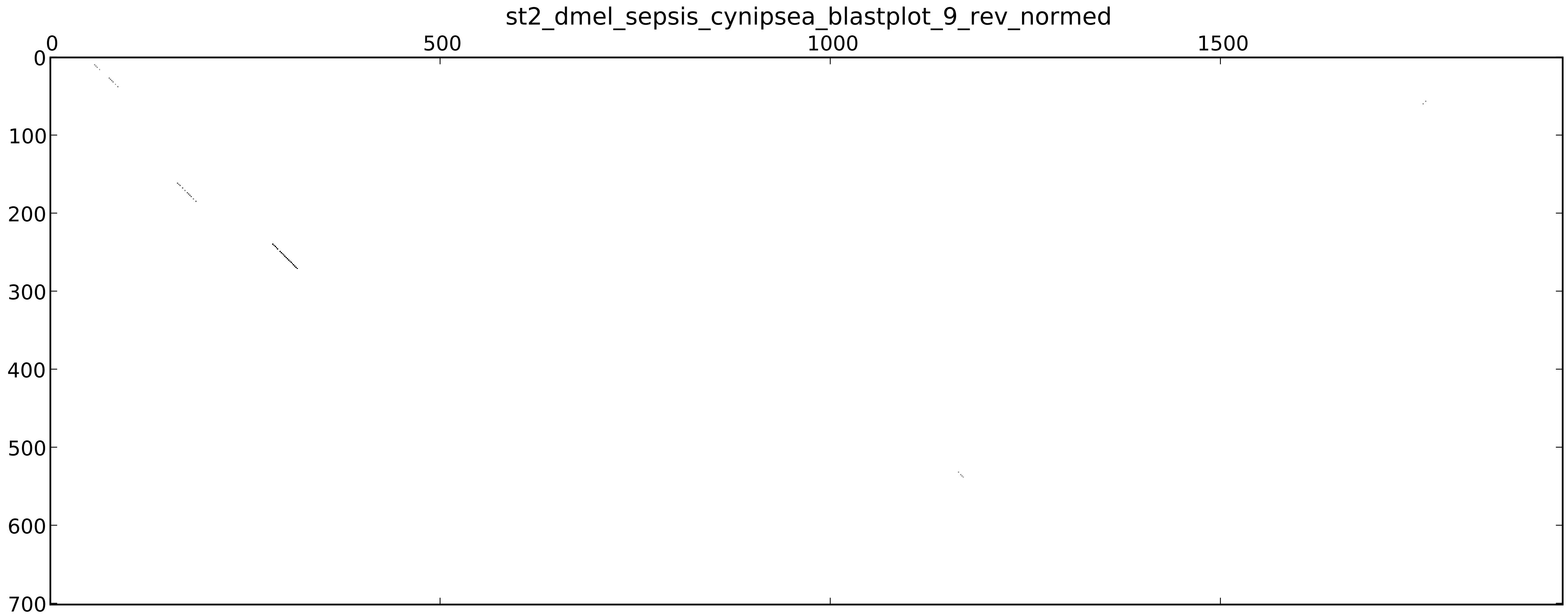
st2_dmel_sepsis_cynipsea_blastplot_9_rev_normed
0
500
1000
1500
0
100
200
300
400
500
600
700

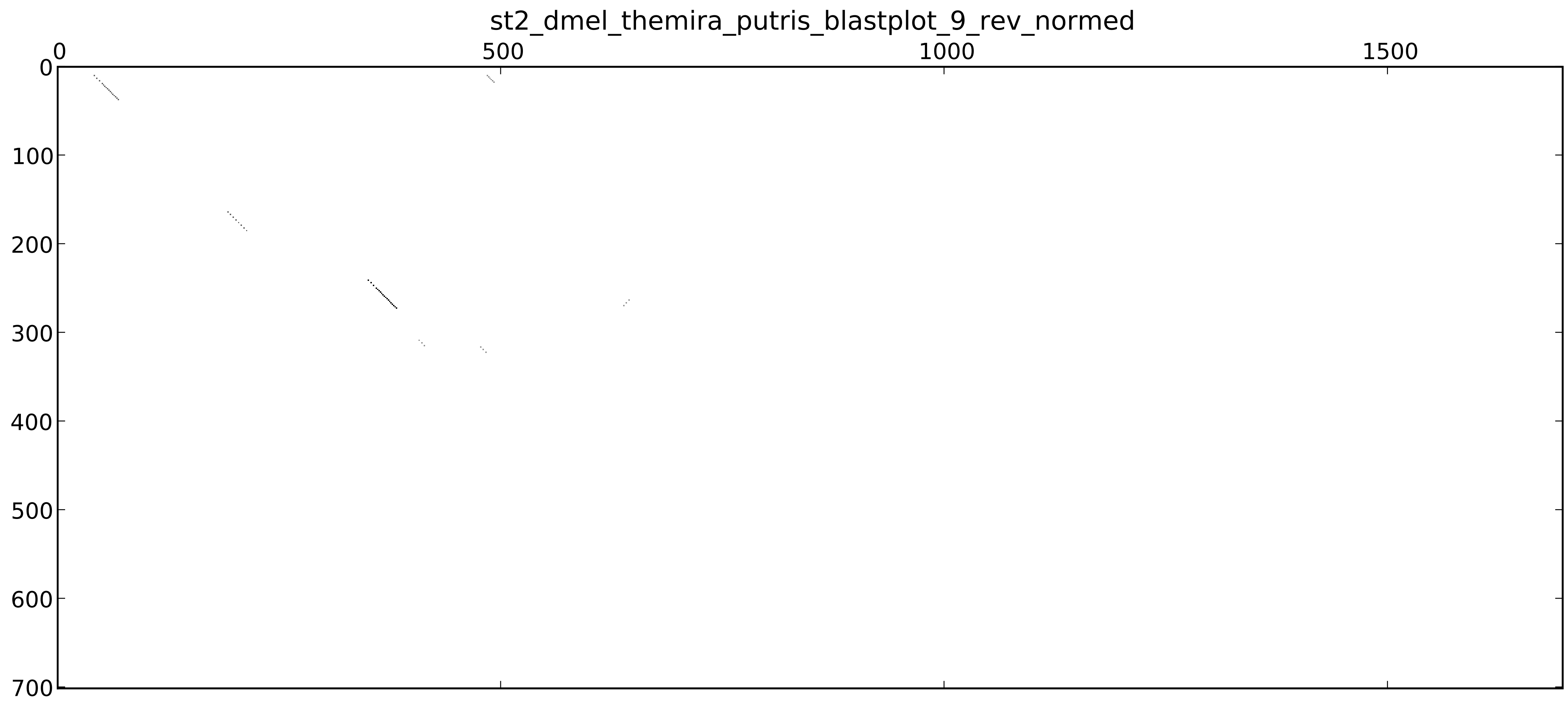
st2_dmel_themira_putris_blastplot_9_rev_normed
0
500
1000
1500
0
100
200
300
400
500
600
700

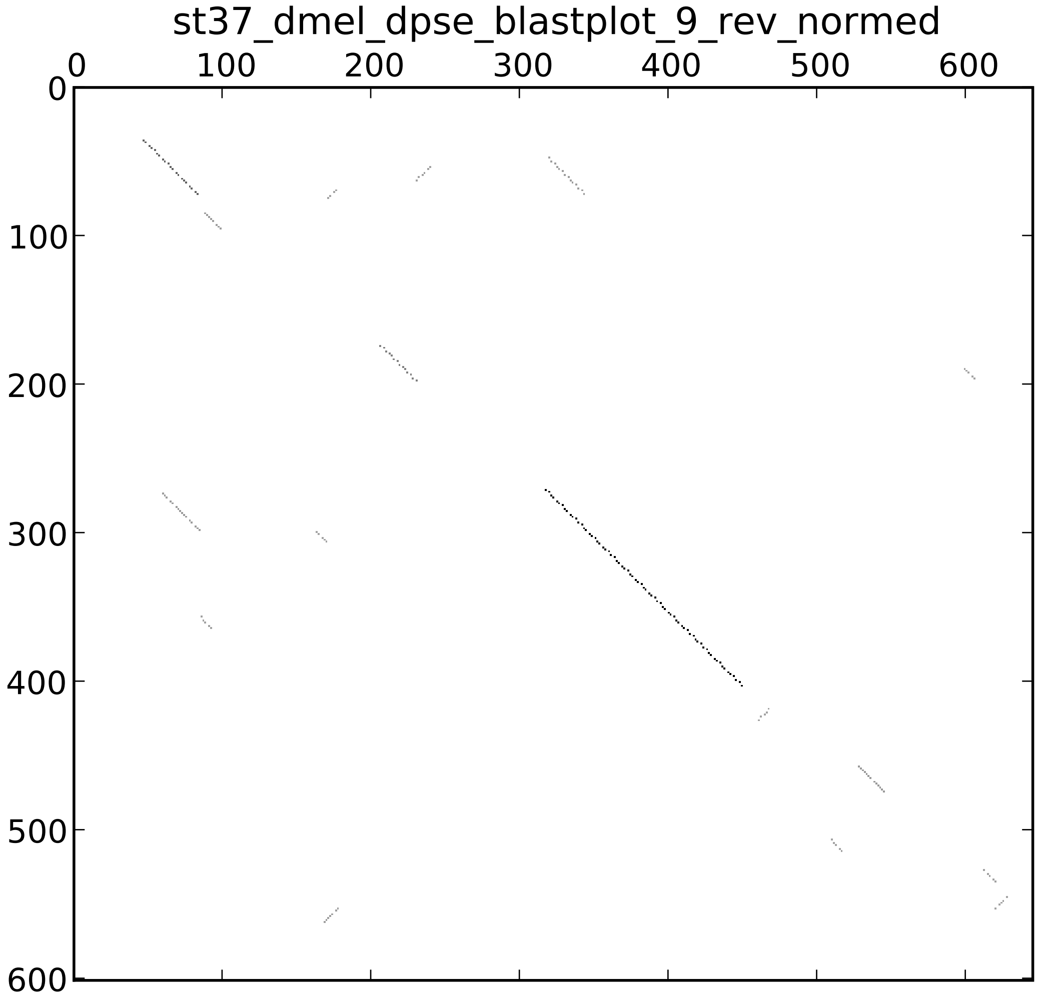

st37_dmel_dpse_blastplot_9_rev_normed
0
100
200
300
400
500
600
0
100
200
300
400
500
600

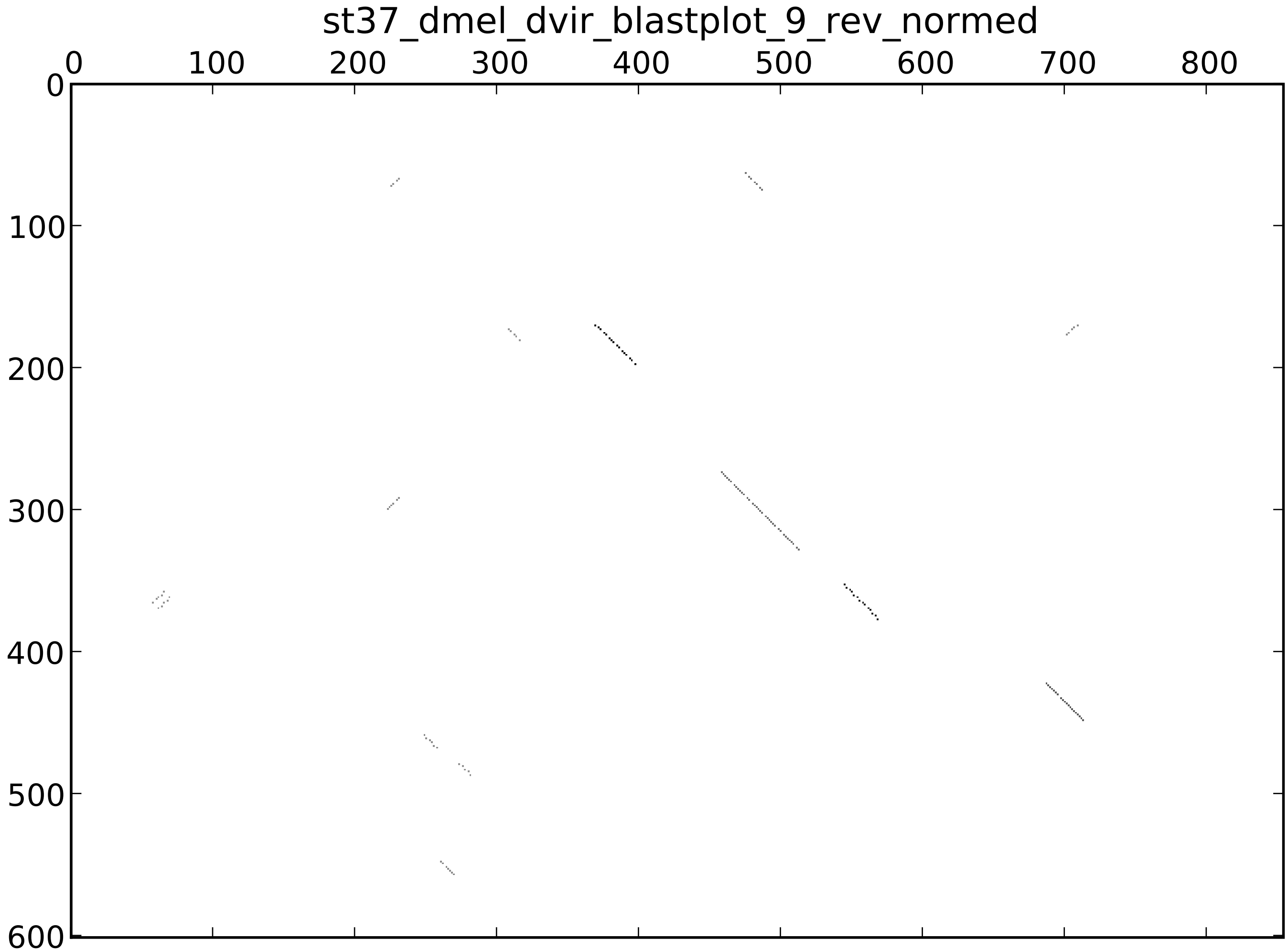

st37_dmel_dvir_blastplot_9_rev_normed
0
100
200
300
400
500
600
700
800
0
100
200
300
400
500
600

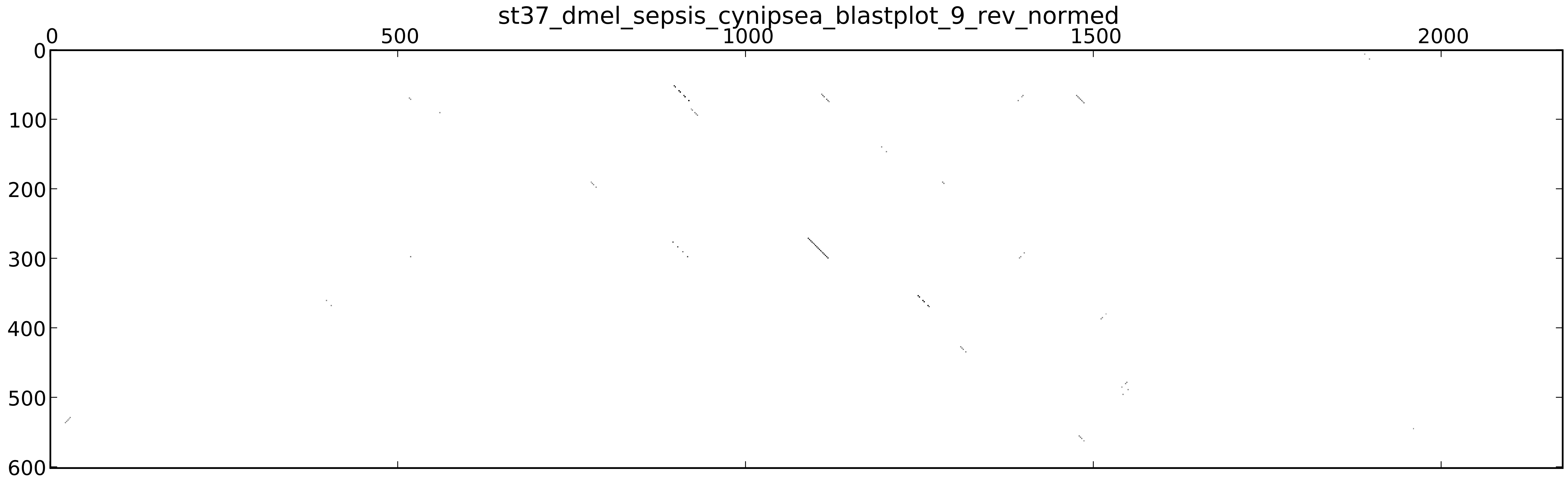
st37_dmel_sepsis_cynipsea_blastplot_9_rev_normed
0
500
1000
1500
2000
0
100
200
300
400
500
600

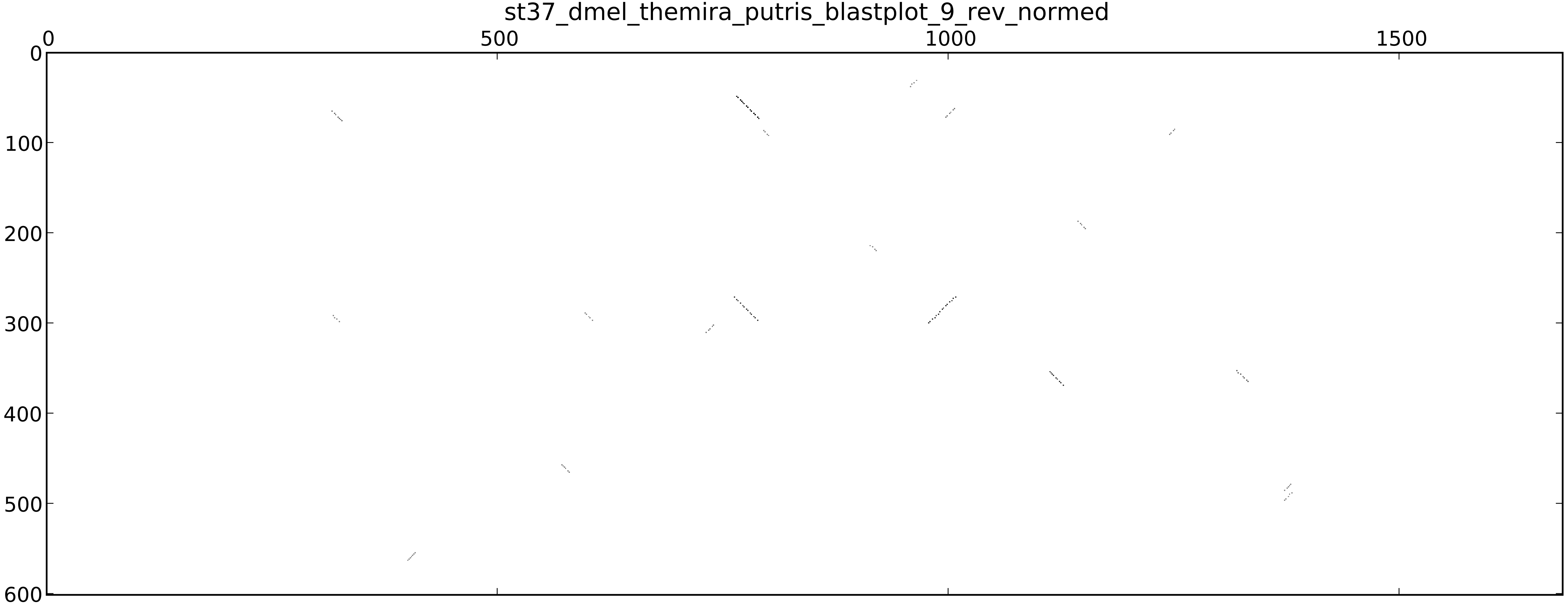
st37_dmel_themira_putris_blastplot_9_rev_normed
0
500
1000
1500
0
100
200
300
400
500
600

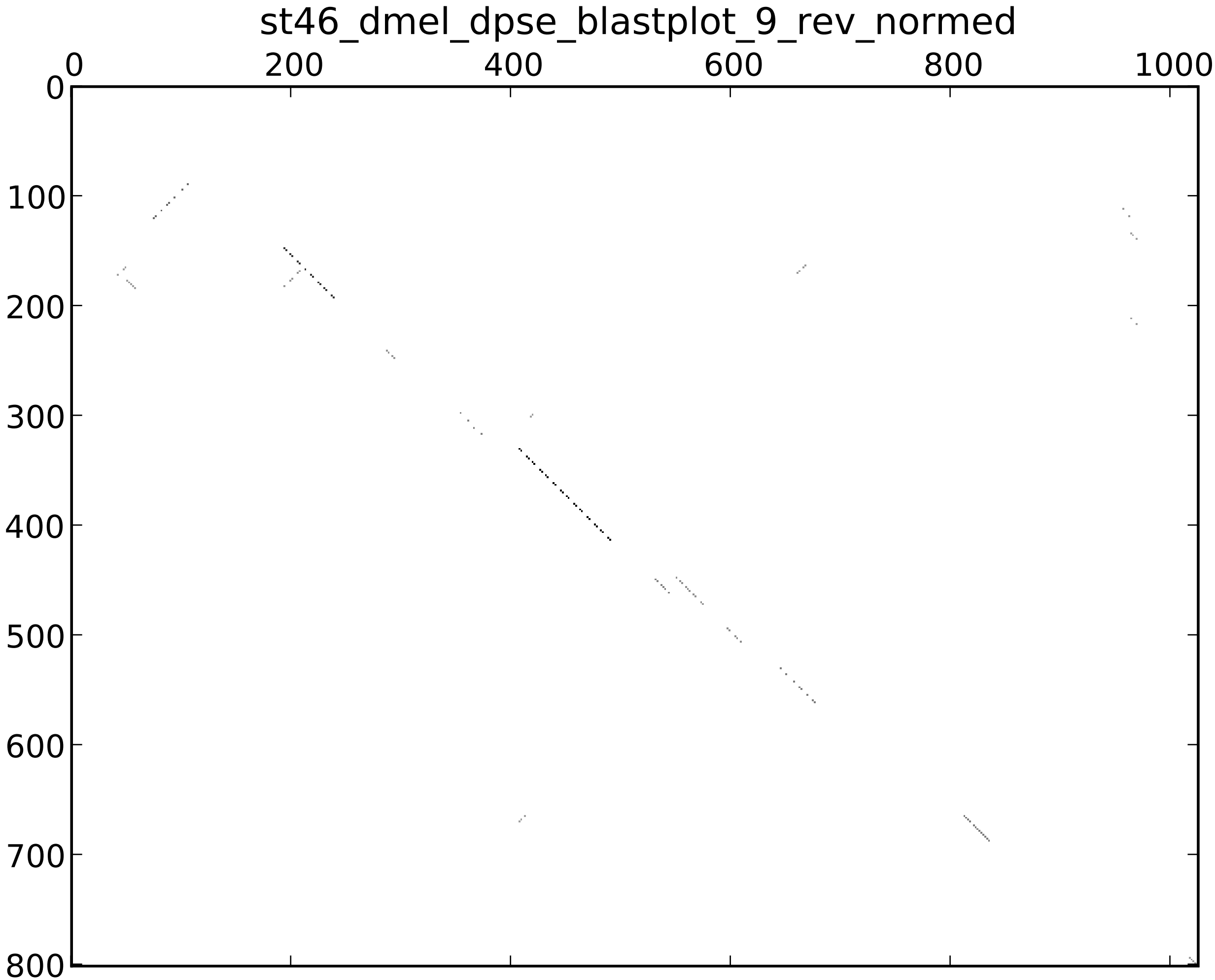

st46_dmel_dpse_blastplot_9_rev_normed
0
200
400
600
800
1000
0
100
200
300
400
500
600
700
800

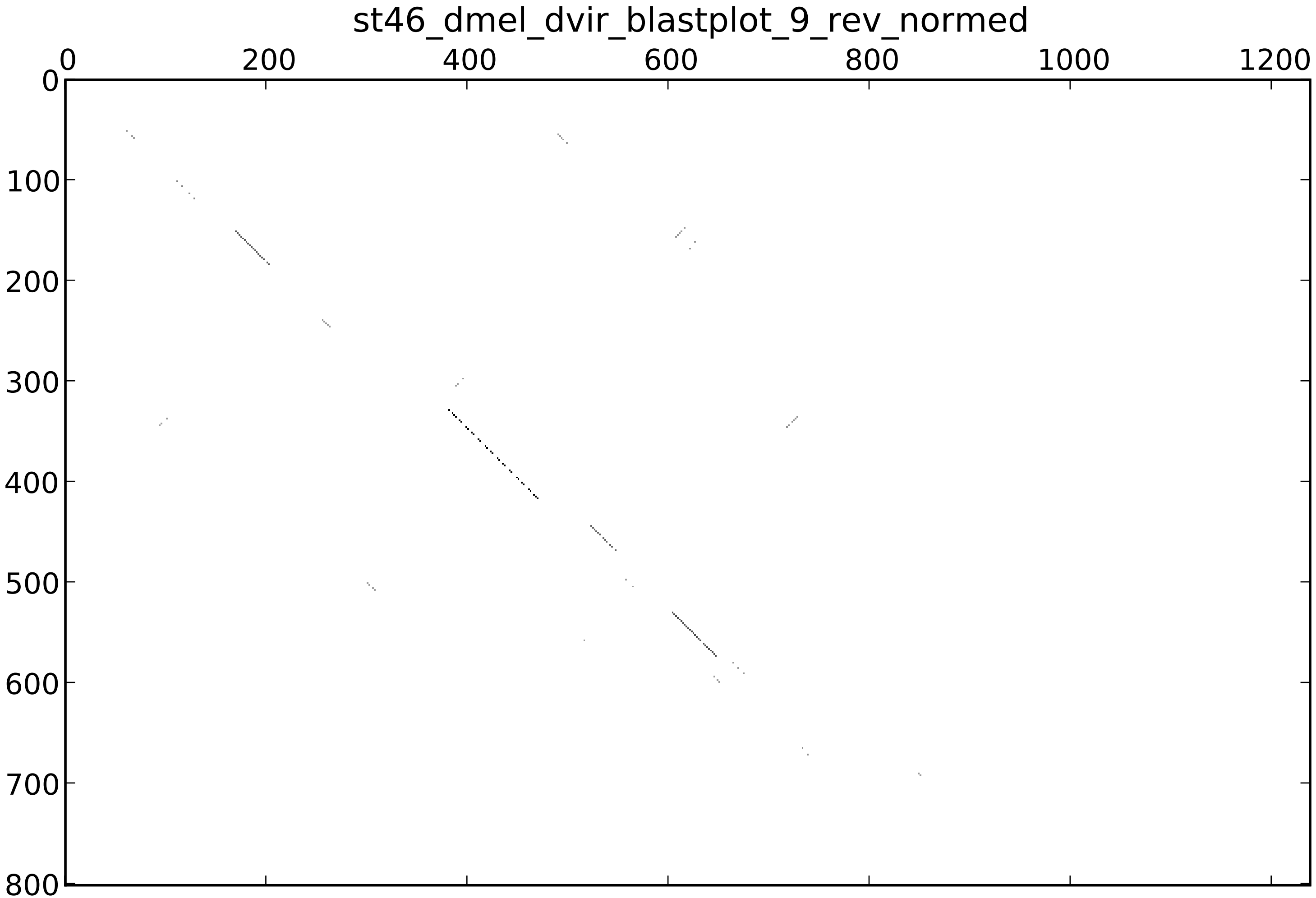
st46_dmel_dvir_blastplot_9_rev_normed
0
200
400
600
800
1000
1200
0
100
200
300
400
500
600
700
800

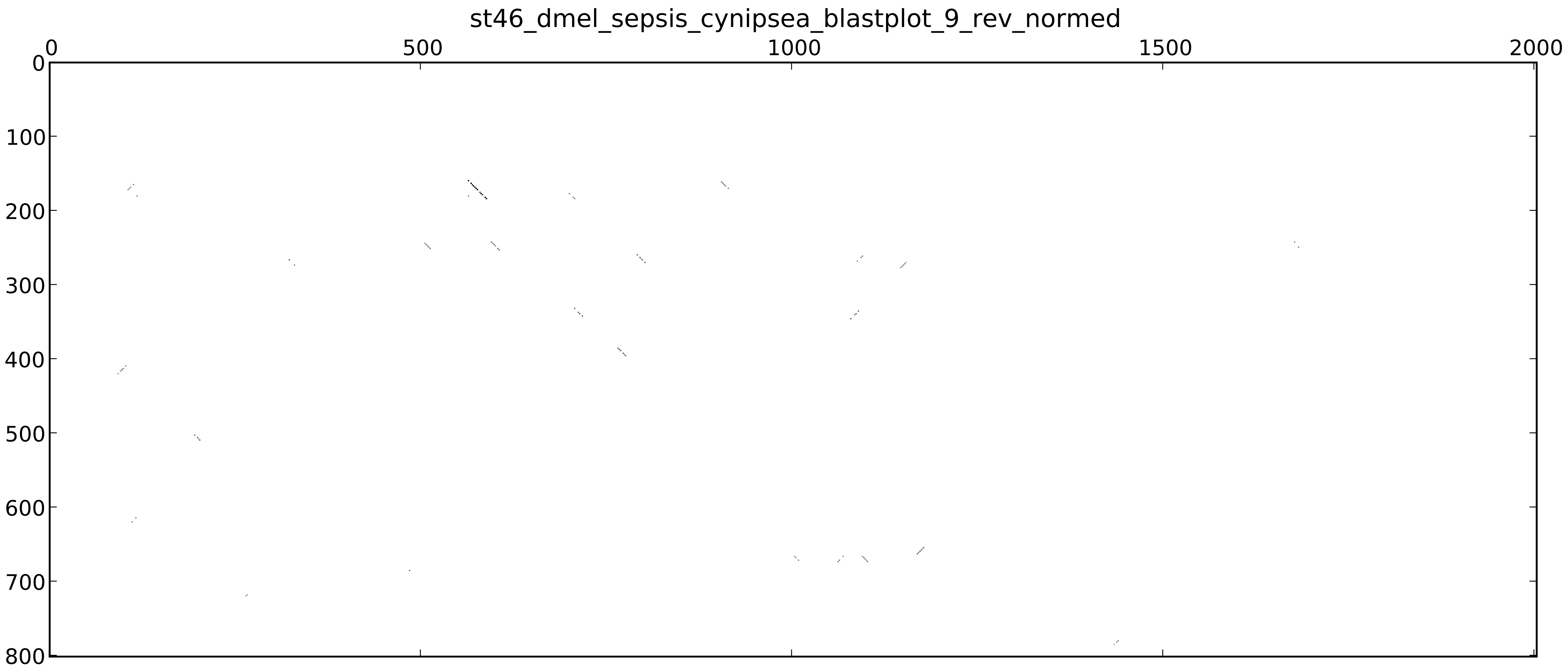
st46_dmel_sepsis_cynipsea_blastplot_9_rev_normed
0
500
1000
1500
2000
0
100
200
300
400
500
600
700
800

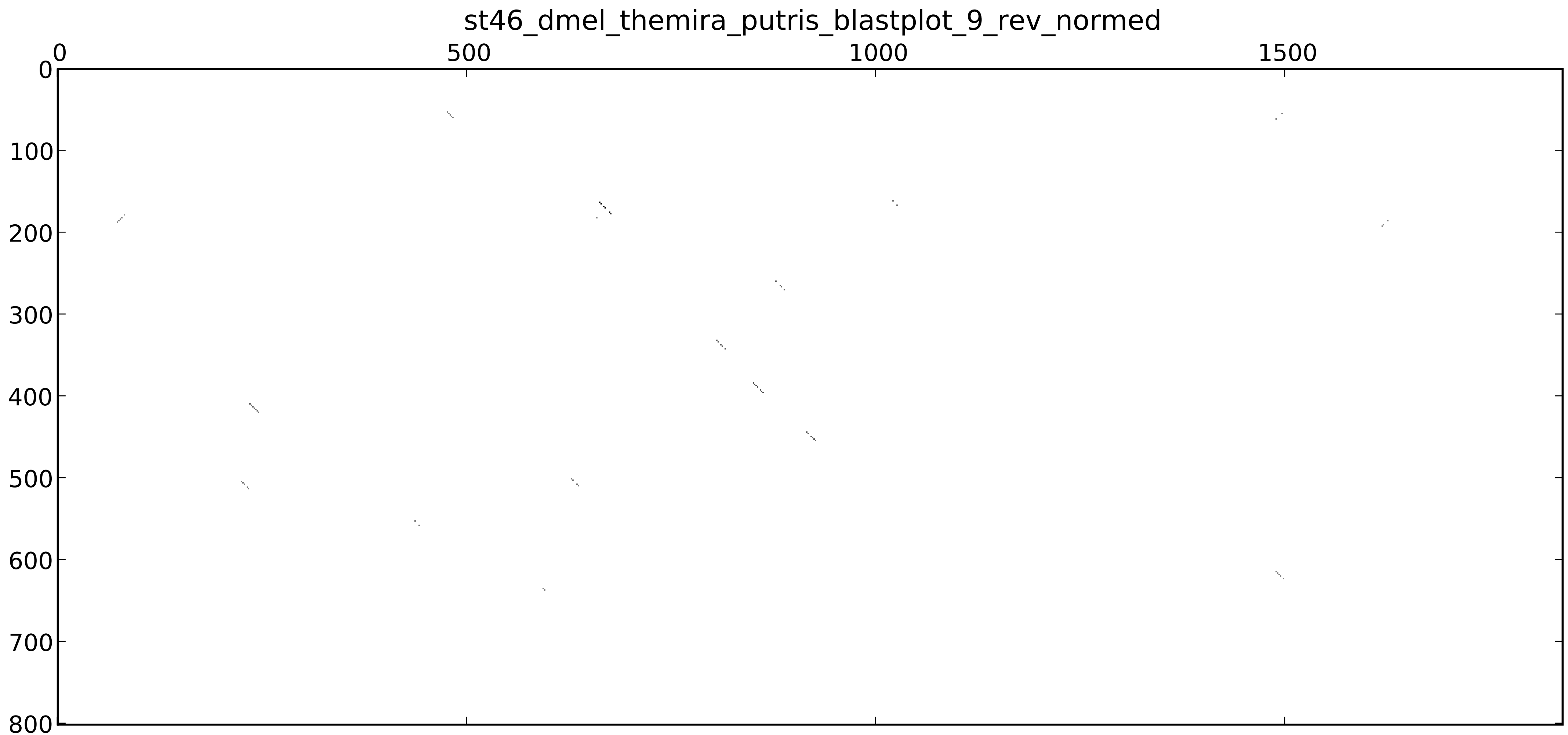
st46_dmel_themira_putris_blastplot_9_rev_normed
0
500
1000
1500
0
100
200
300
400
500
600
700
800

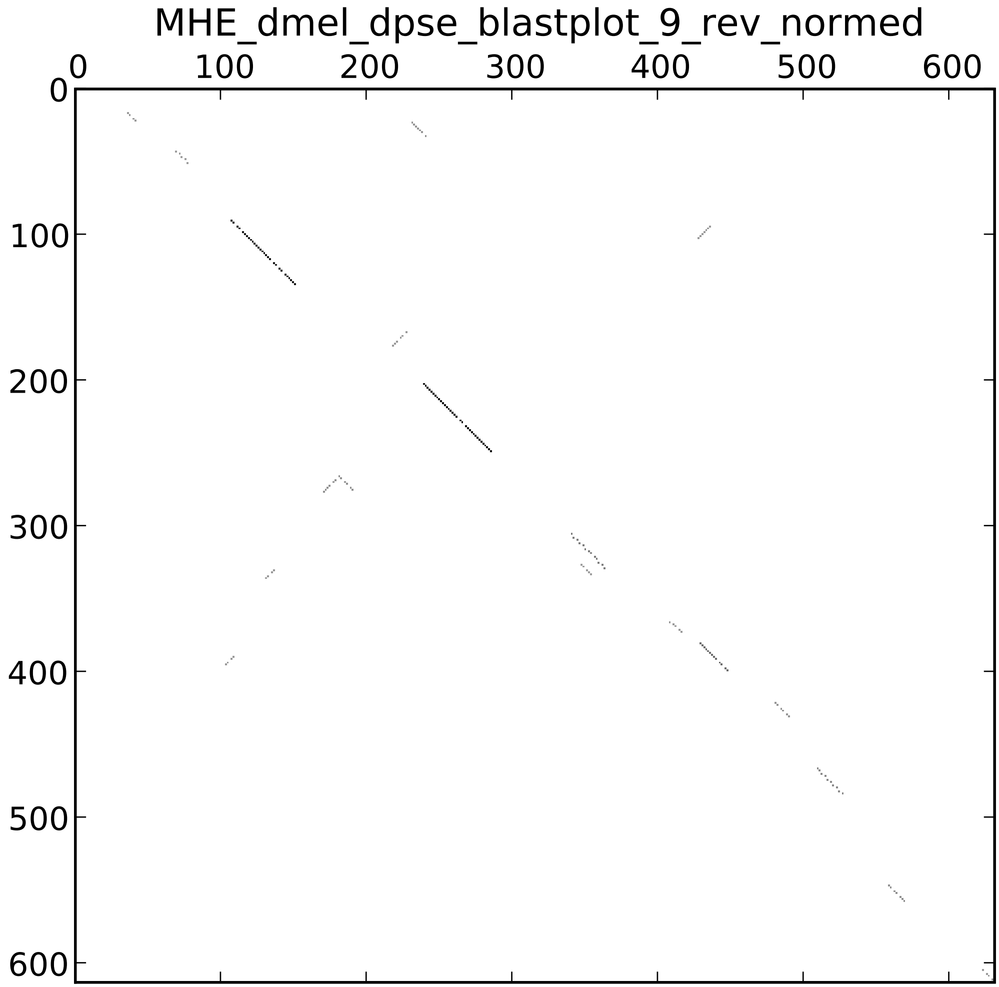
MHE_dmel_dpse_blastplot_9_rev_normed
0
100
200
300
400
500
600
0
100
200
300
400
500
600

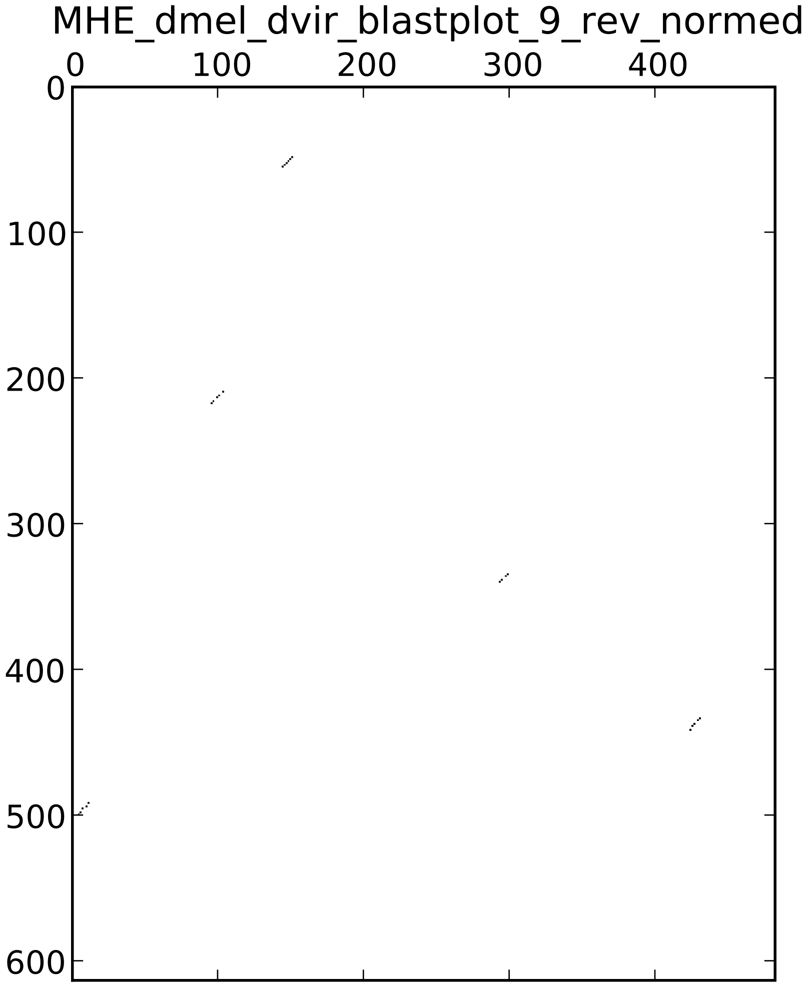
MHE_dmel_dvir_blastplot_9_rev_normed
0
100
200
300
400
0
100
200
300
400
500
600

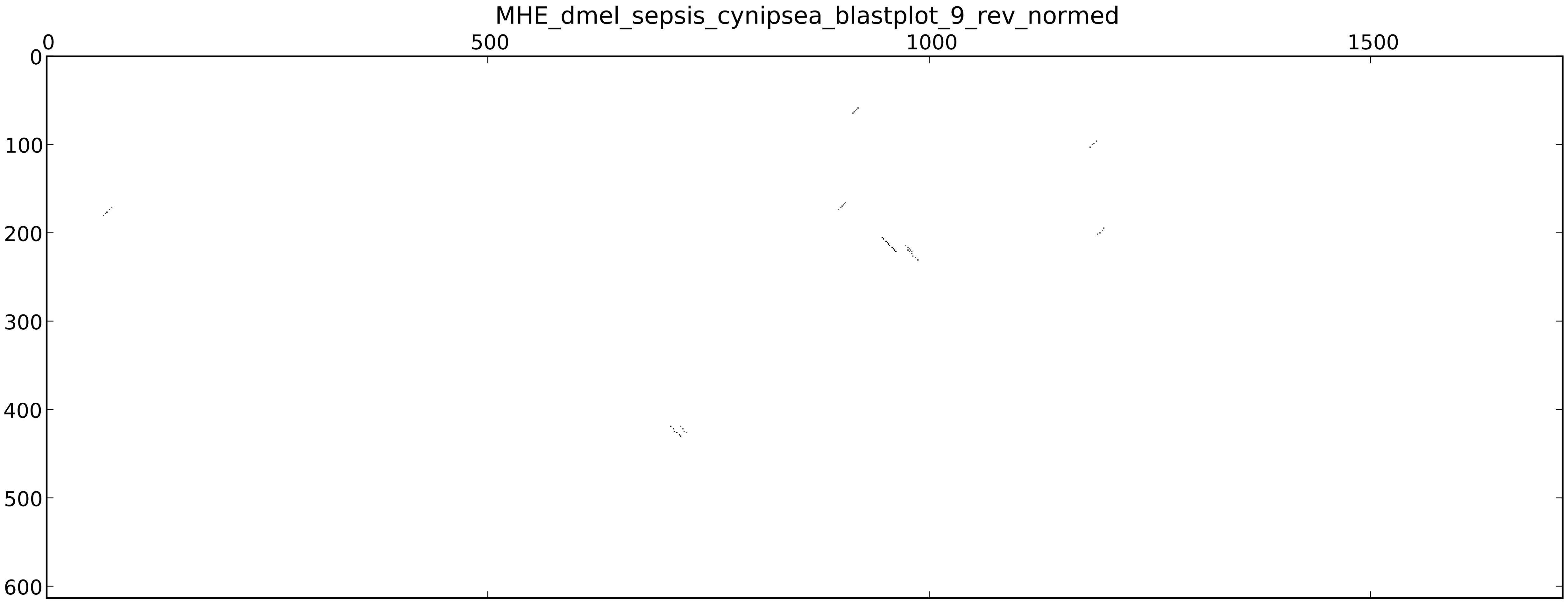
MHE_dmel_sepsis_cynipsea_blastplot_9_rev_normed
0
500
1000
1500
0
100
200
300
400
500
600

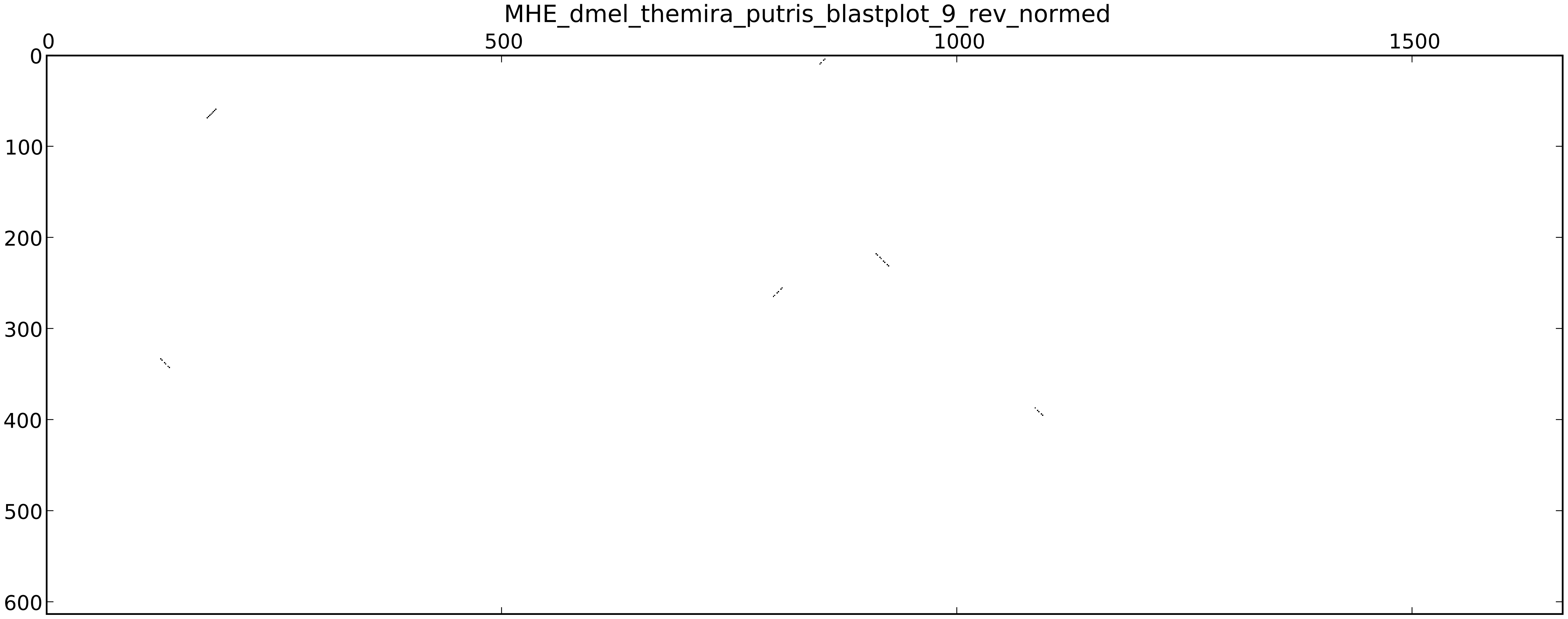
MHE_dmel_themira_putris_blastplot_9_rev_normed
0
500
1000
1500
0
100
200
300
400
500
600

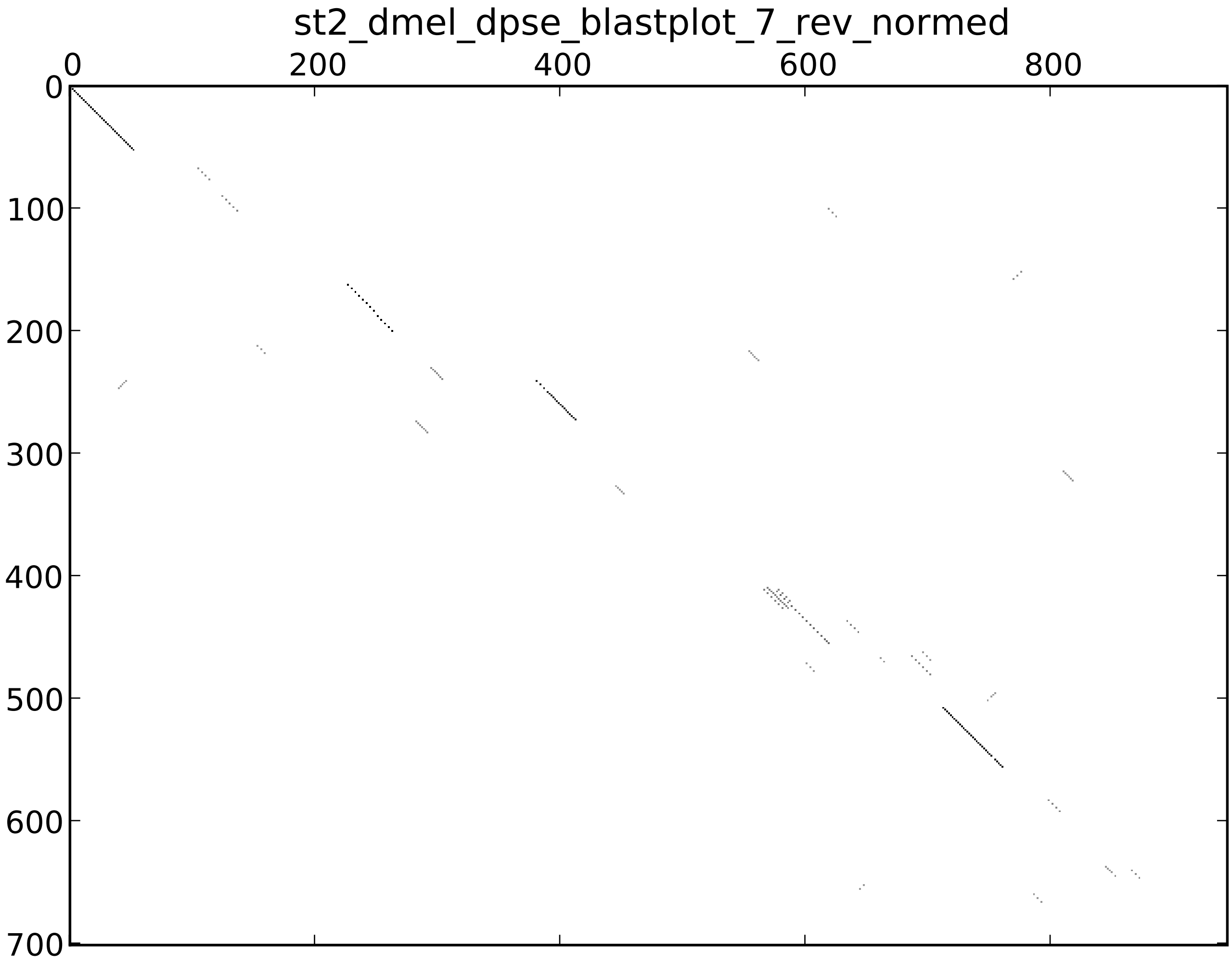
st2_dmel_dpse_blastplot_7_rev_normed
0
200
400
600
800
0
100
200
300
400
500
600
700

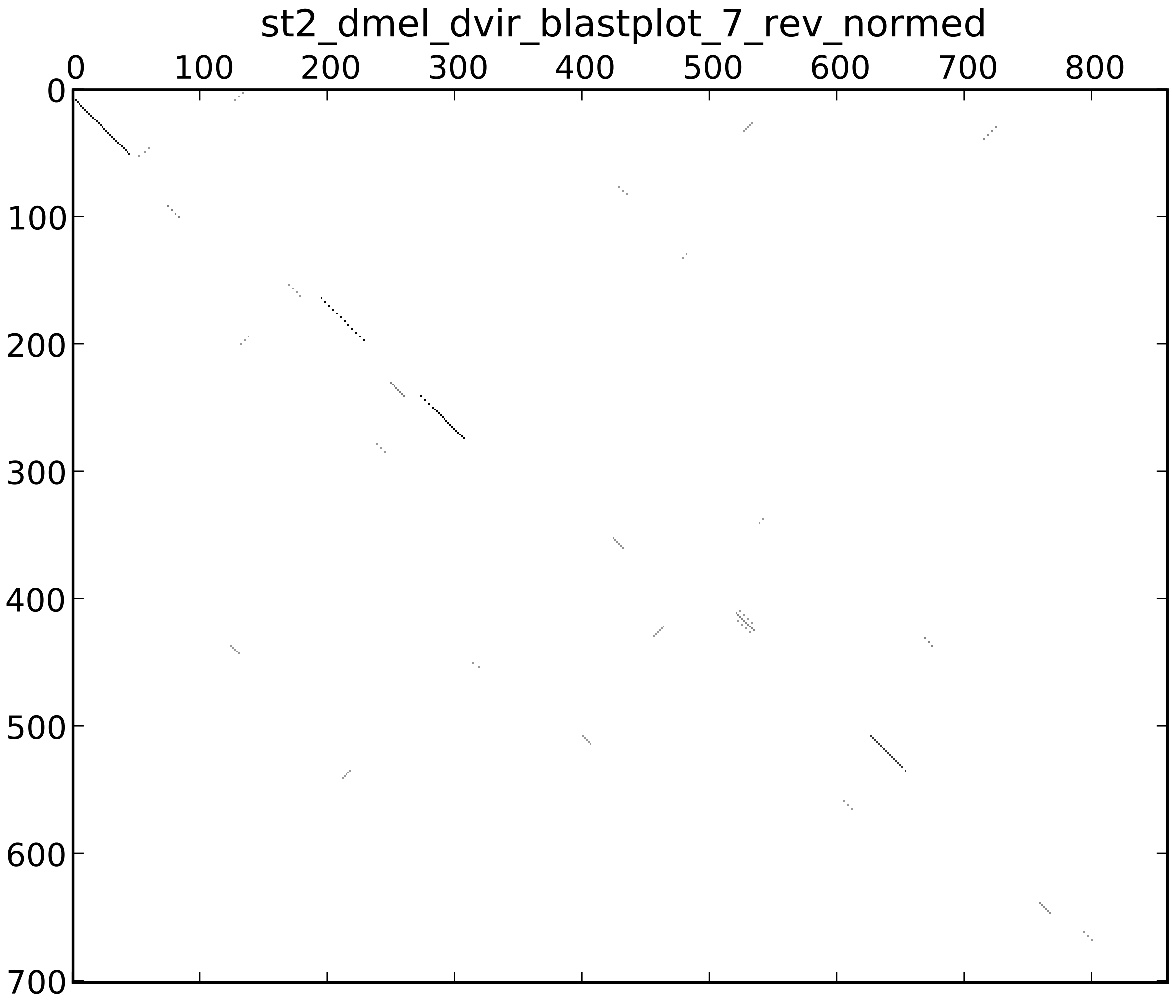

st2_dmel_dvir_blastplot_7_rev_normed
0
100
200
300
400
500
600
700
800
0
100
200
300
400
500
600
700

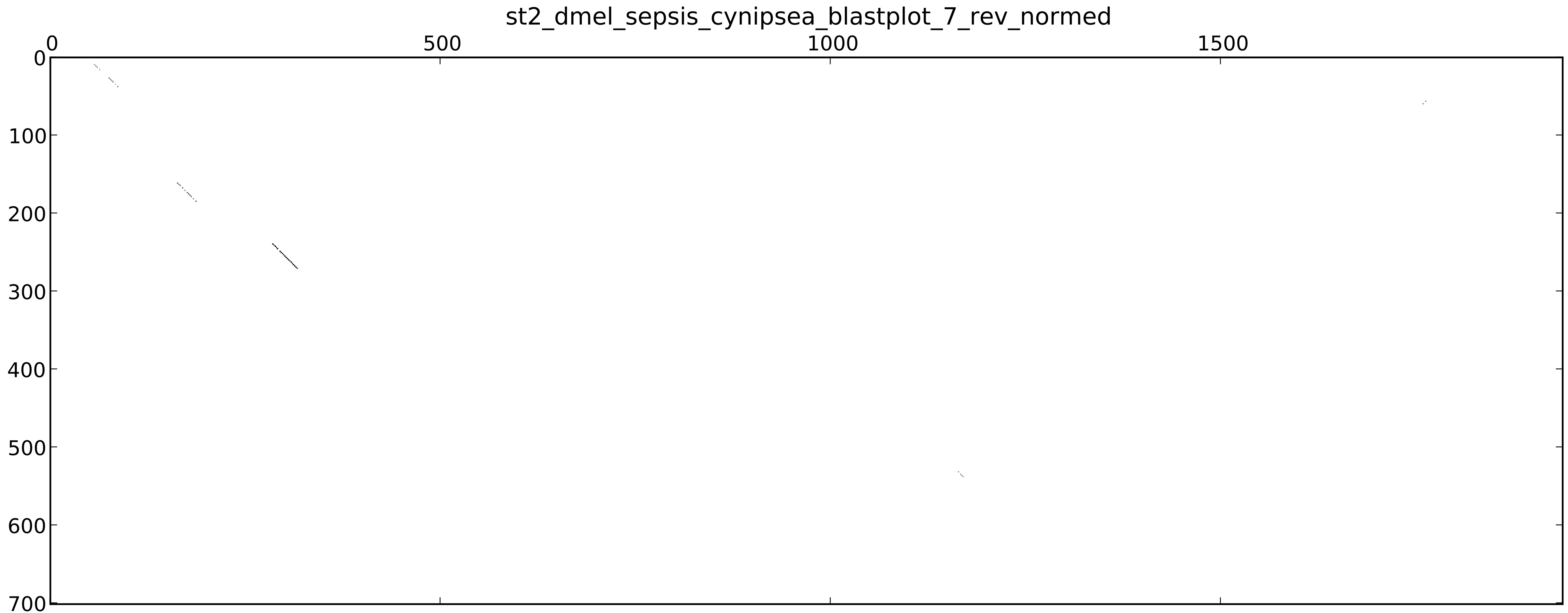
st2_dmel_sepsis_cynipsea_blastplot_7_rev_normed
0
500
1000
1500
0
100
200
300
400
500
600
700

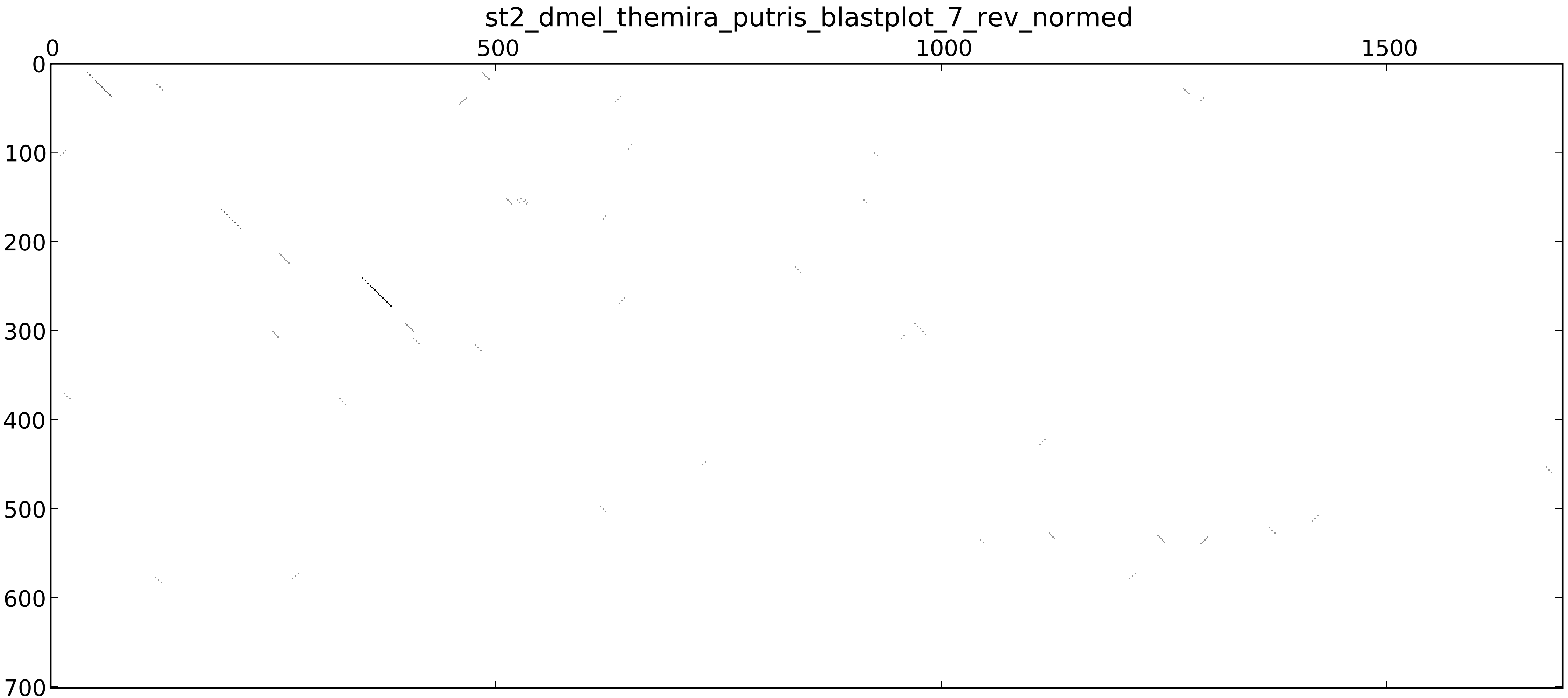

st2_dmel_themira_putris_blastplot_7_rev_normed
0
500
1000
1500
0
100
200
300
400
500
600
700

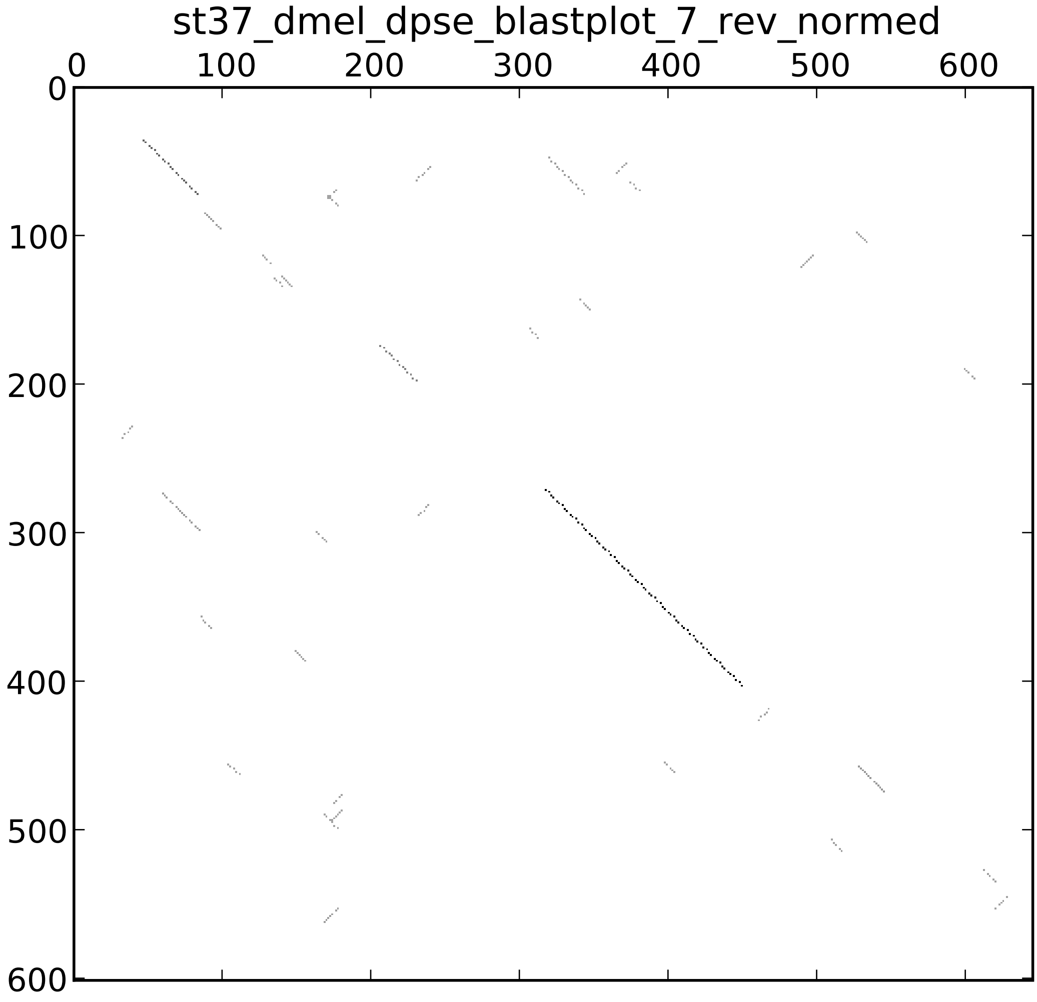
st37_dmel_dpse_blastplot_7_rev_normed
0
100
200
300
400
500
600
0
100
200
300
400
500
600

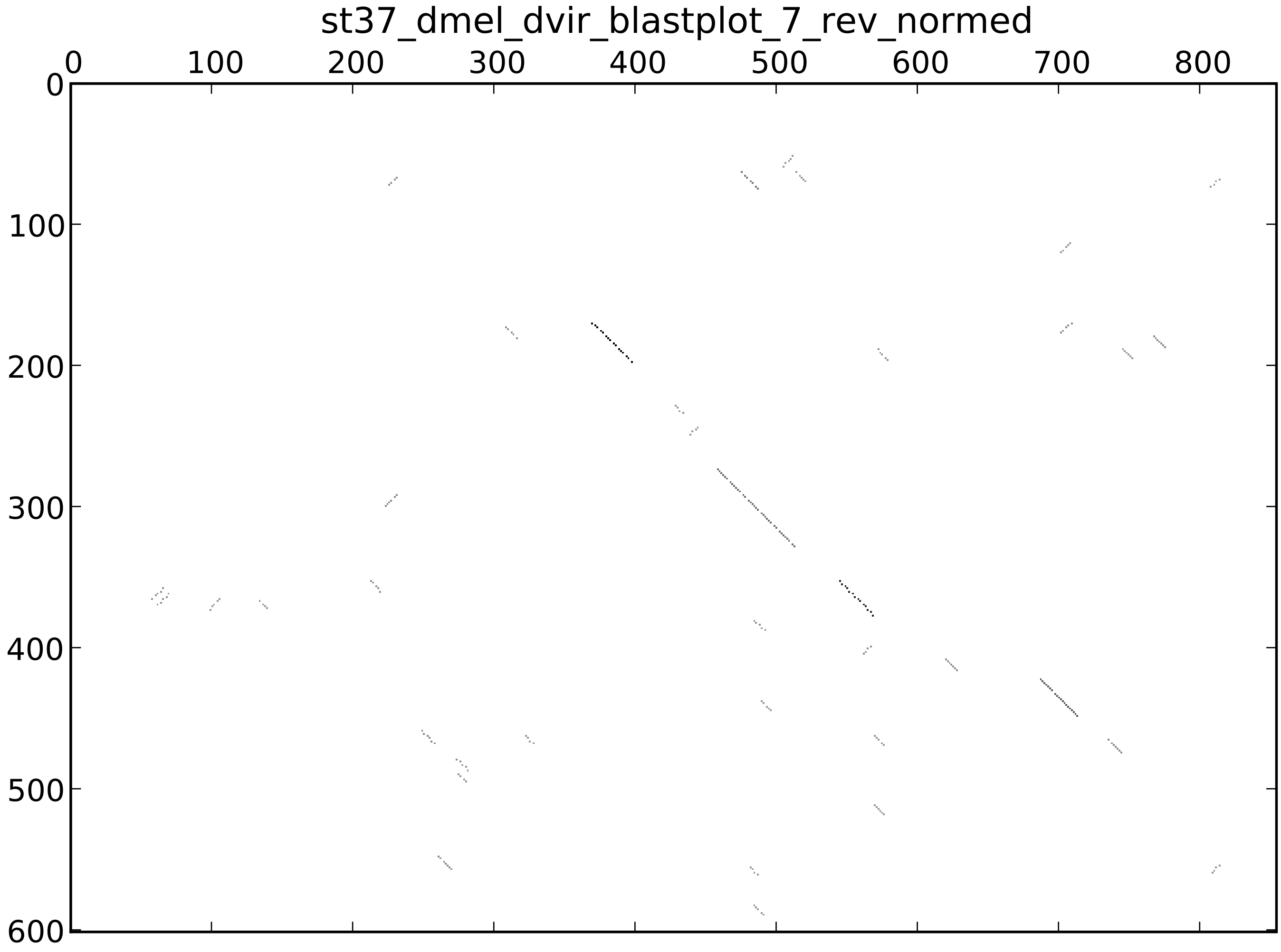
st37_dmel_dvir_blastplot_7_rev_normed
0
100
200
300
400
500
600
700
800
0
100
200
300
400
500
600

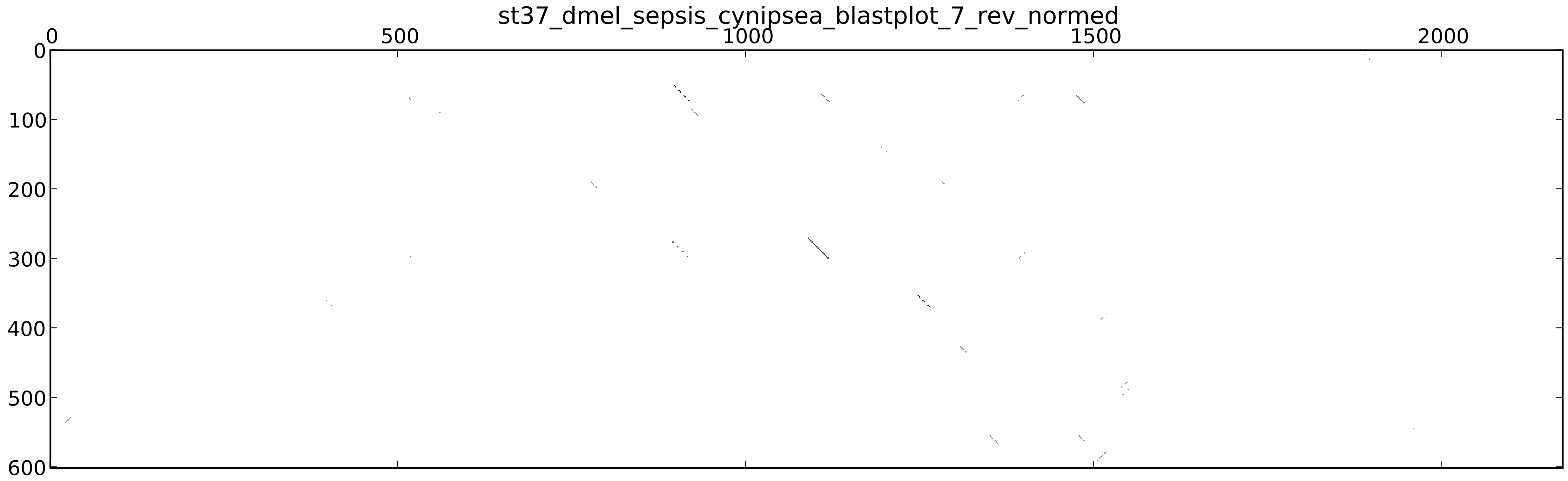
st37_dmel_sepsis_cynipsea_blastplot_7_rev_normed
0
500
1000
1500
2000
0
100
200
300
400
500
600

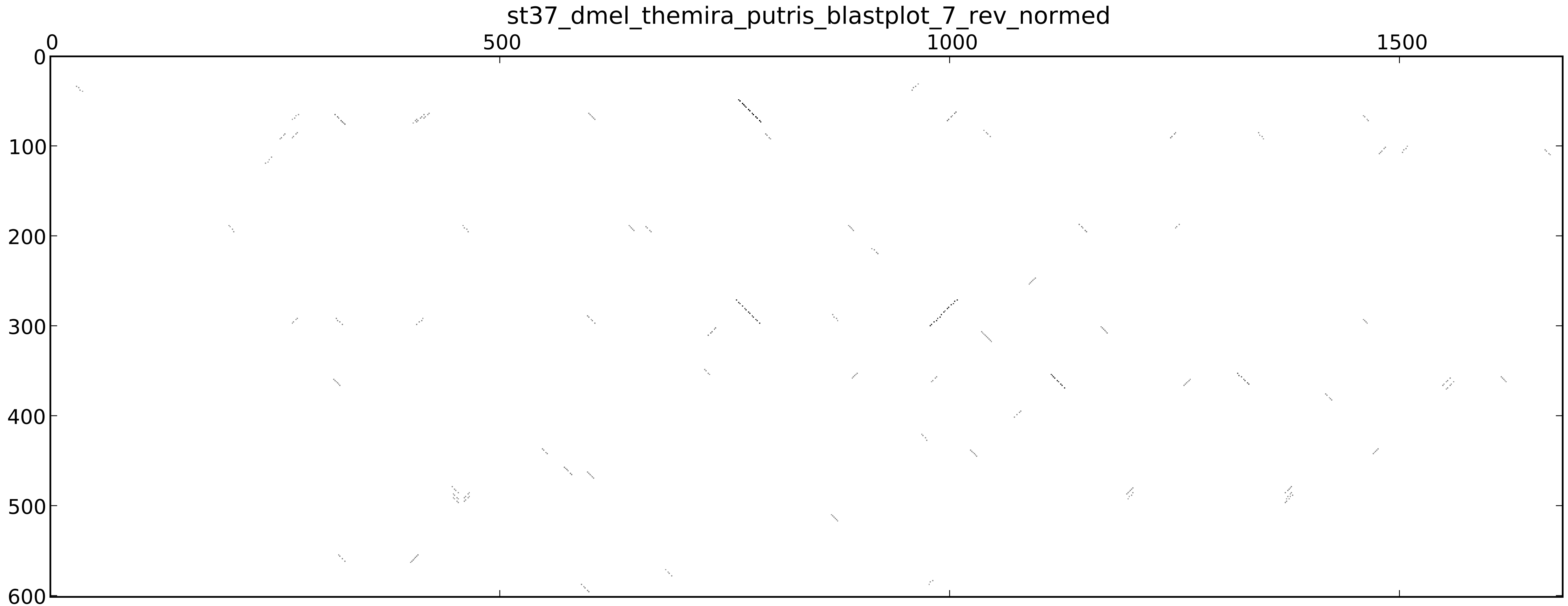
st37_dmel_themira_putris_blastplot_7_rev_normed
0
500
1000
1500
0
100
200
300
400
500
600

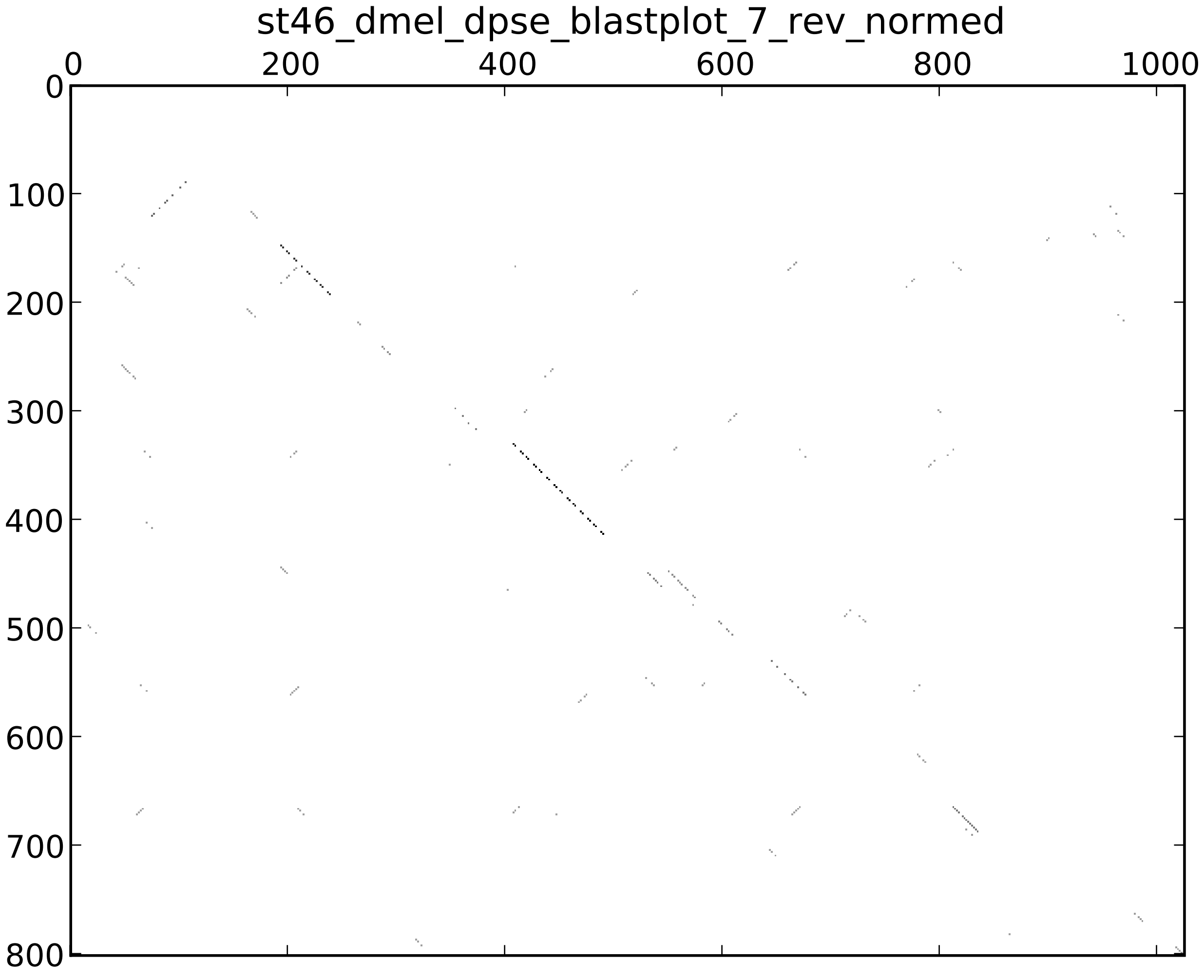

st46_dmel_dpse_blastplot_7_rev_normed
0
200
400
600
800
1000
0
100
200
300
400
500
600
700
800

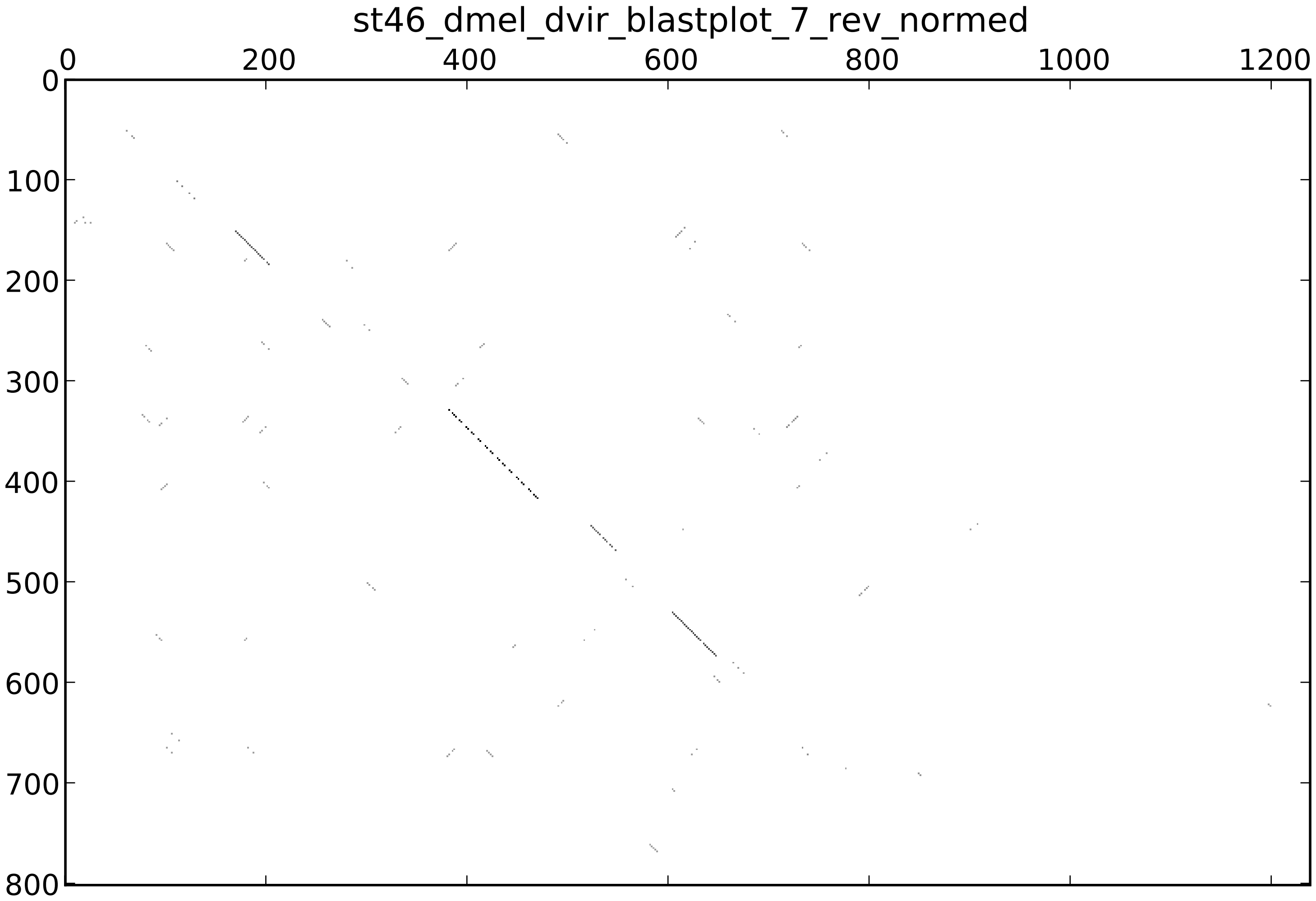
st46_dmel_dvir_blastplot_7_rev_normed
0
200
400
600
800
1000
1200
0
100
200
300
400
500
600
700
800

st46_dmel_sepsis_cynipsea_blastplot_7_rev_normed

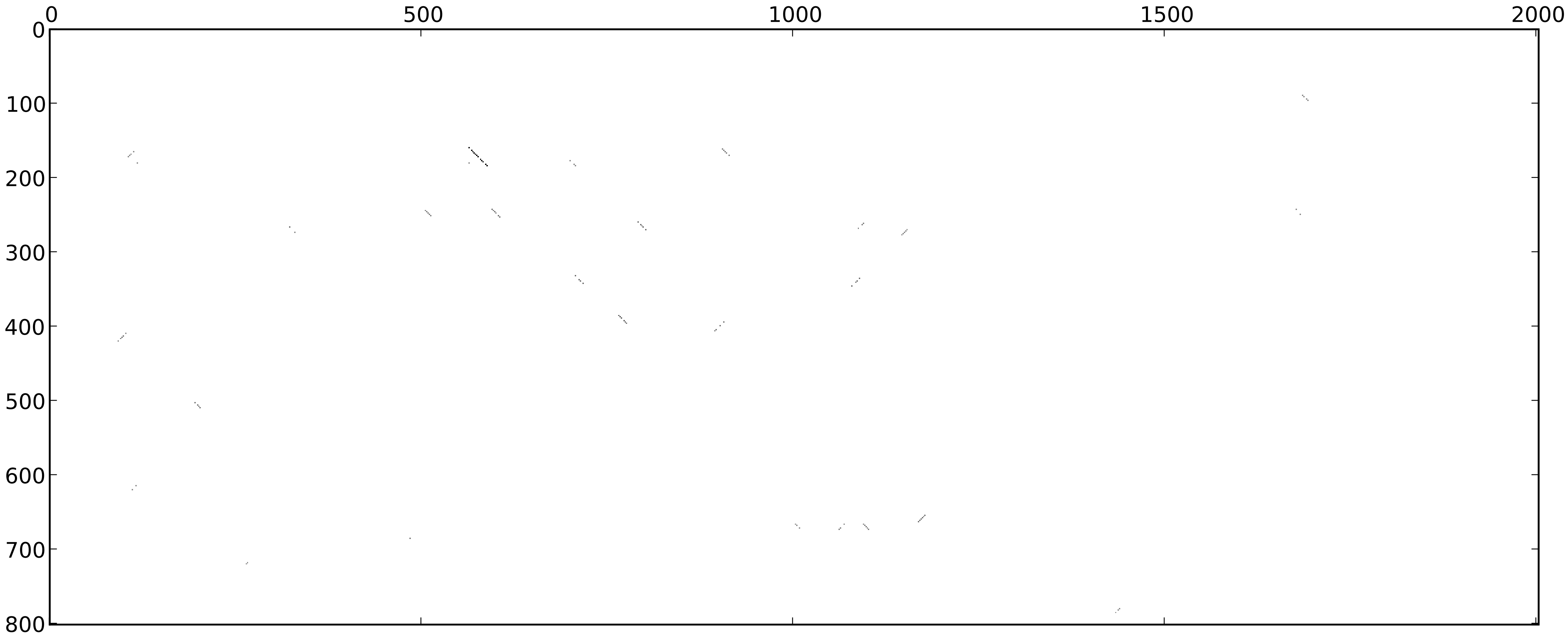

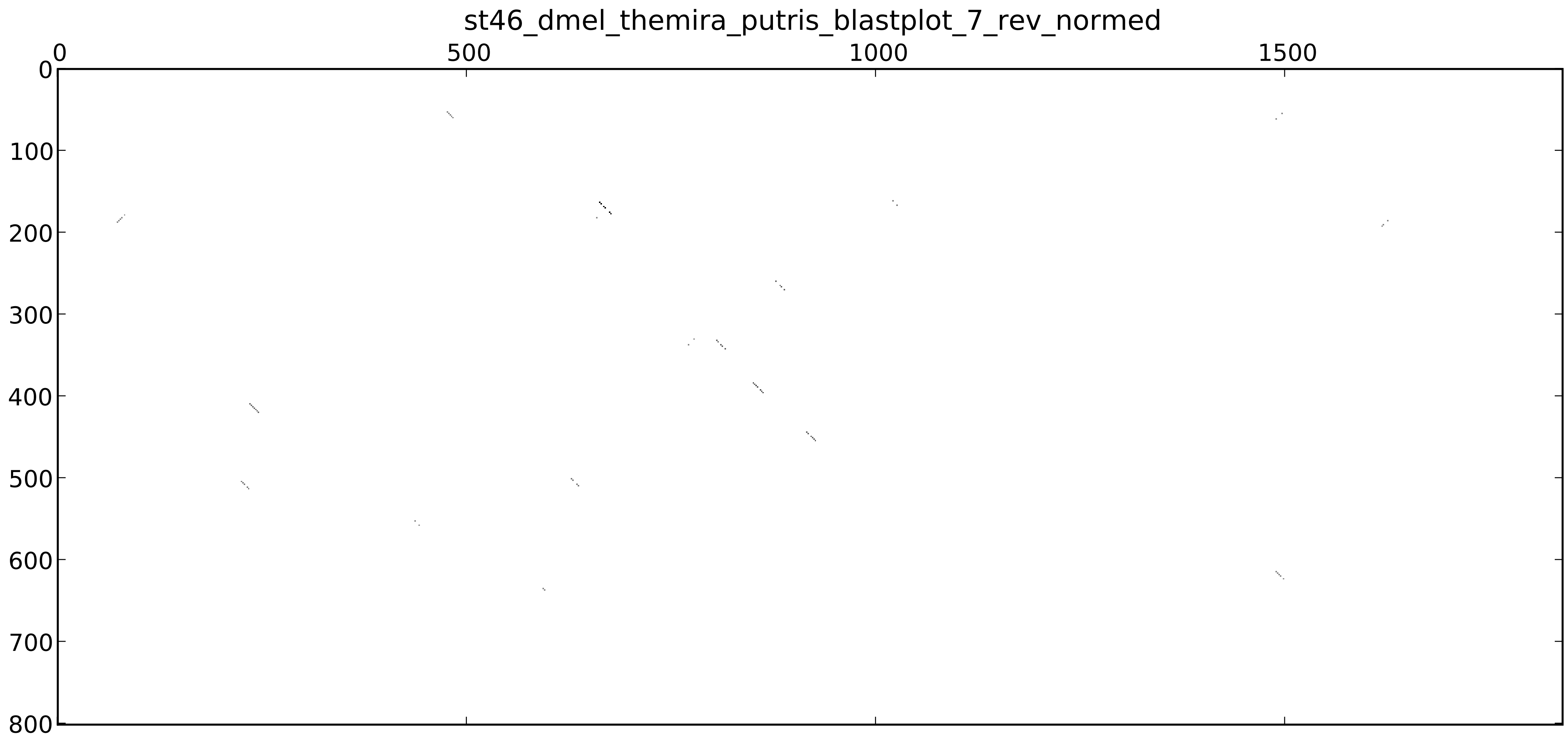
st46_dmel_themira_putris_blastplot_7_rev_normed
0
500
1000
1500
0
100
200
300
400
500
600
700
800

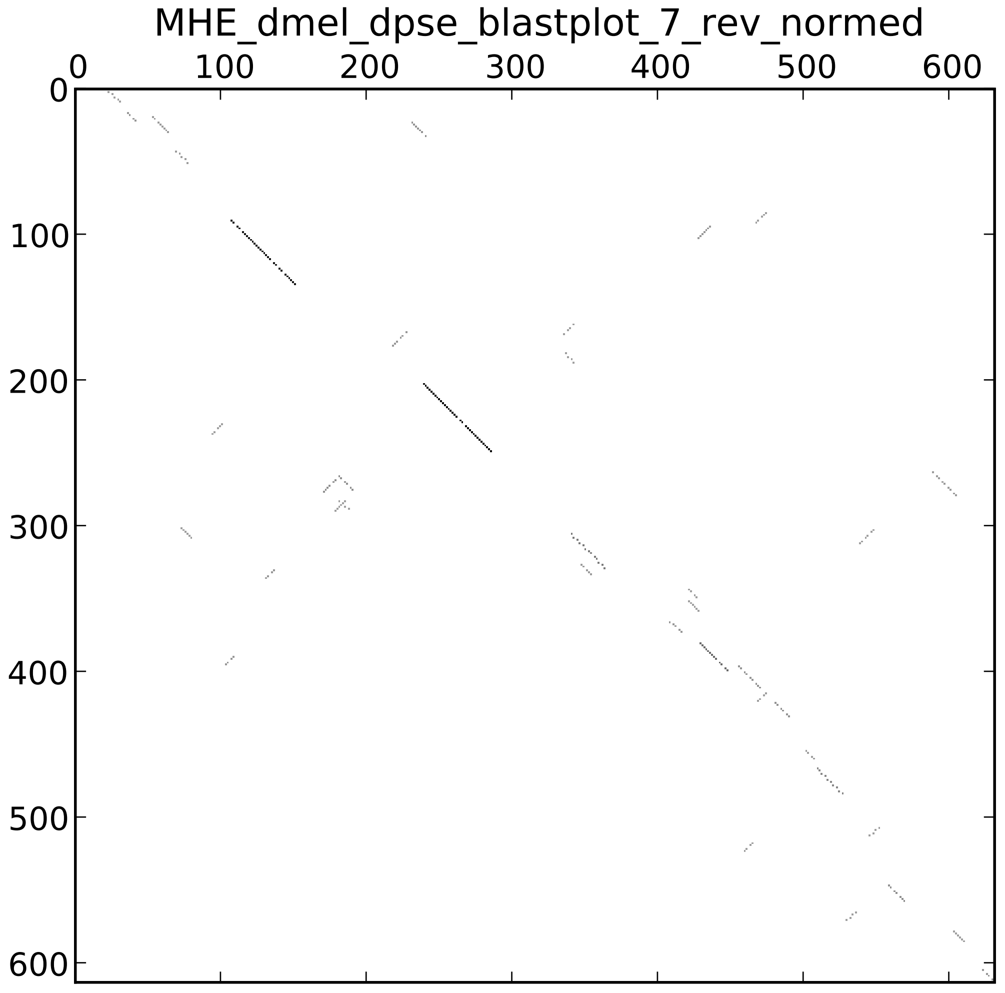

MHE_dmel_dpse_blastplot_7_rev_normed
0
100
200
300
400
500
600
0
100
200
300
400
500
600

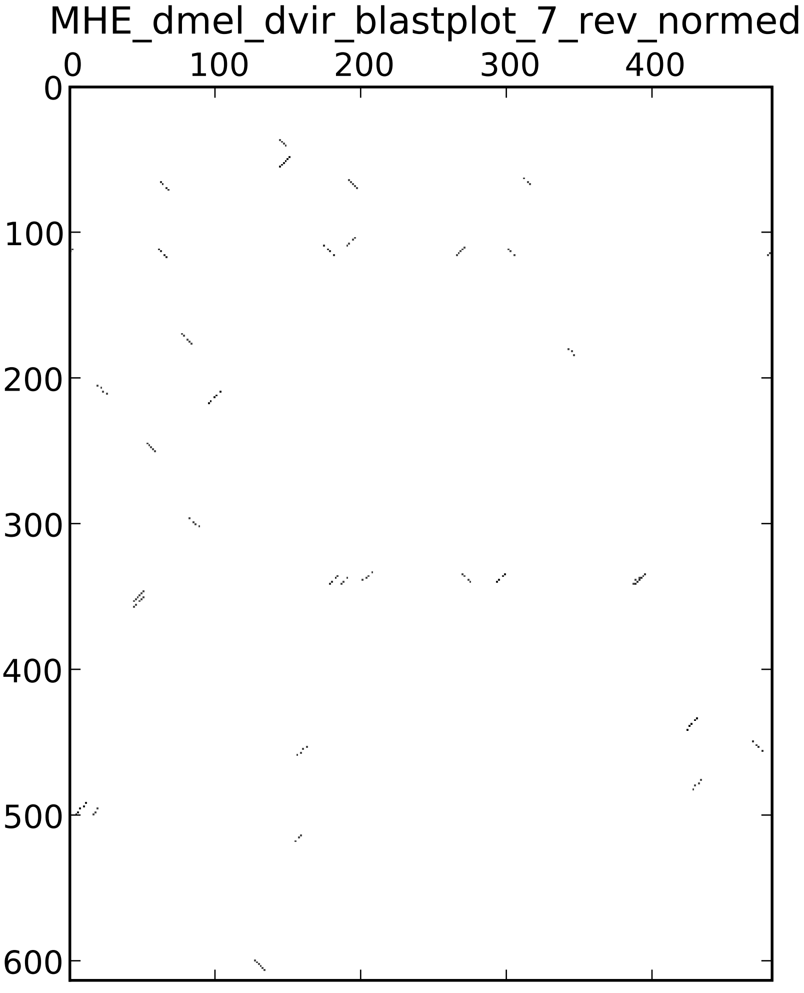
MHE_dmel_dvir_blastplot_7_rev_normed
0
100
200
300
400
0
100
200
300
400
500
600

MHE_dmel_sepsis_cynipsea_blastplot_7_rev_normed

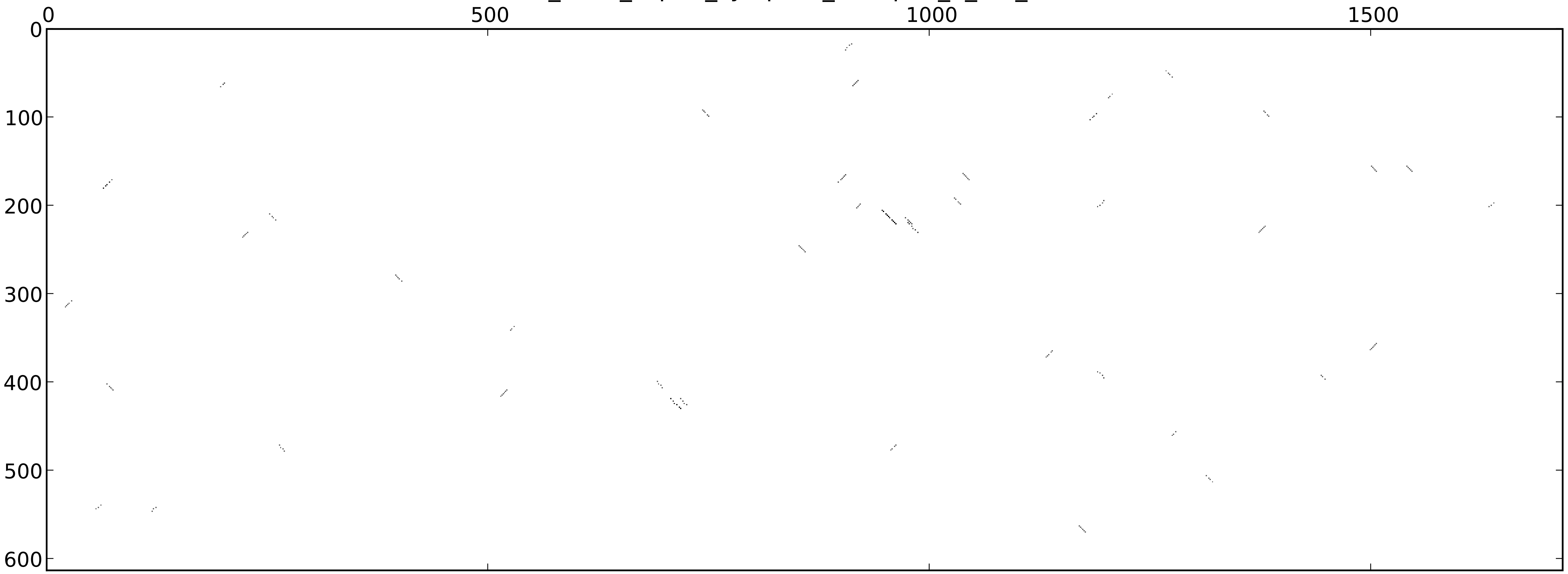

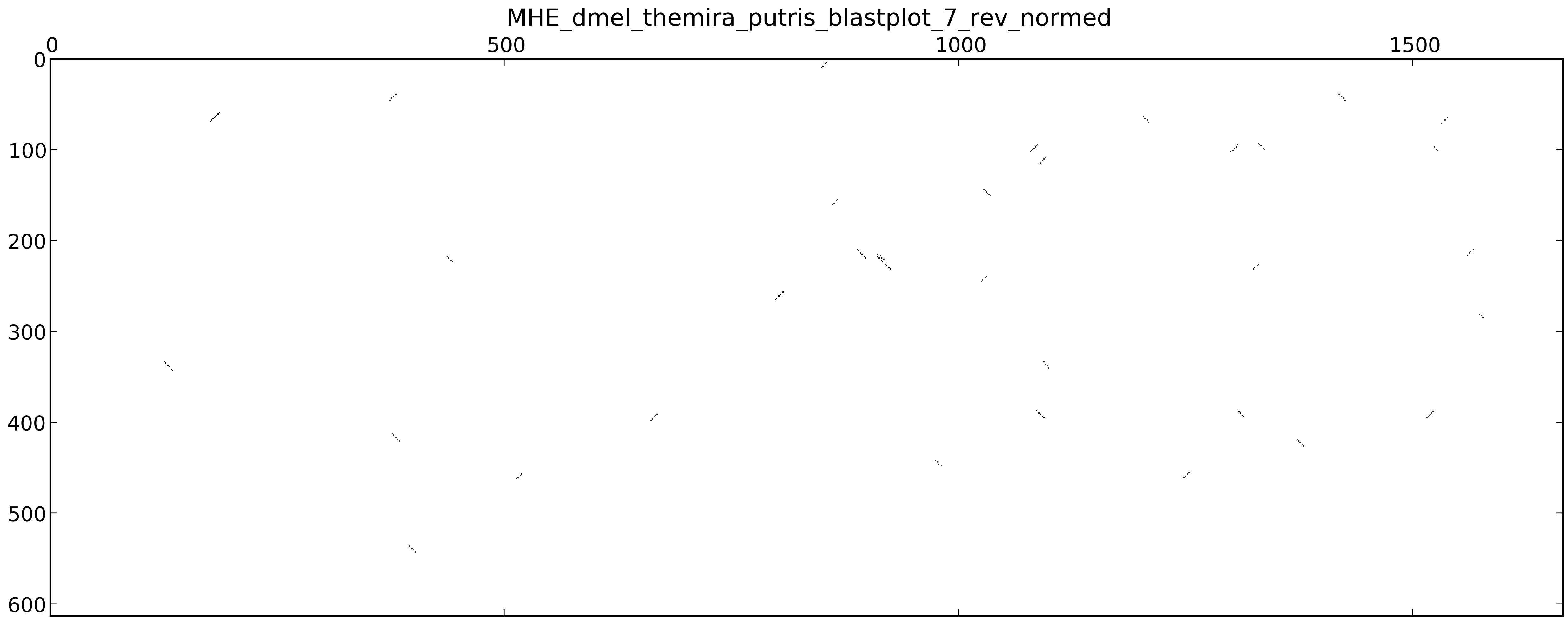
MHE_dmel_themira_putris_blastplot_7_rev_normed
0
500
1000
1500
0
100
200
300
400
500
600

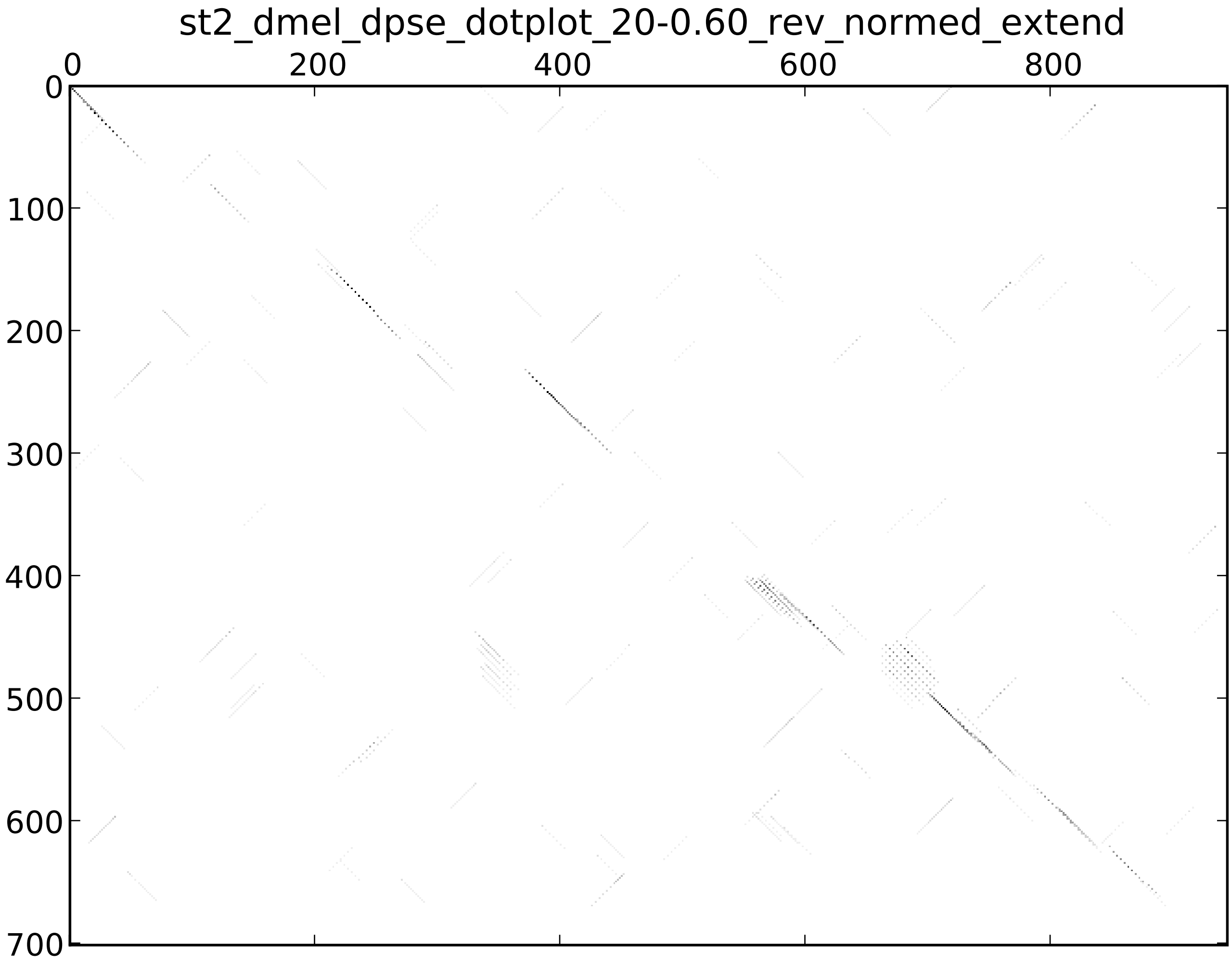
st2_dmel_dpse_dotplot_20-0.60_rev_normed_extend
0
200
400
600
800
0
100
200
300
400
500
600
700

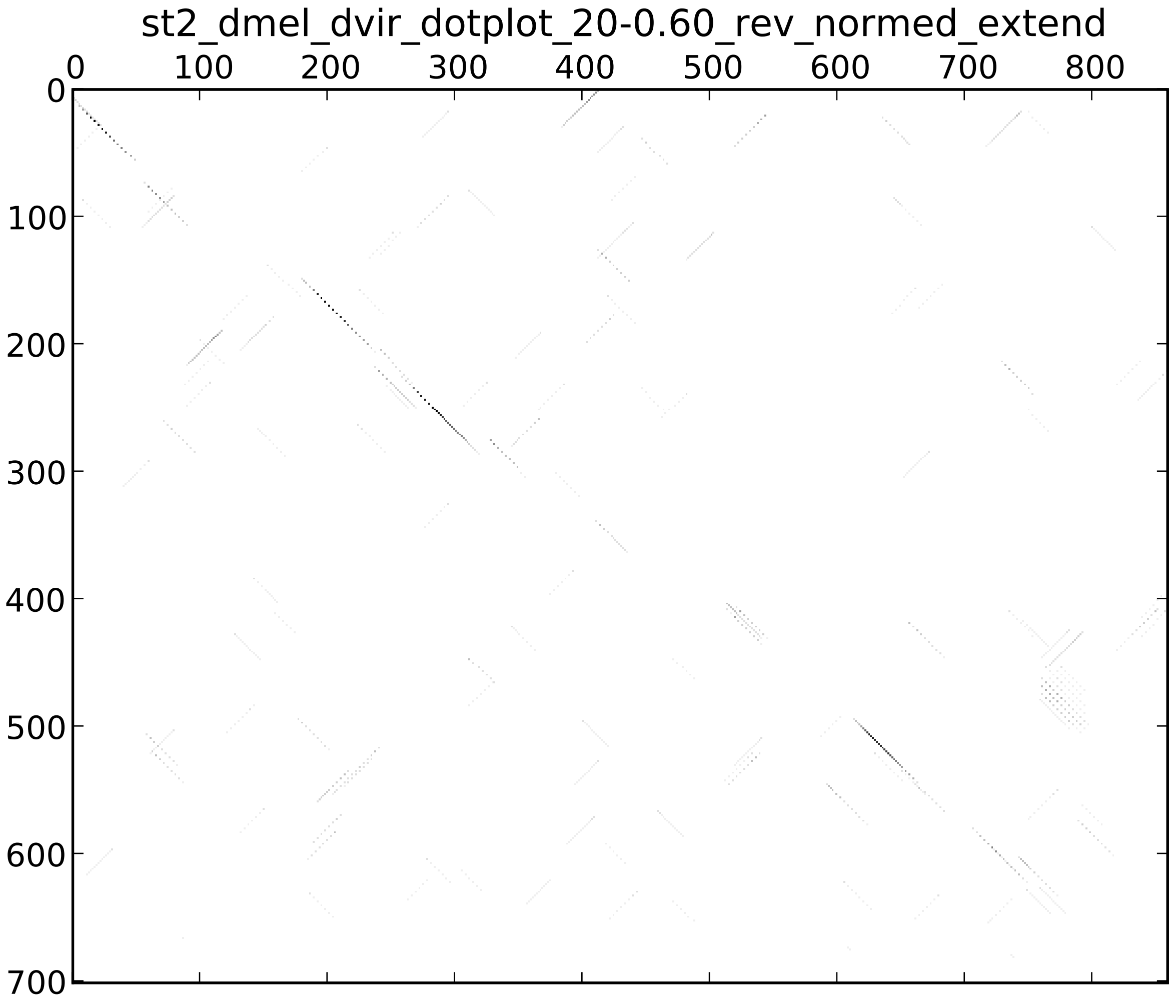
st2_dmel_dvir_dotplot_20-0.60_rev_normed_extend
0
100
200
300
400
500
600
700
800
0
100
200
300
400
500
600
700

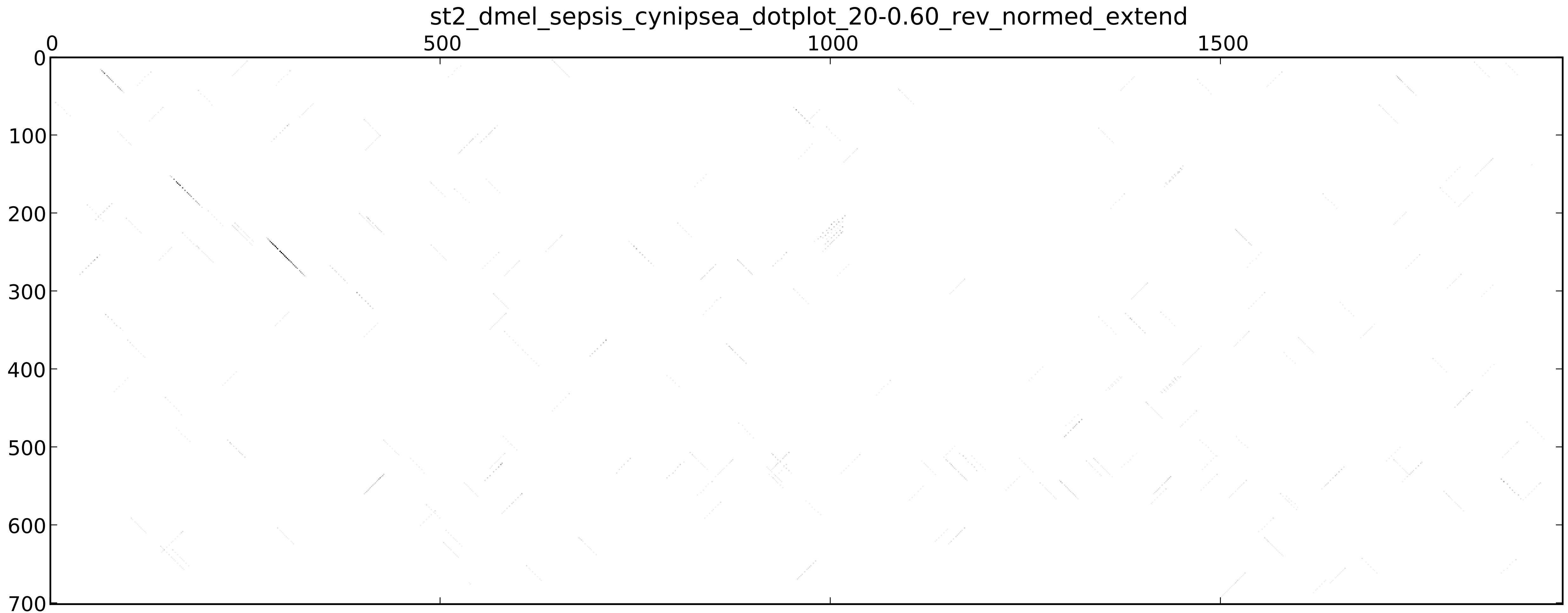
st2_dmel_sepsis_cynipsea_dotplot_20-0.60_rev_normed_extend
0
500
1000
1500
0
100
200
300
400
500
600
700

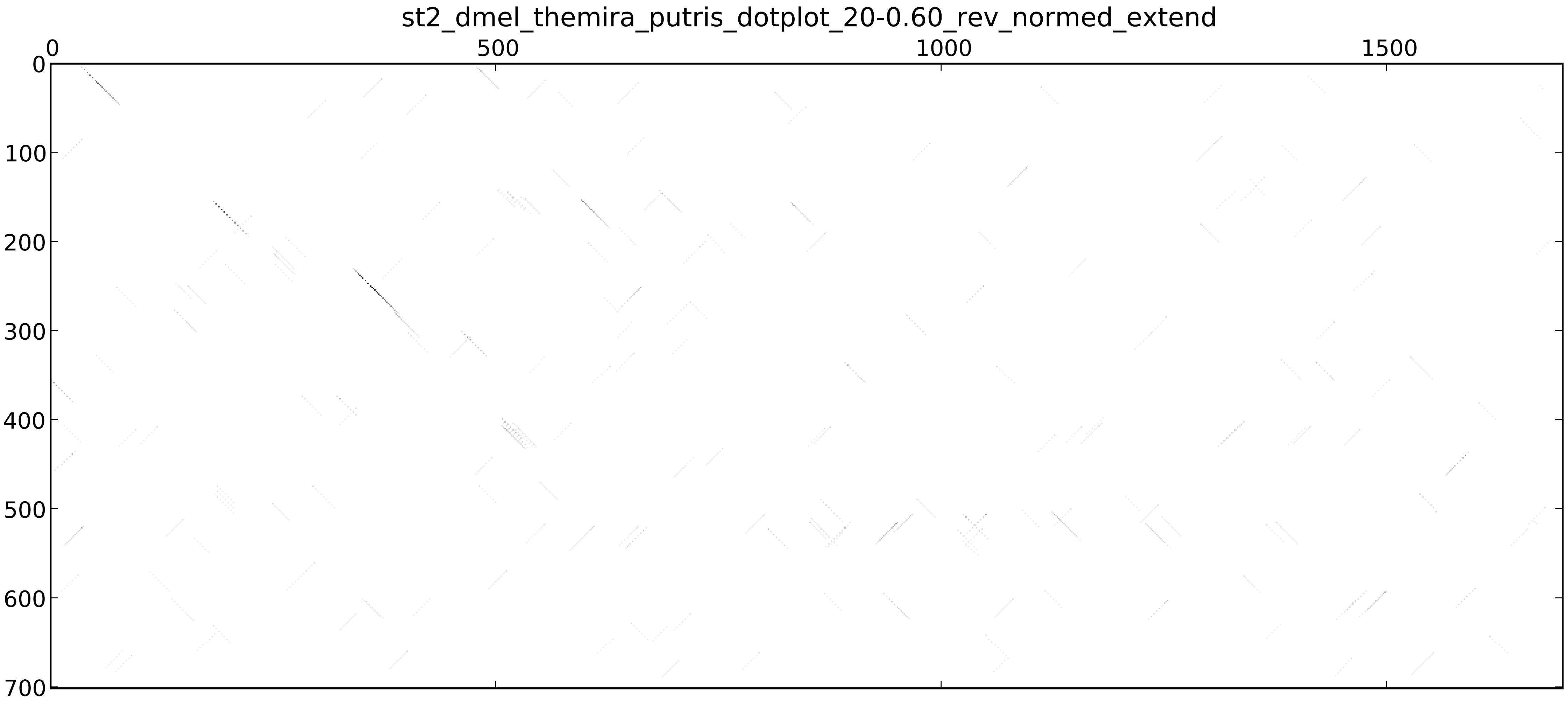
st2_dmel_themira_putris_dotplot_20-0.60_rev_normed_extend
0
500
1000
1500
0
100
200
300
400
500
600
700

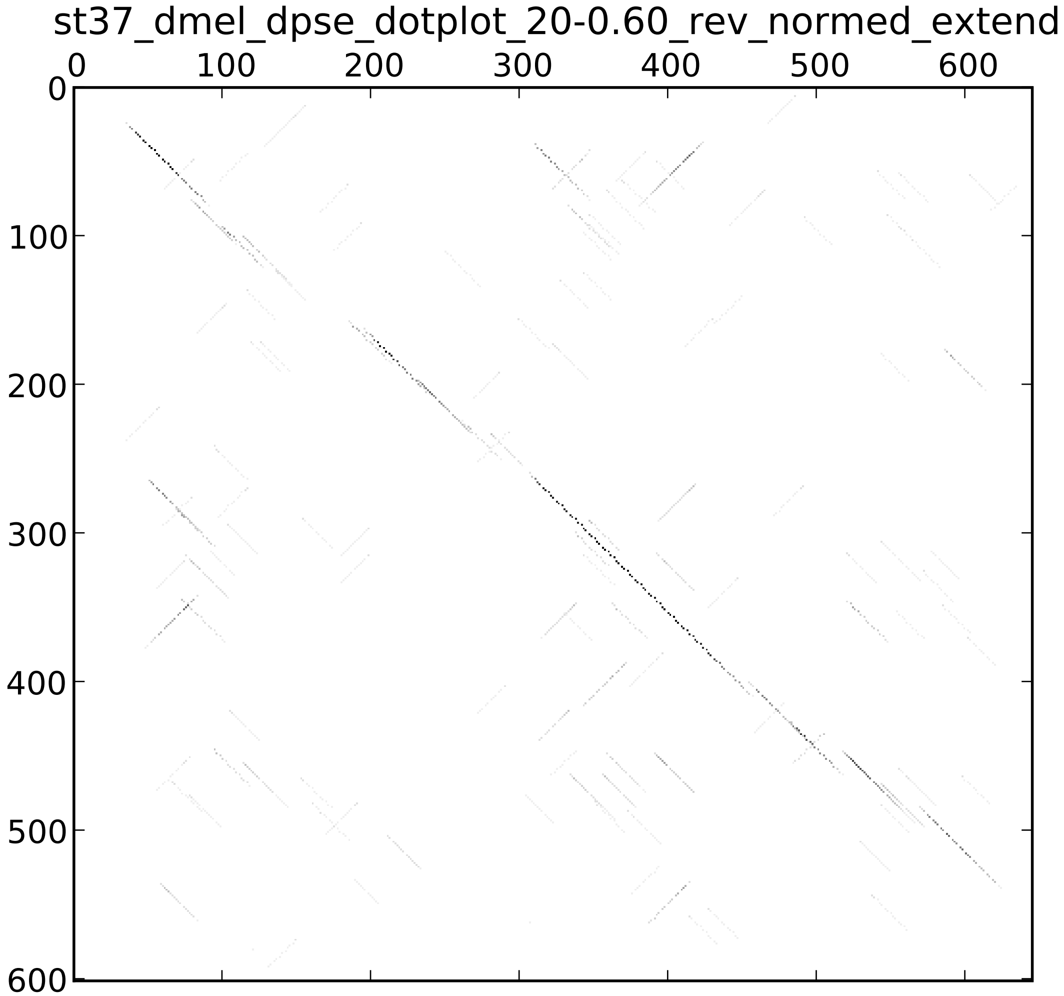
st37_dmel_dpse_dotplot_20-0.60_rev_normed_extend
0
100
200
300
400
500
600
0
100
200
300
400
500
600

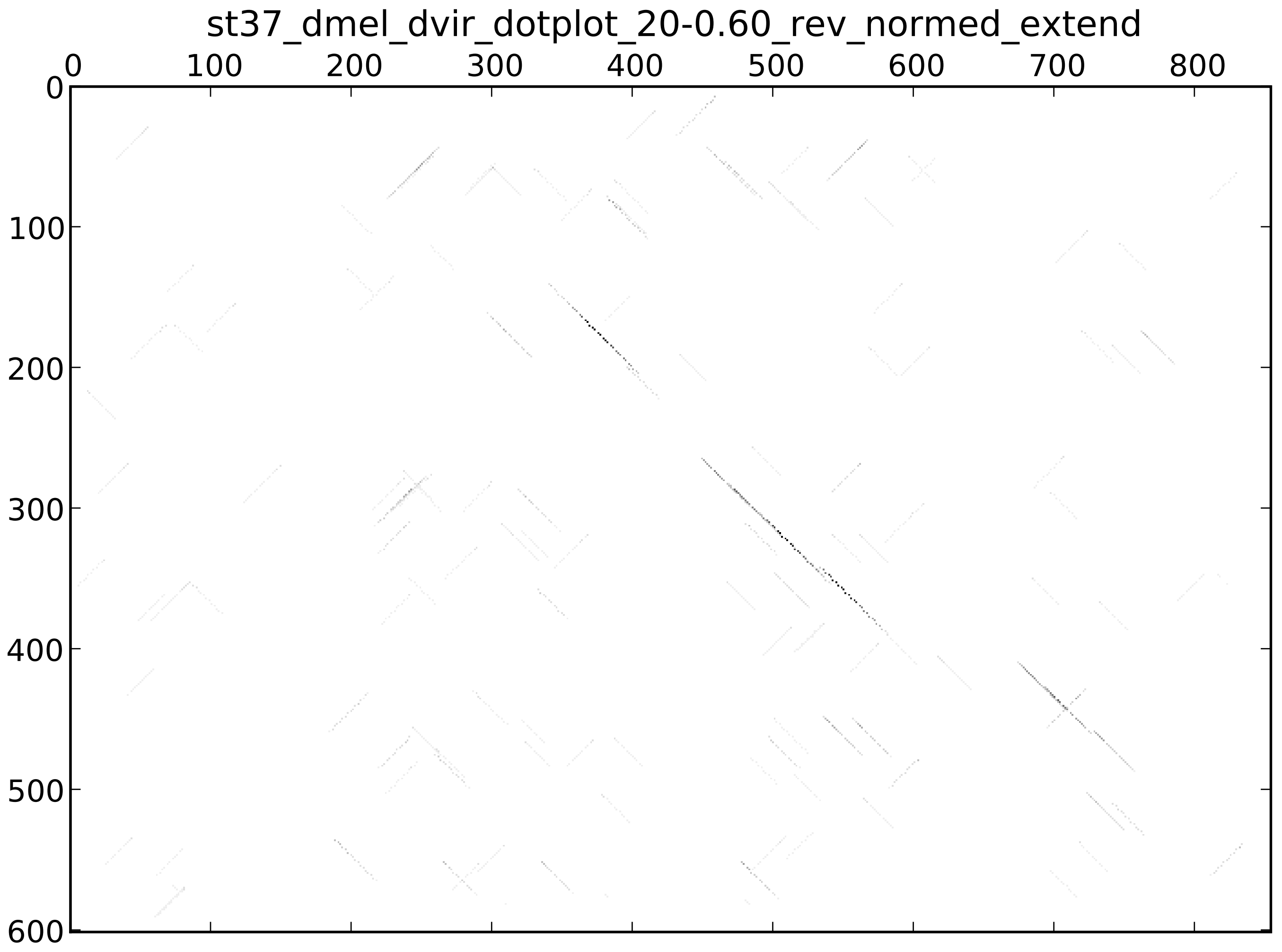
st37_dmel_dvir_dotplot_20-0.60_rev_normed_extend
0
100
200
300
400
500
600
700
800
0
100
200
300
400
500
600

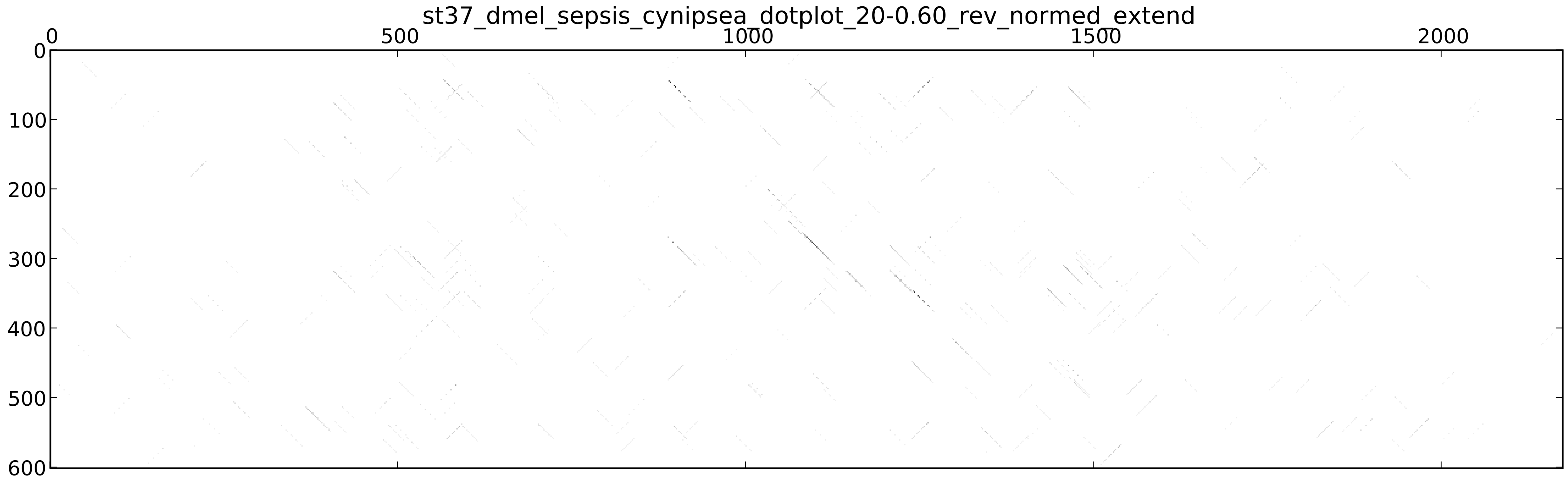

st37_dmel_sepsis_cynipsea_dotplot_20-0.60_rev_normed_extend
0
500
1000
1500
2000
0
100
200
300
400
500
600

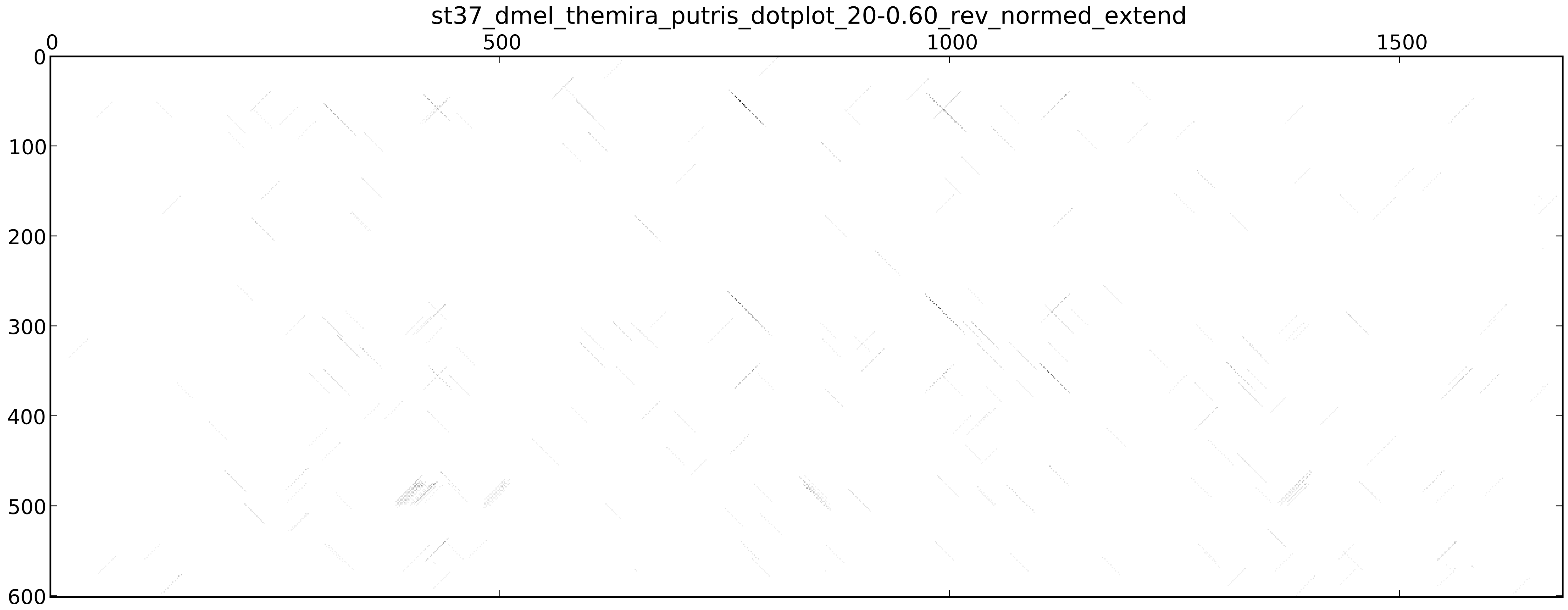
st37_dmel_themira_putris_dotplot_20-0.60_rev_normed_extend
0
500
1000
1500
0
100
200
300
400
500
600

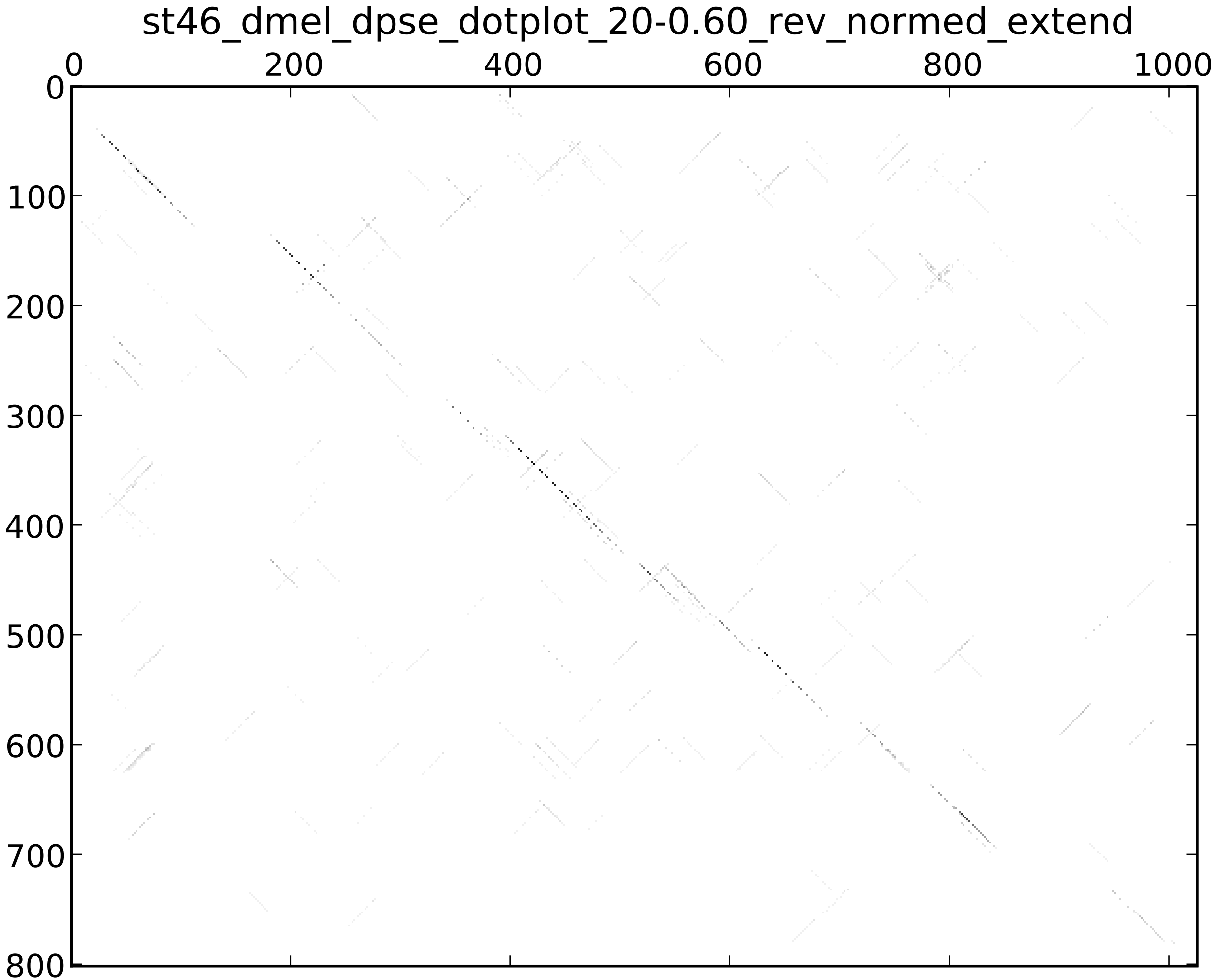

st46_dmel_dpse_dotplot_20-0.60_rev_normed_extend
0
200
400
600
800
1000
0
100
200
300
400
500
600
700
800

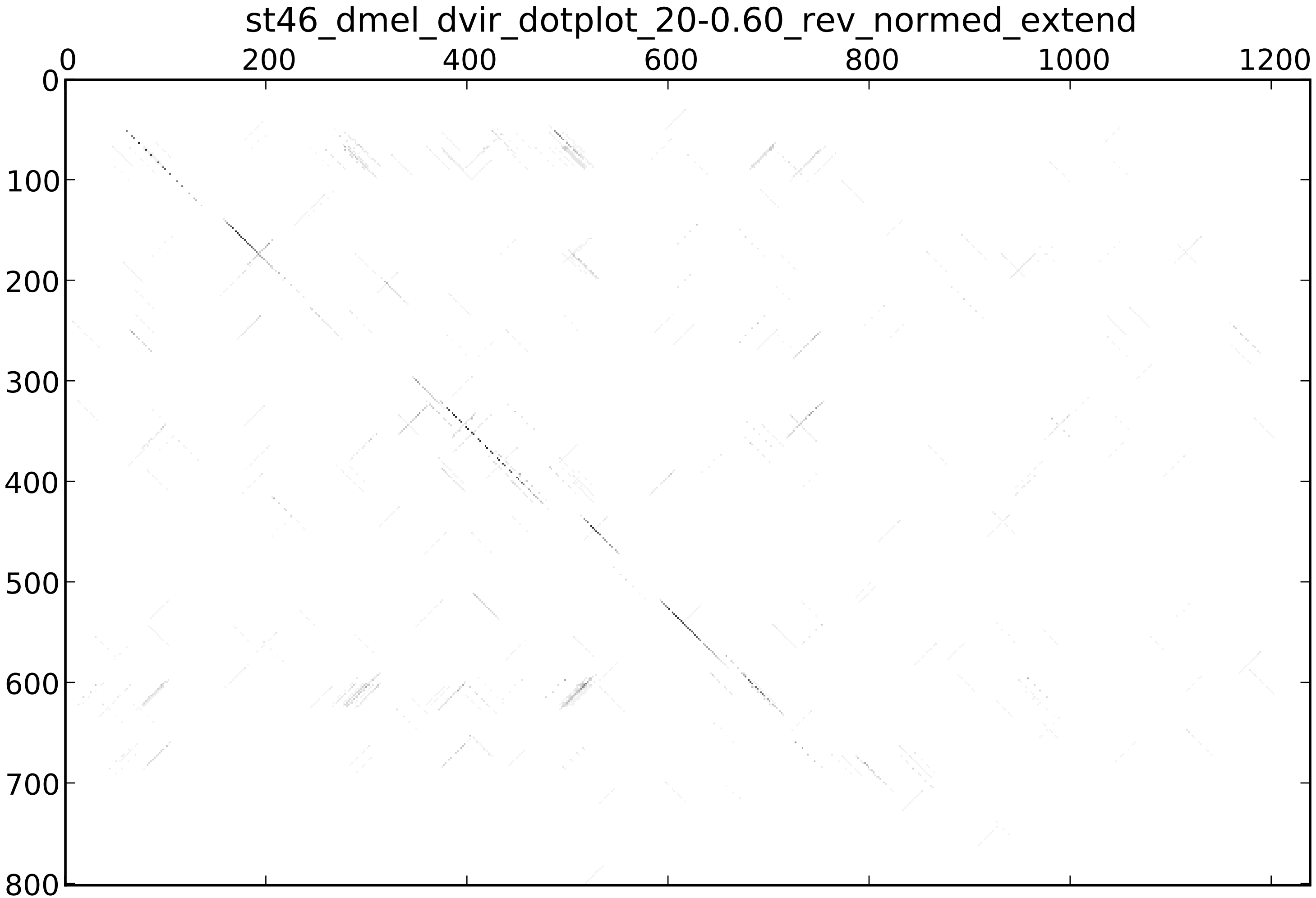

st46_dmel_dvir_dotplot_20-0.60_rev_normed_extend
0
200
400
600
800
1000
1200
0
100
200
300
400
500
600
700
800

st46_dmel_sepsis_cynipsea_dotplot_20-0.60_rev_normed_extend

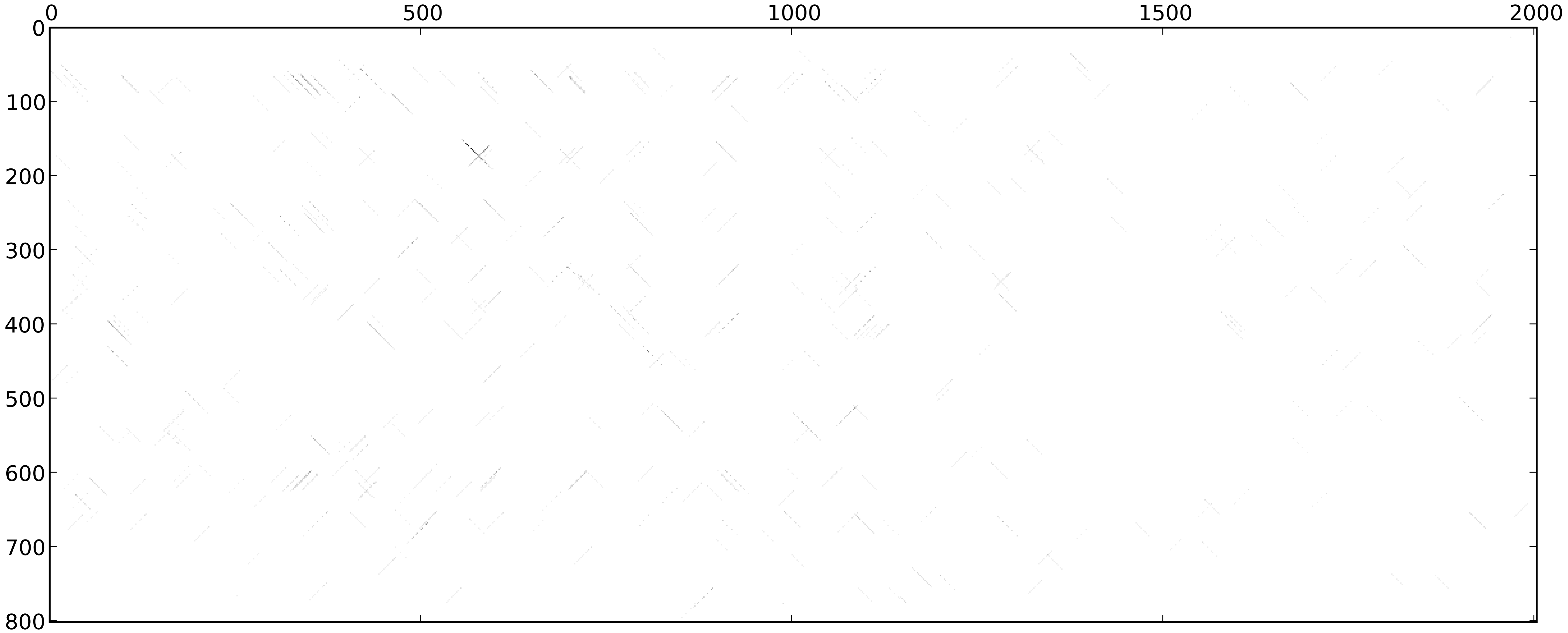

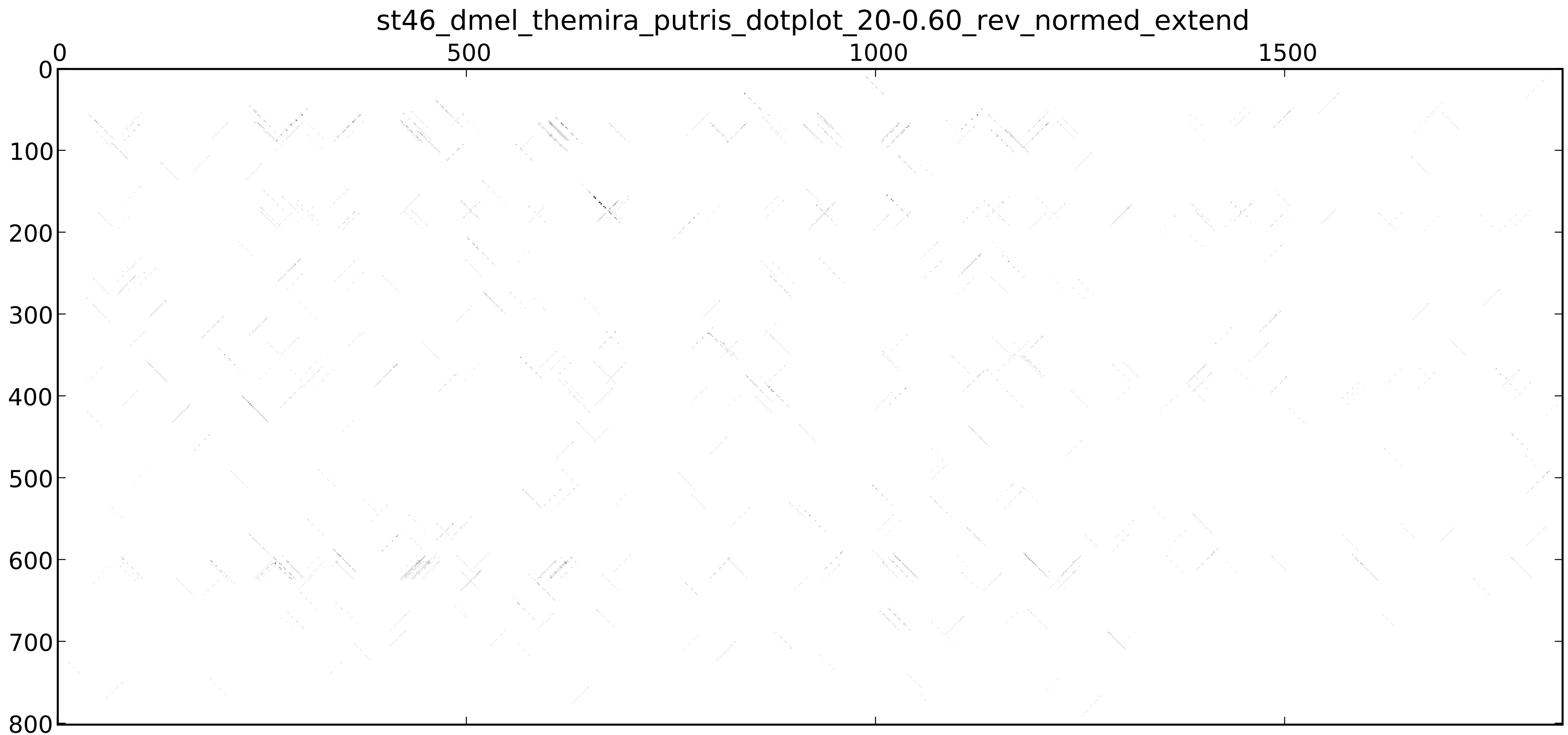

st46_dmel_themira_putris_dotplot_20-0.60_rev_normed_extend
0
500
1000
1500
0
100
200
300
400
500
600
700
800

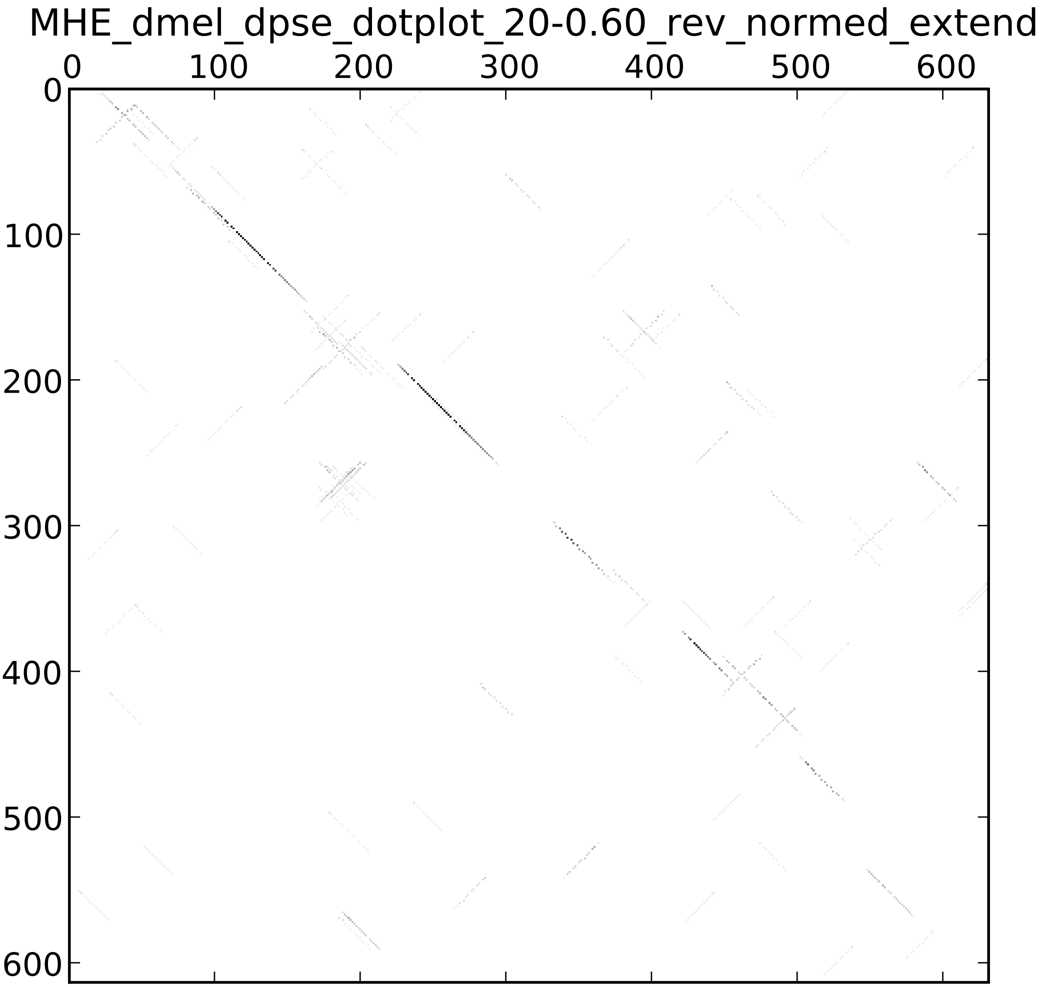
MHE_dmel_dpse_dotplot_20-0.60_rev_normed_extend
0
100
200
300
400
500
600
0
100
200
300
400
500
600

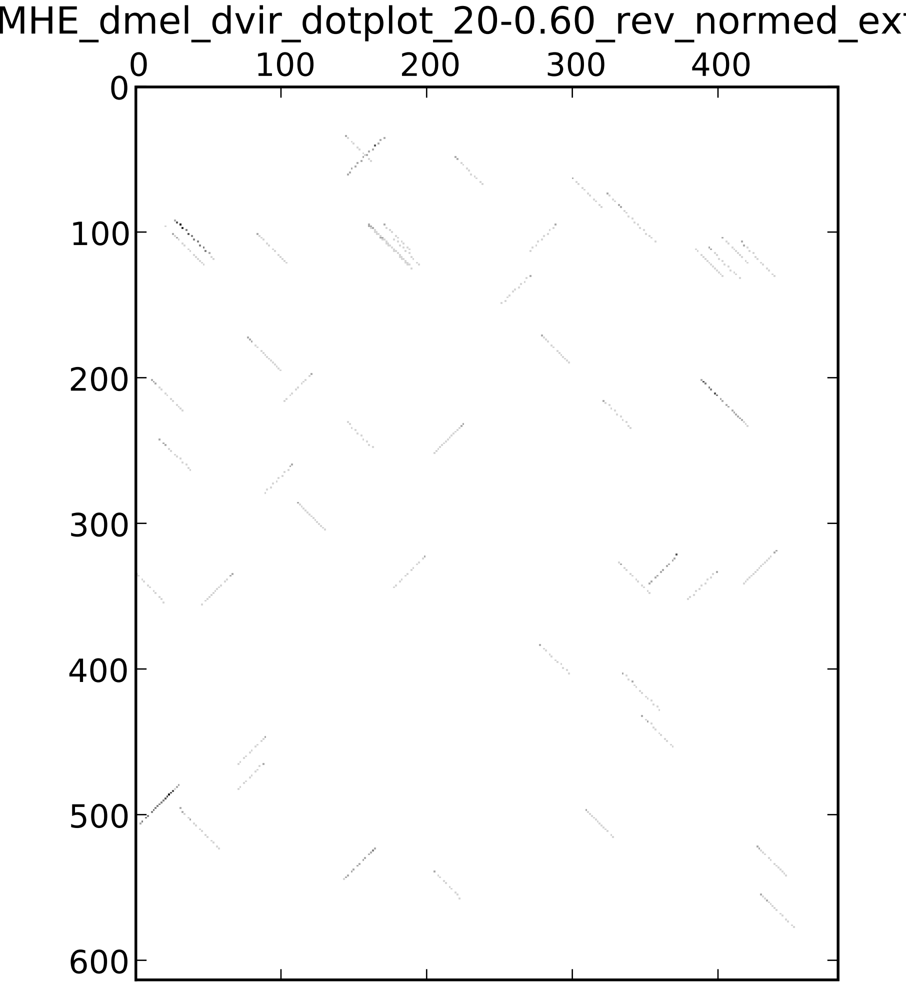
MHE_dmel_dvir_dotplot_20-0.60_rev_normed_ext
0
100
200
300
400
0
100
200
300
400
500
600

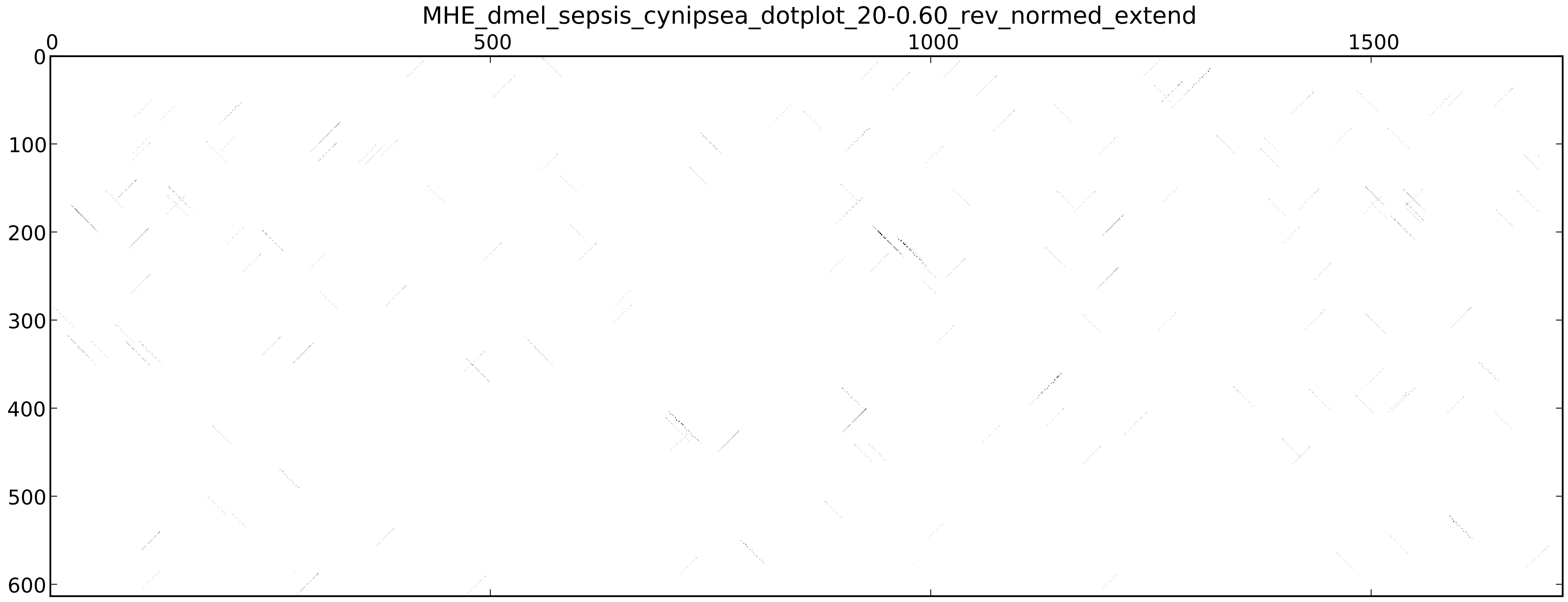
MHE_dmel_sepsis_cynipsea_dotplot_20-0.60_rev_normed_extend
0
500
1000
1500
0
100
200
300
400
500
600

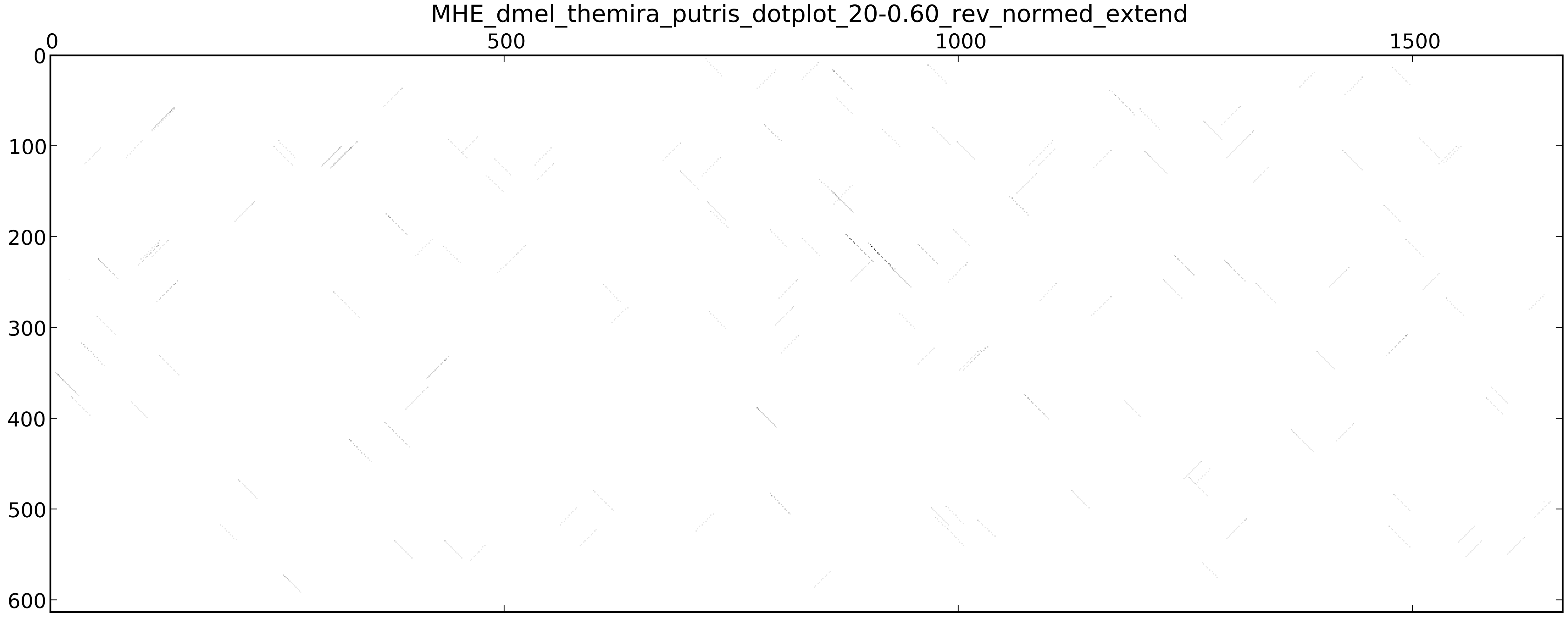
MHE_dmel_themira_putris_dotplot_20-0.60_rev_normed_extend
0
500
1000
1500
0
100
200
300
400
500
600

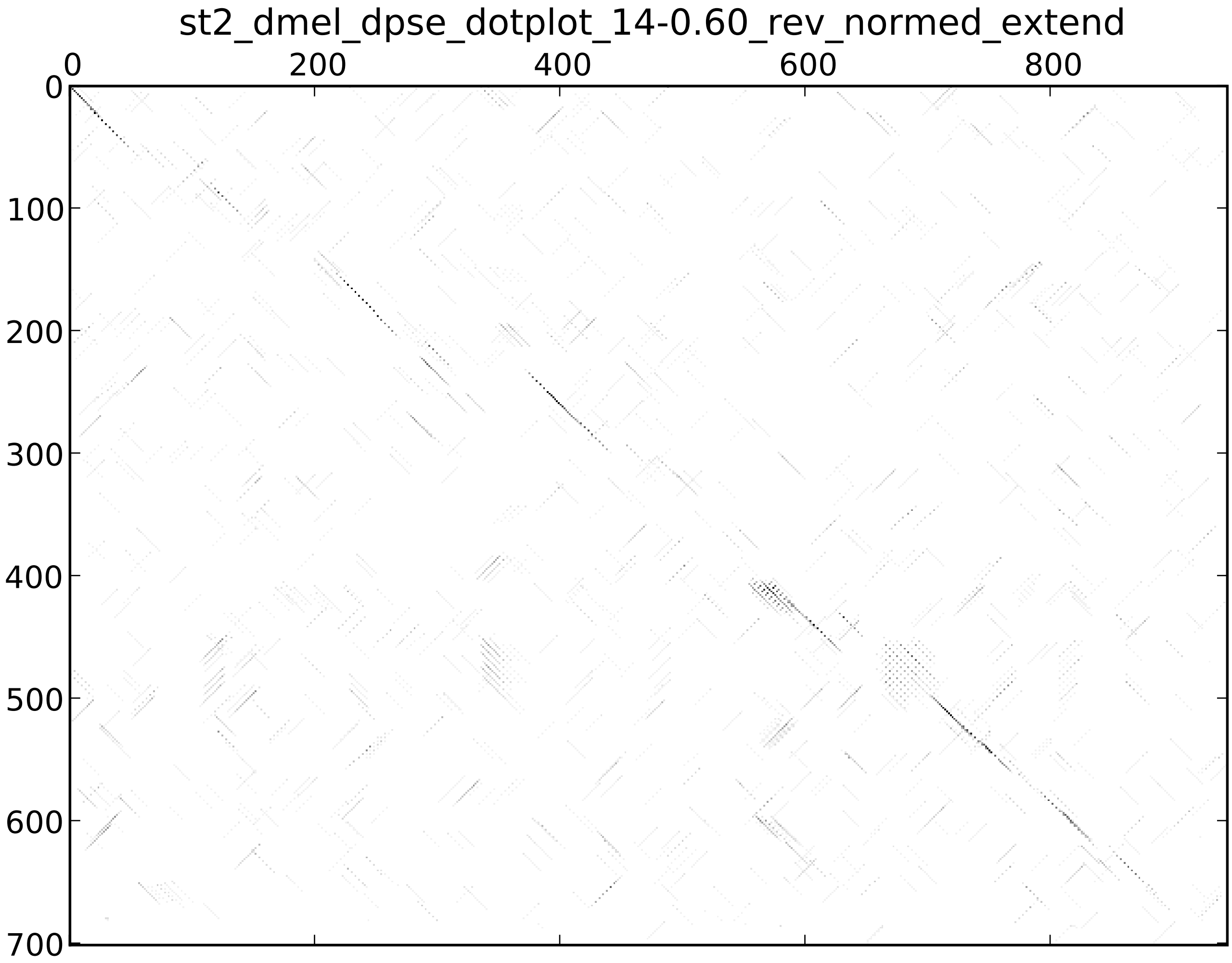
st2_dmel_dpse_dotplot_14-0.60_rev_normed_extend
0
200
400
600
800
0
100
200
300
400
500
600
700

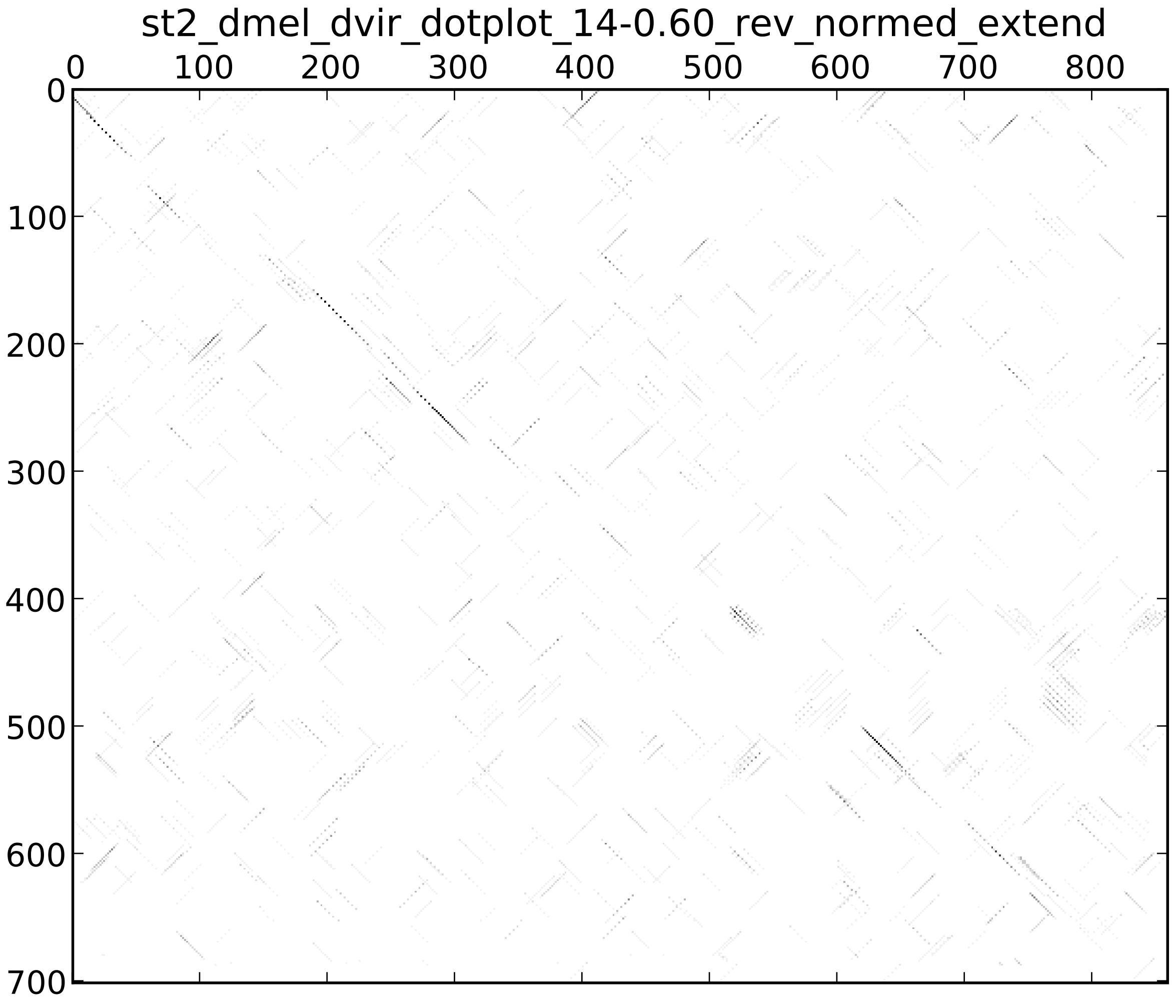
st2_dmel_dvir_dotplot_14-0.60_rev_normed_extend
0
100
200
300
400
500
600
700
800
0
100
200
300
400
500
600
700

st2_dmel_sepsis_cynipsea_dotplot_14-0.60_rev_normed_extend

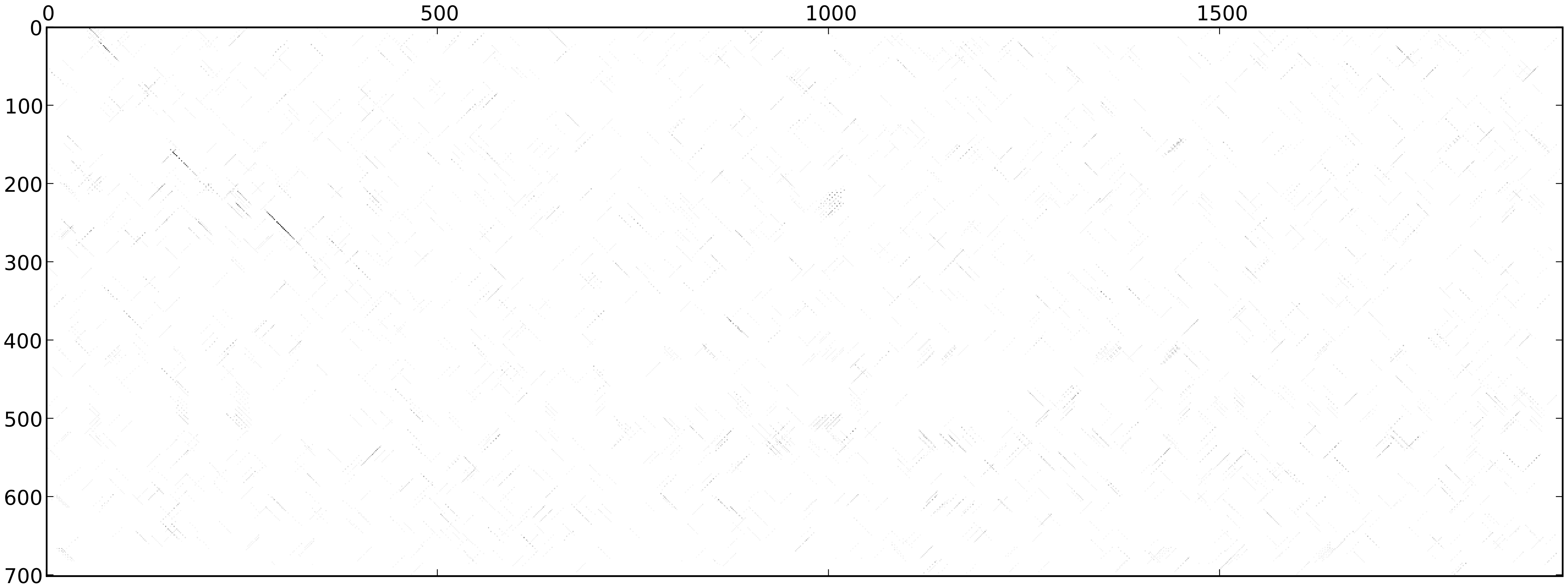

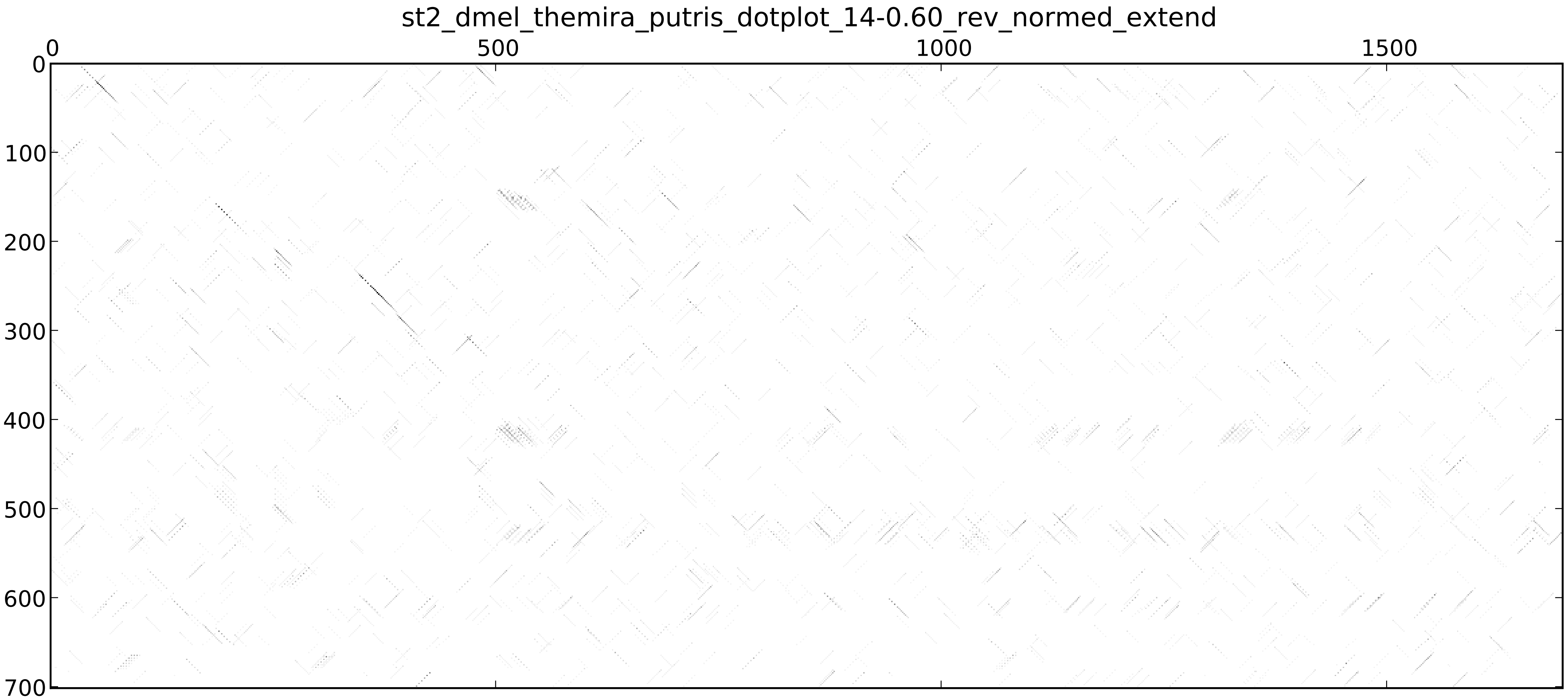
st2_dmel_themira_putris_dotplot_14-0.60_rev_normed_extend
0
500
1000
1500
0
100
200
300
400
500
600
700

st37_dmel_dpse_dotplot_14-0.60_rev_normed_extend

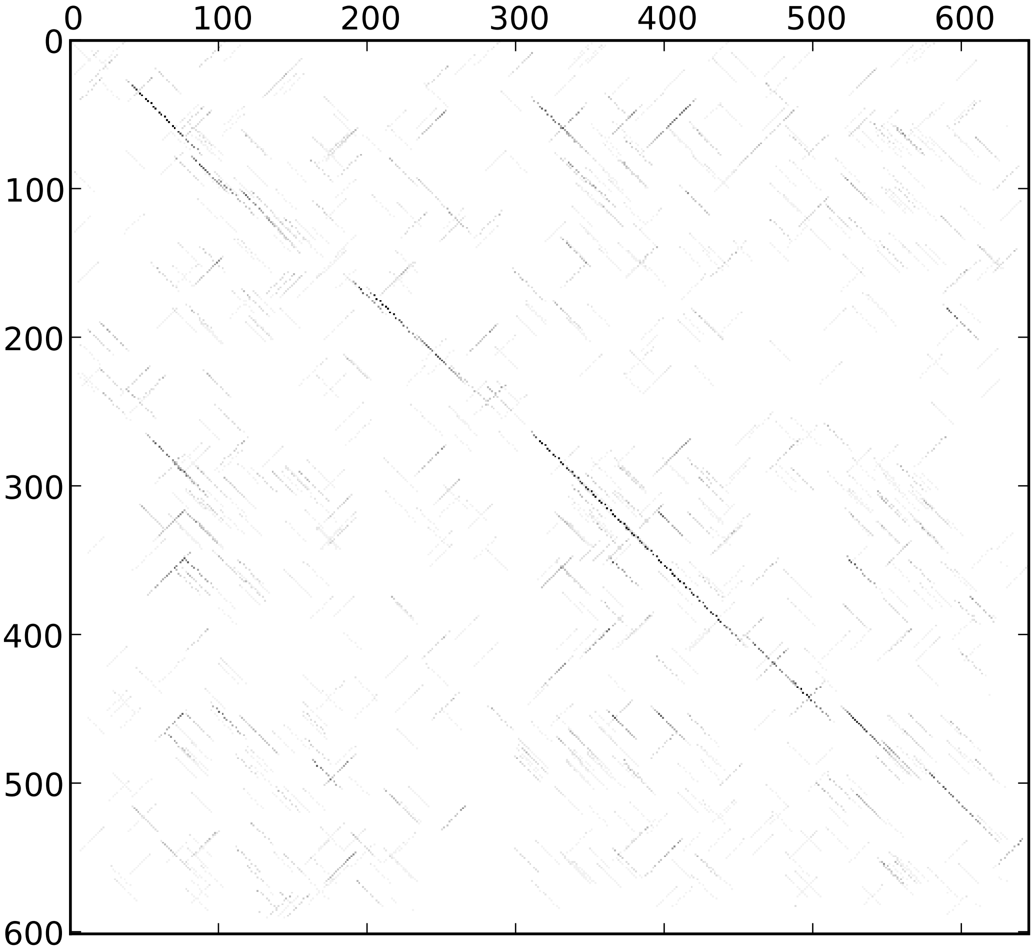

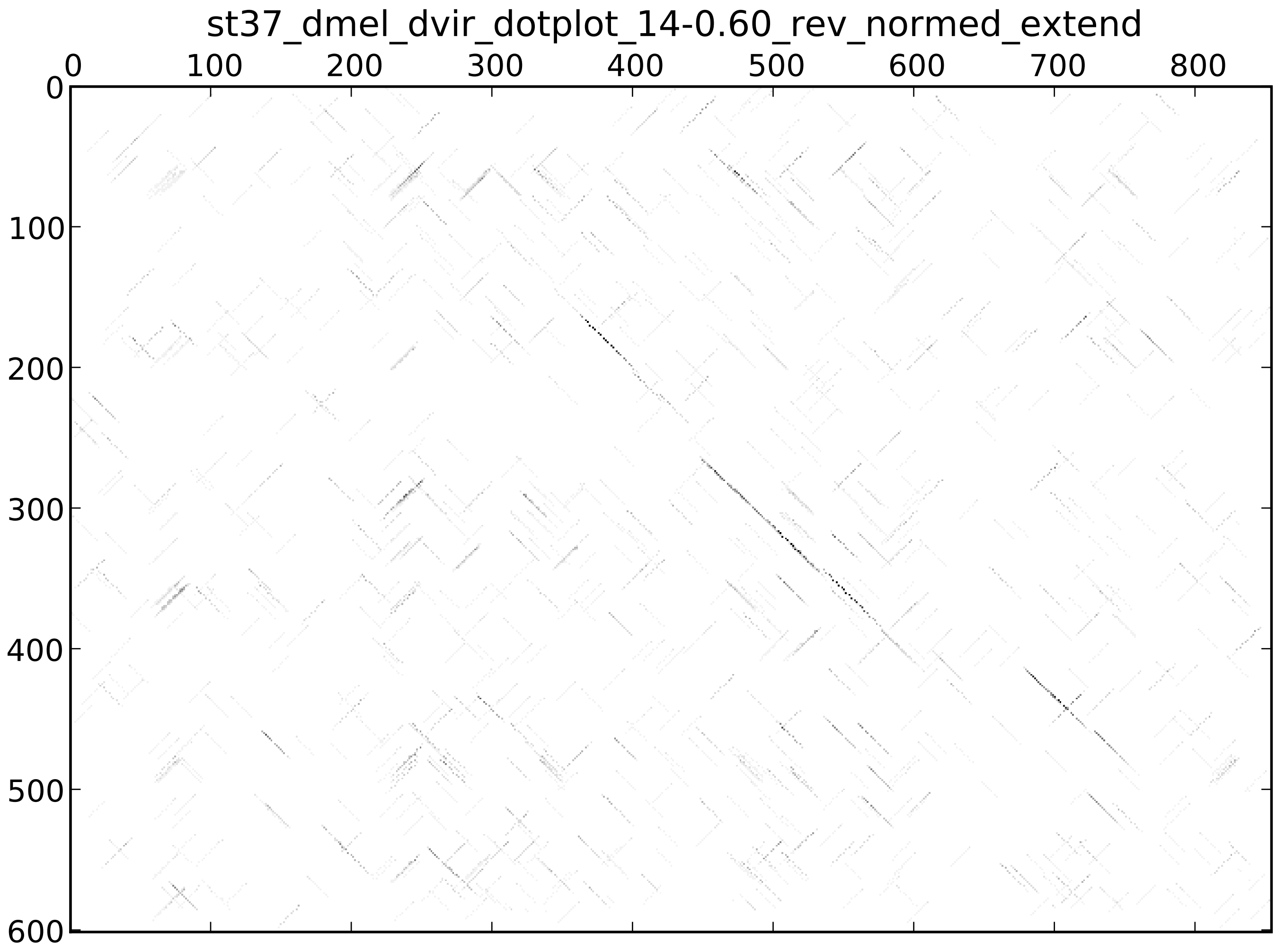
st37_dmel_dvir_dotplot_14-0.60_rev_normed_extend
0
100
200
300
400
500
600
700
800
0
100
200
300
400
500
600

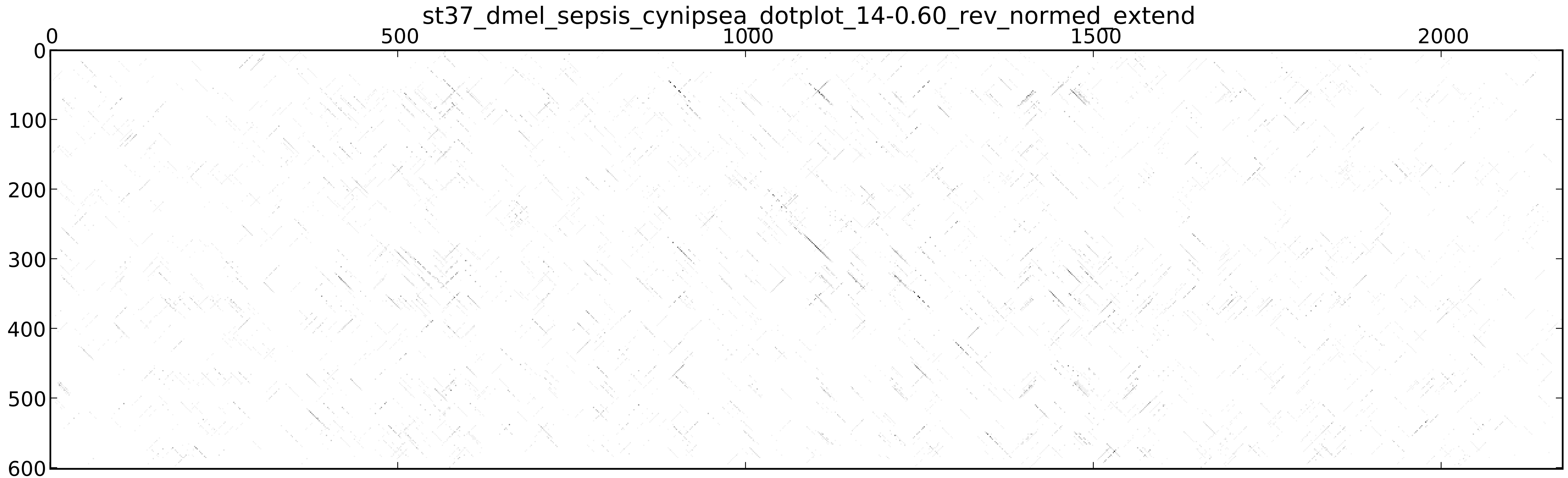
st37_dmel_sepsis_cynipsea_dotplot_14-0.60_rev_normed_extend
0
500
1000
1500
2000
0
100
200
300
400
500
600

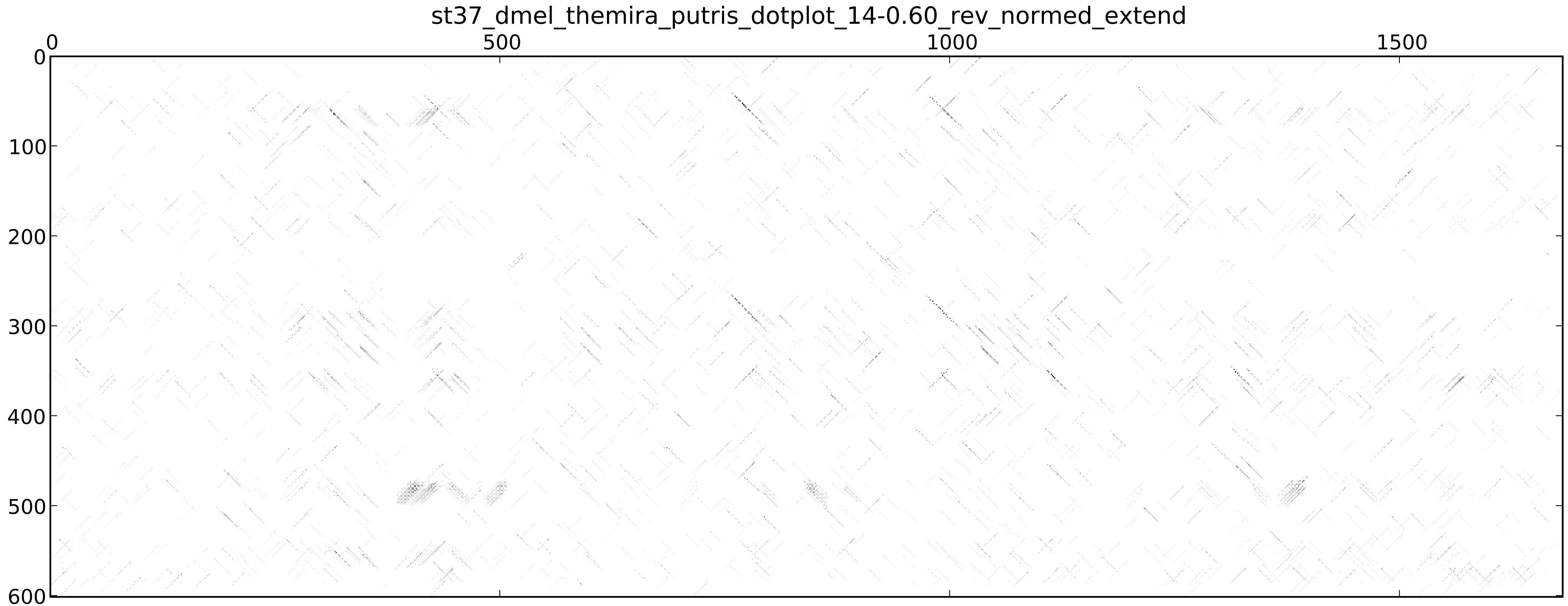
st37_dmel_themira_putris_dotplot_14-0.60_rev_normed_extend
0
500
1000
1500
0
100
200
300
400
500
600

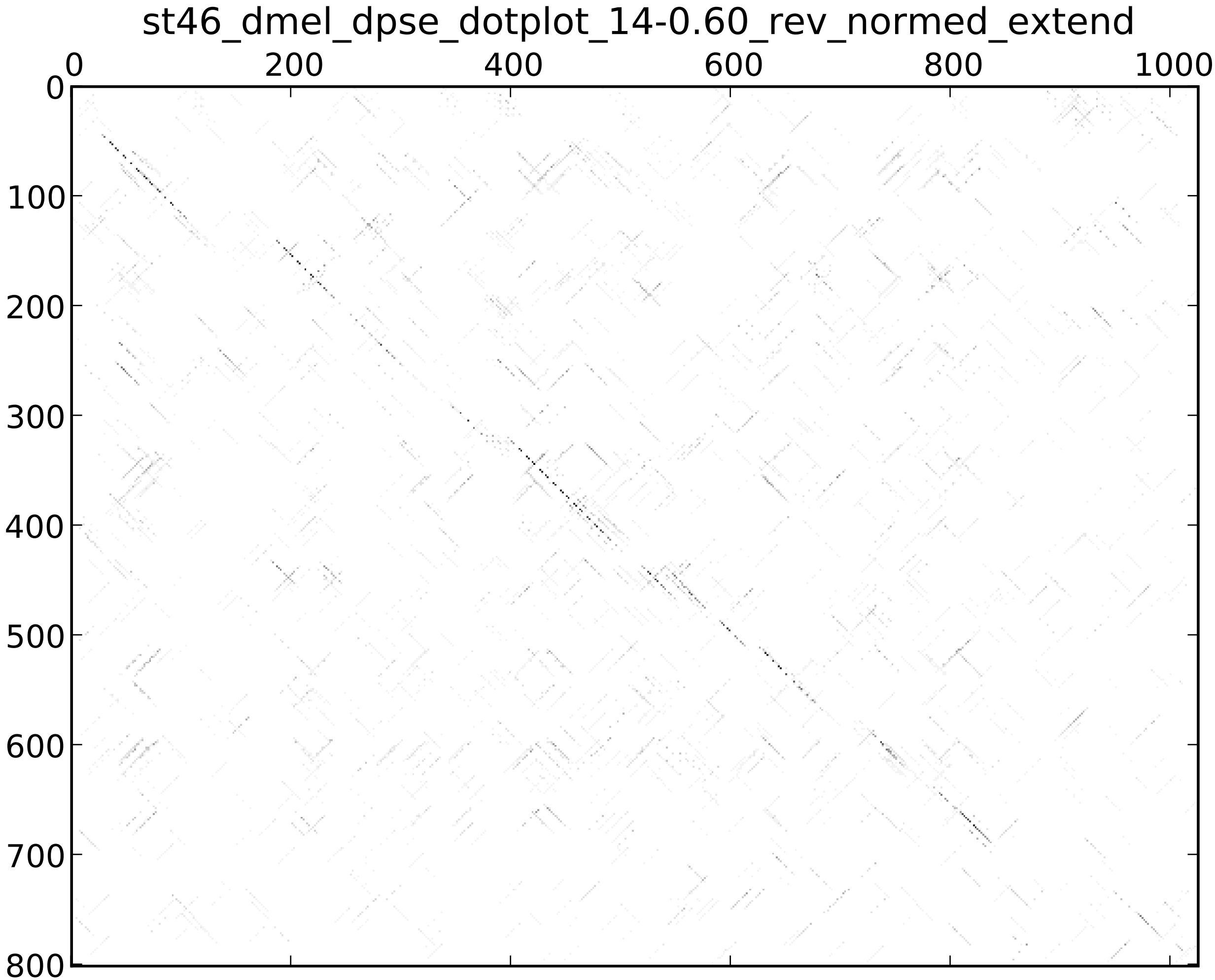
st46_dmel_dpse_dotplot_14-0.60_rev_normed_extend
0
200
400
600
800
1000
0
100
200
300
400
500
600
700
800

st46_dmel_dvir_dotplot_14-0.60_rev_normed_extend

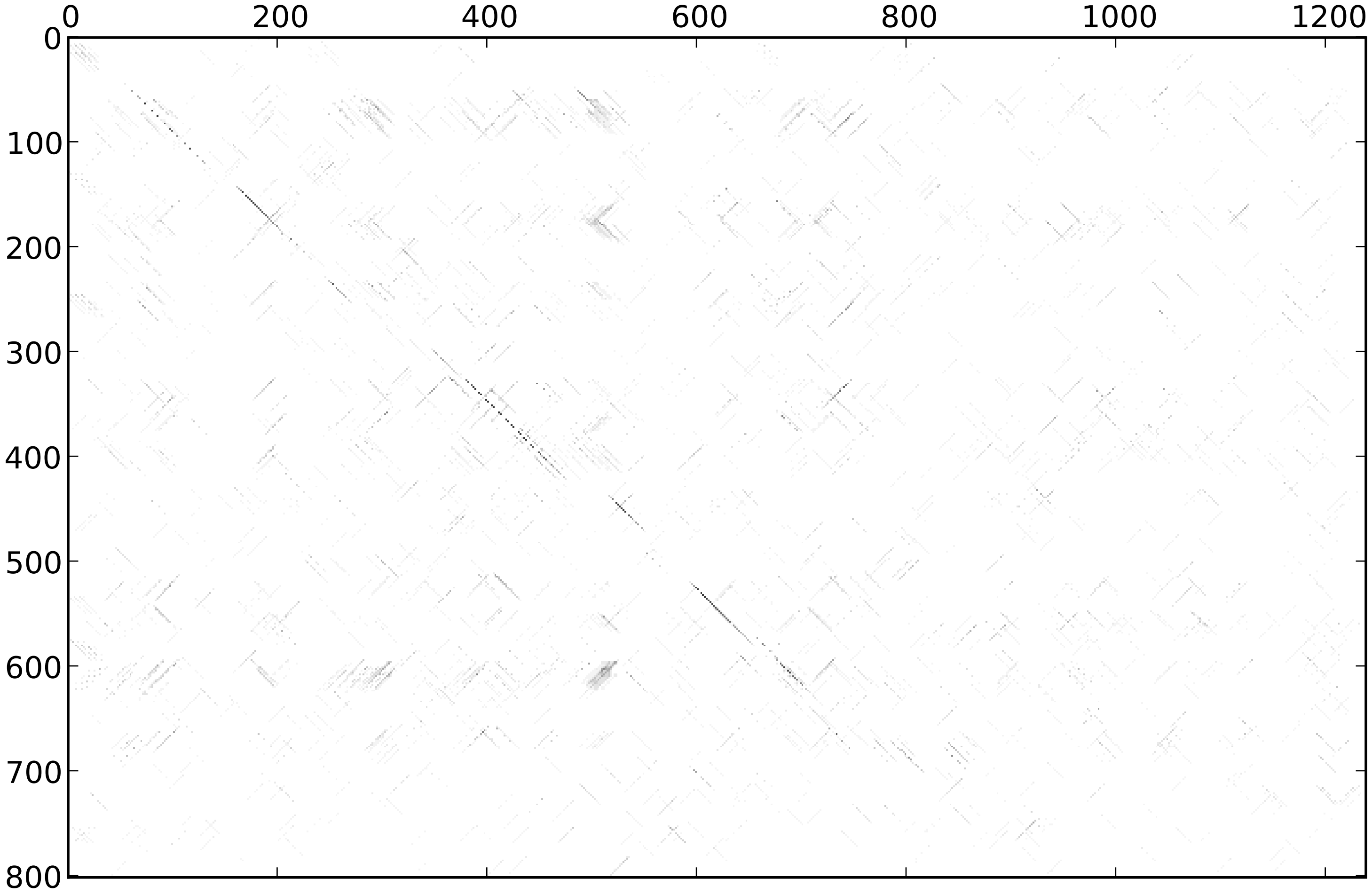

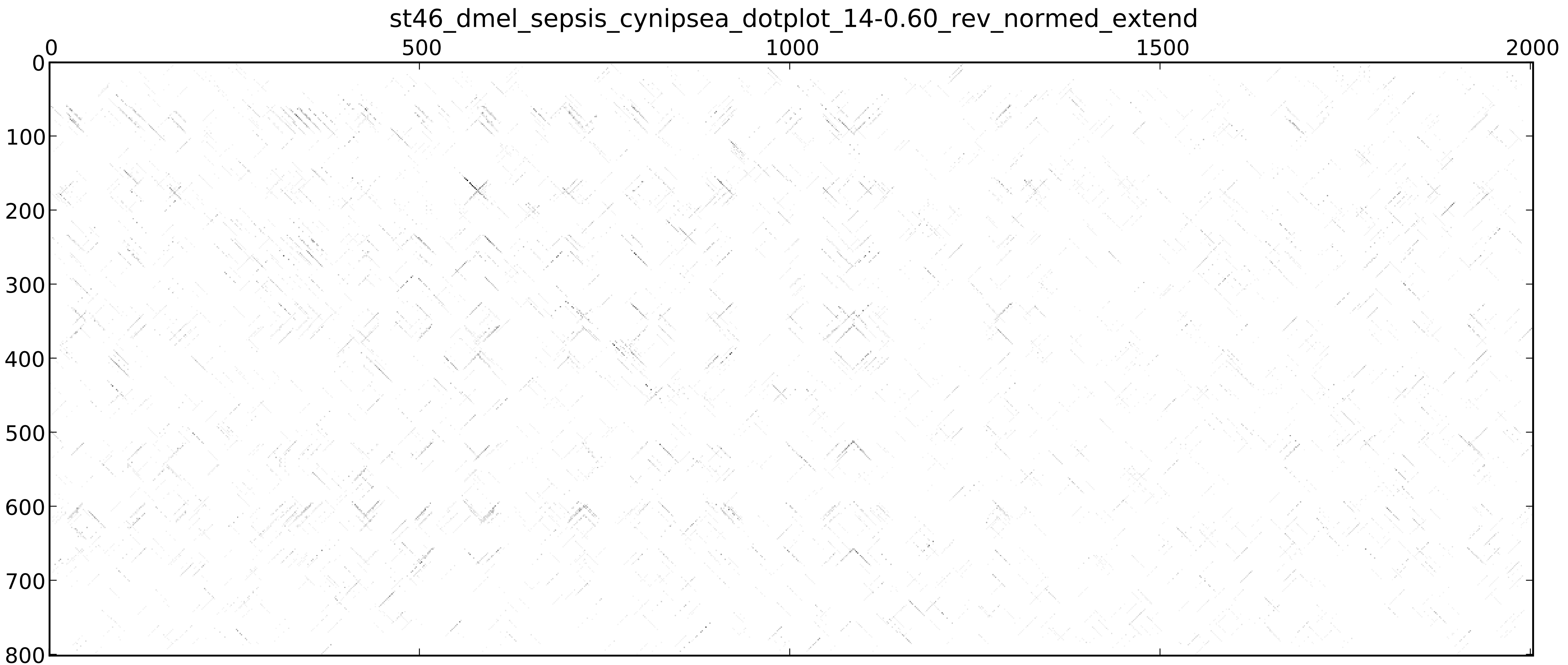
st46_dmel_sepsis_cynipsea_dotplot_14-0.60_rev_normed_extend
0
500
1000
1500
2000
0
100
200
300
400
500
600
700
800

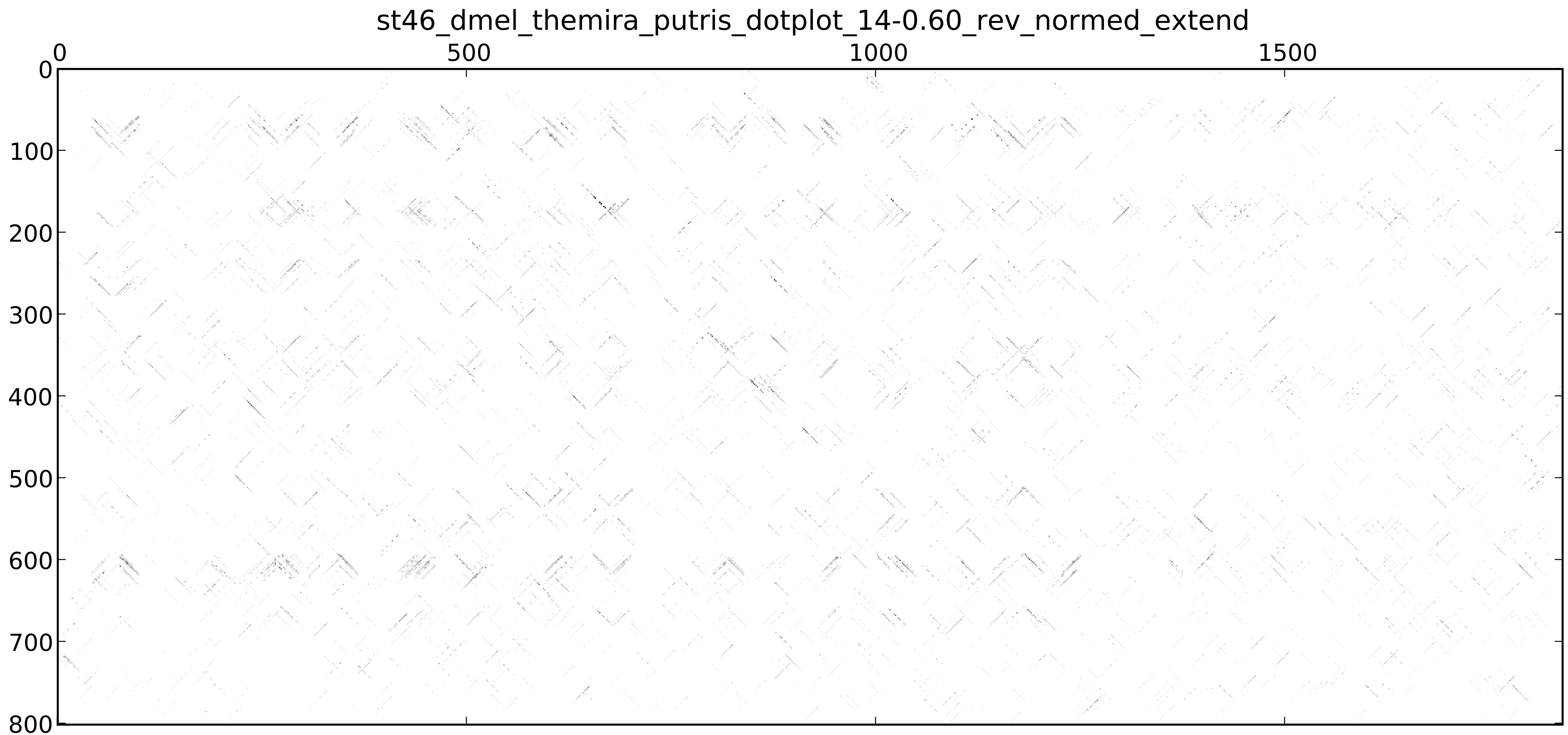
st46_dmel_themira_putris_dotplot_14-0.60_rev_normed_extend
0
500
1000
1500
0
100
200
300
400
500
600
700
800

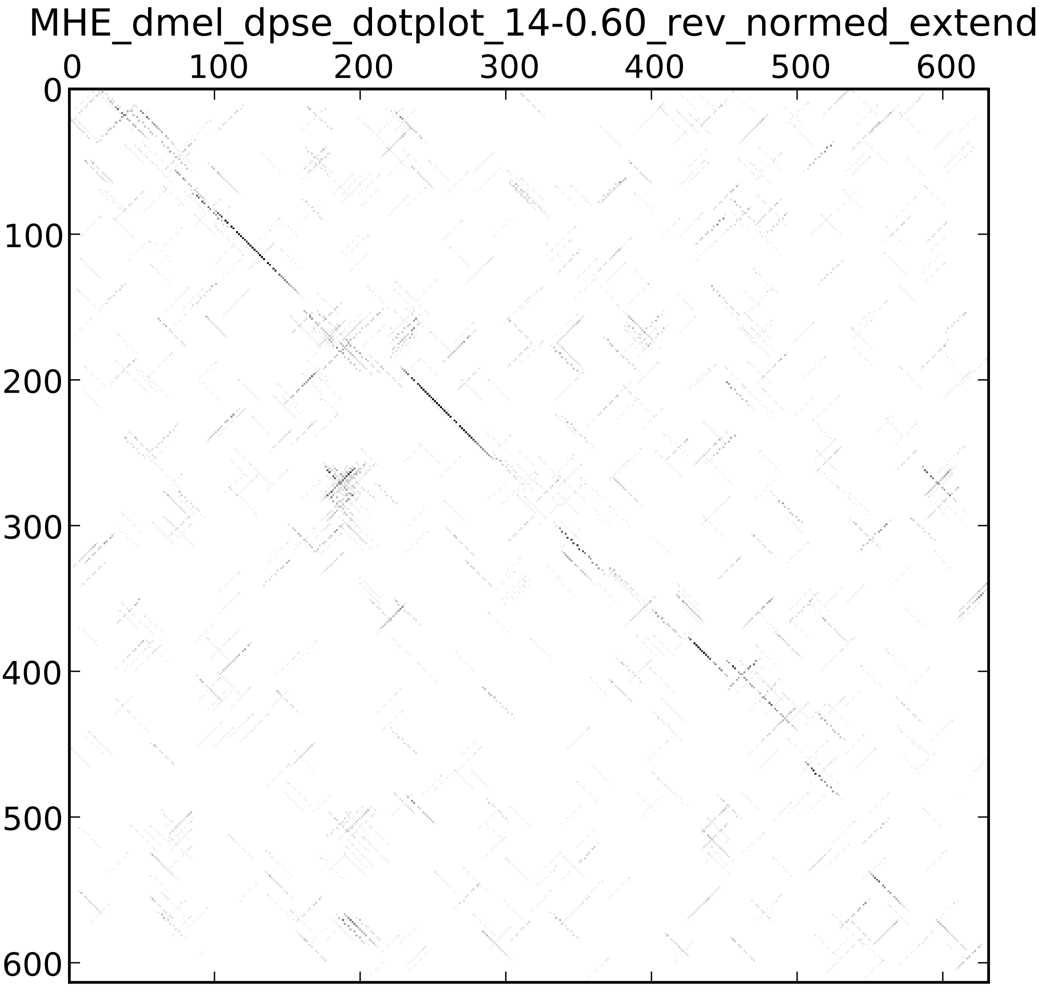
MHE_dmel_dpse_dotplot_14-0.60_rev_normed_extend
0
100
200
300
400
500
600
0
100
200
300
400
500
600

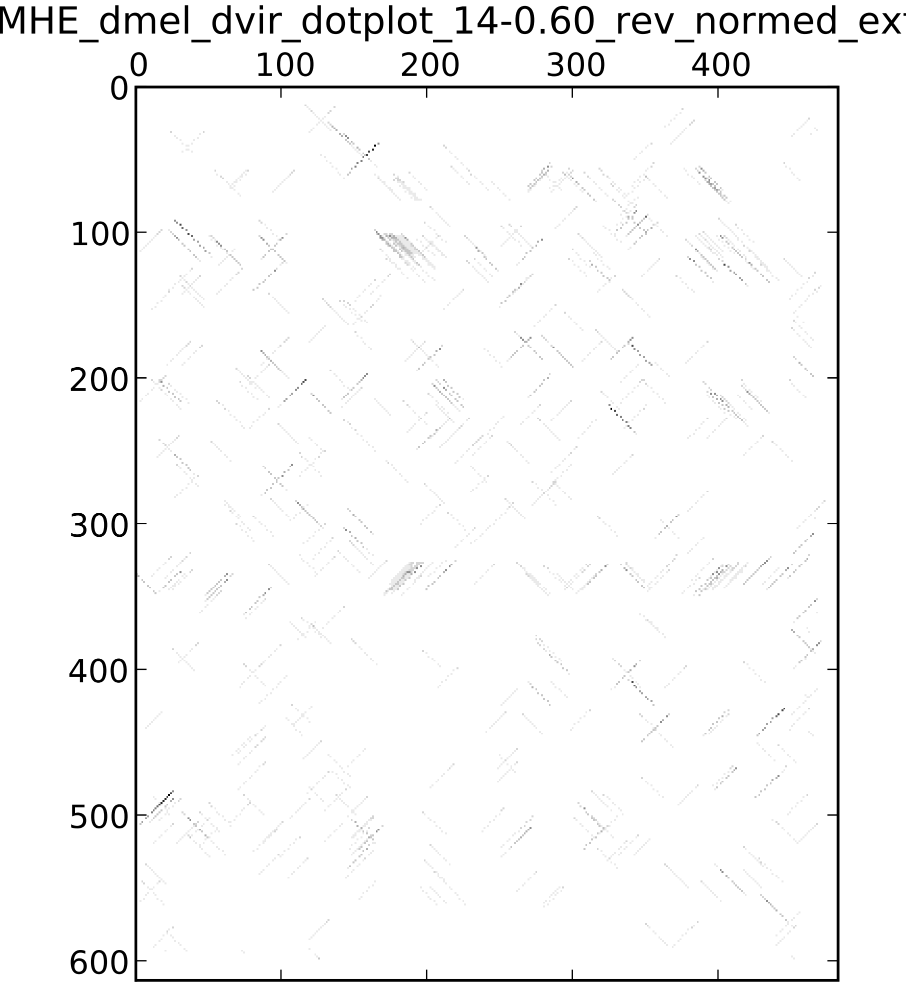

MHE_dmel_dvir_dotplot_14-0.60_rev_normed_ext
0
100
200
300
400
0
100
200
300
400
500
600

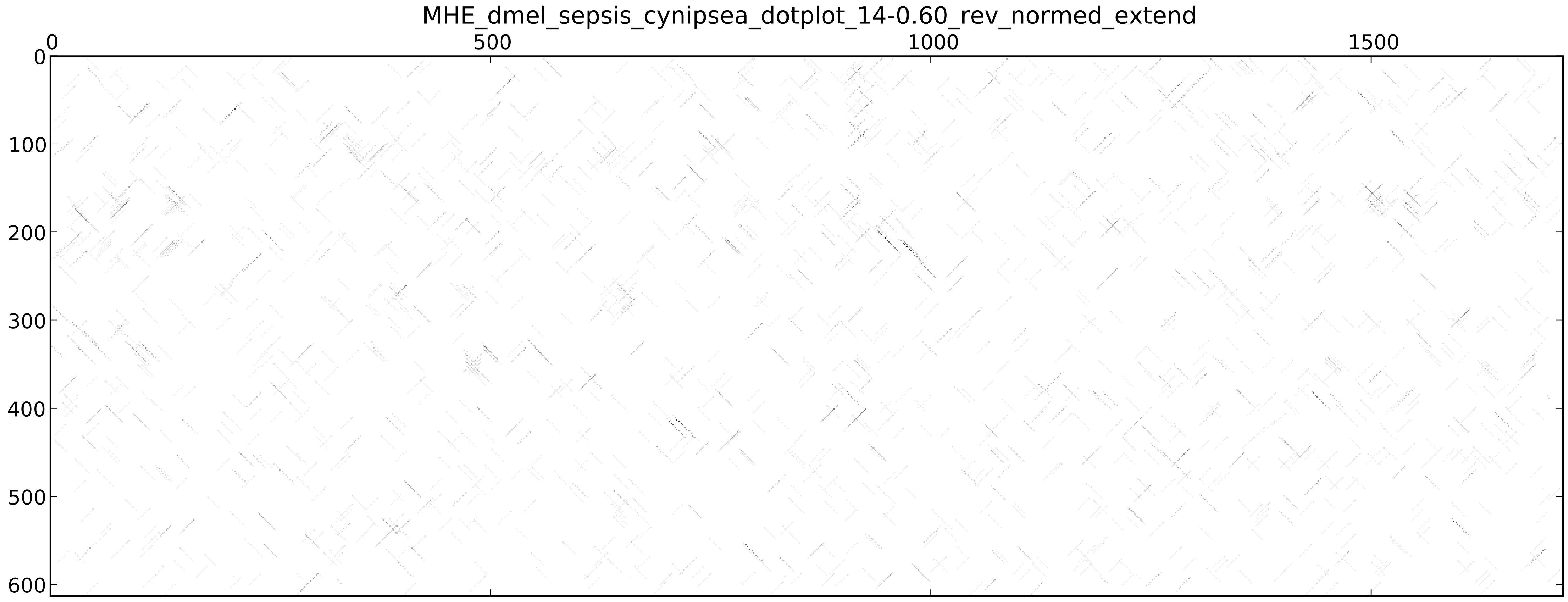

MHE_dmel_sepsis_cynipsea_dotplot_14-0.60_rev_normed_extend
0
500
1000
1500
0
100
200
300
400
500
600

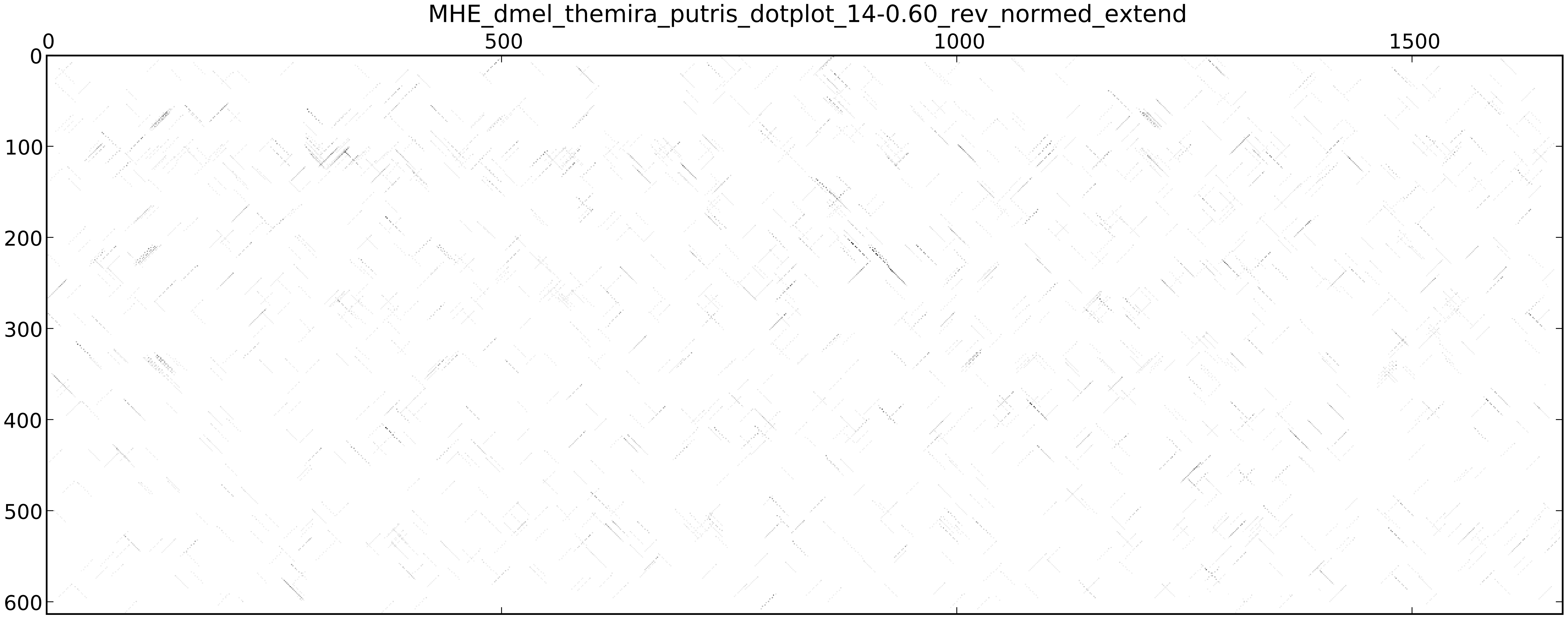
MHE_dmel_themira_putris_dotplot_14-0.60_rev_normed_extend
0
500
1000
1500
0
100
200
300
400
500
600
