# Supplementary material for: A Careful Look at Binding Site Reorganization in the even-skipped Enhancers of Drosophila and Sepsids
Source: PLoS Genet. 2008 Nov 28;4(11):e1000268. doi: 10.1371/journal.pgen.1000268 (PMC2582681; doi:10.1371/journal.pgen.1000268)

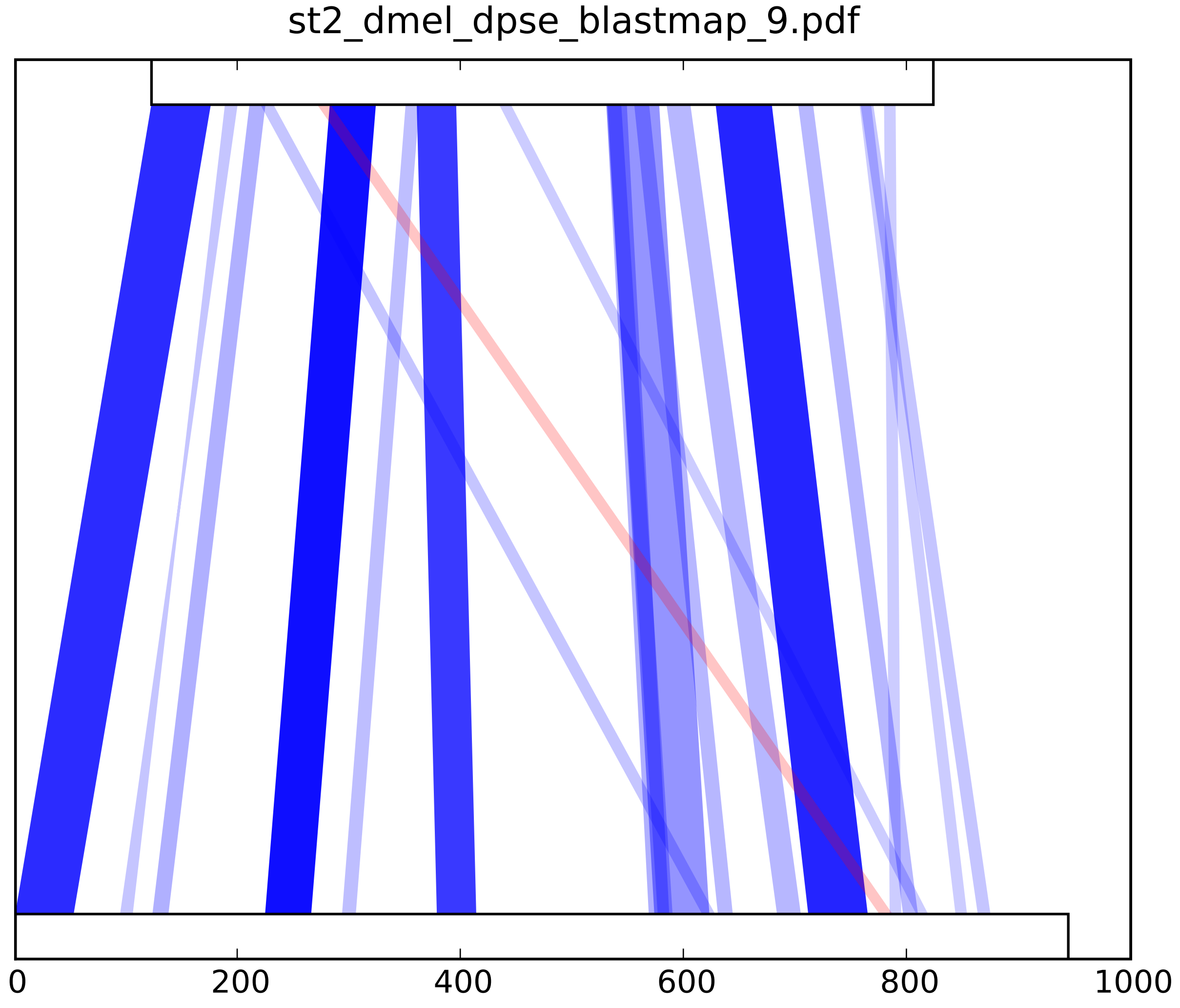
st2_dmel_dpse_blastmap_9.pdf
0
200
400
600
800
1000

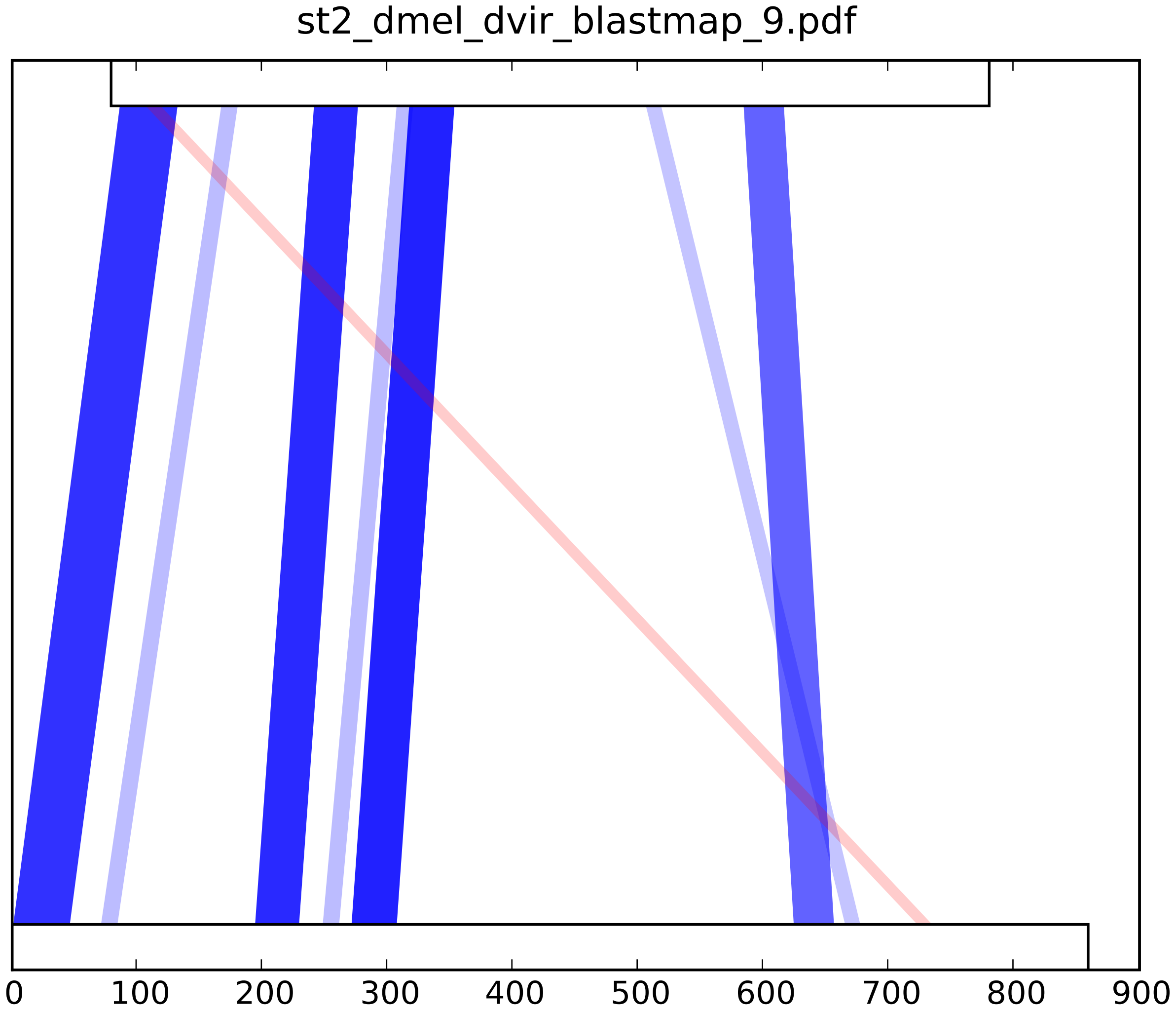
st2_dmel_dvir_blastmap_9.pdf
0
100
200
300
400
500
600
700
800
900

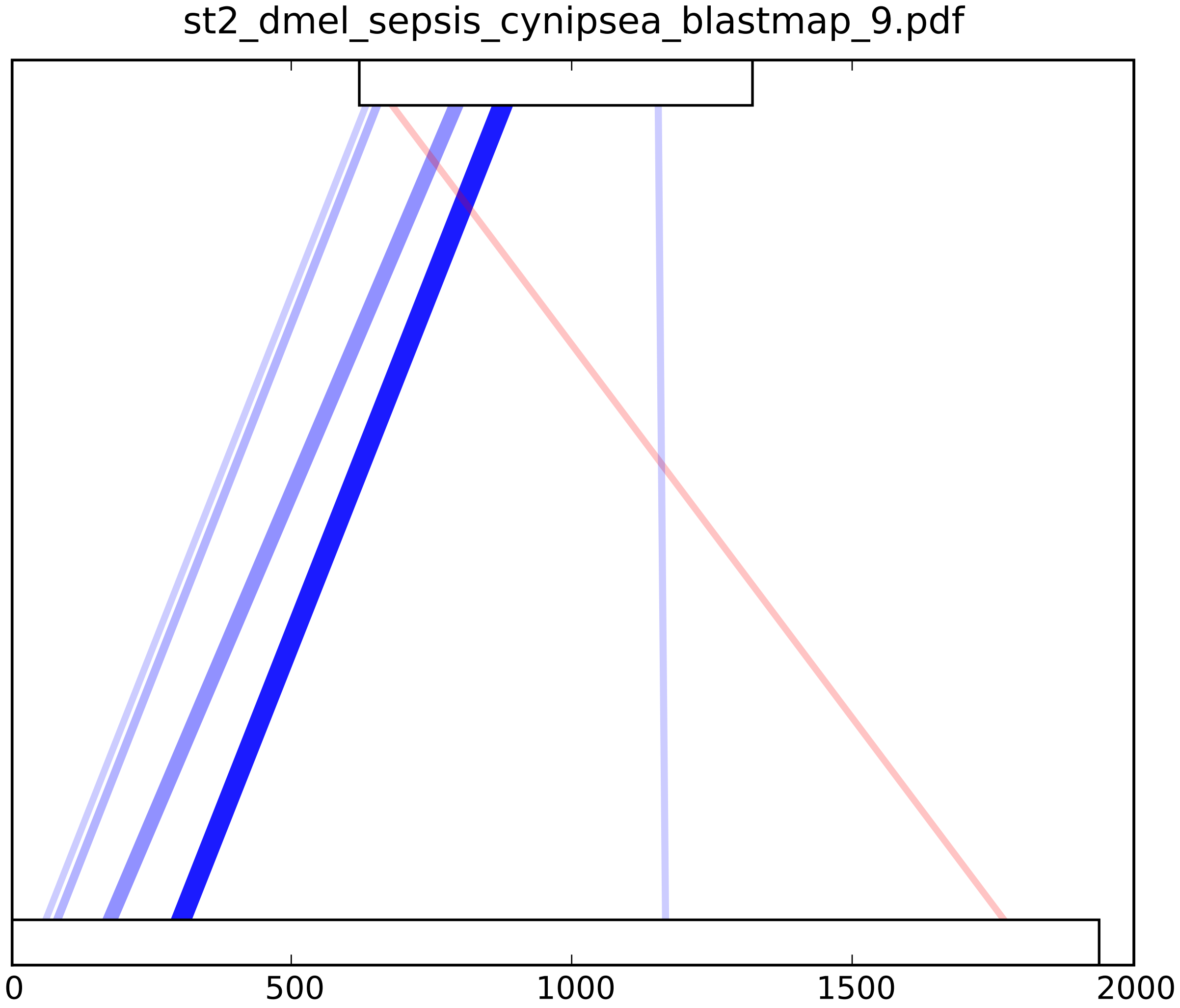

st2_dmel_sepsis_cynipsea_blastmap_9.pdf
0
500
1000
1500
2000

st2_dmel_themira_putris_blastmap_9.pdf

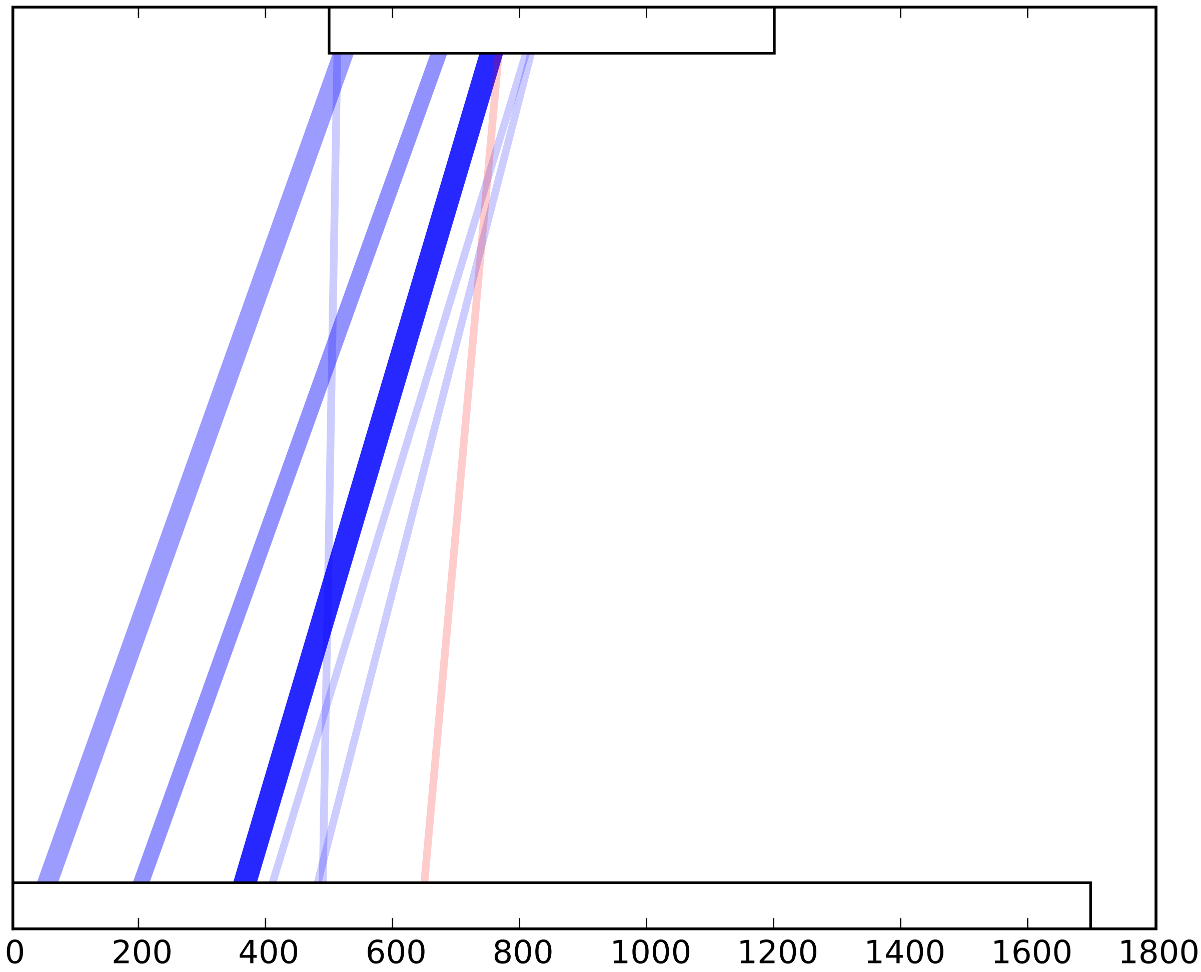

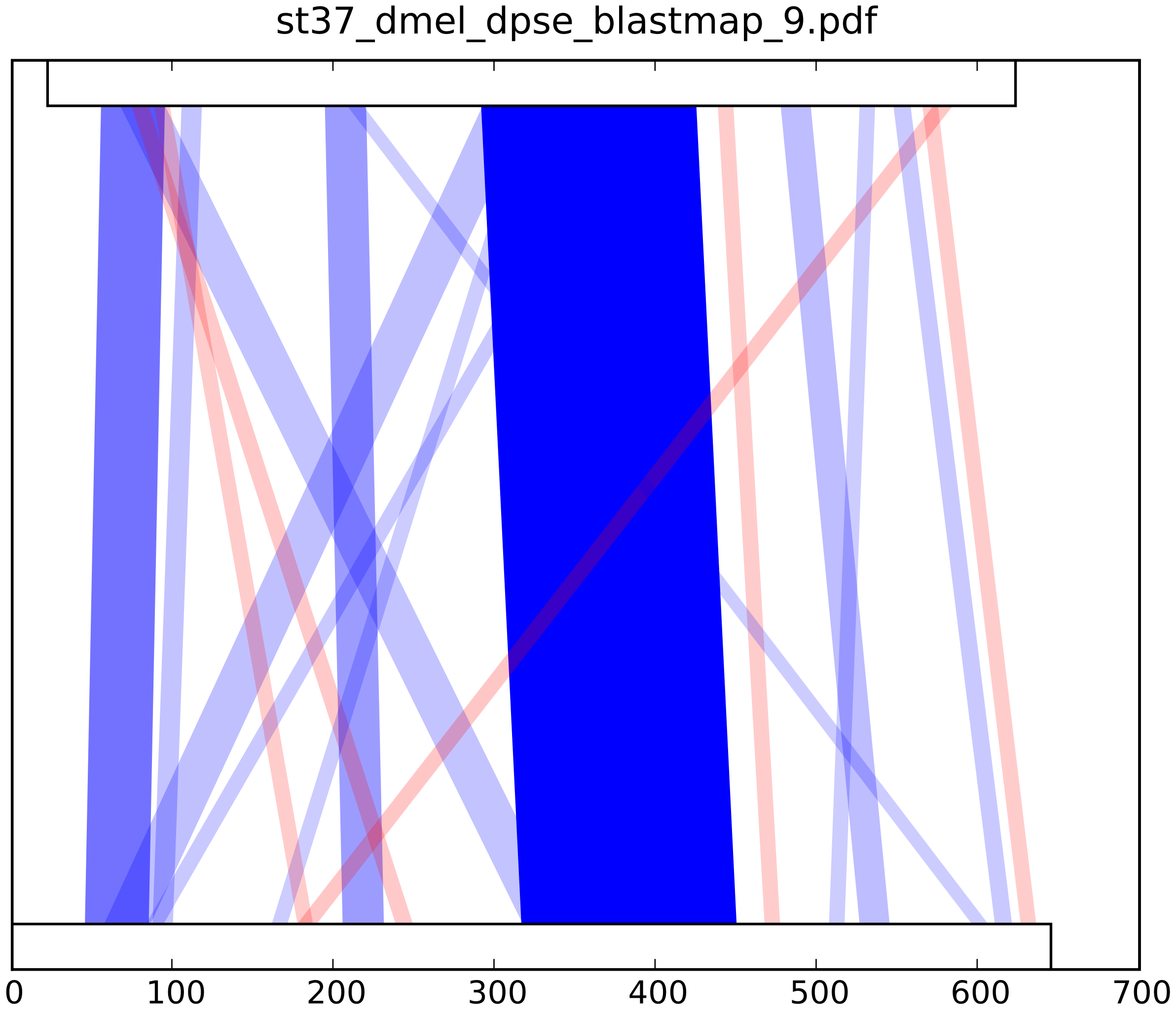

st37_dmel_dpse_blastmap_9.pdf
0
100
200
300
400
500
600
700

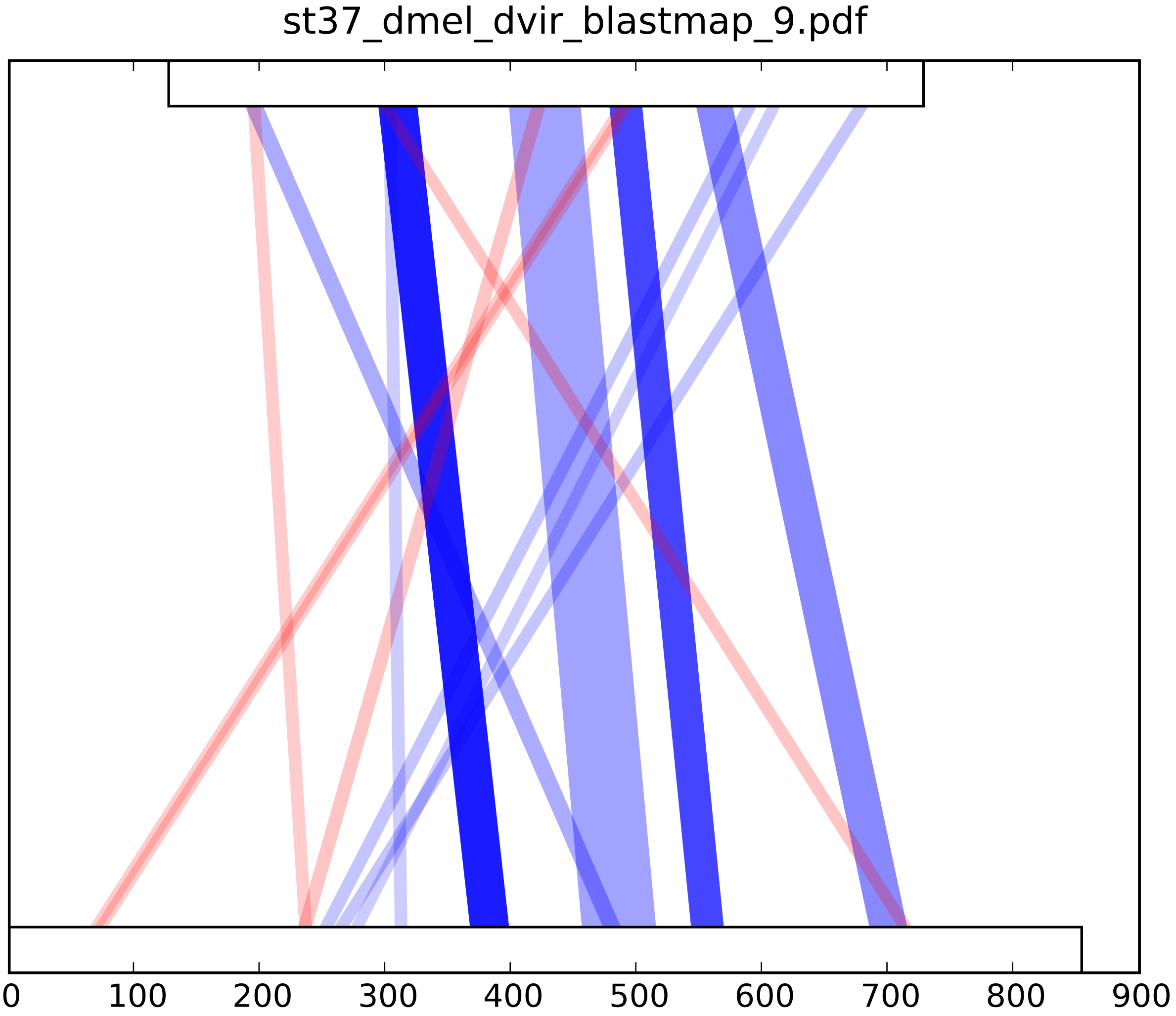
st37_dmel_dvir_blastmap_9.pdf
0
100
200
300
400
500
600
700
800
900

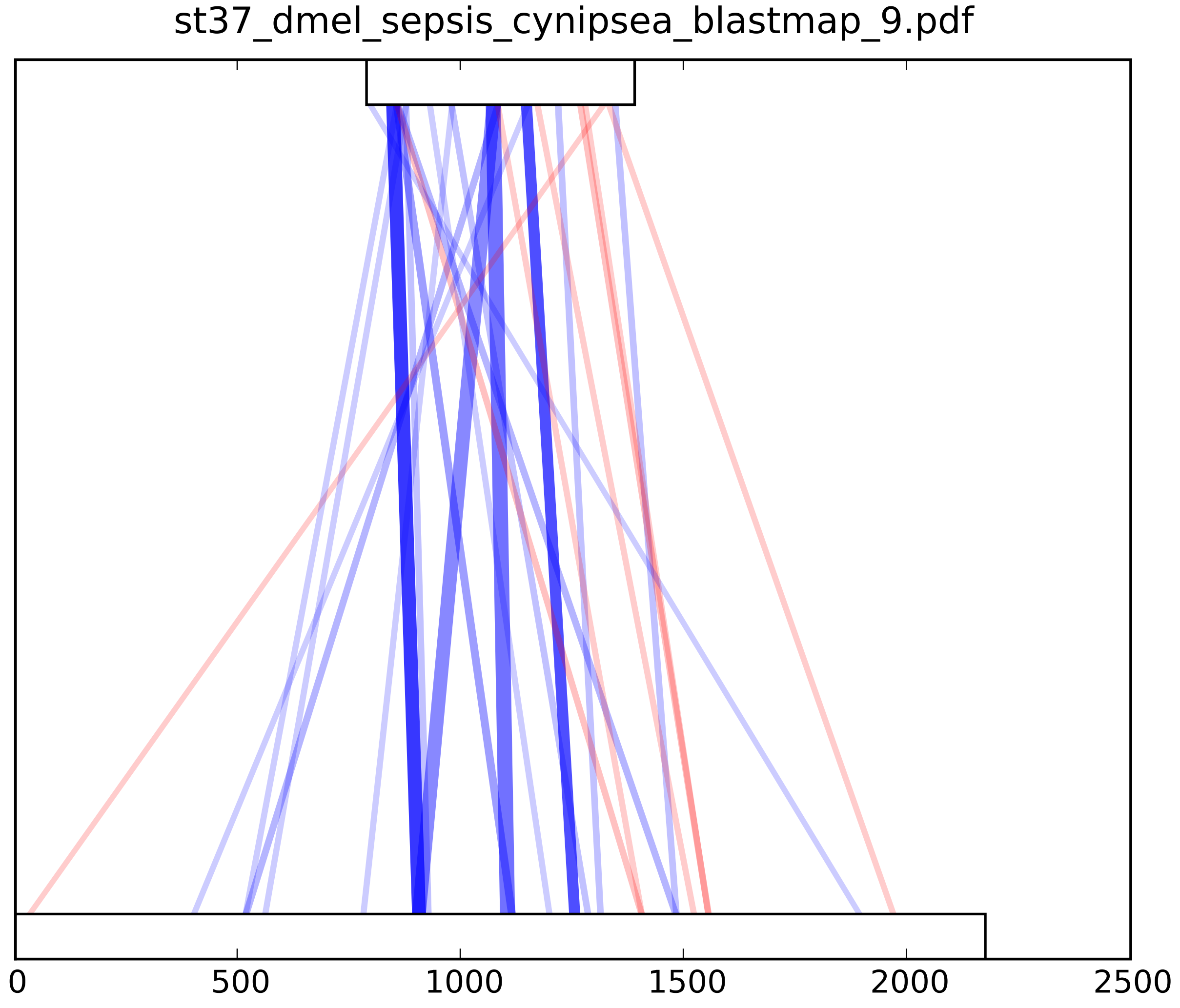
st37_dmel_sepsis_cynipsea_blastmap_9.pdf
0
500
1000
1500
2000
2500

st37_dmel_themira_putris_blastmap_9.pdf

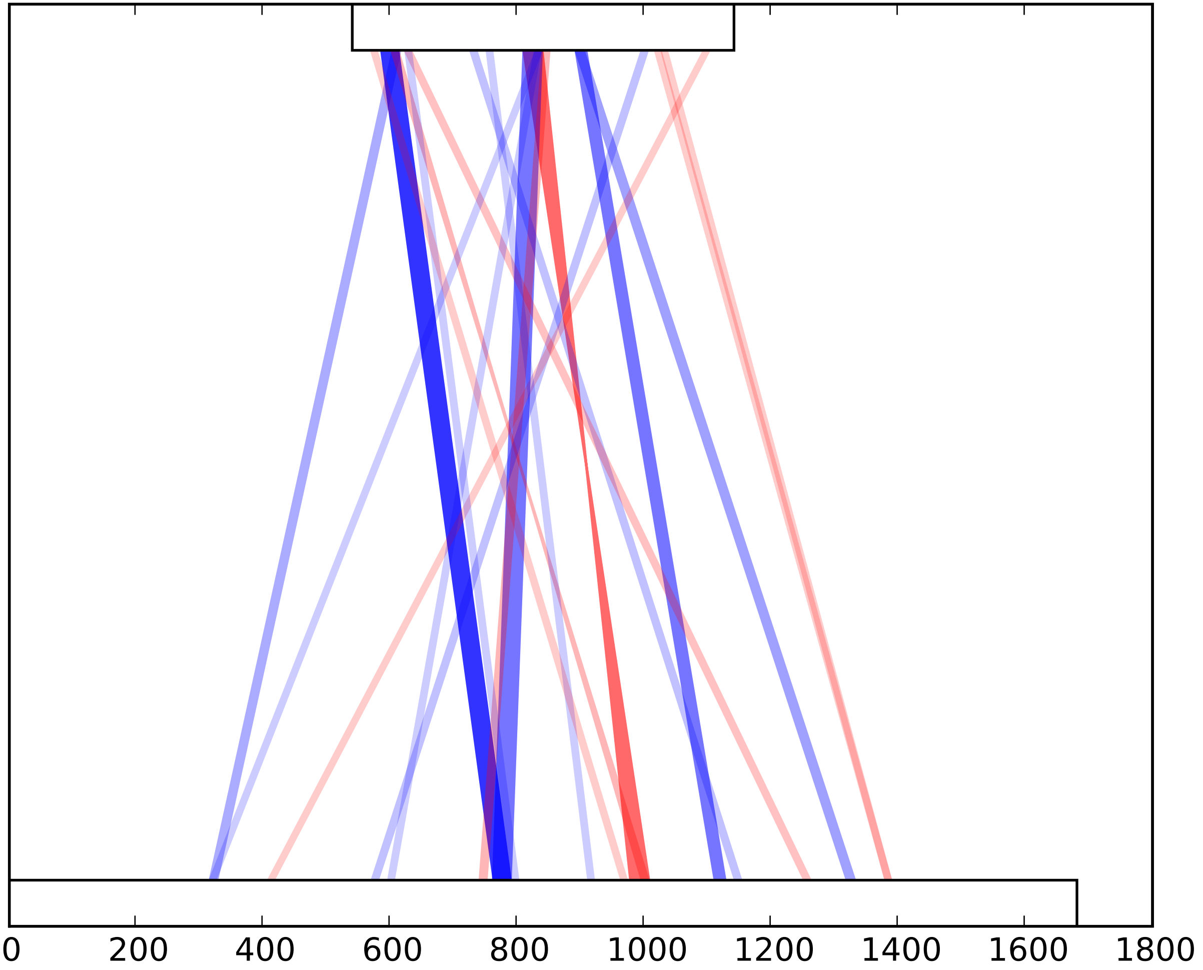

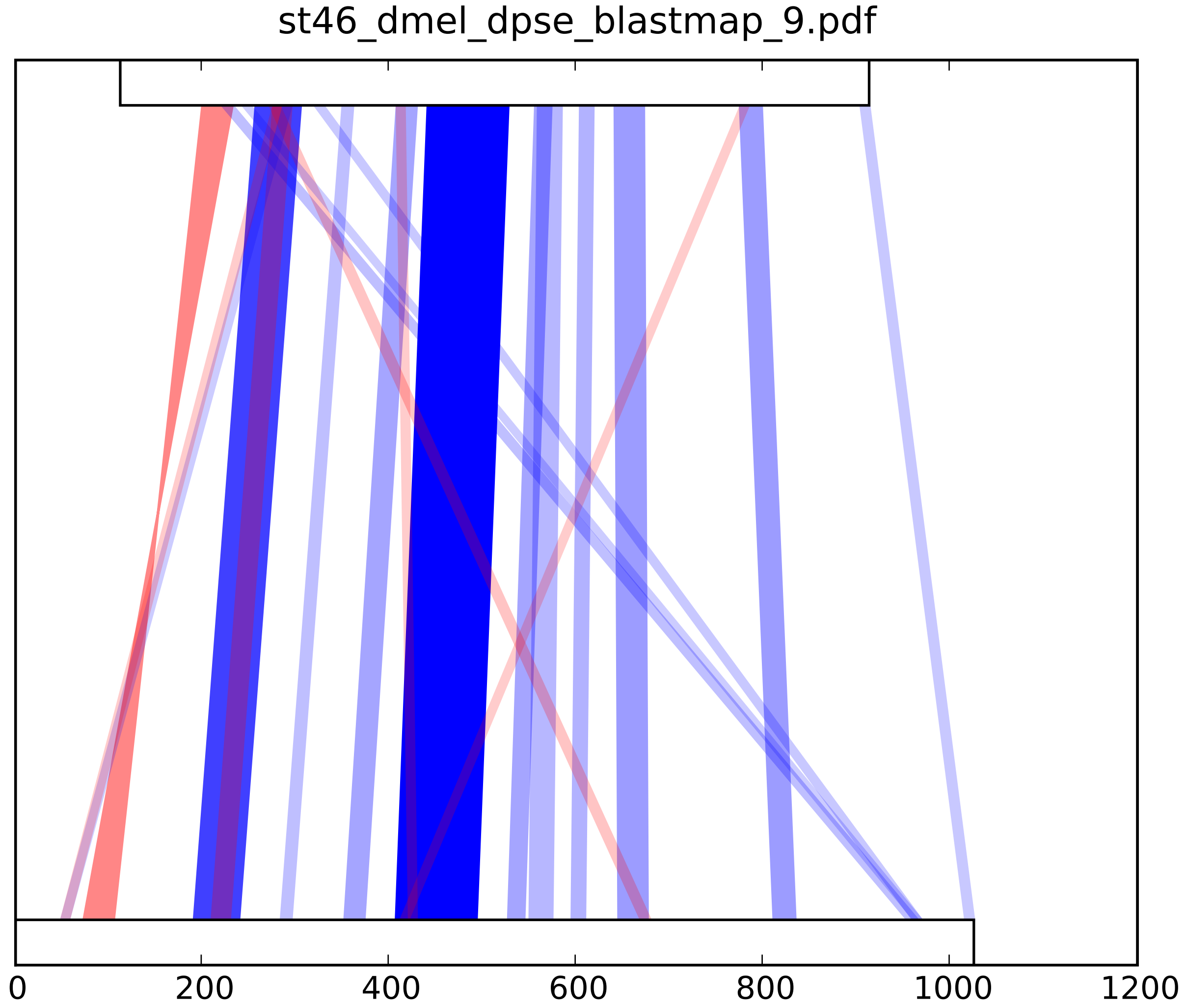
st46_dmel_dpse_blastmap_9.pdf
0
200
400
600
800
1000
1200

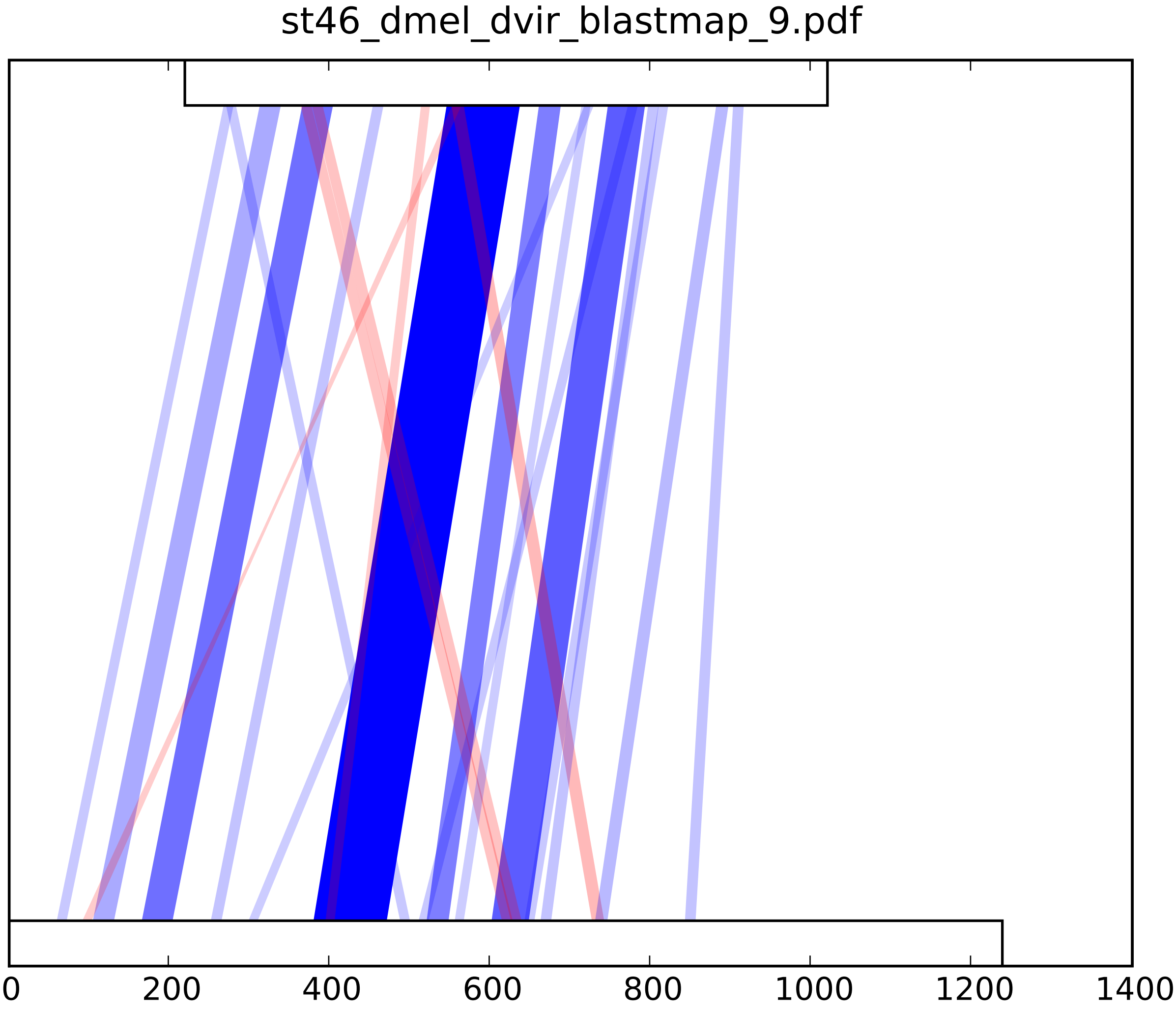
st46_dmel_dvir_blastmap_9.pdf
0
200
400
600
800
1000
1200
1400

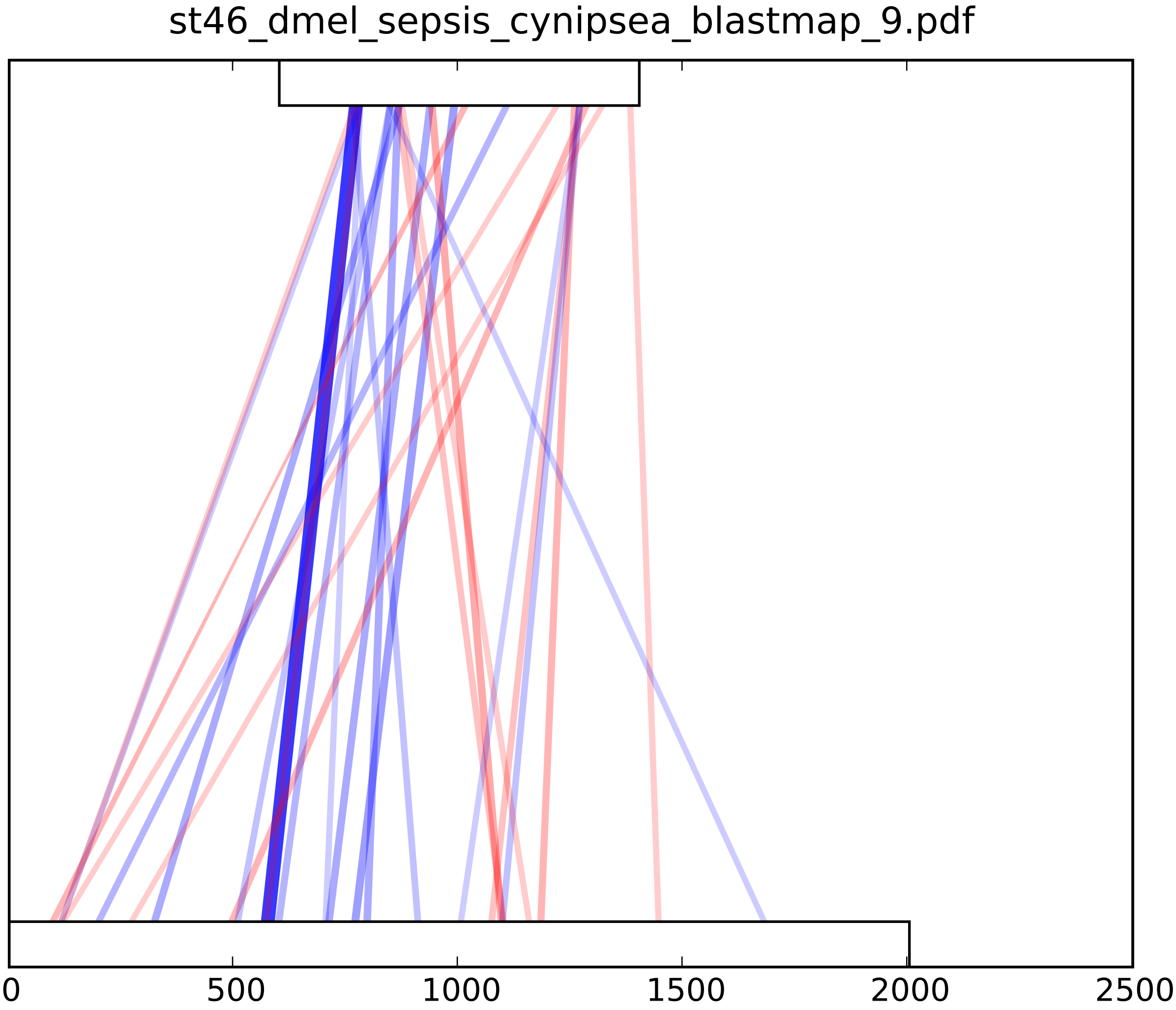
st46_dmel_sepsis_cynipsea_blastmap_9.pdf
0
500
1000
1500
2000
2500

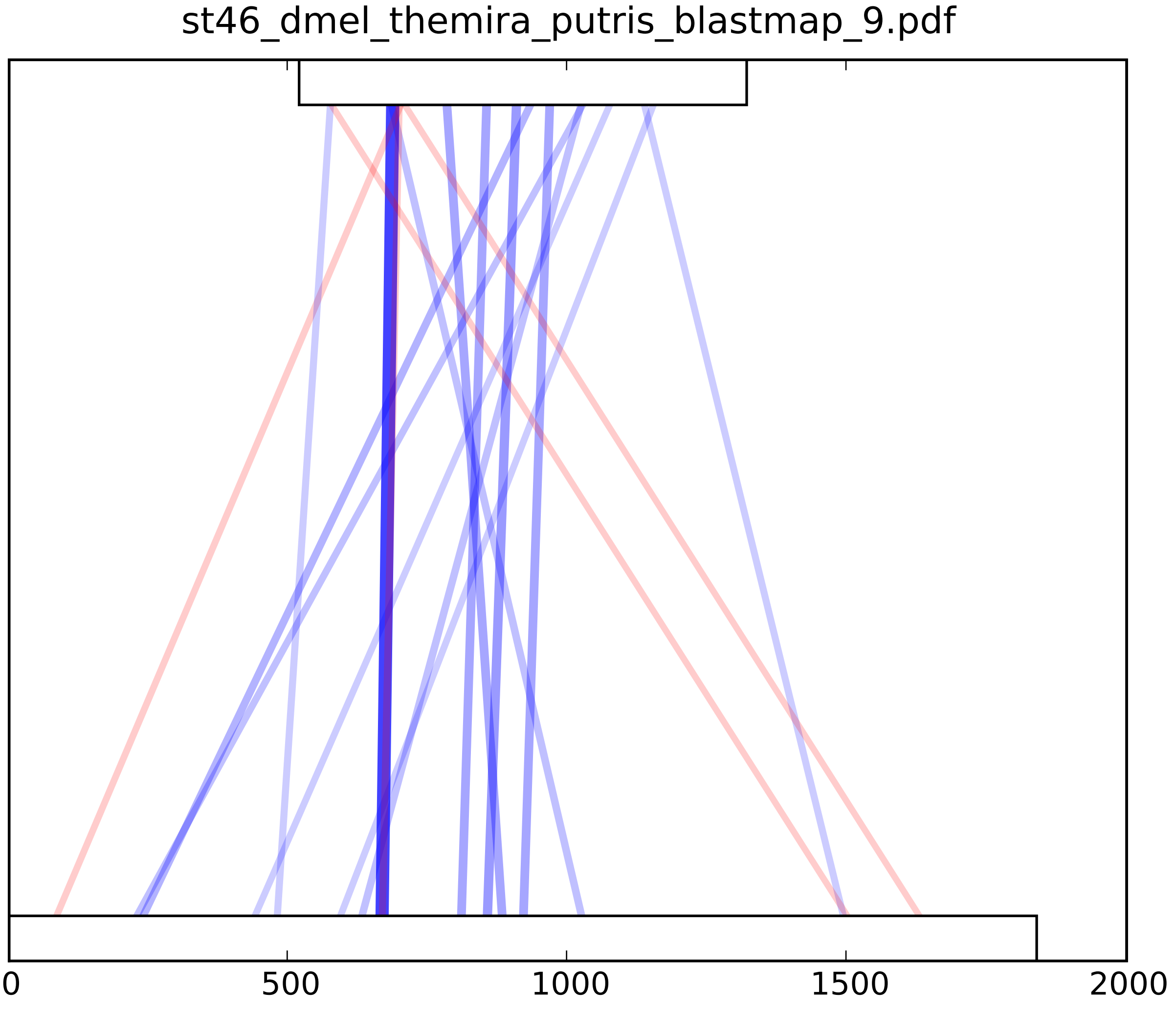
st46_dmel_themira_putris_blastmap_9.pdf
0
500
1000
1500
2000

MHE_dmel_dpse_blastmap_9.pdf

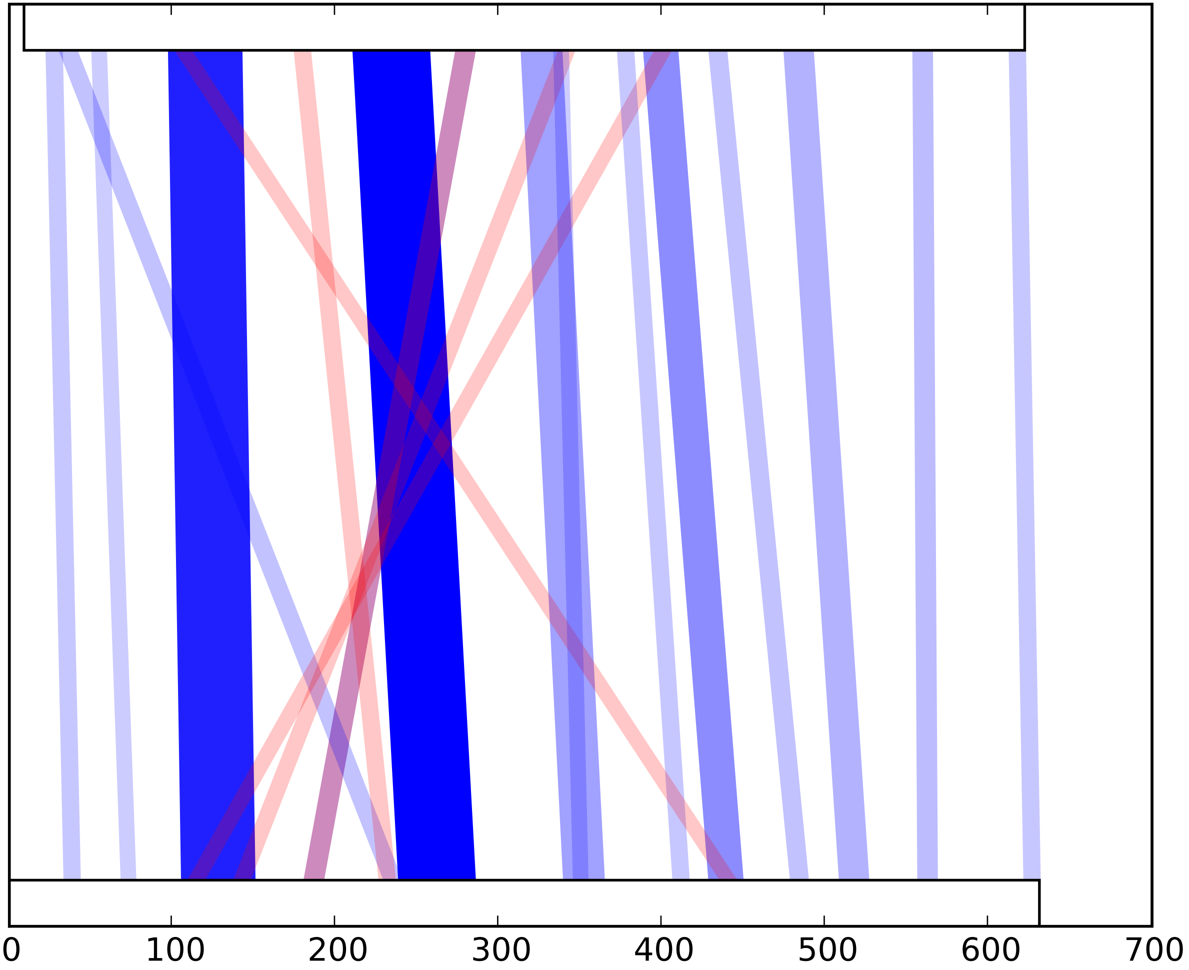

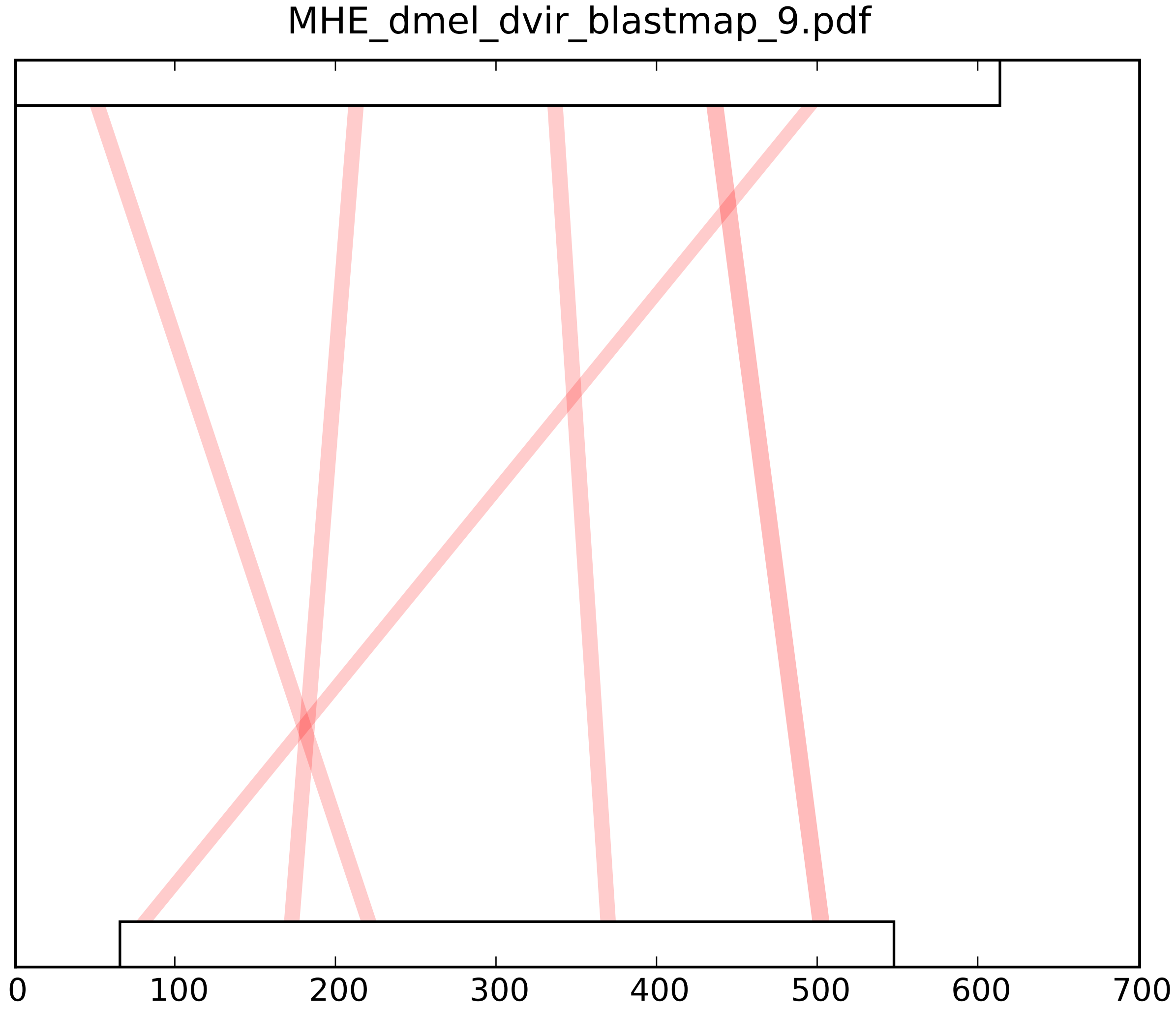
MHE_dmel_dvir_blastmap_9.pdf
0
100
200
300
400
500
600
700

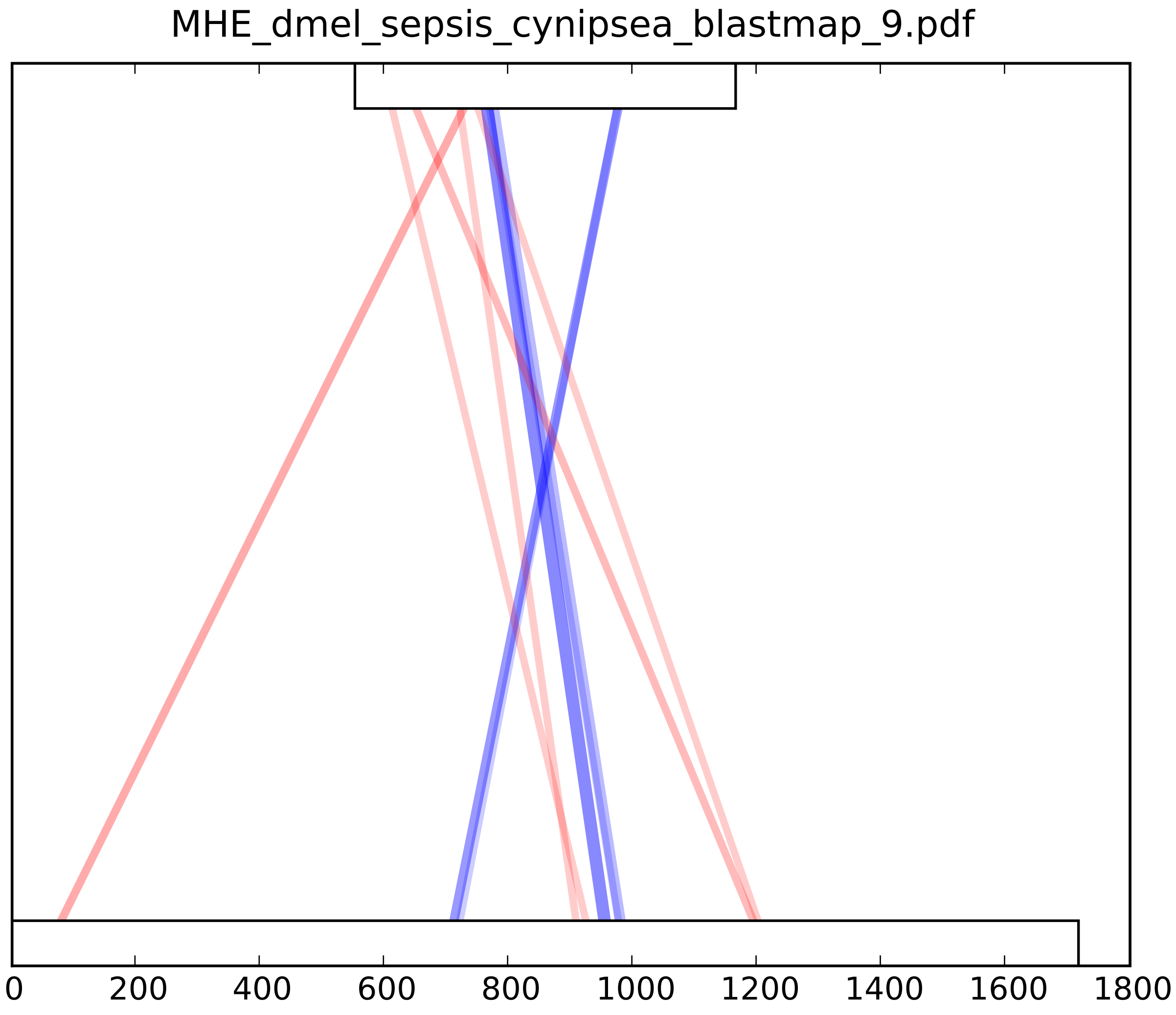
MHE_dmel_sepsis_cynipsea_blastmap_9.pdf
0
200
400
600
800
1000
1200
1400
1600
1800

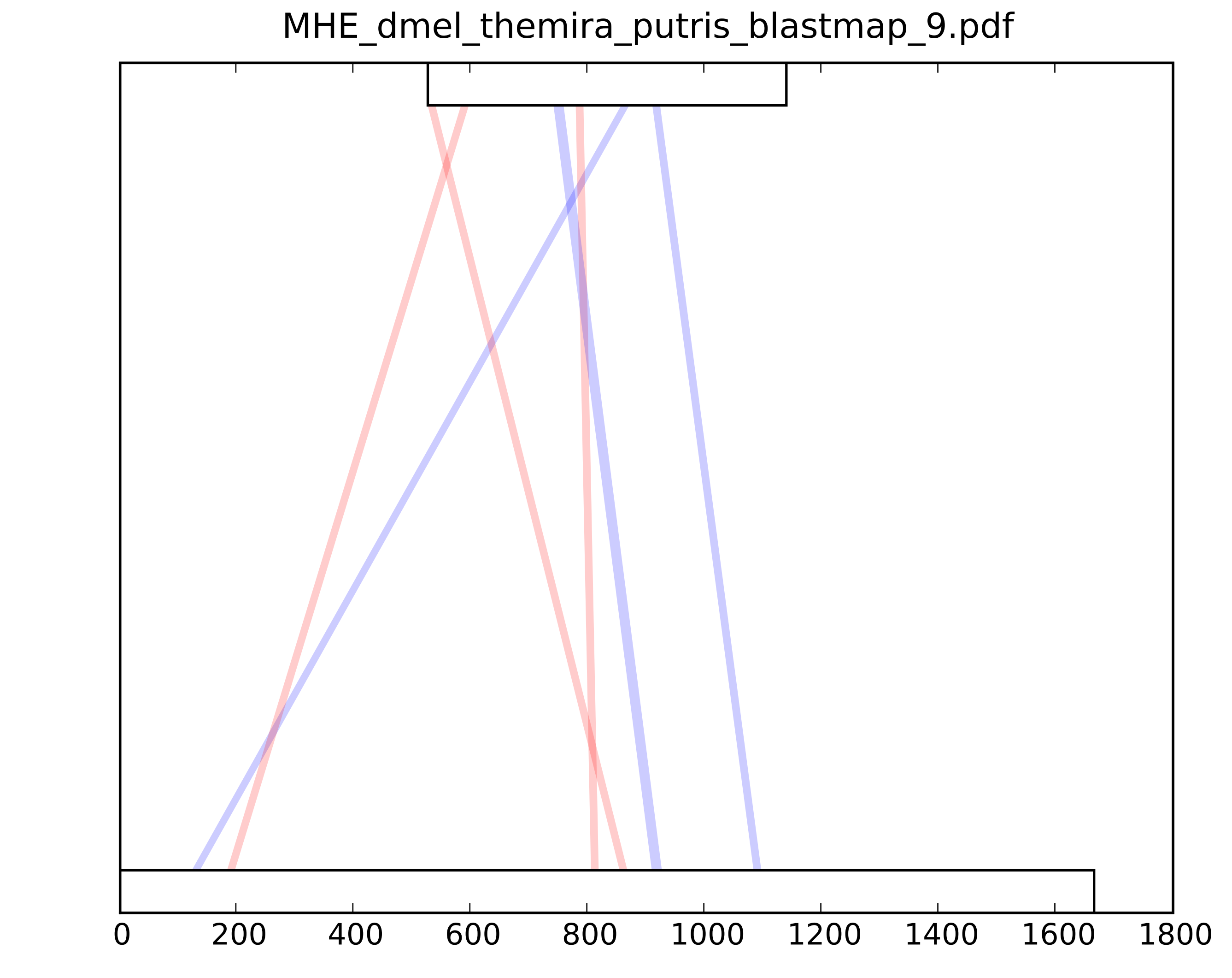
MHE_dmel_themira_putris_blastmap_9.pdf
0
200
400
600
800
1000
1200
1400
1600
1800

st2_dmel_dpse_blastmap_7.pdf

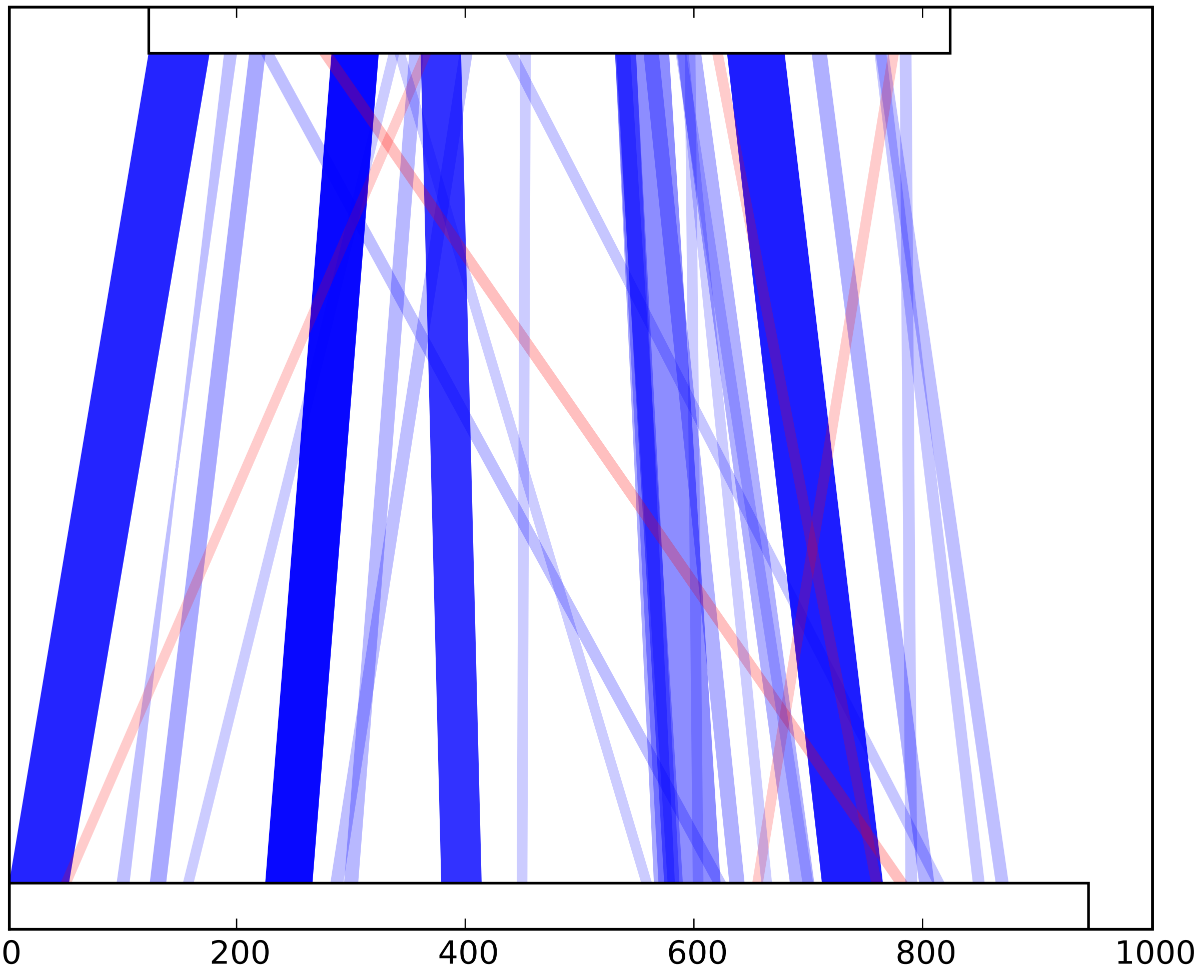

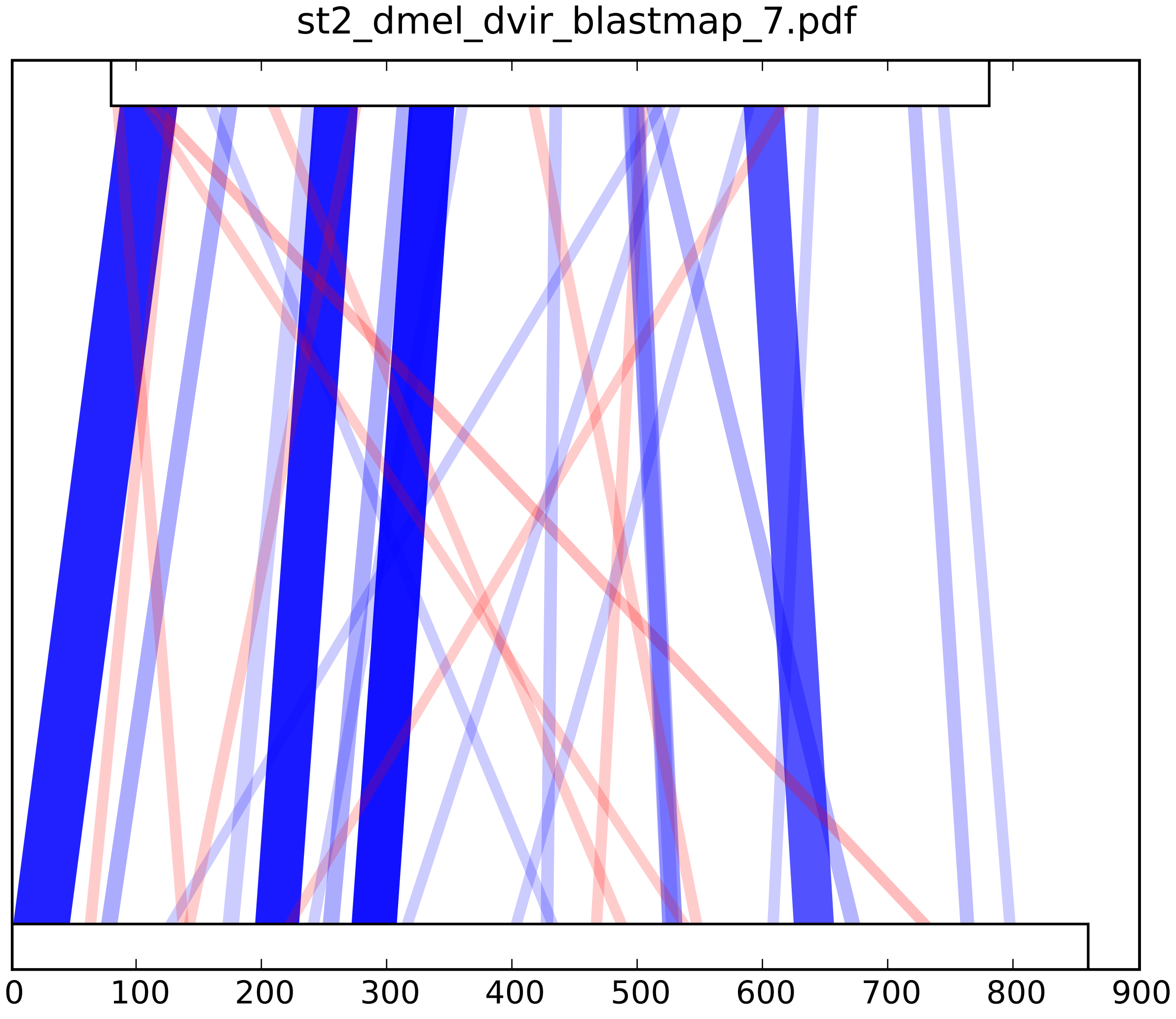
st2_dmel_dvir_blastmap_7.pdf
0
100
200
300
400
500
600
700
800
900

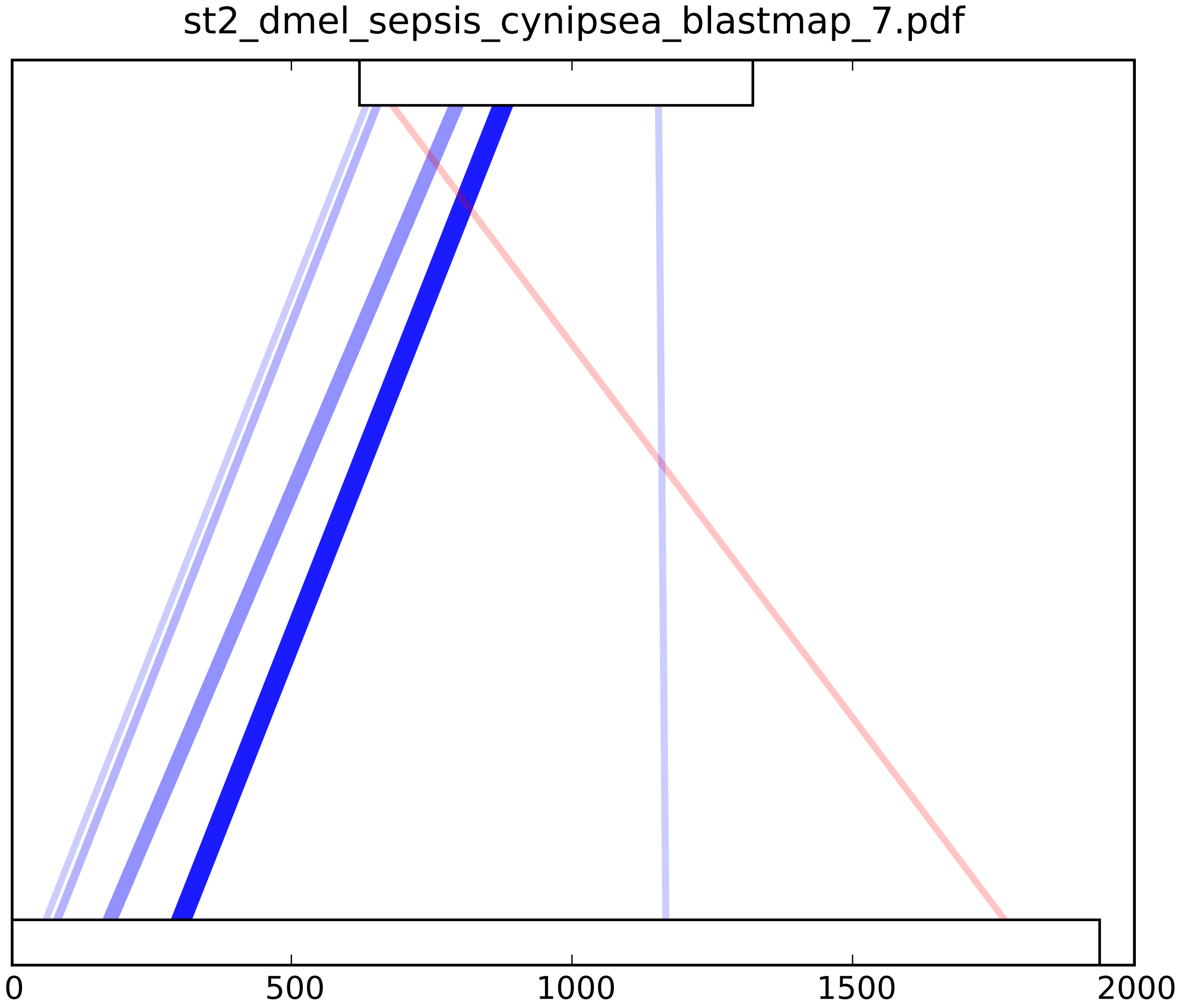
st2_dmel_sepsis_cynipsea_blastmap_7.pdf
0
500
1000
1500
2000

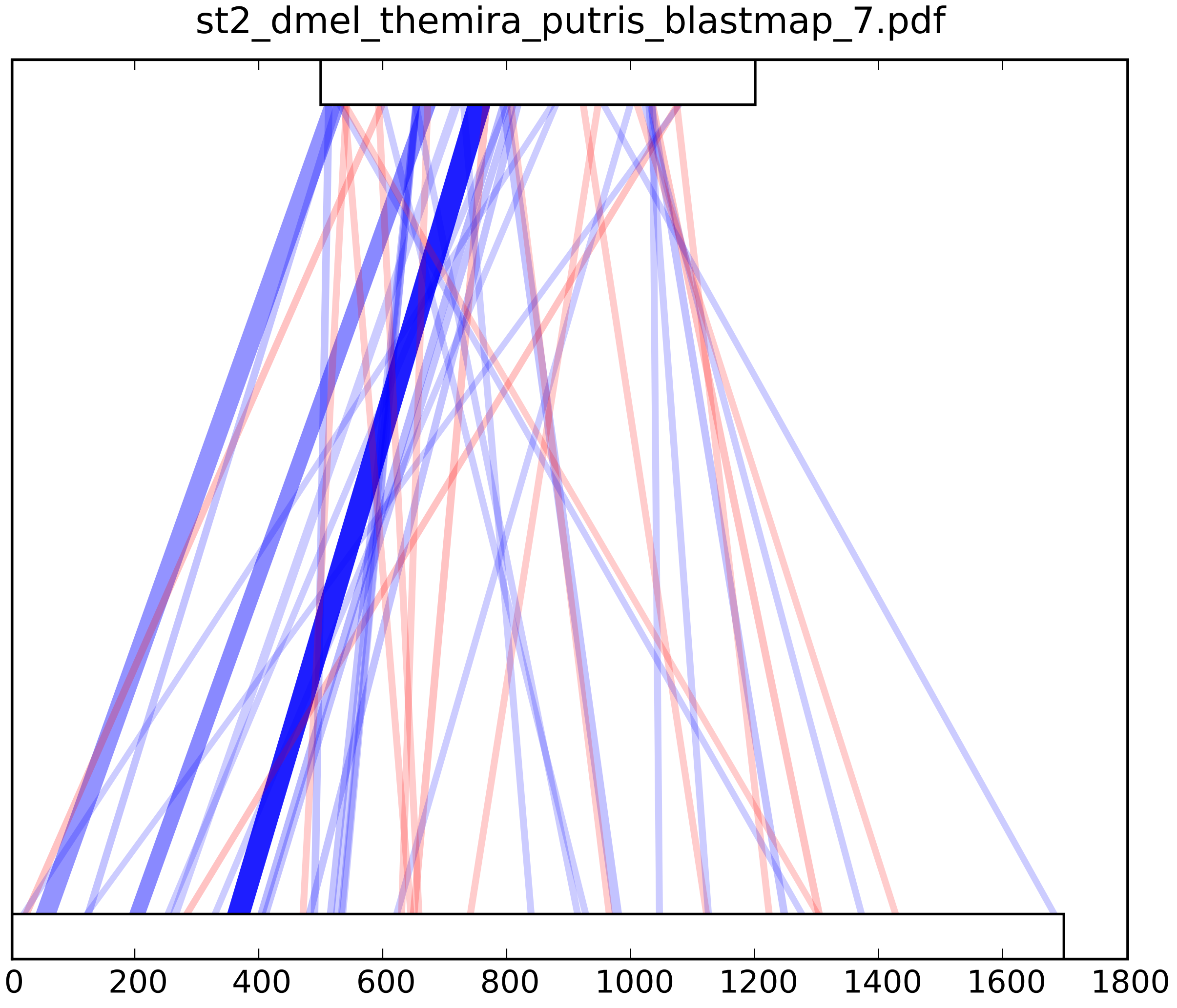
st2_dmel_themira_putris_blastmap_7.pdf
0
200
400
600
800
1000
1200
1400
1600
1800

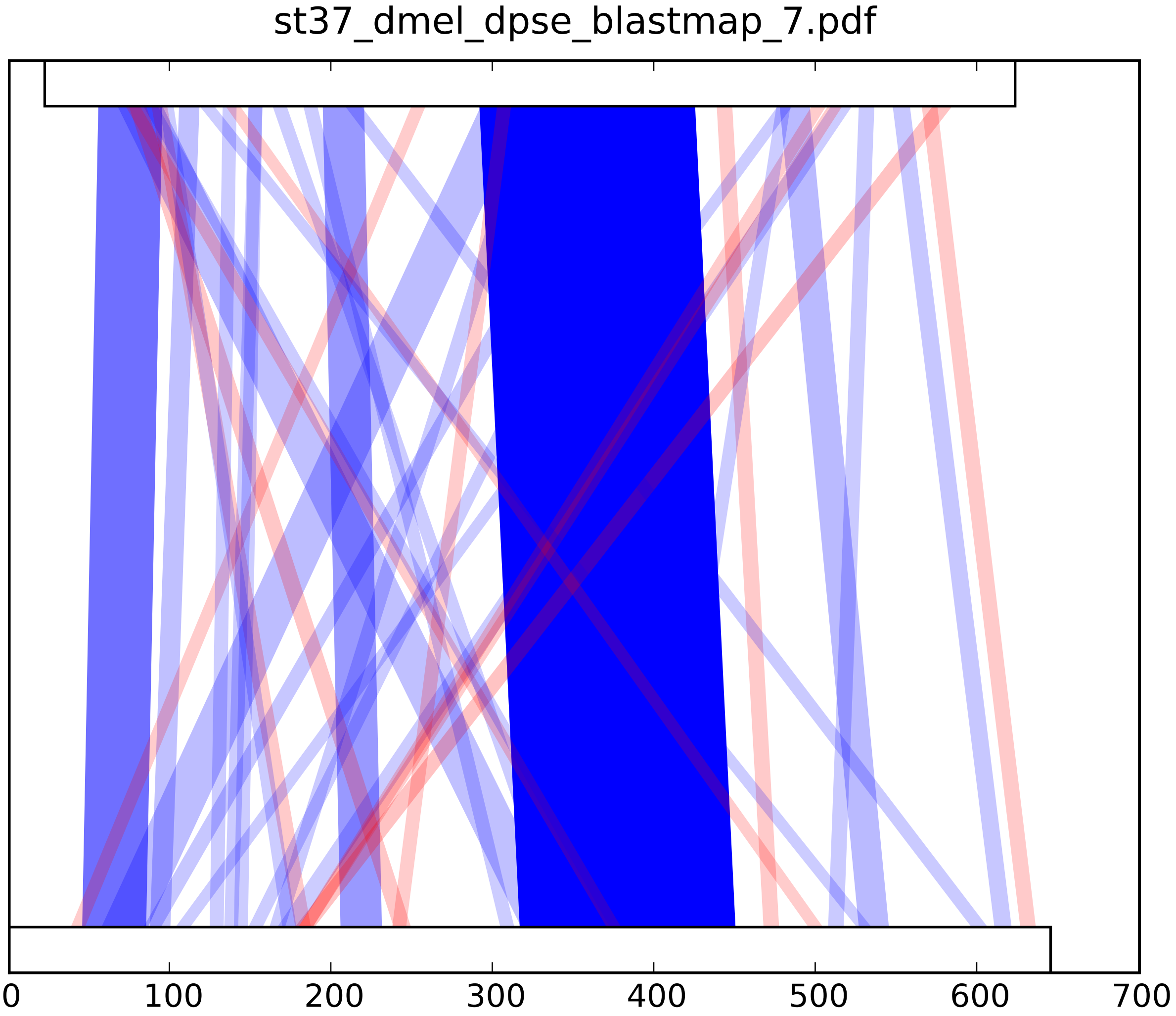
st37_dmel_dpse_blastmap_7.pdf
0
100
200
300
400
500
600
700

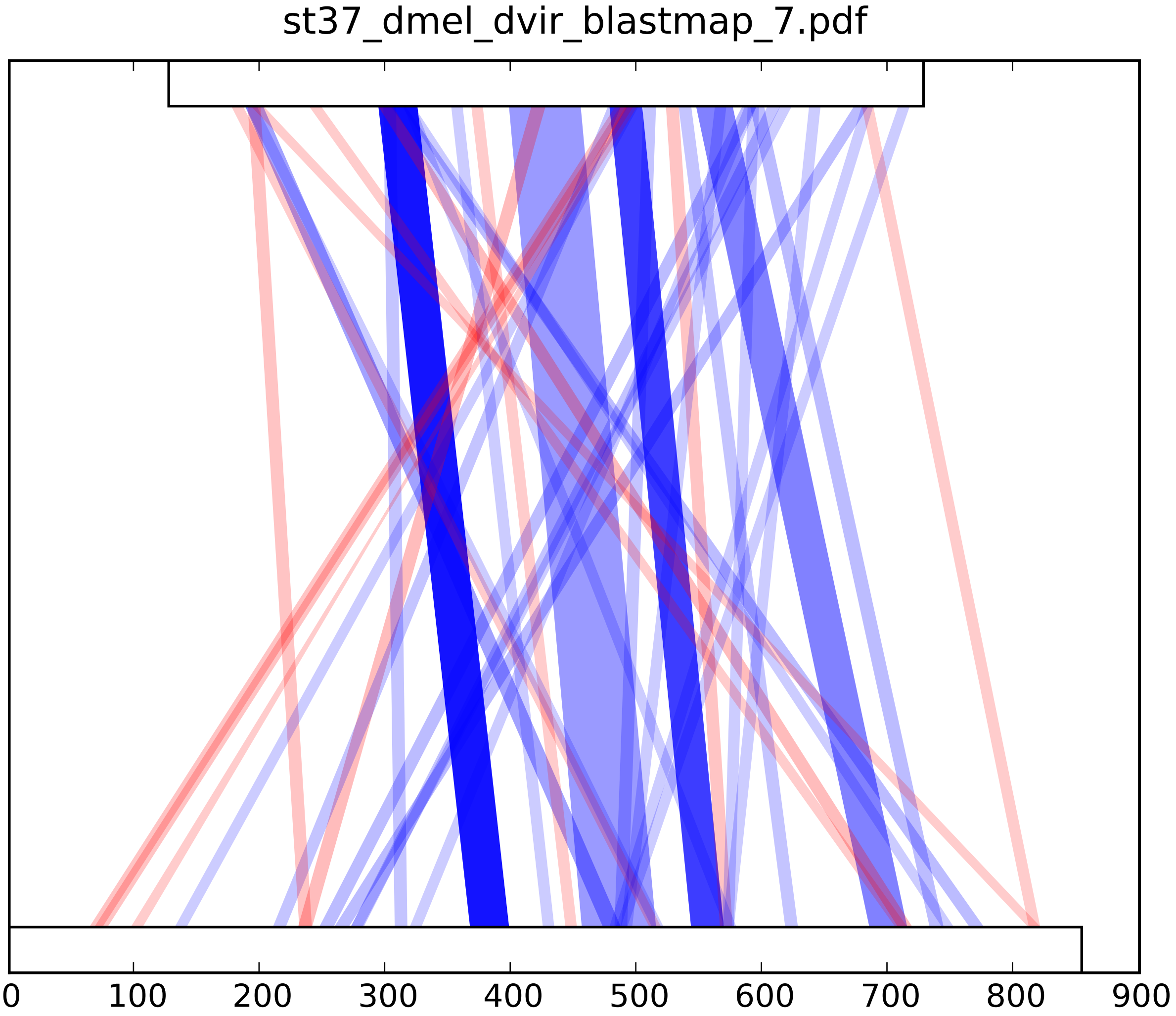
st37_dmel_dvir_blastmap_7.pdf
0
100
200
300
400
500
600
700
800
900

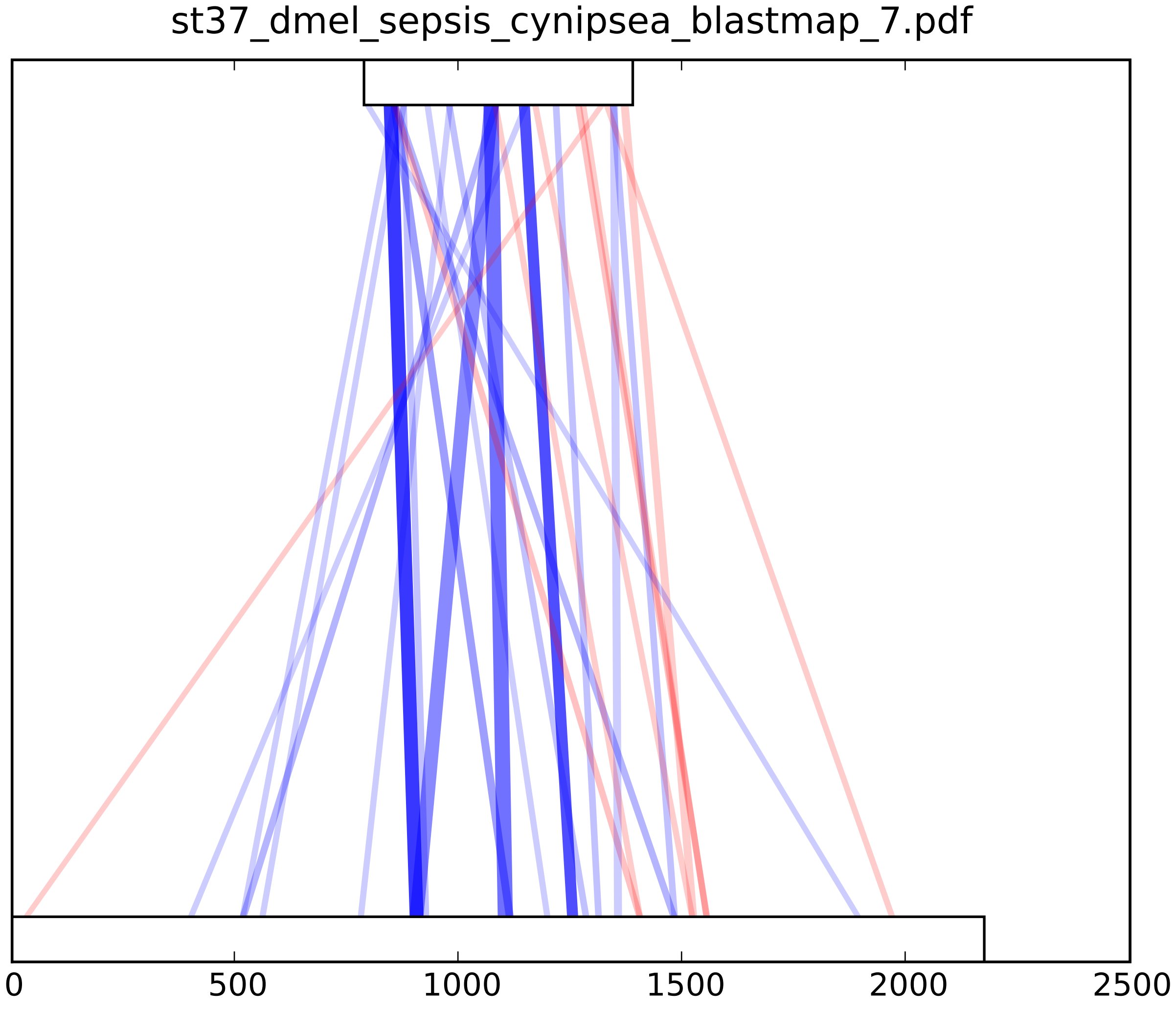

st37_dmel_sepsis_cynipsea_blastmap_7.pdf
0
500
1000
1500
2000
2500

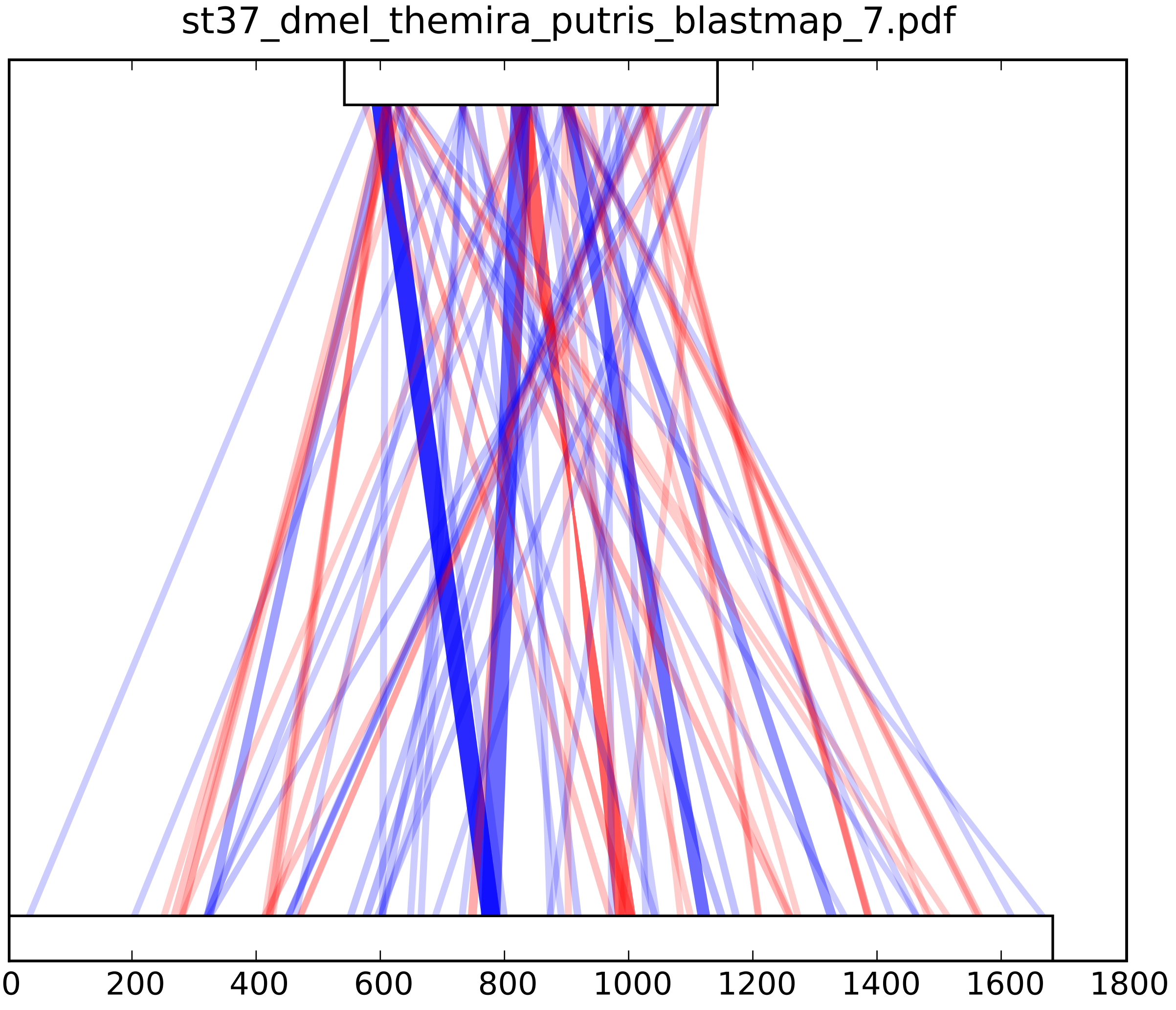

st37_dmel_themira_putris_blastmap_7.pdf
0
200
400
600
800
1000
1200
1400
1600
1800

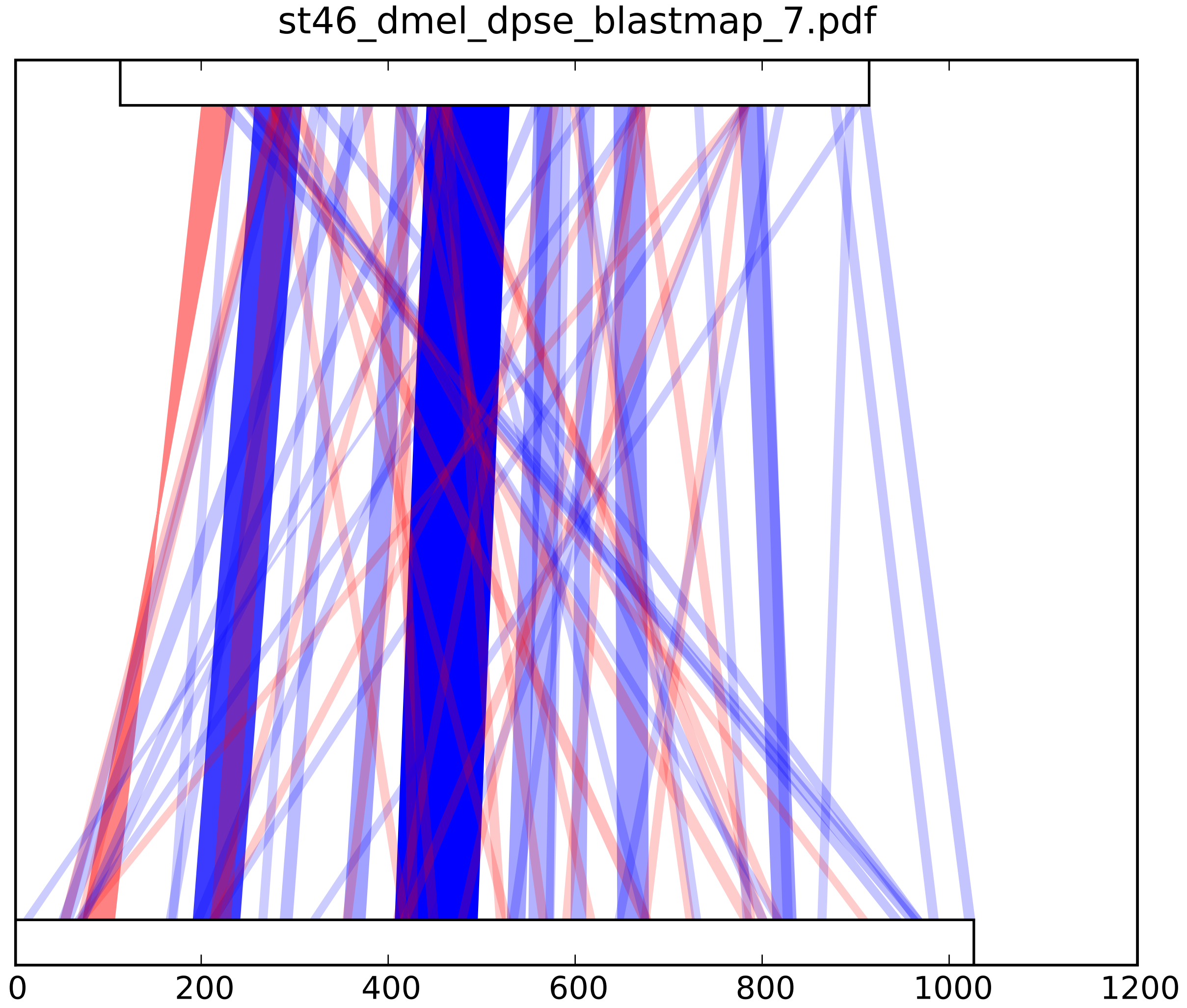
st46_dmel_dpse_blastmap_7.pdf
0
200
400
600
800
1000
1200

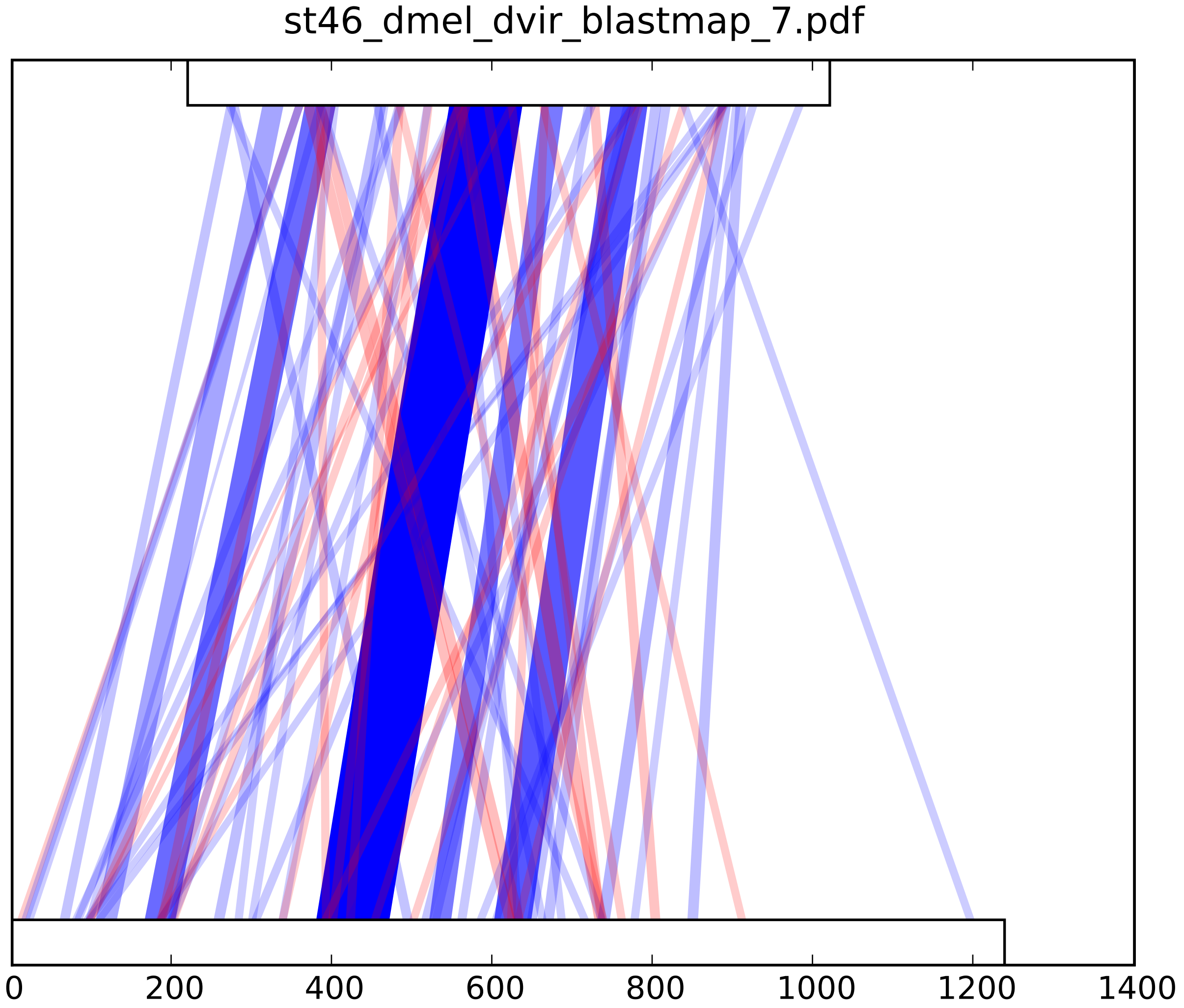
st46_dmel_dvir_blastmap_7.pdf
0
200
400
600
800
1000
1200
1400

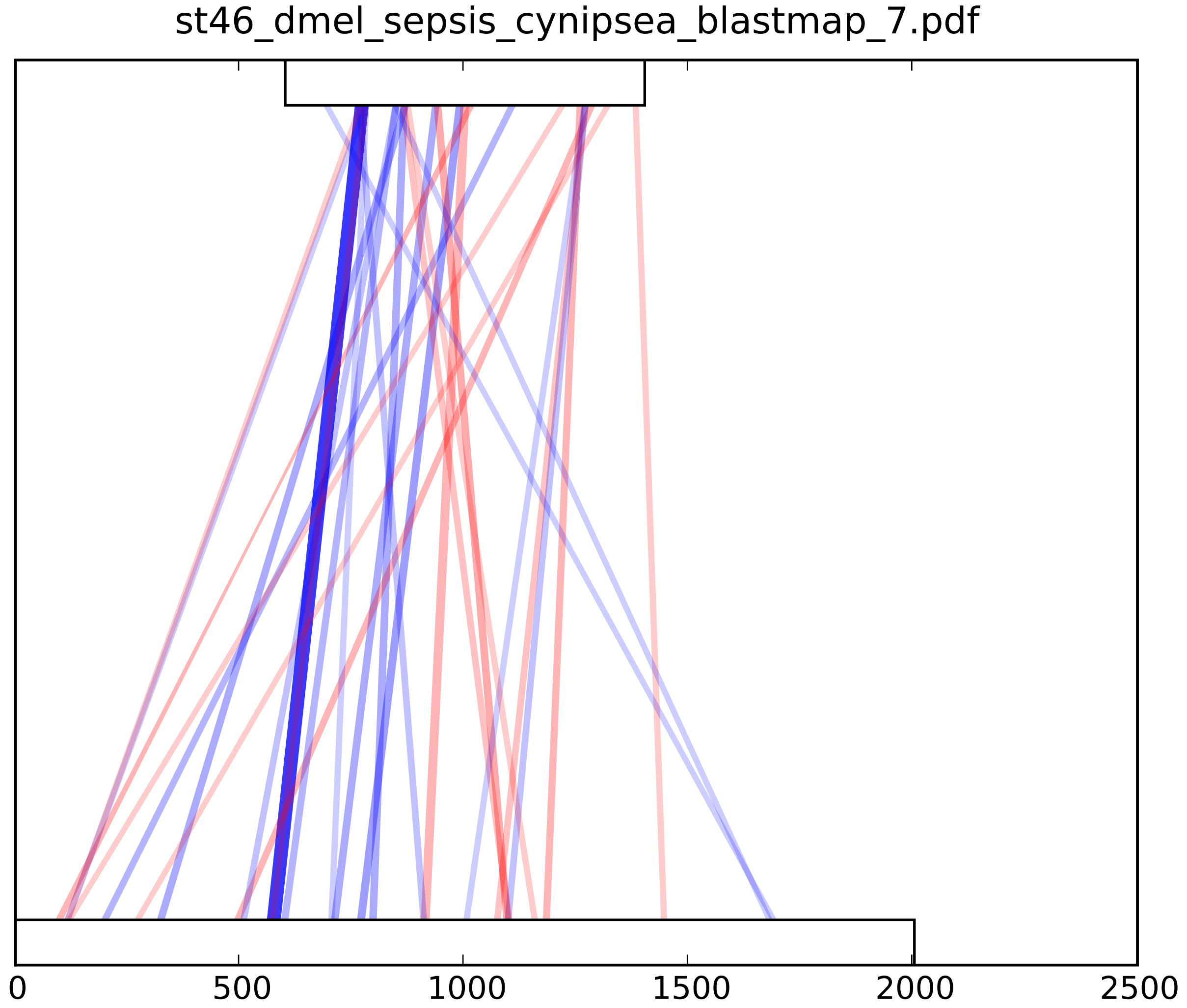
st46_dmel_sepsis_cynipsea_blastmap_7.pdf
0
500
1000
1500
2000
2500

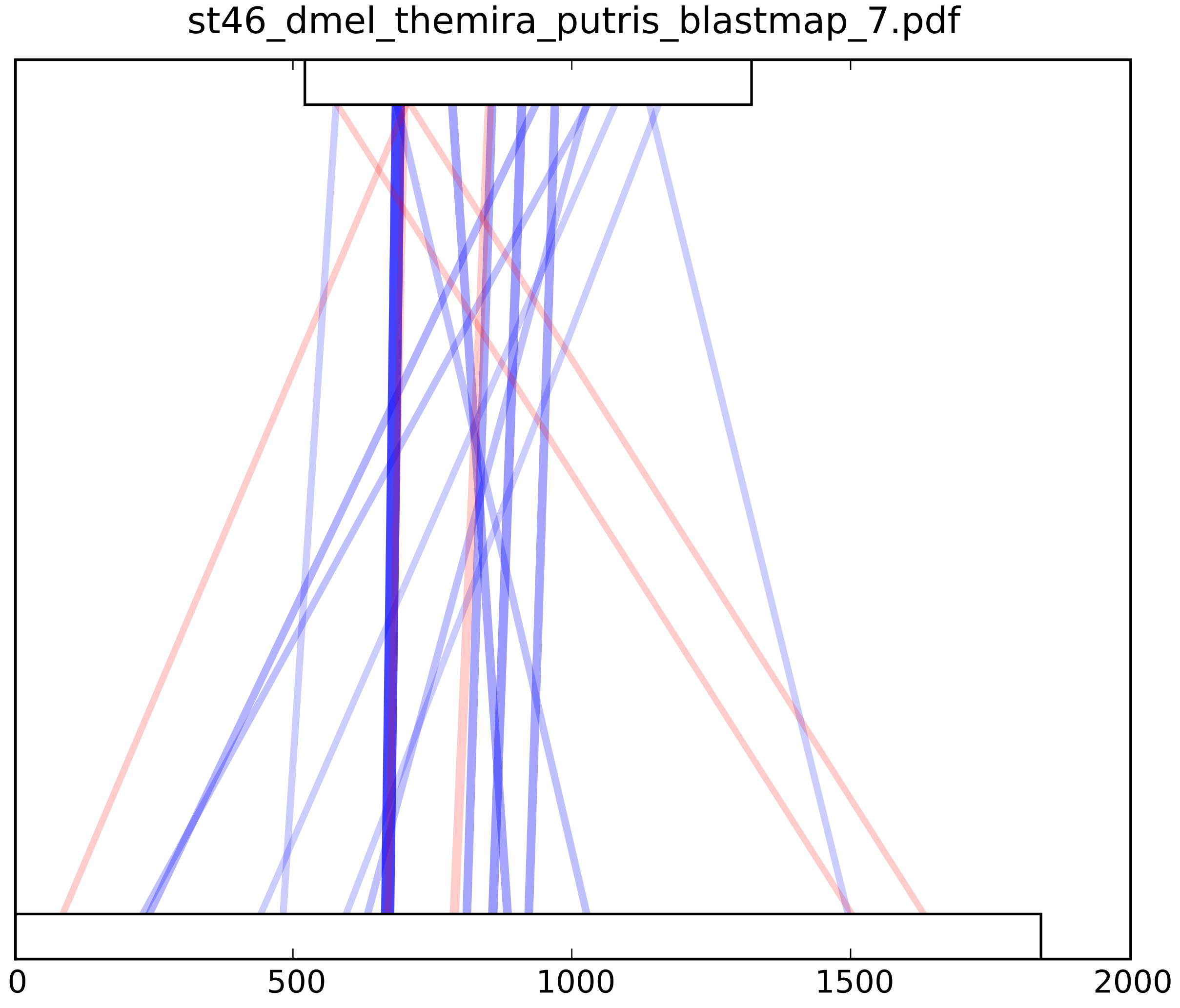
st46_dmel_themira_putris_blastmap_7.pdf
0
500
1000
1500
2000

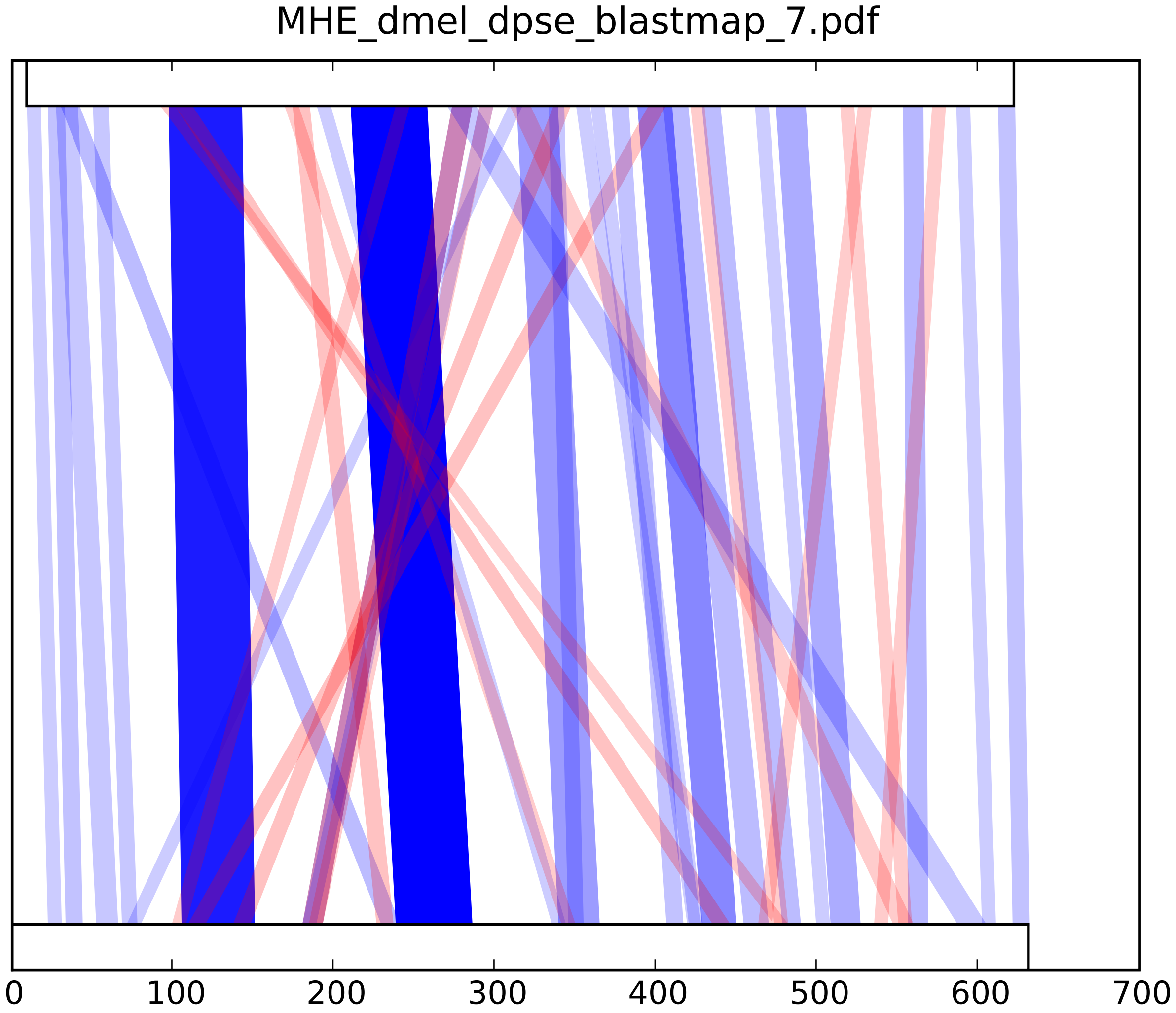

MHE_dmel_dpse_blastmap_7.pdf
0
100
200
300
400
500
600
700

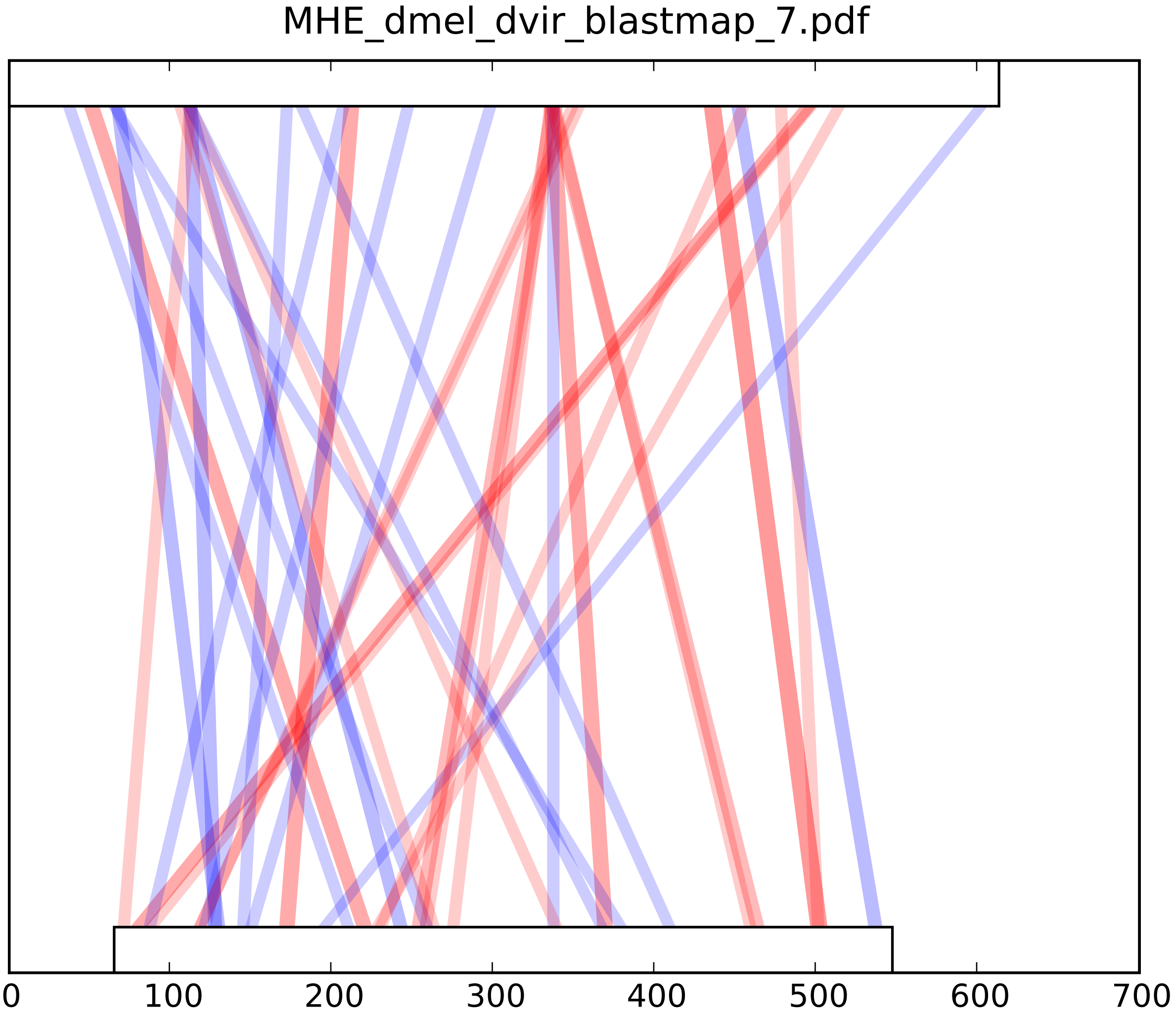
MHE_dmel_dvir_blastmap_7.pdf
0
100
200
300
400
500
600
700

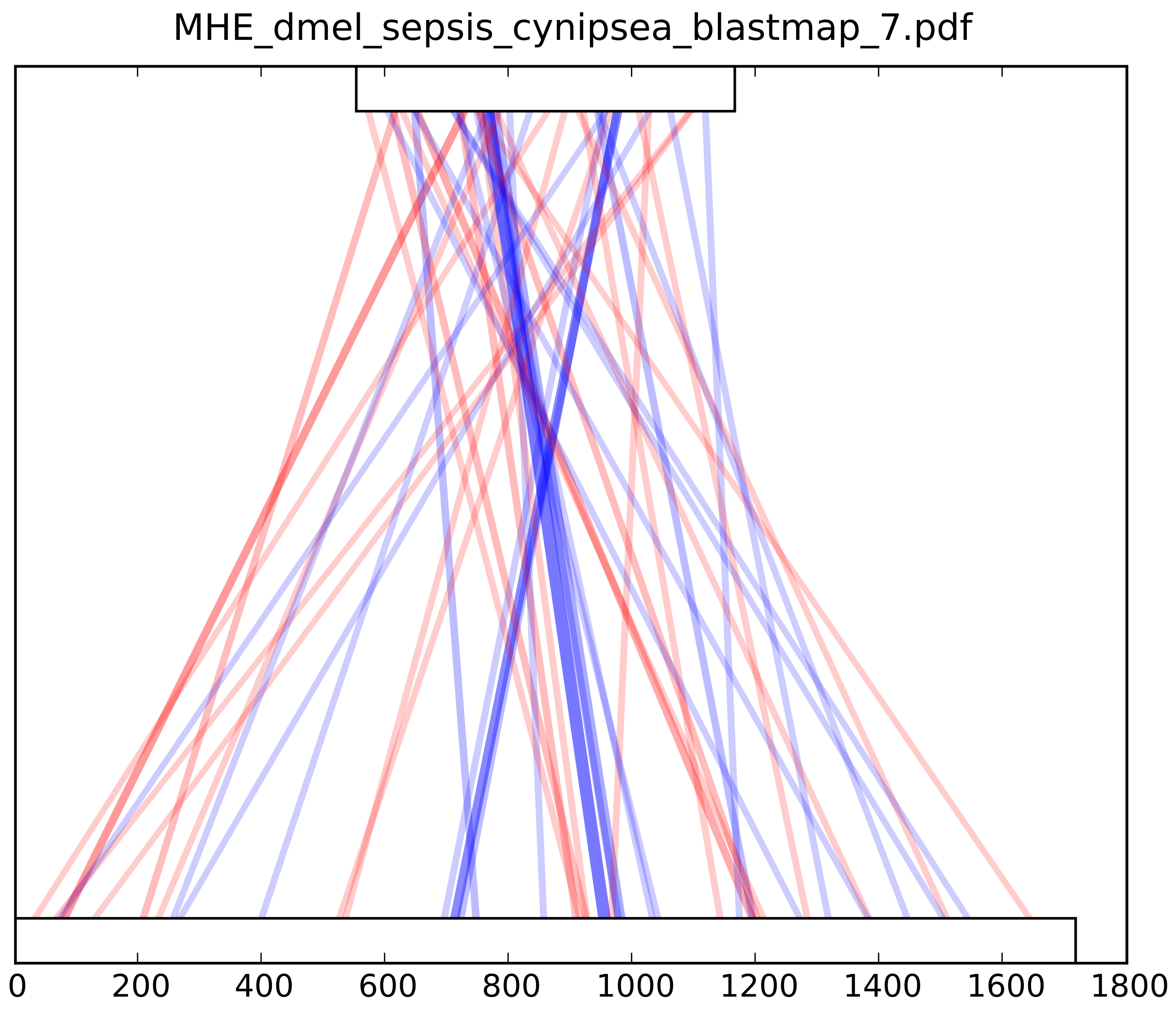

MHE_dmel_sepsis_cynipsea_blastmap_7.pdf
0
200
400
600
800
1000
1200
1400
1600
1800

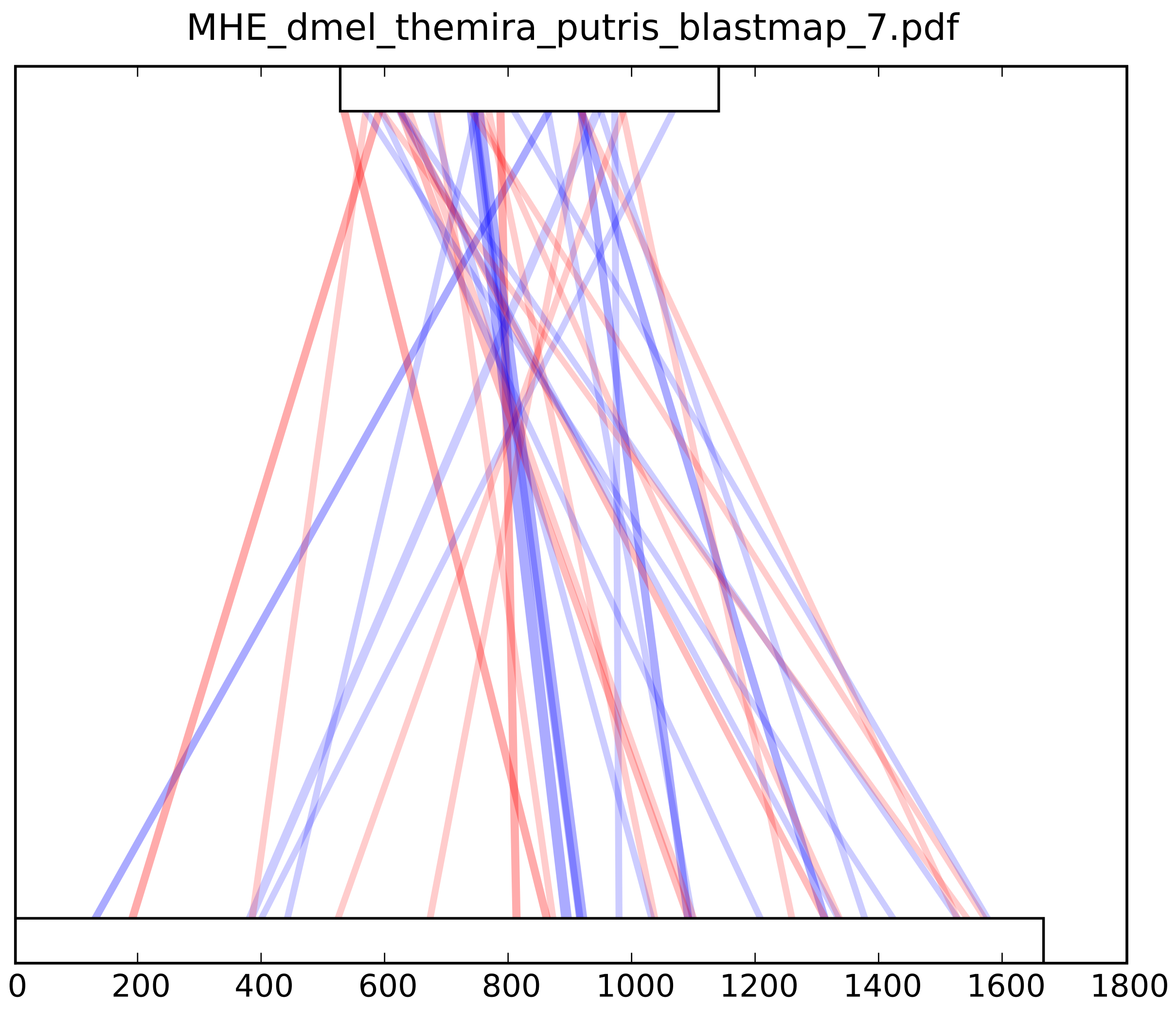

MHE_dmel_themira_putris_blastmap_7.pdf
0
200
400
600
800
1000
1200
1400
1600
1800

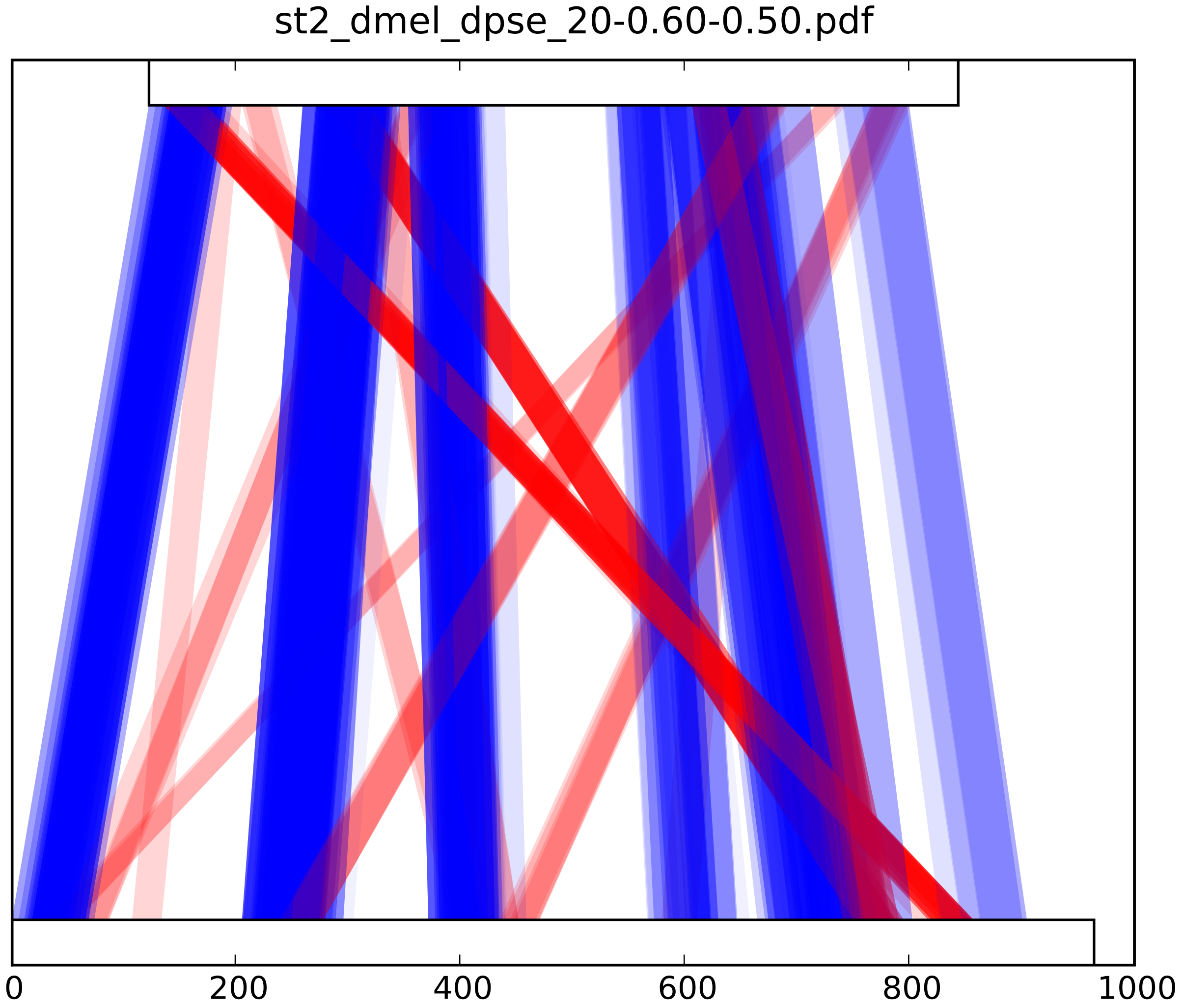
st2_dmel_dpse_20-0.60-0.50.pdf
0
200
400
600
800
1000

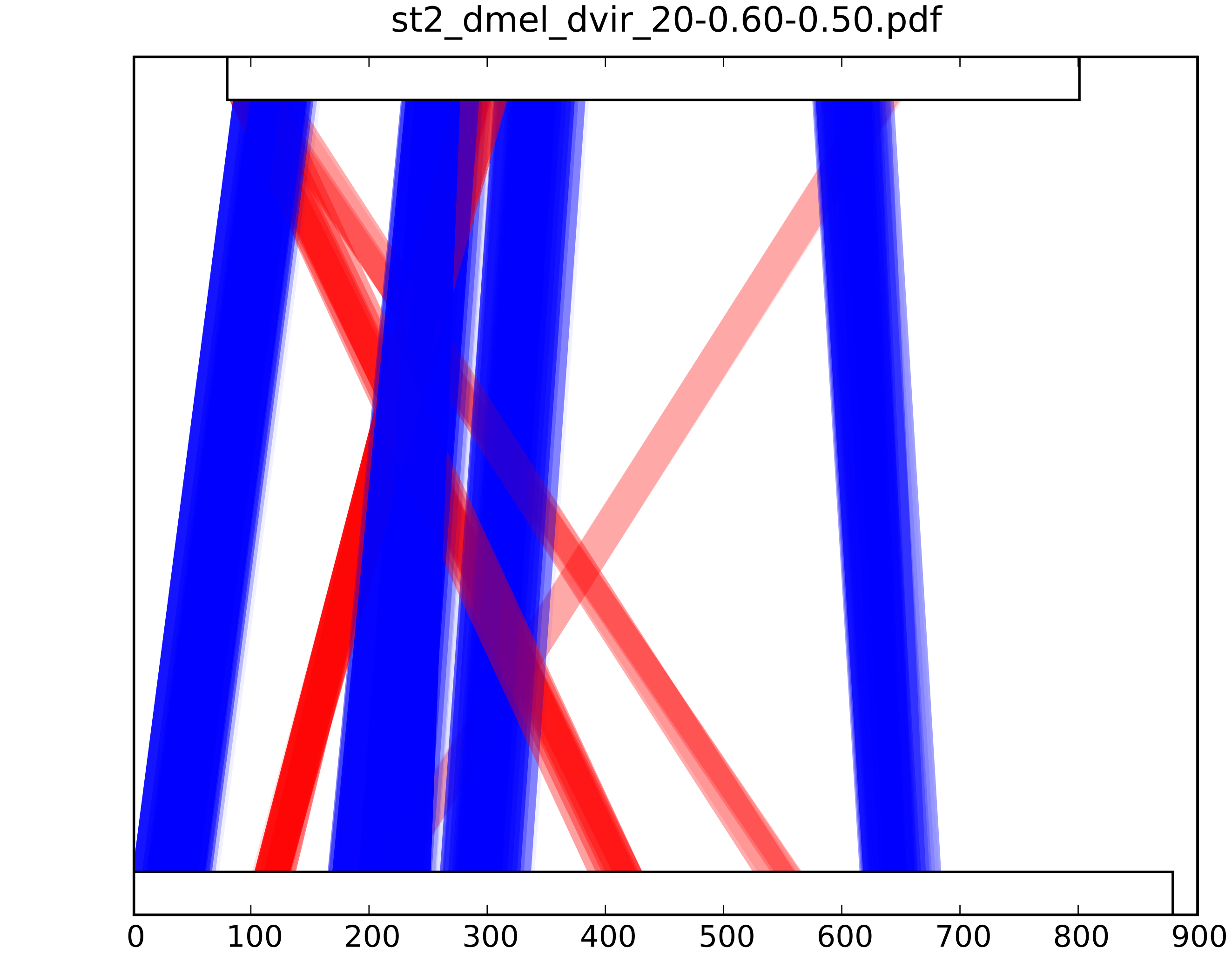
st2_dmel_dvir_20-0.60-0.50.pdf
0
100
200
300
400
500
600
700
800
900

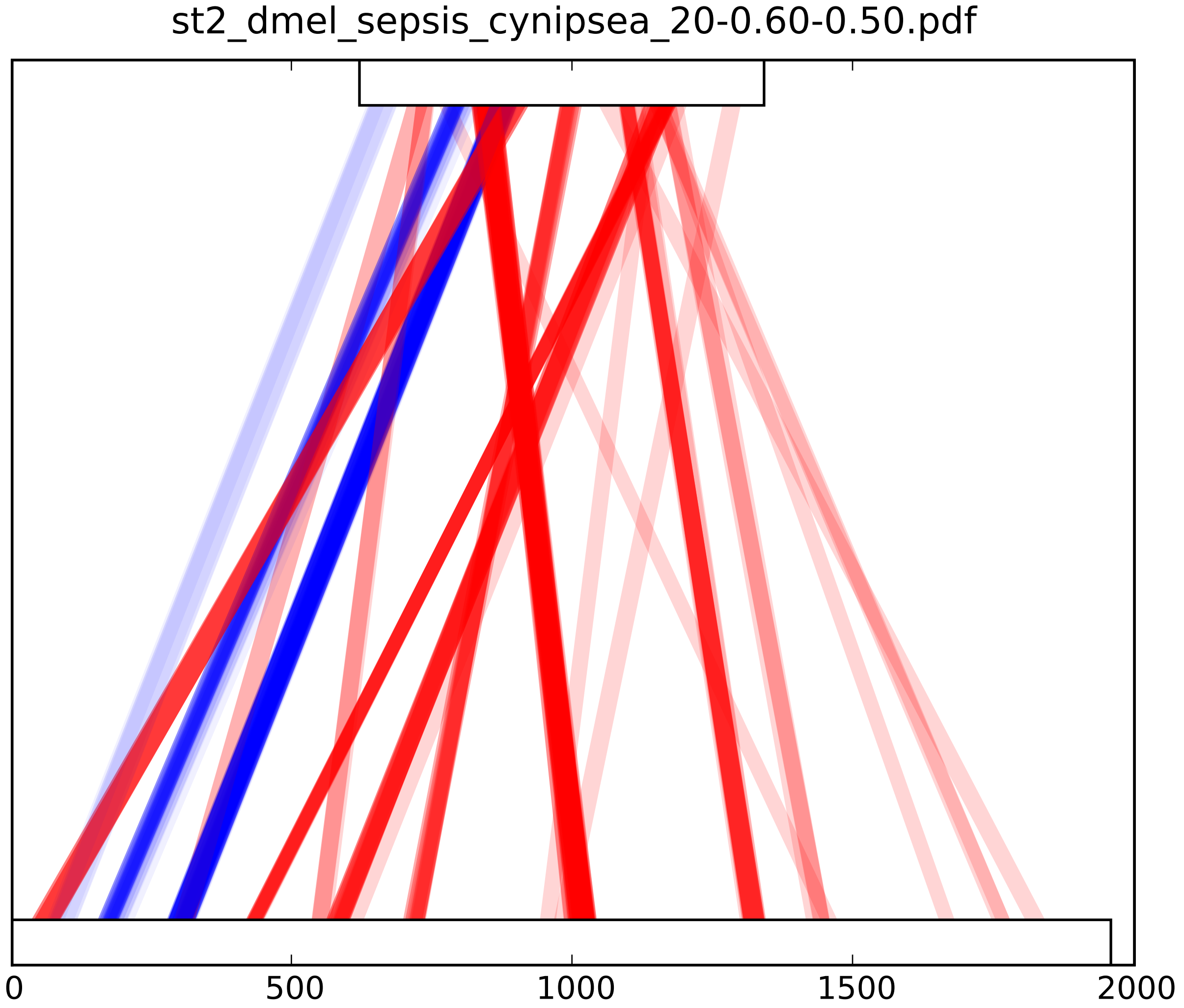
st2_dmel_sepsis_cynipsea_20-0.60-0.50.pdf
0
500
1000
1500
2000

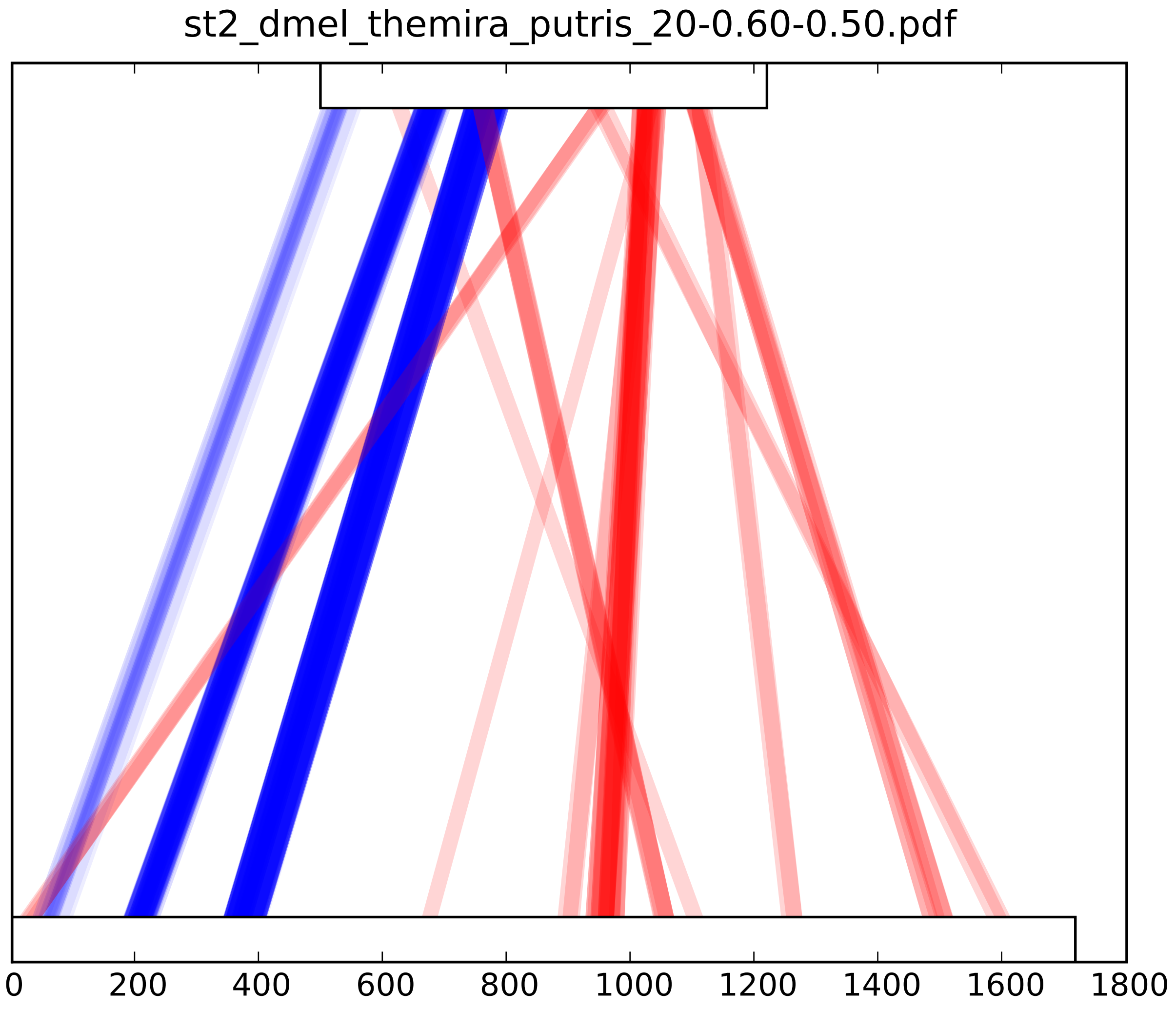
st2_dmel_themira_putris_20-0.60-0.50.pdf
0
200
400
600
800
1000
1200
1400
1600
1800

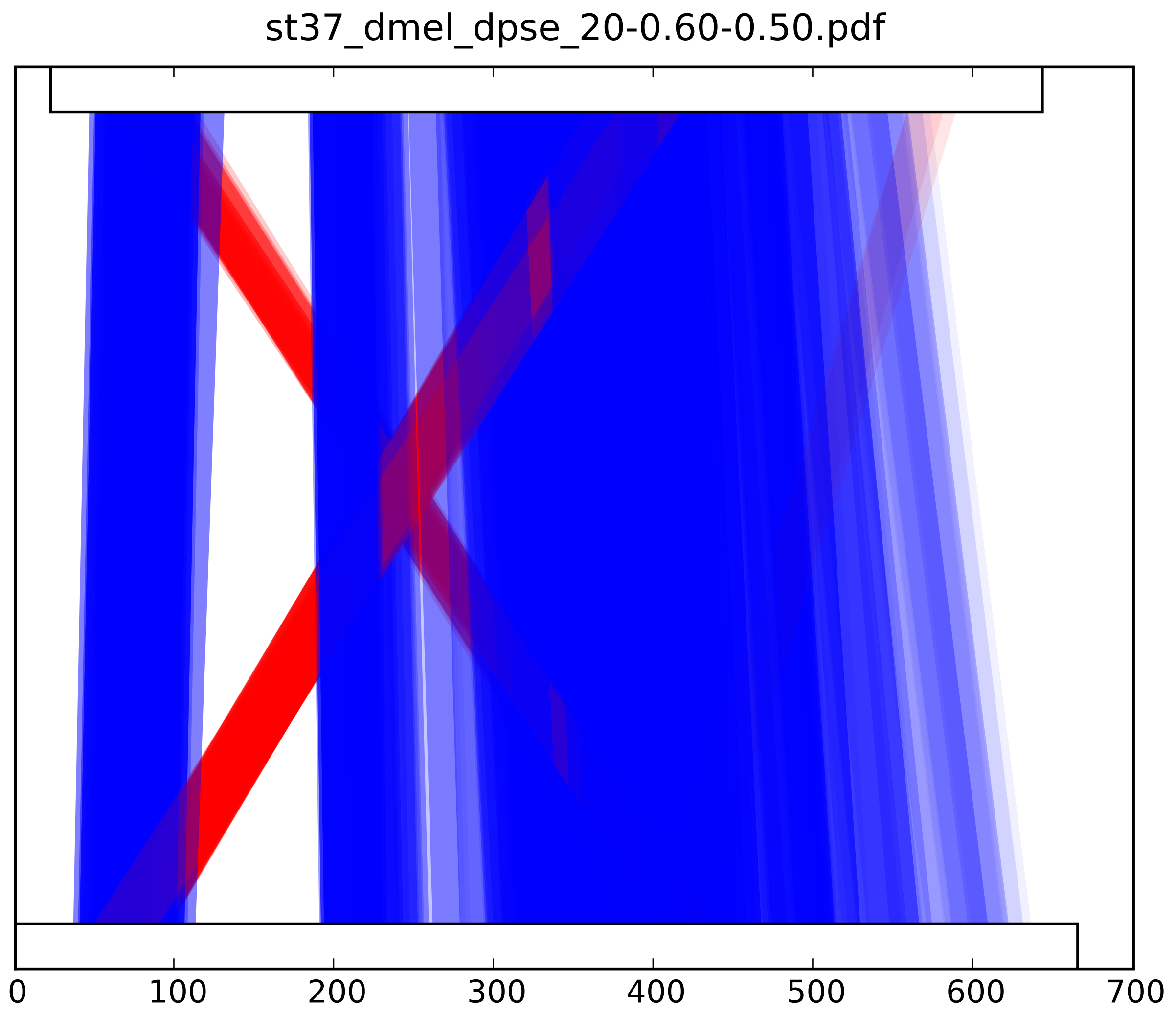
st37_dmel_dpse_20-0.60-0.50.pdf
0
100
200
300
400
500
600
700

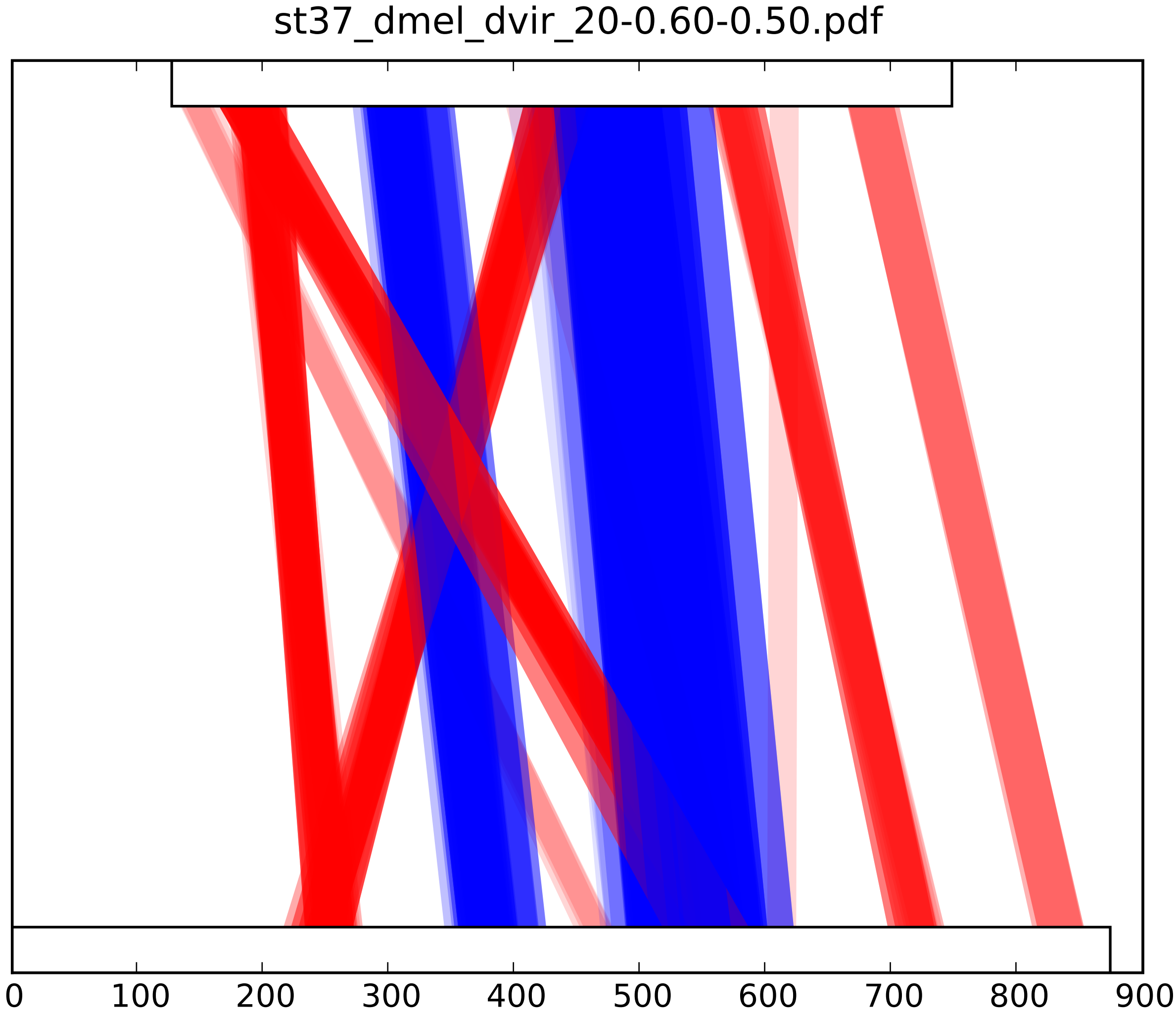

st37_dmel_dvir_20-0.60-0.50.pdf
0
100
200
300
400
500
600
700
800
900

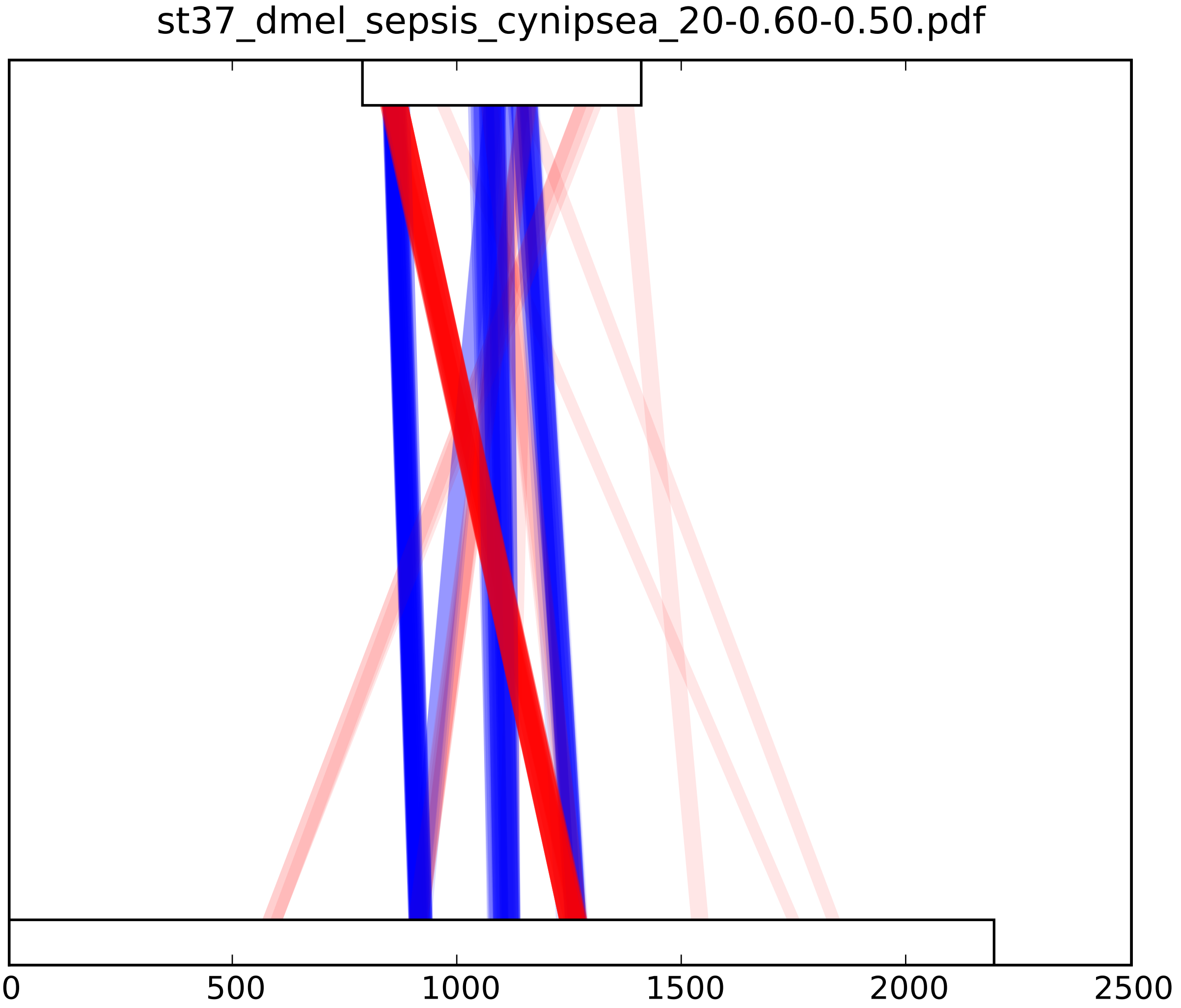
st37_dmel_sepsis_cynipsea_20-0.60-0.50.pdf
0
500
1000
1500
2000
2500

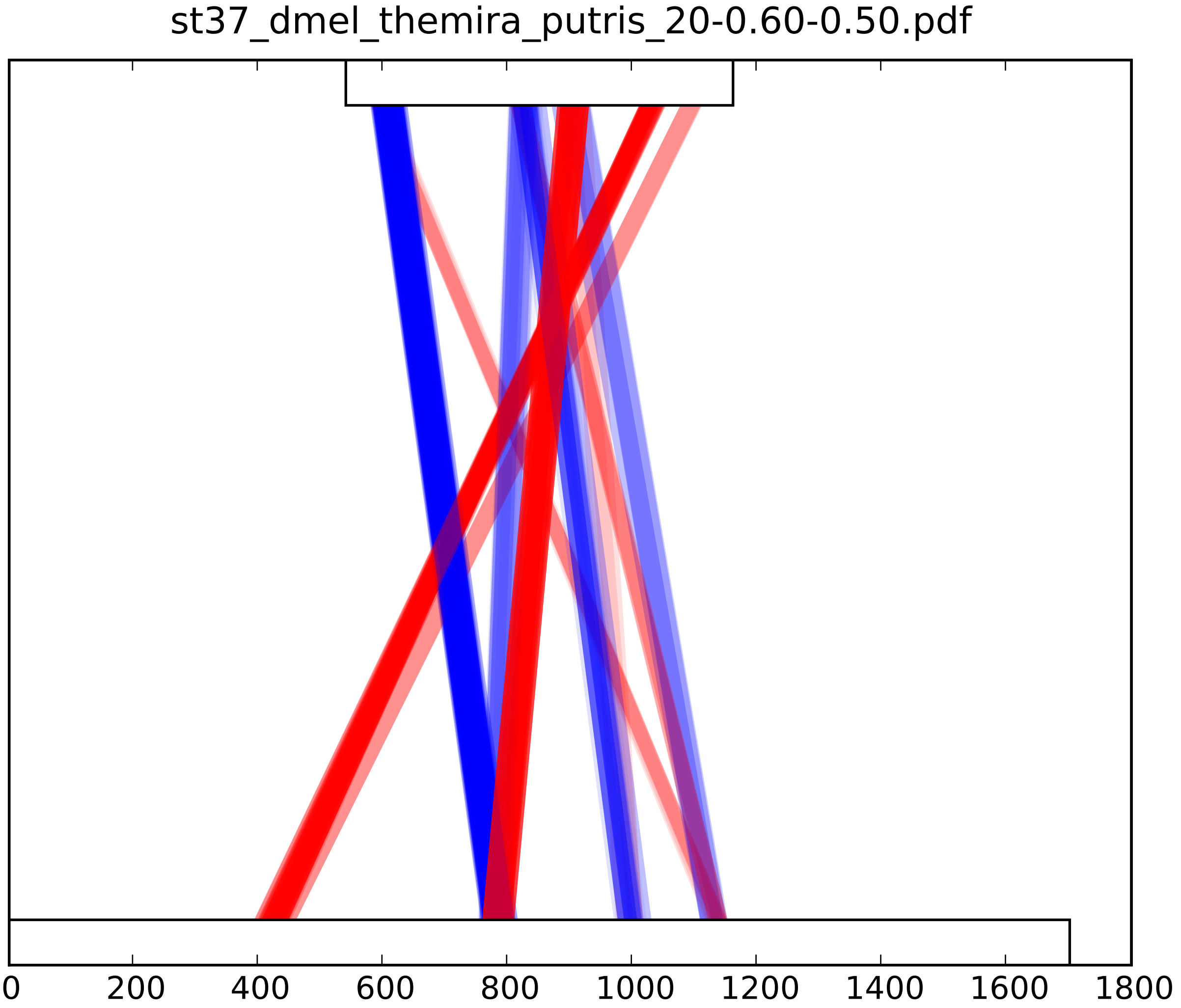
st37_dmel_themira_putris_20-0.60-0.50.pdf
0
200
400
600
800
1000
1200
1400
1600
1800

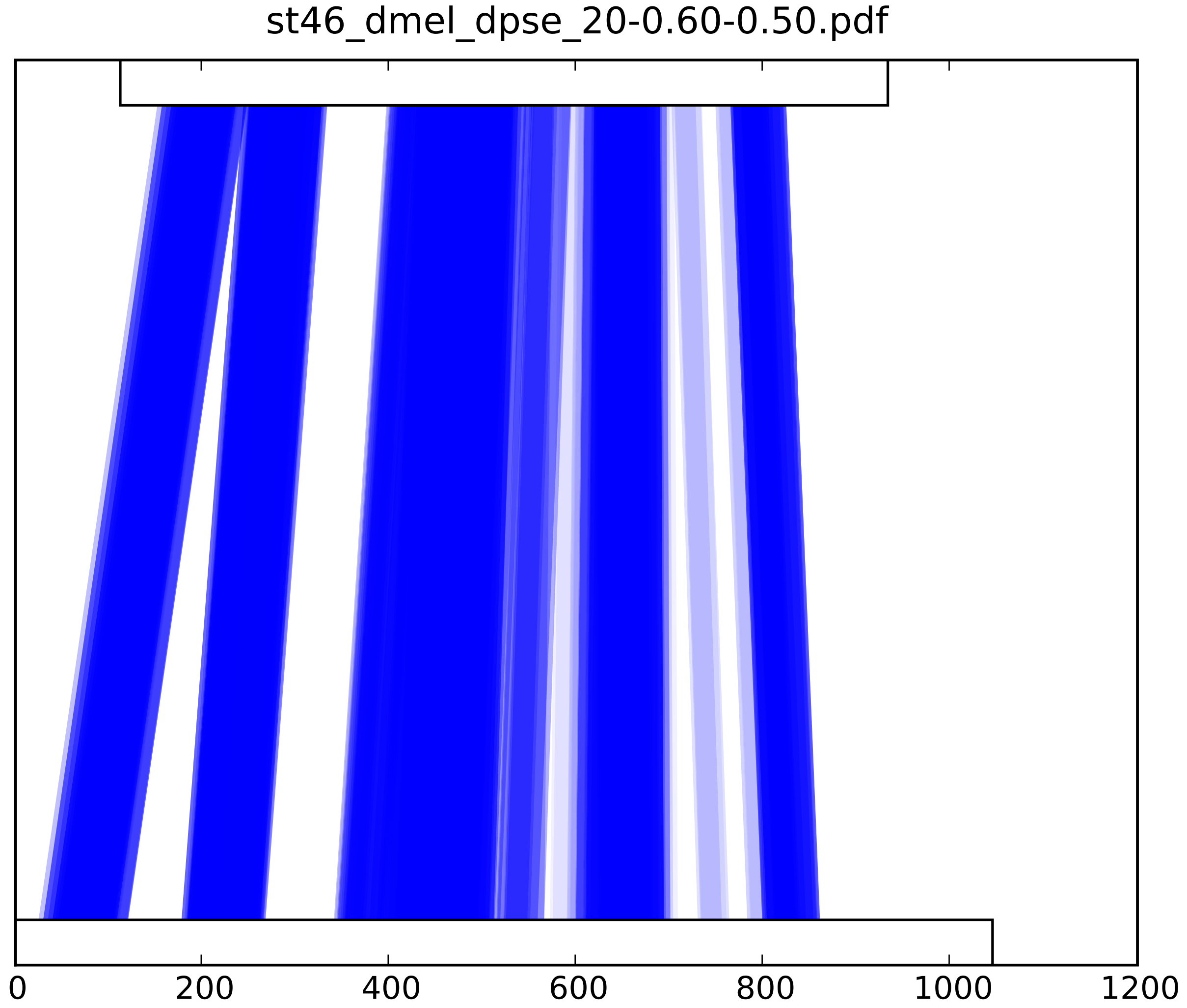
st46_dmel_dpse_20-0.60-0.50.pdf
0
200
400
600
800
1000
1200

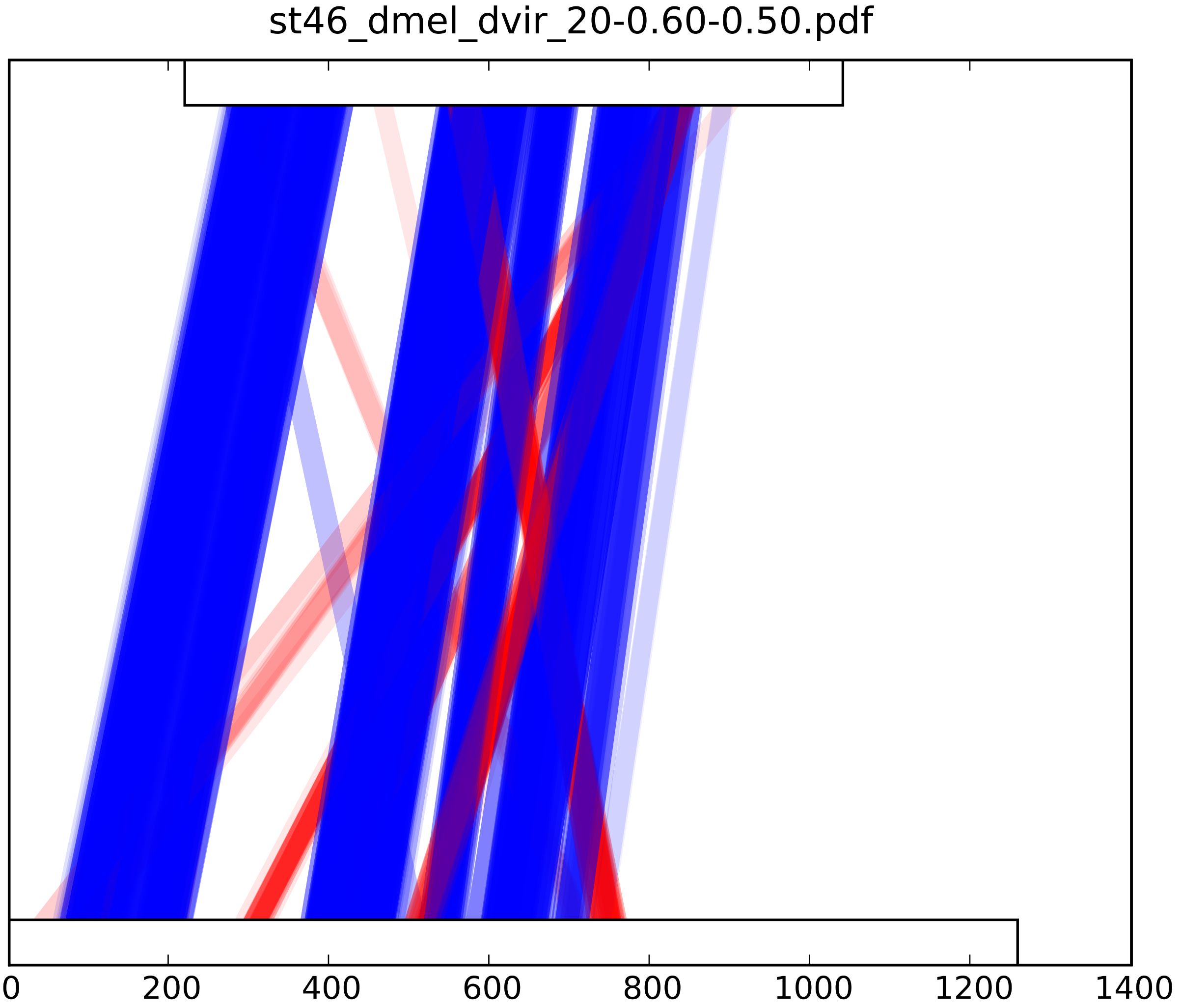

st46_dmel_dvir_20-0.60-0.50.pdf
0
200
400
600
800
1000
1200
1400

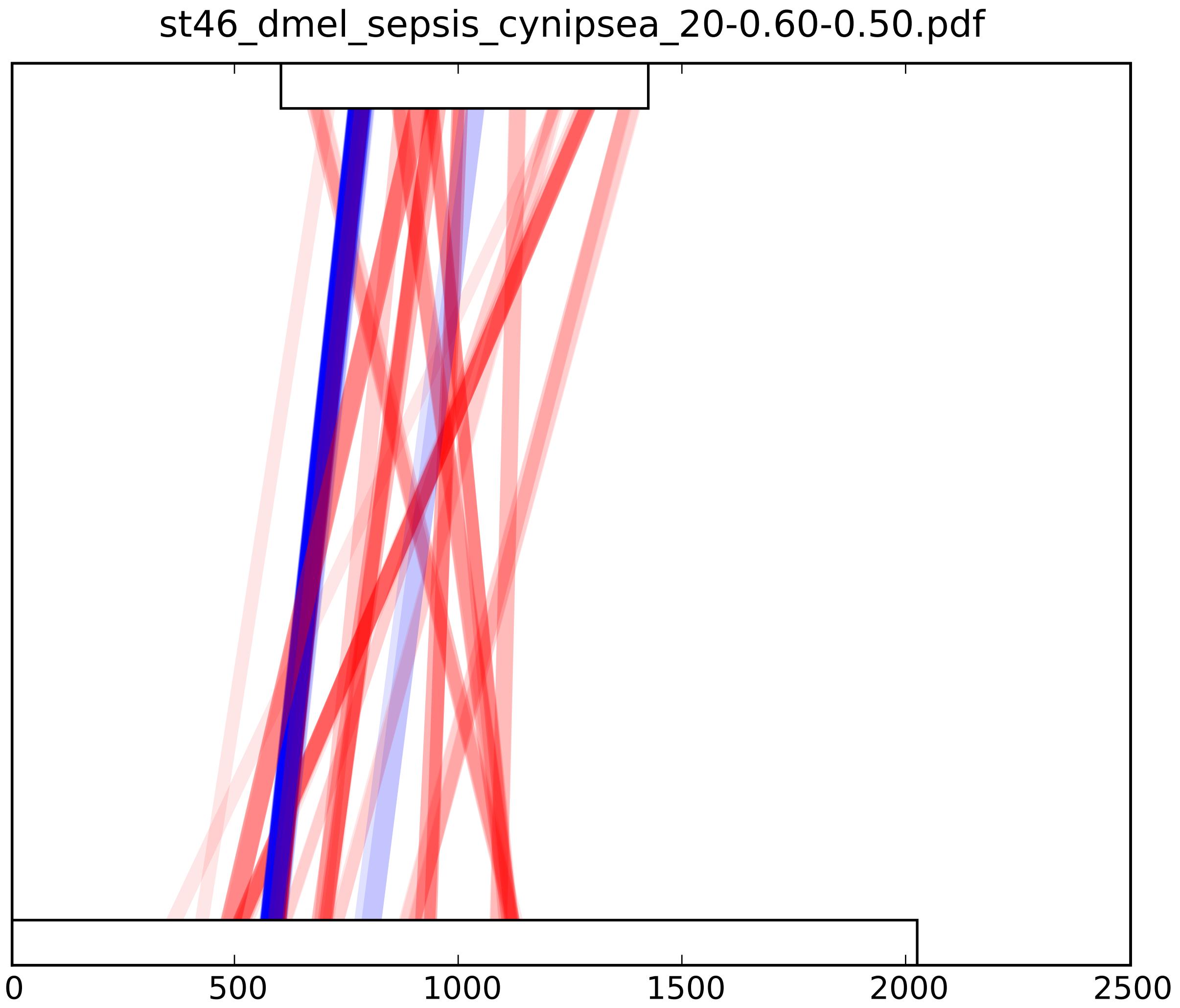
st46_dmel_sepsis_cynipsea_20-0.60-0.50.pdf
0
500
1000
1500
2000
2500

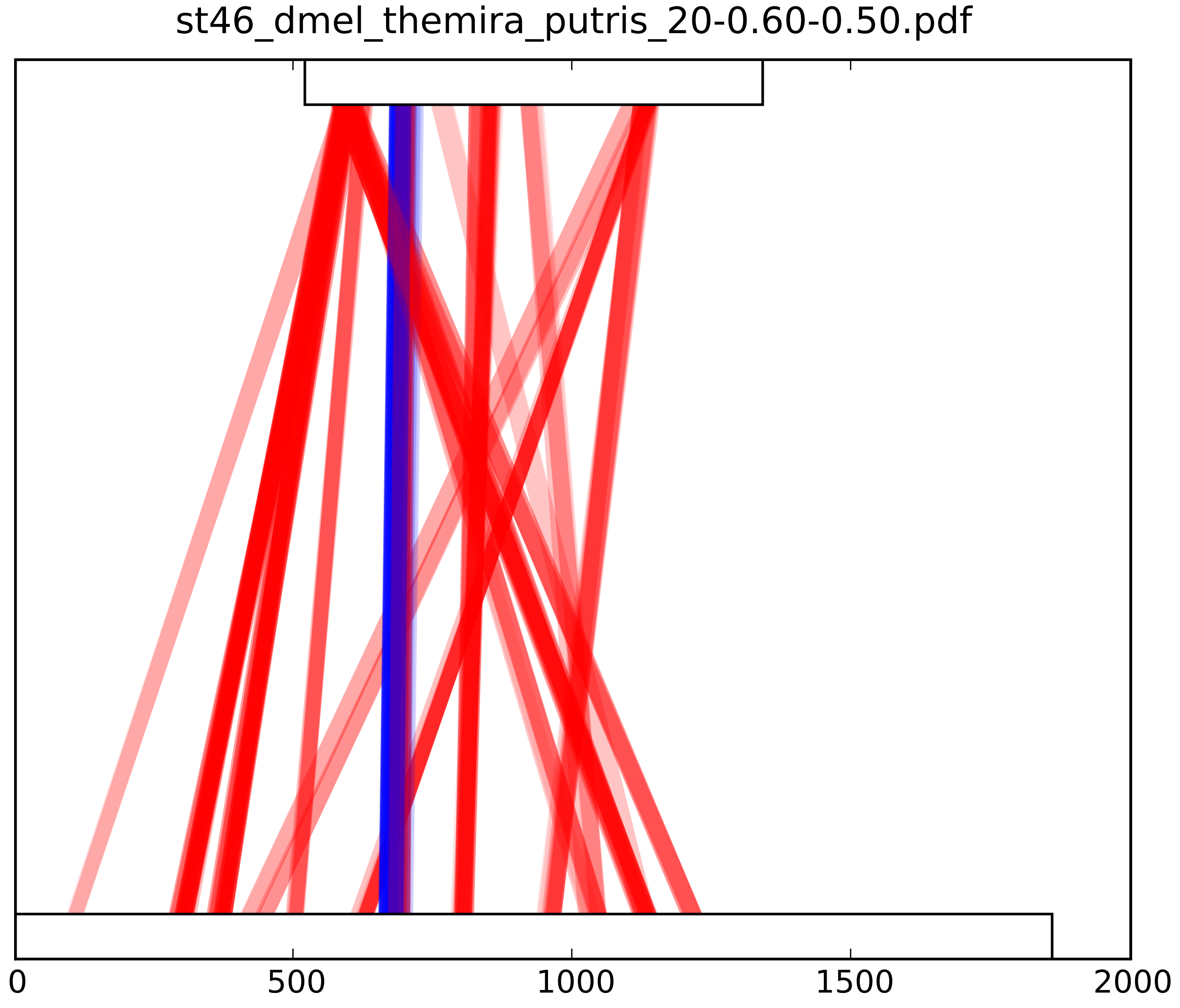
st46_dmel_themira_putris_20-0.60-0.50.pdf
0
500
1000
1500
2000

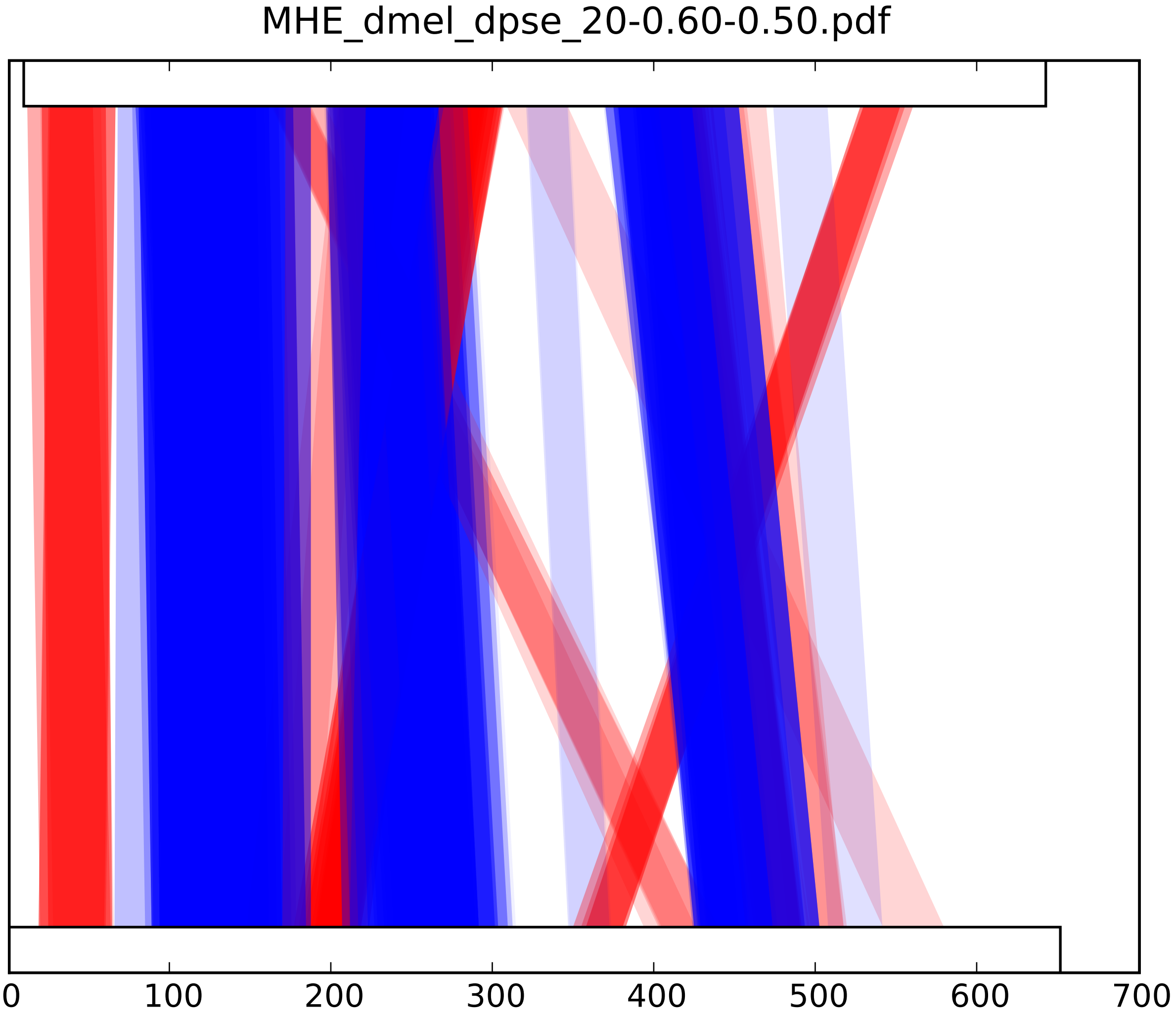
MHE_dmel_dpse_20-0.60-0.50.pdf
0
100
200
300
400
500
600
700

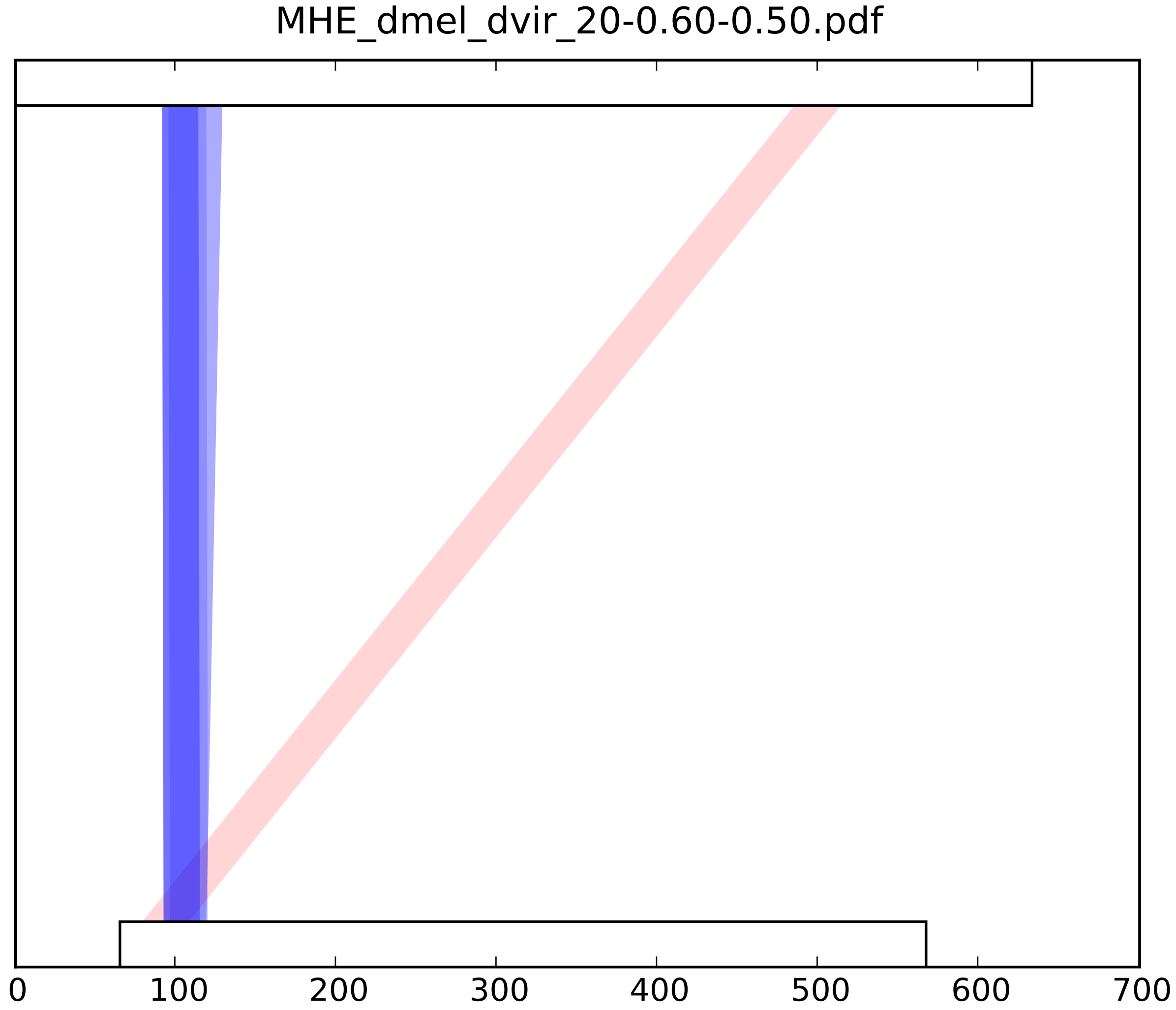
MHE_dmel_dvir_20-0.60-0.50.pdf
0
100
200
300
400
500
600
700

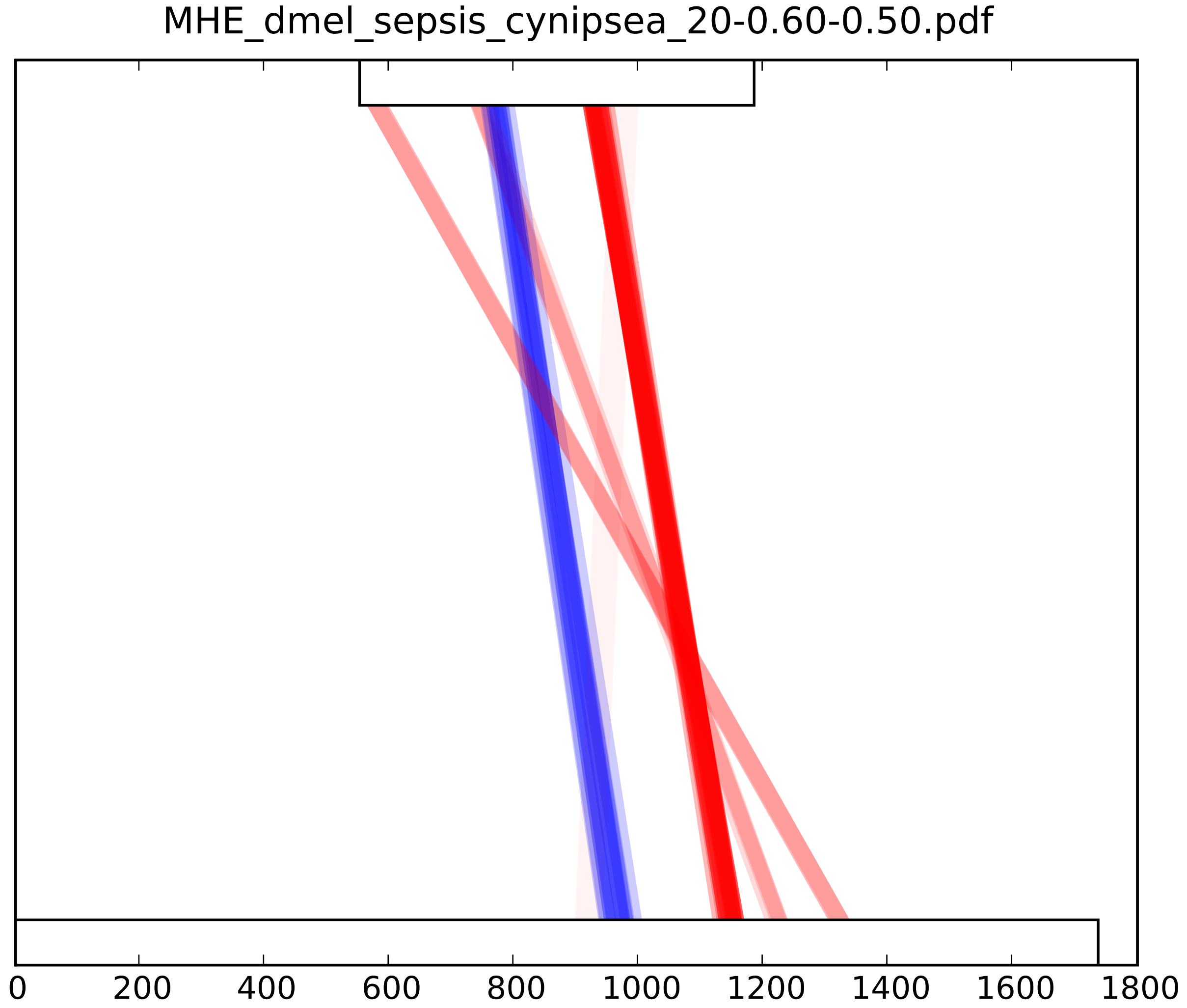

MHE_dmel_sepsis_cynipsea_20-0.60-0.50.pdf
0
200
400
600
800
1000
1200
1400
1600
1800

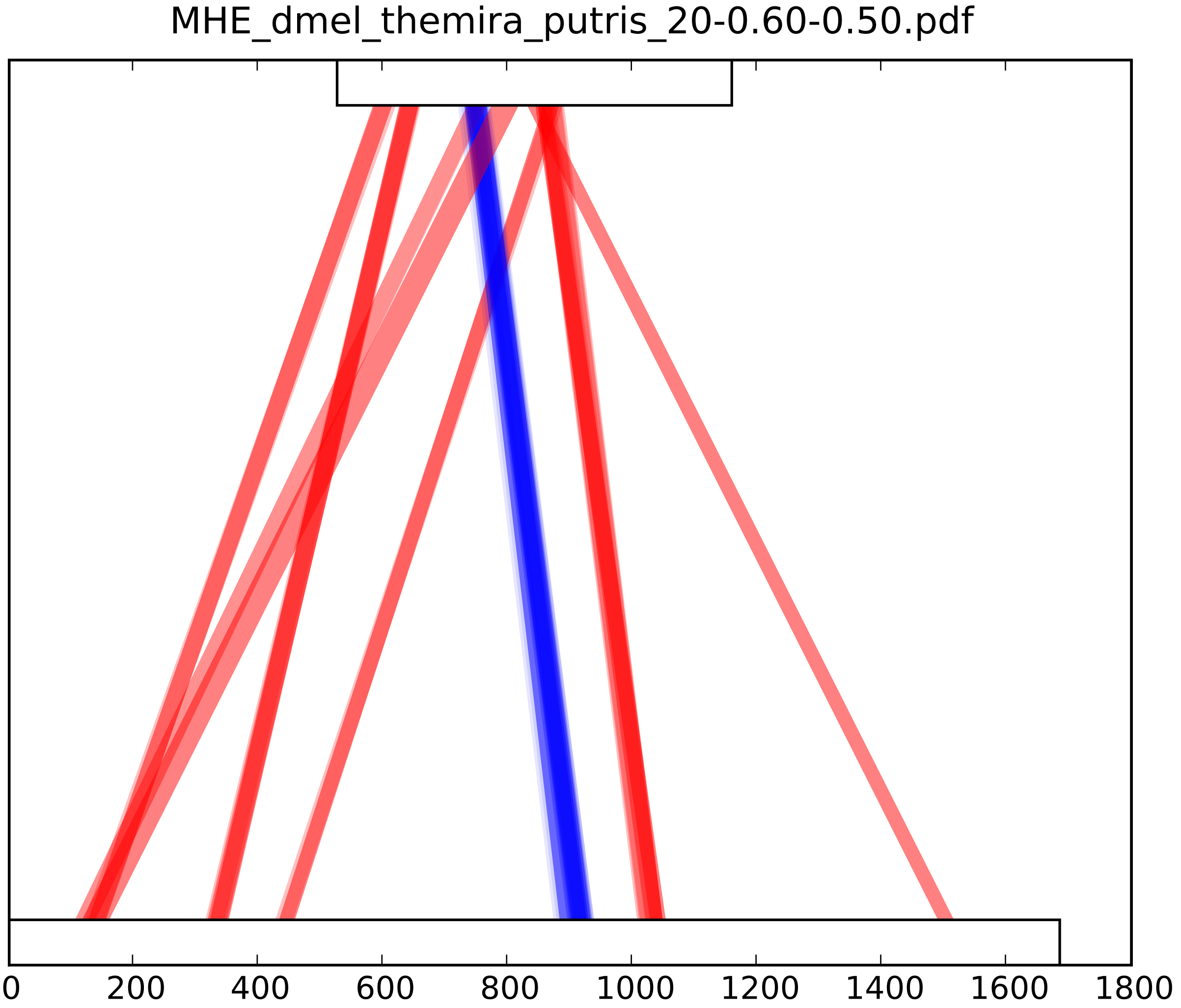
MHE_dmel_themira_putris_20-0.60-0.50.pdf
0
200
400
600
800
1000
1200
1400
1600
1800

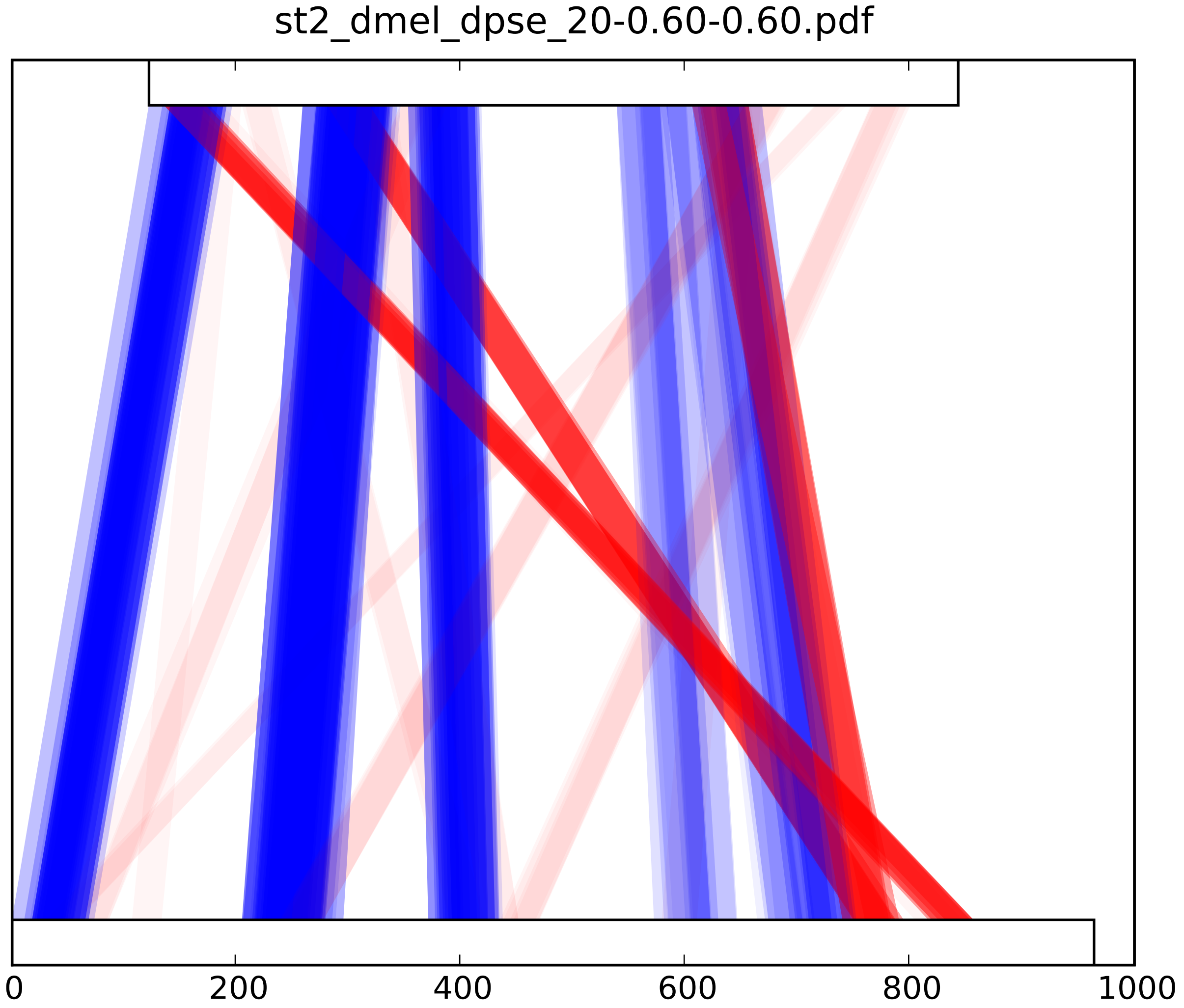
st2_dmel_dpse_20-0.60-0.60.pdf
0
200
400
600
800
1000

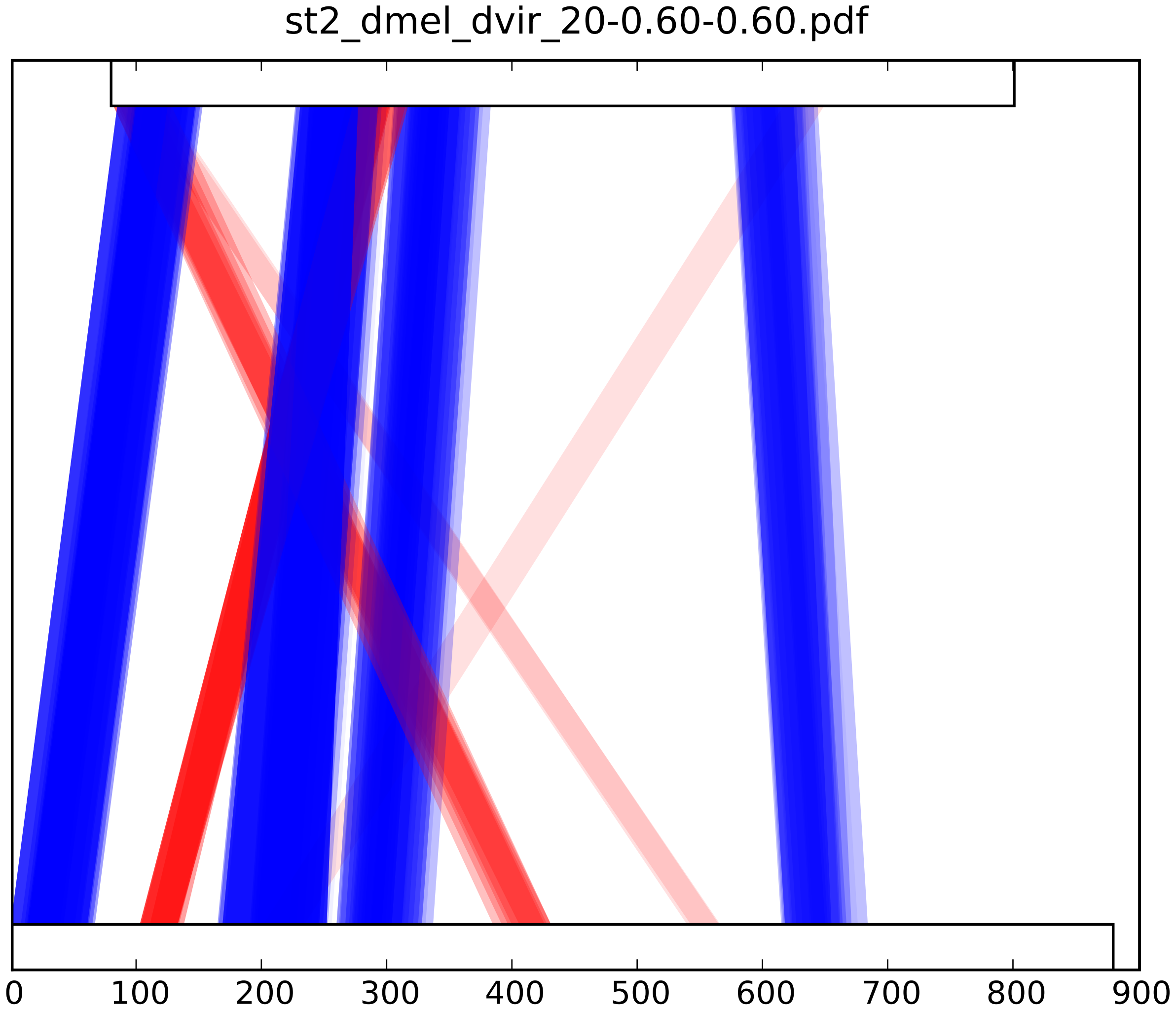
st2_dmel_dvir_20-0.60-0.60.pdf
0
100
200
300
400
500
600
700
800
900

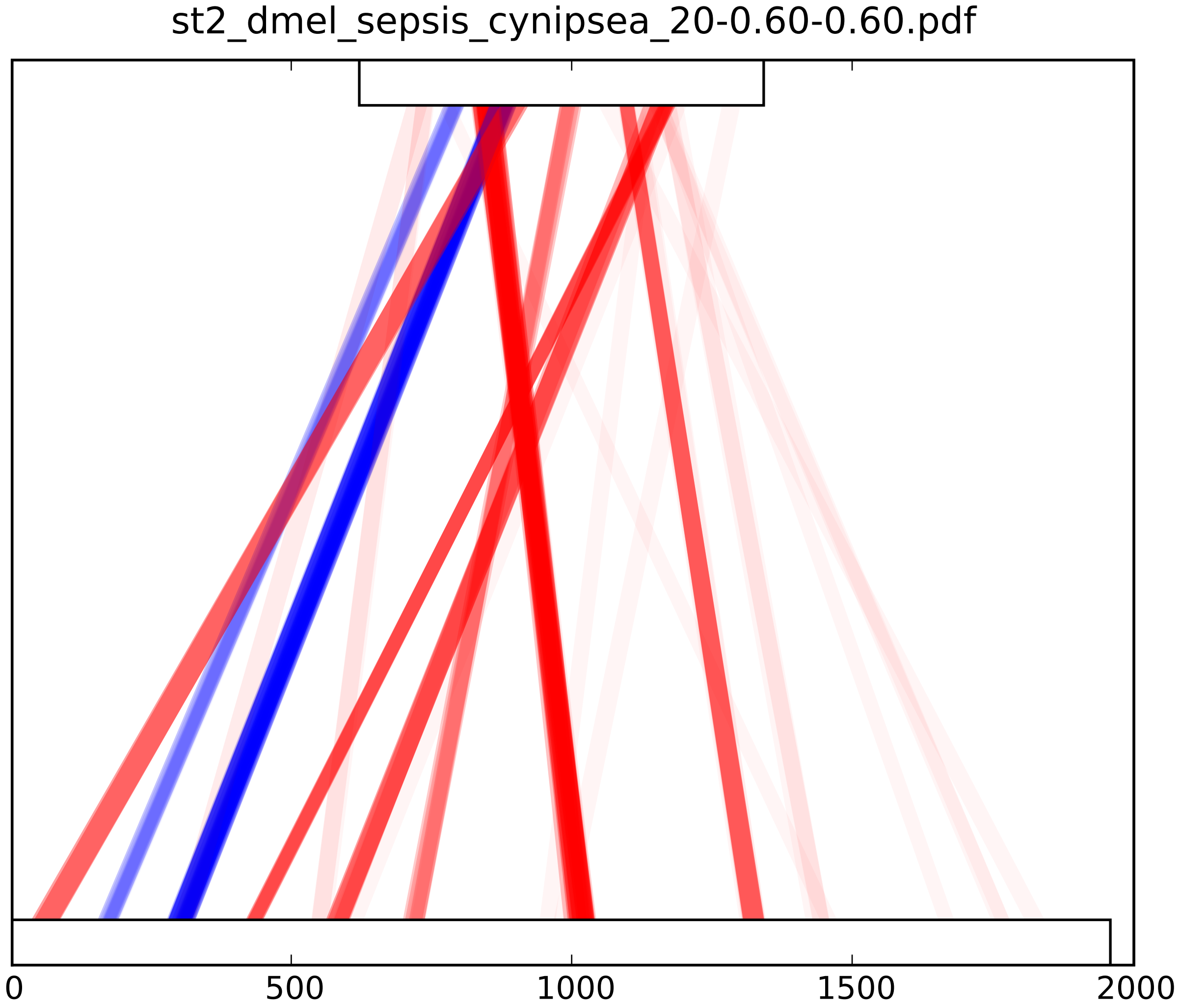
st2_dmel_sepsis_cynipsea_20-0.60-0.60.pdf
0
500
1000
1500
2000

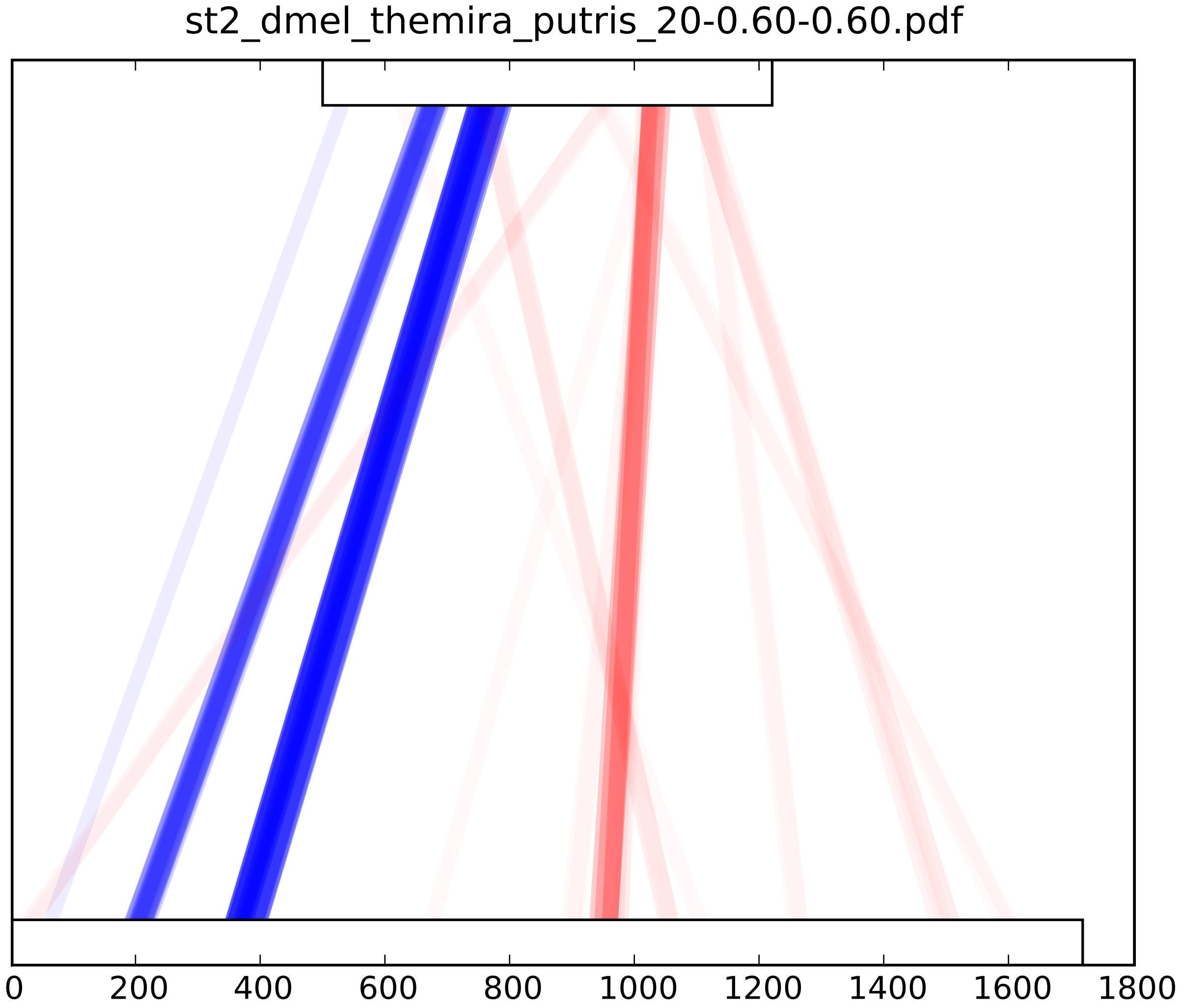

st2_dmel_themira_putris_20-0.60-0.60.pdf
0
200
400
600
800
1000
1200
1400
1600
1800

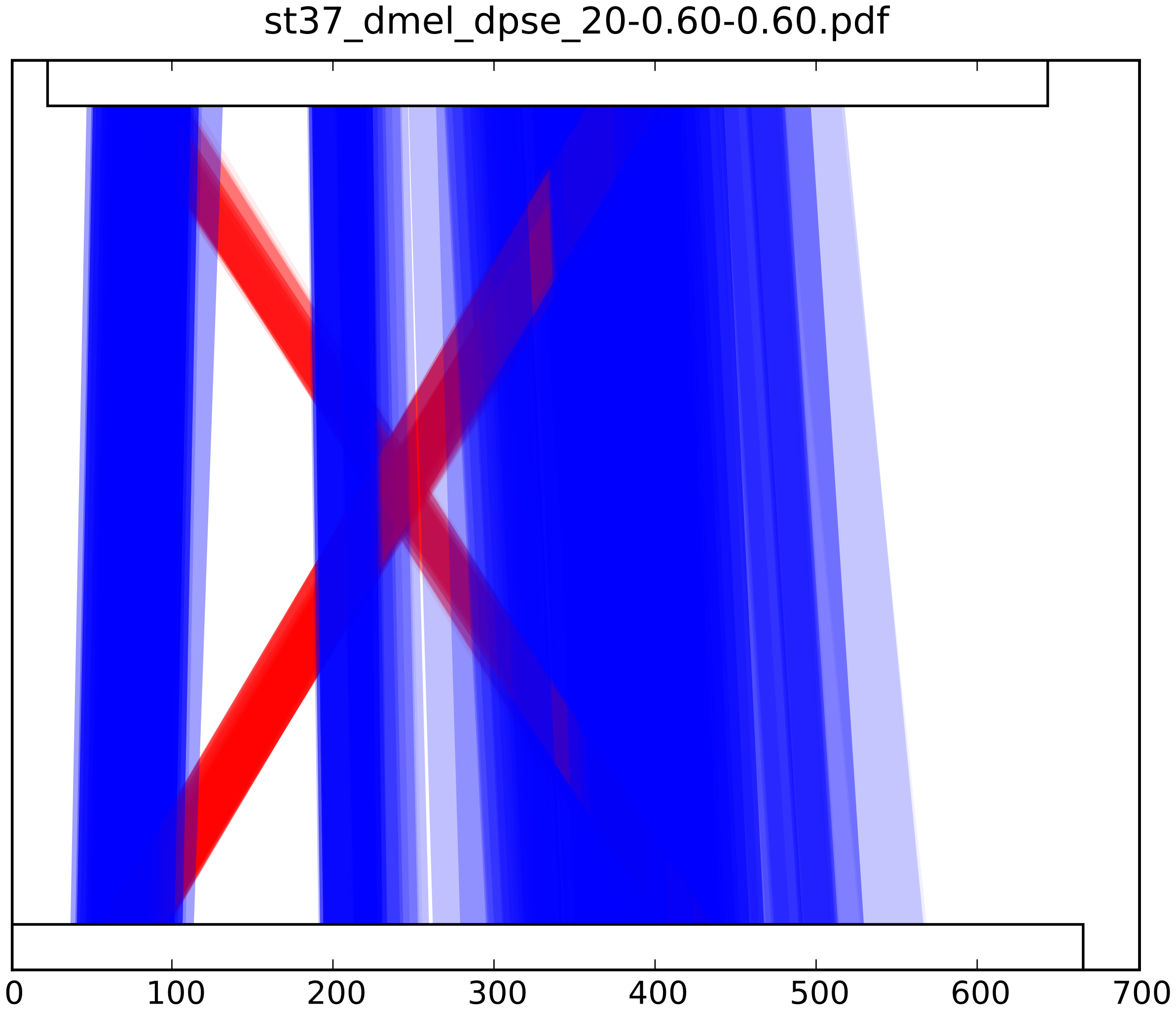

st37_dmel_dpse_20-0.60-0.60.pdf
0
100
200
300
400
500
600
700

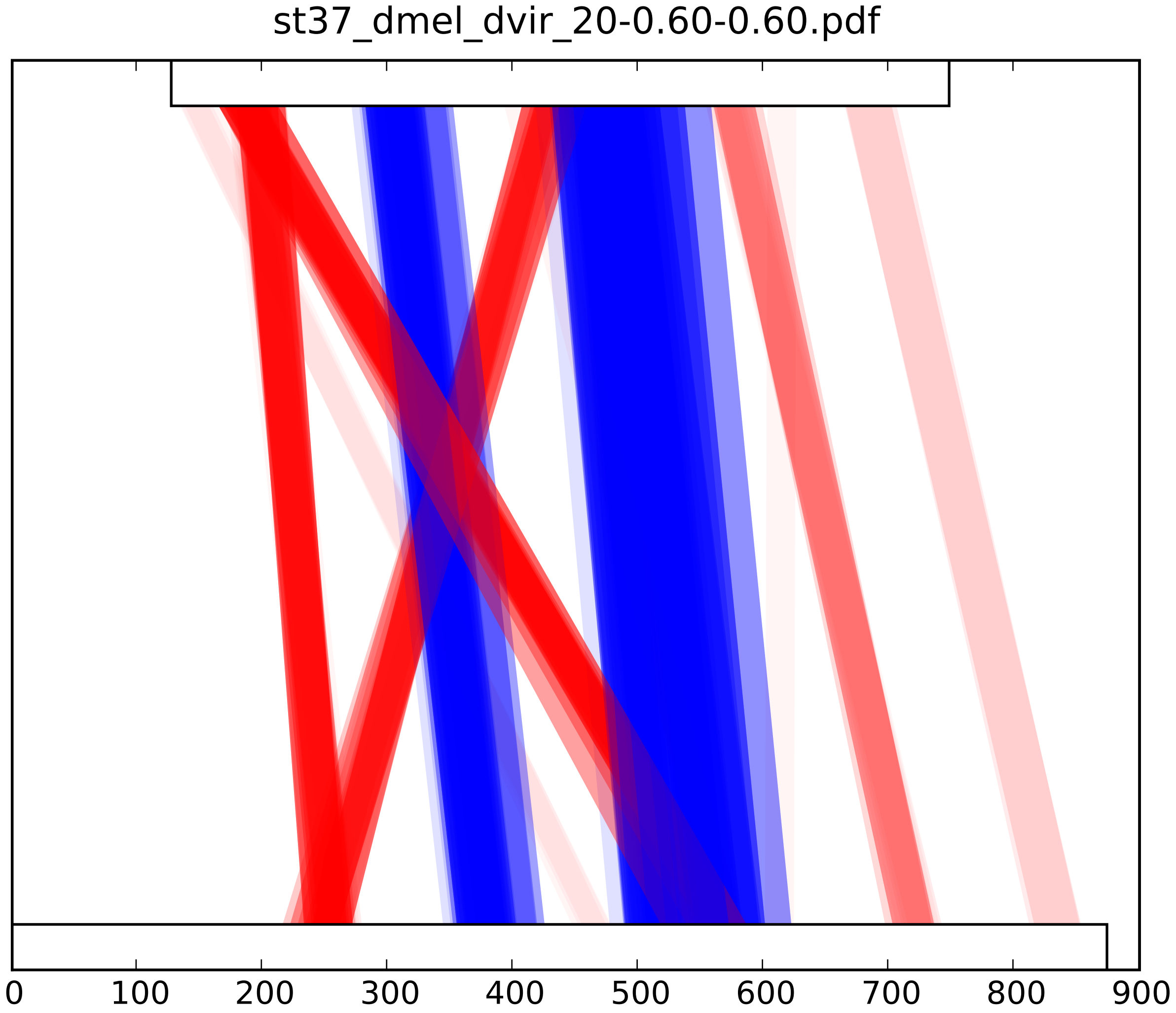

st37_dmel_dvir_20-0.60-0.60.pdf
0
100
200
300
400
500
600
700
800
900

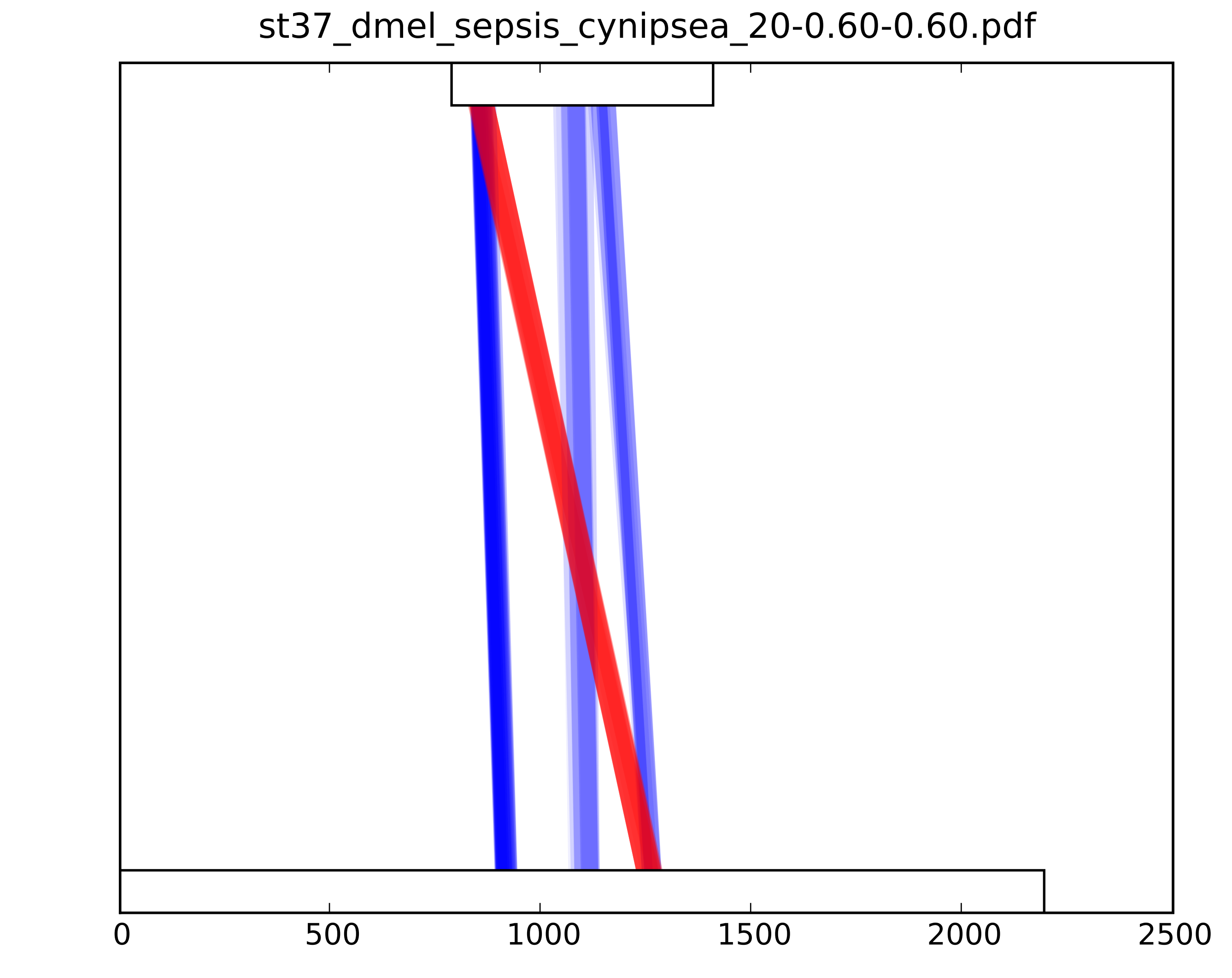
st37_dmel_sepsis_cynipsea_20-0.60-0.60.pdf
0
500
1000
1500
2000
2500

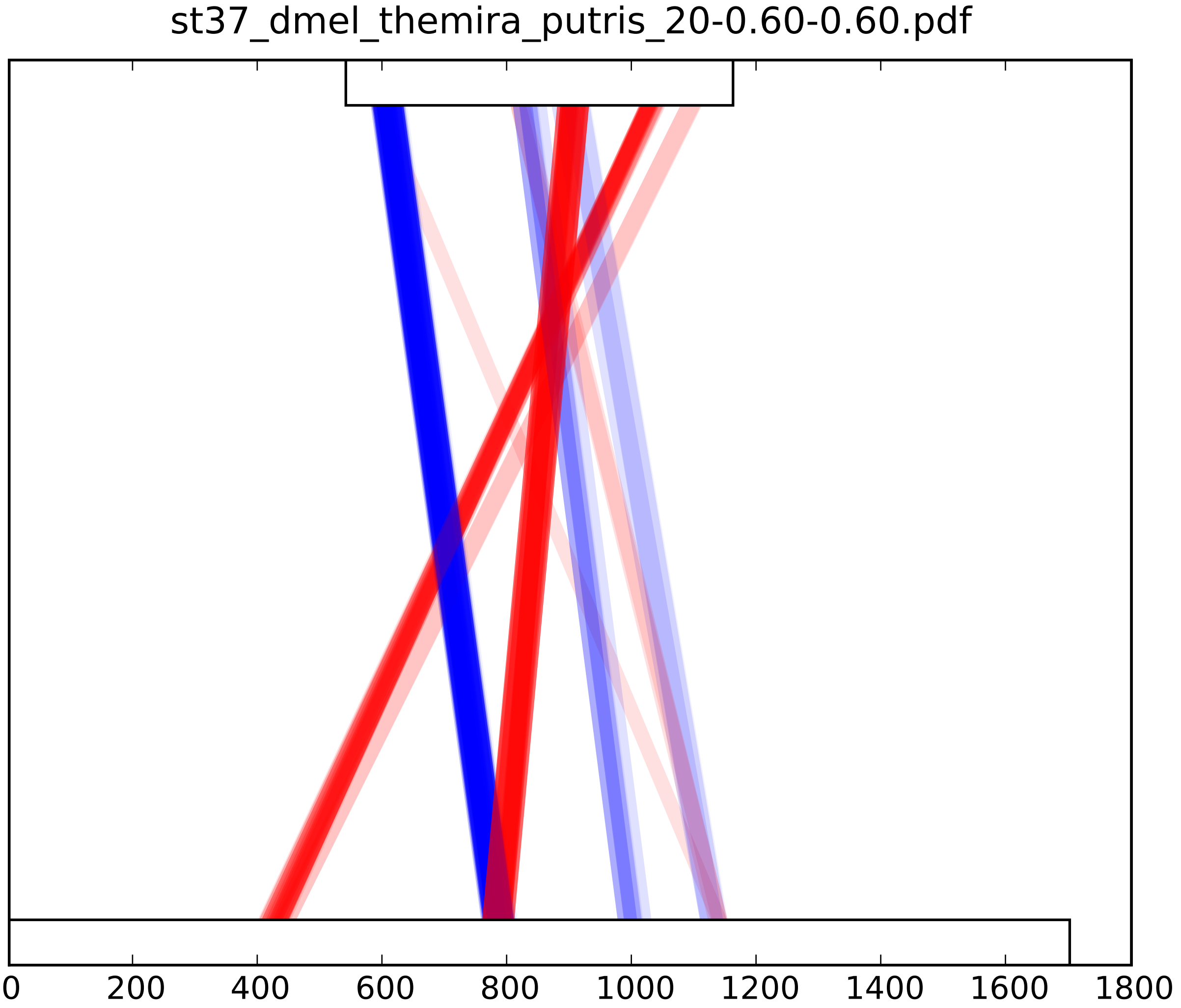
st37_dmel_themira_putris_20-0.60-0.60.pdf
0
200
400
600
800
1000
1200
1400
1600
1800

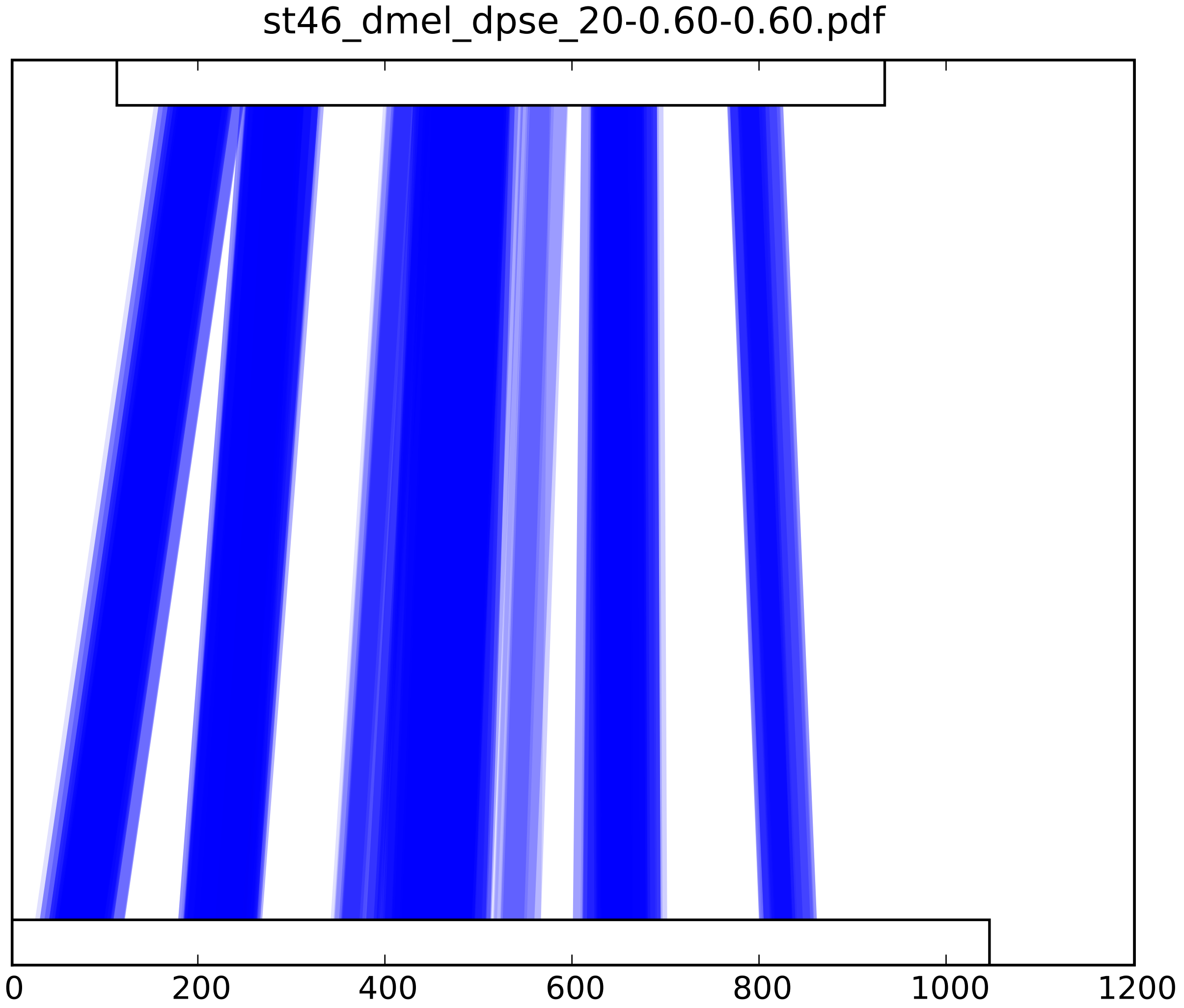

st46_dmel_dpse_20-0.60-0.60.pdf
0
200
400
600
800
1000
1200

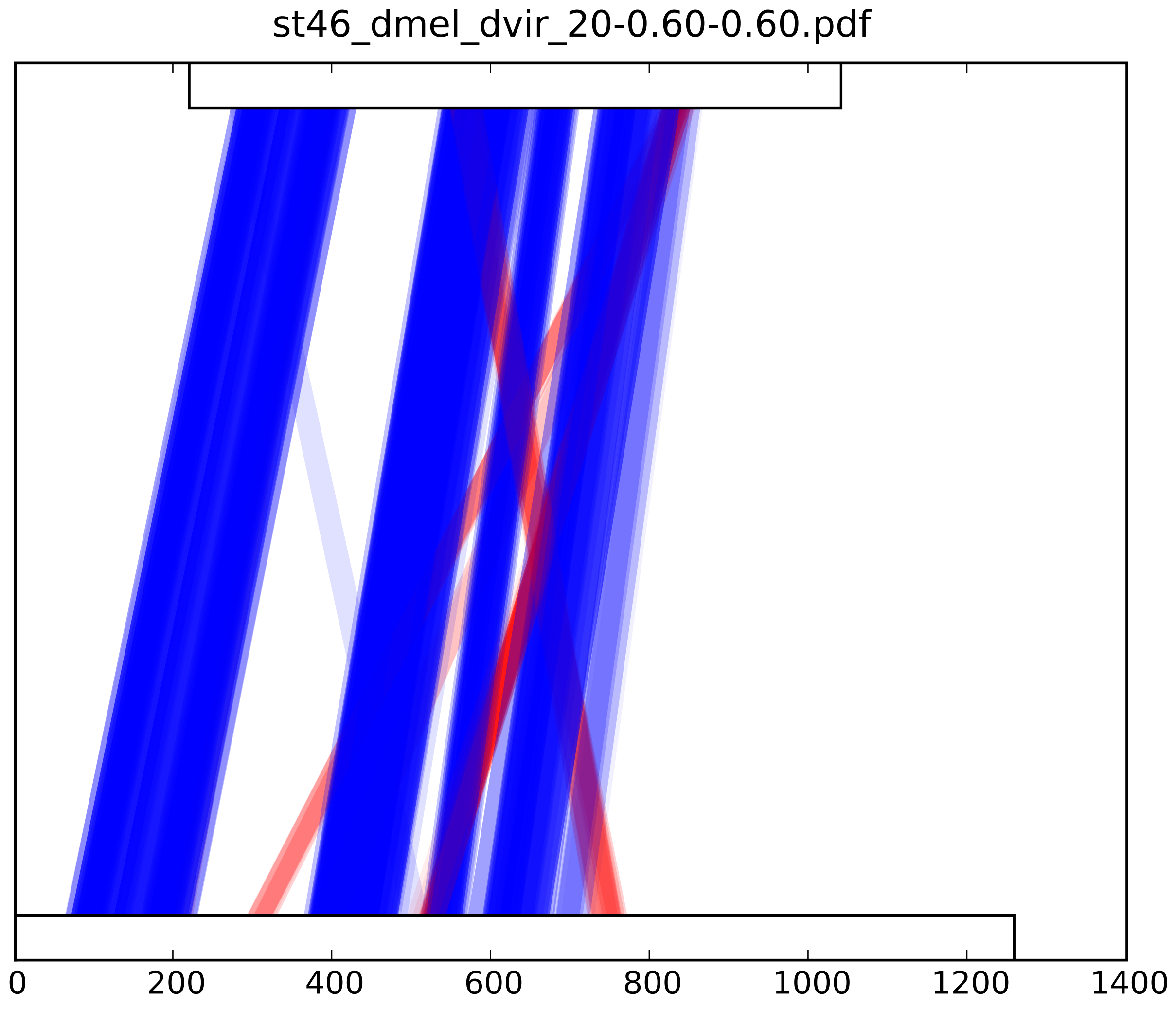
st46_dmel_dvir_20-0.60-0.60.pdf
0
200
400
600
800
1000
1200
1400

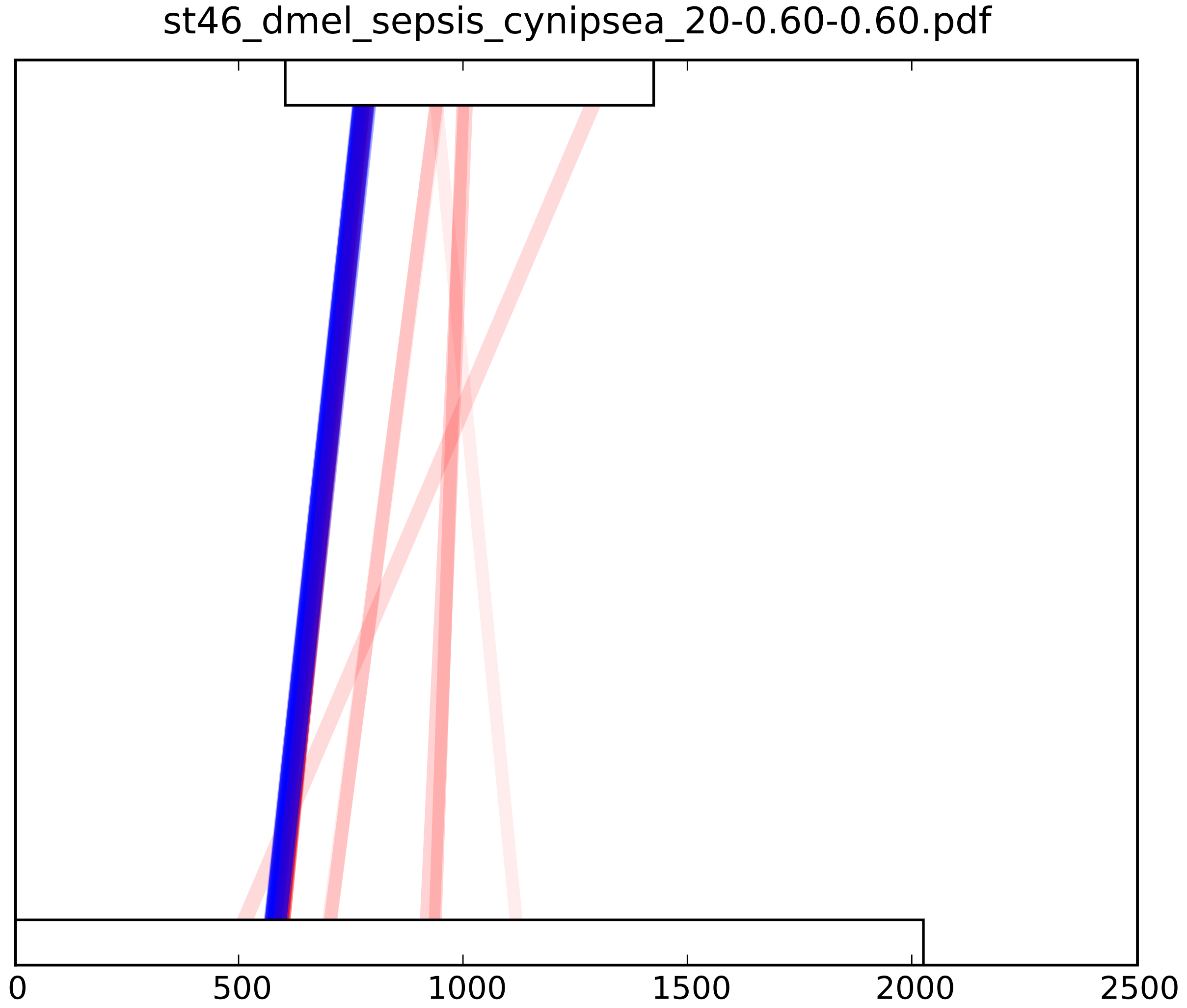
st46_dmel_sepsis_cynipsea_20-0.60-0.60.pdf
0
500
1000
1500
2000
2500

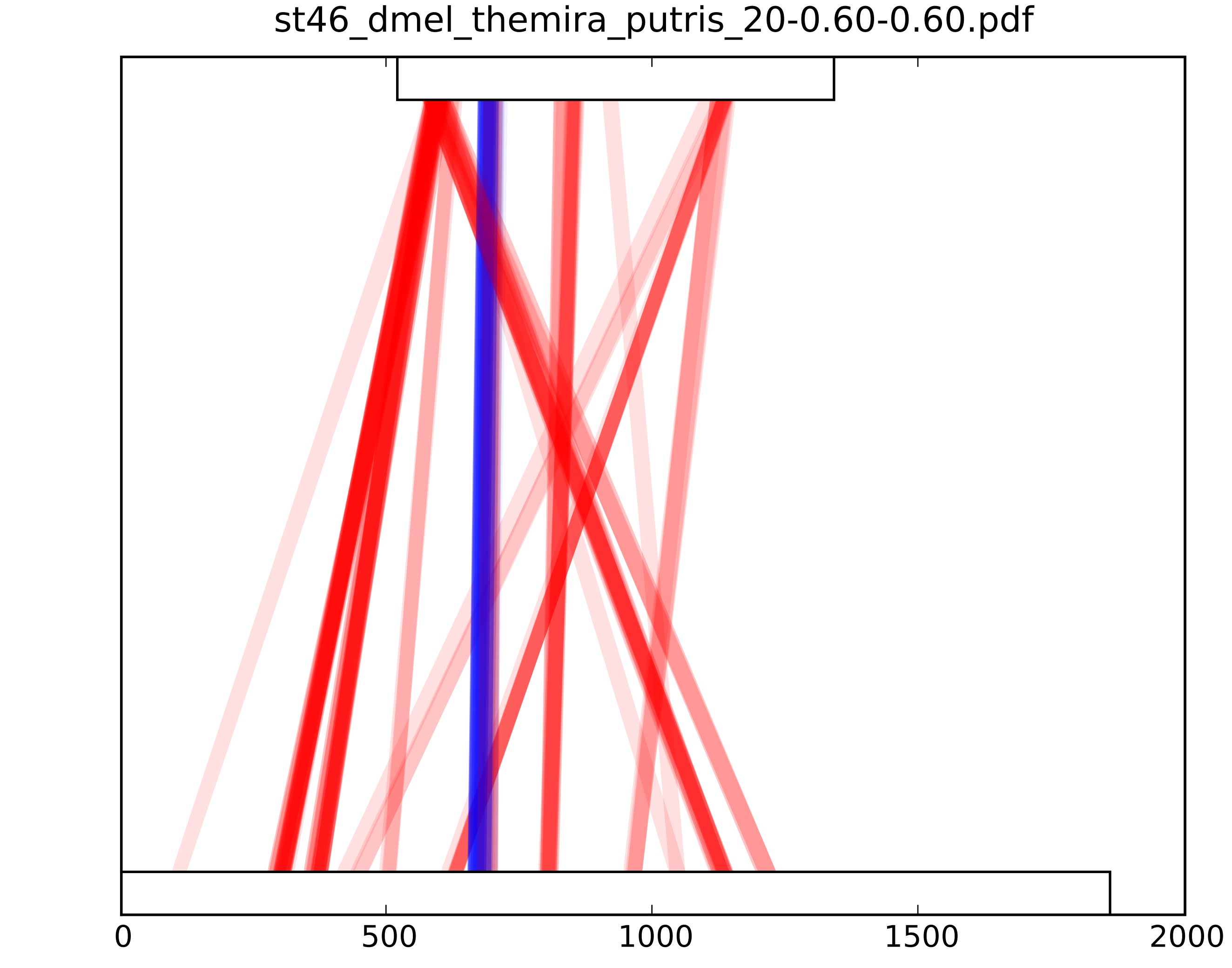
st46_dmel_themira_putris_20-0.60-0.60.pdf
0
500
1000
1500
2000

MHE_dmel_dpse_20-0.60-0.60.pdf

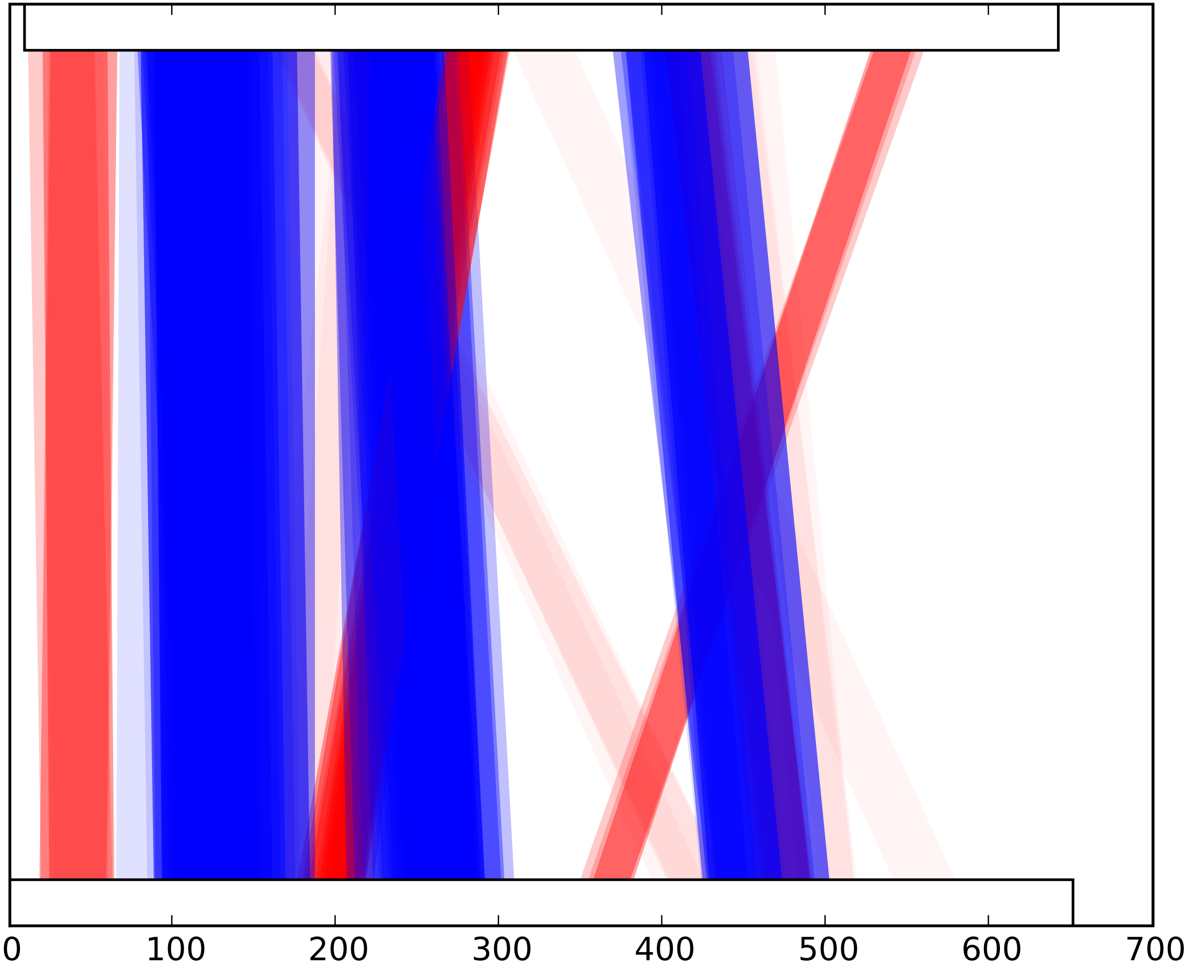

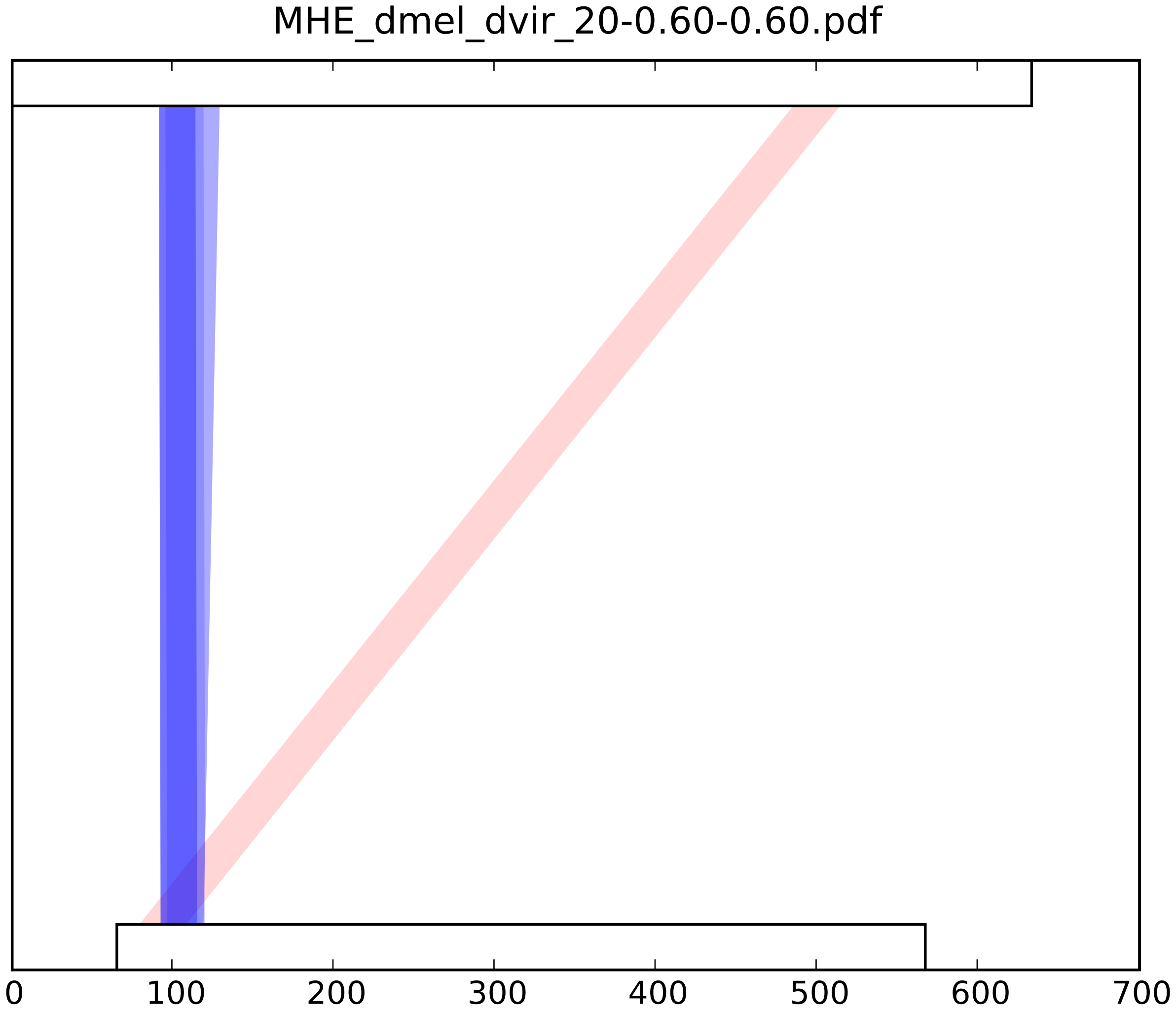
MHE_dmel_dvir_20-0.60-0.60.pdf
0
100
200
300
400
500
600
700

MHE_dmel_sepsis_cynipsea_20-0.60-0.60.pdf

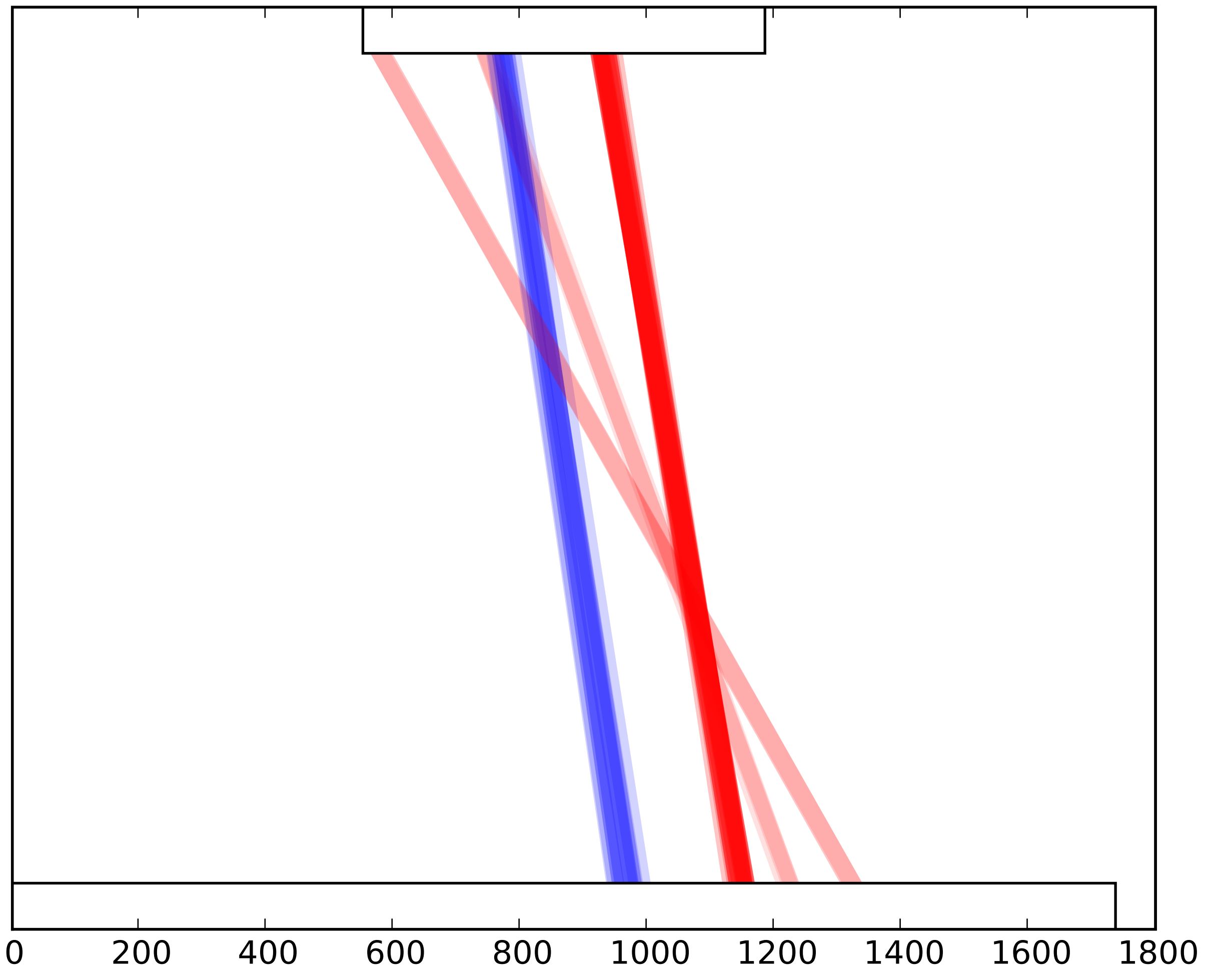

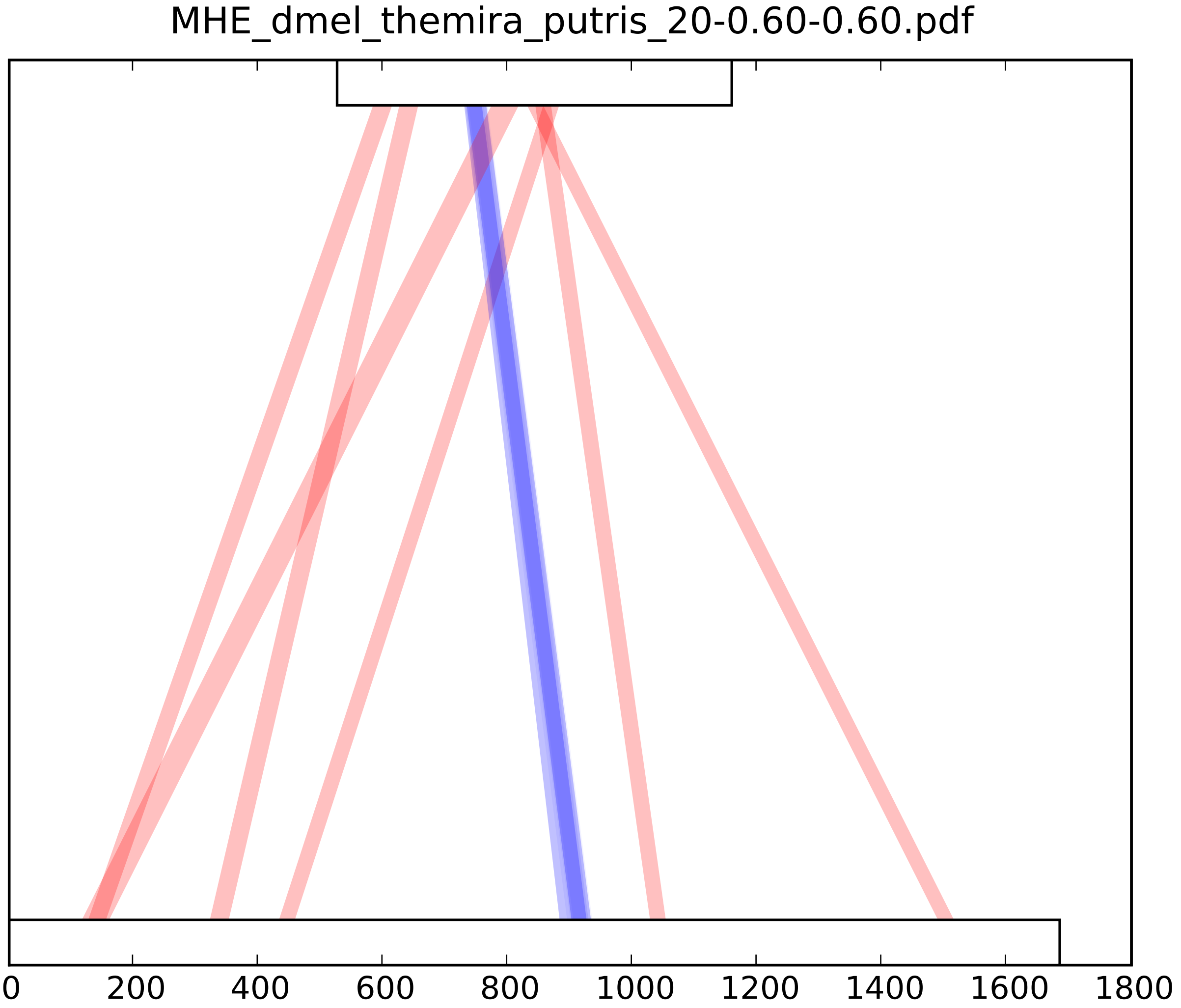
MHE_dmel_themira_putris_20-0.60-0.60.pdf
0
200
400
600
800
1000
1200
1400
1600
1800

st2_dmel_dpse_20-0.60-0.70.pdf

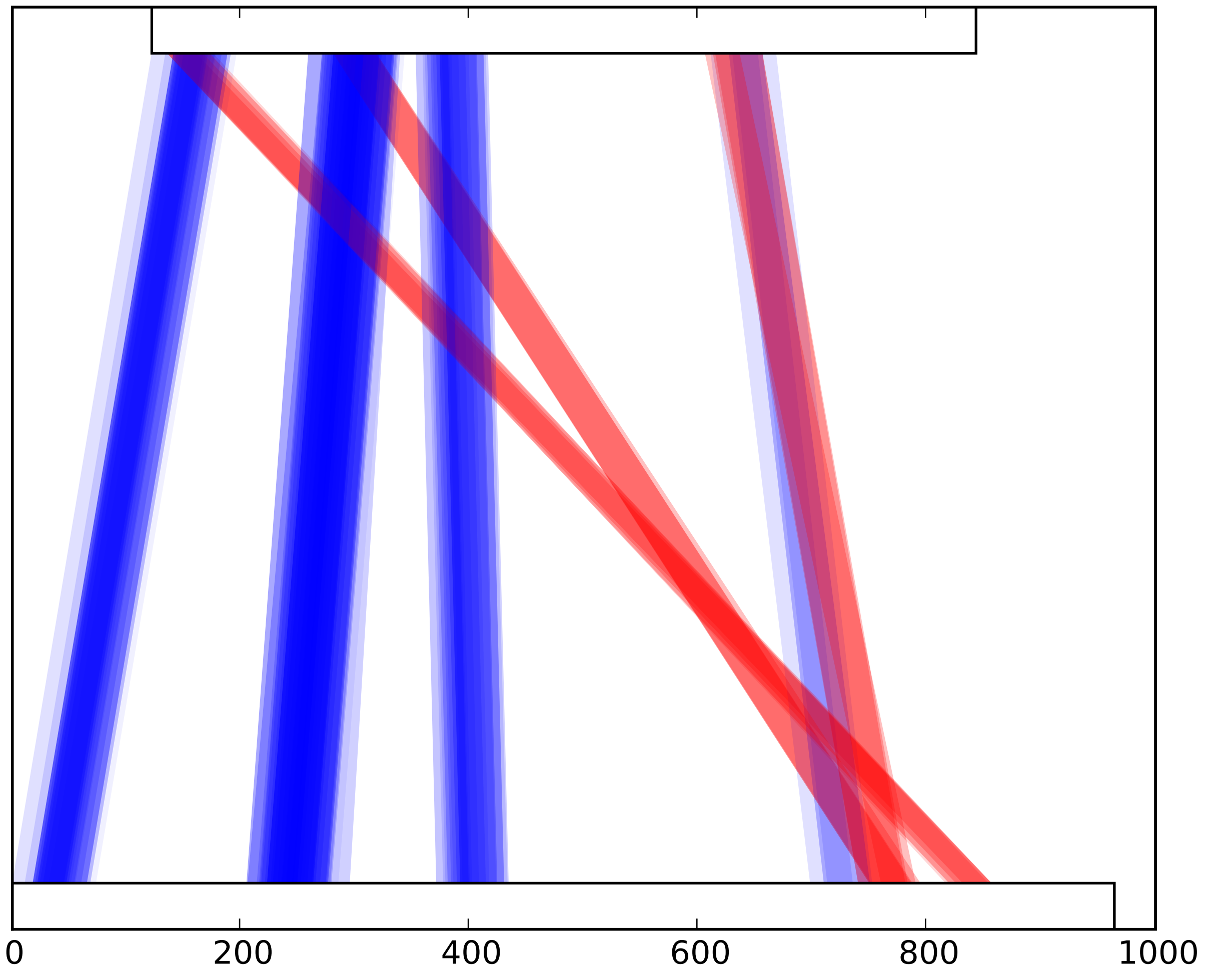

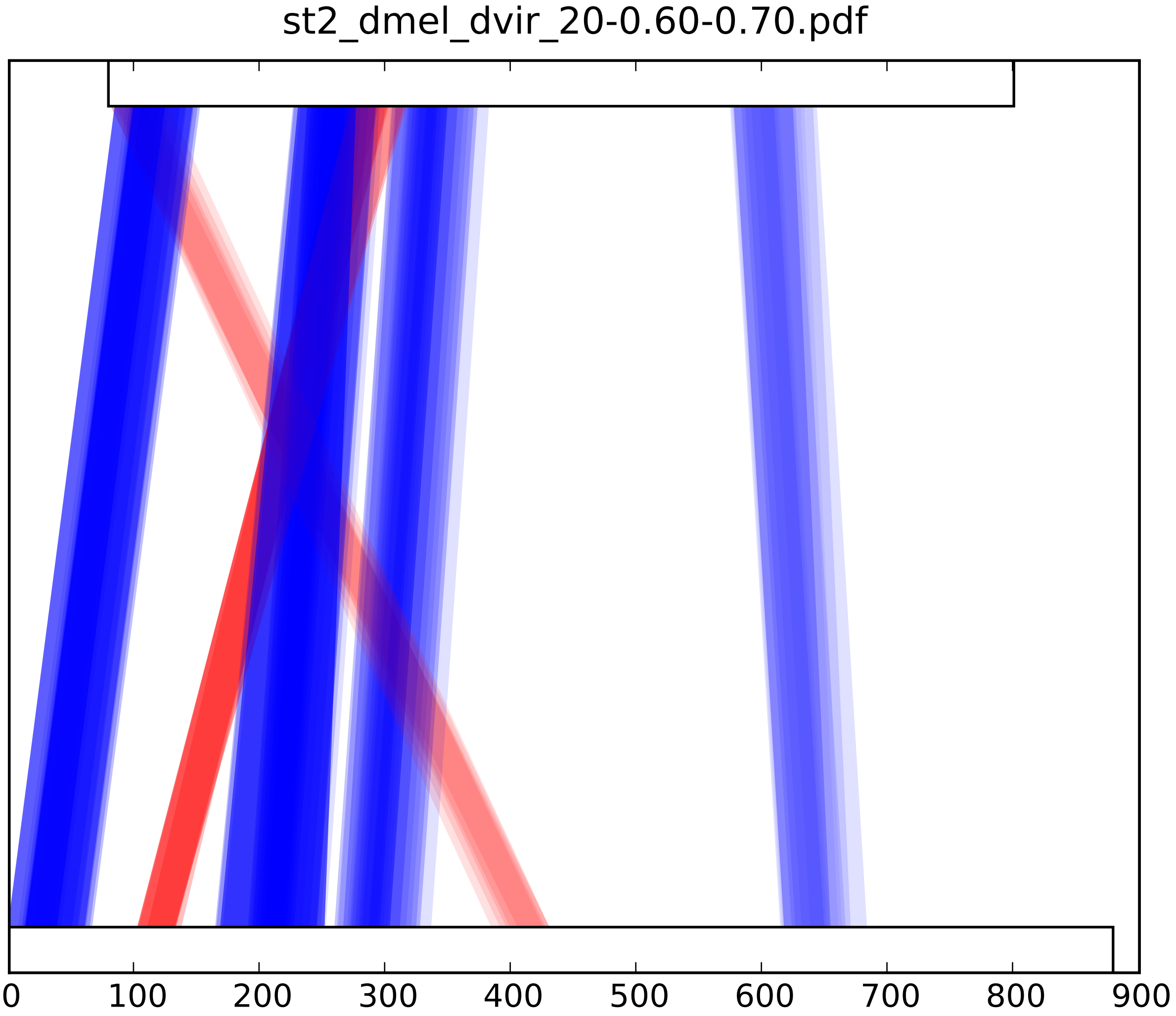
st2_dmel_dvir_20-0.60-0.70.pdf
0
100
200
300
400
500
600
700
800
900

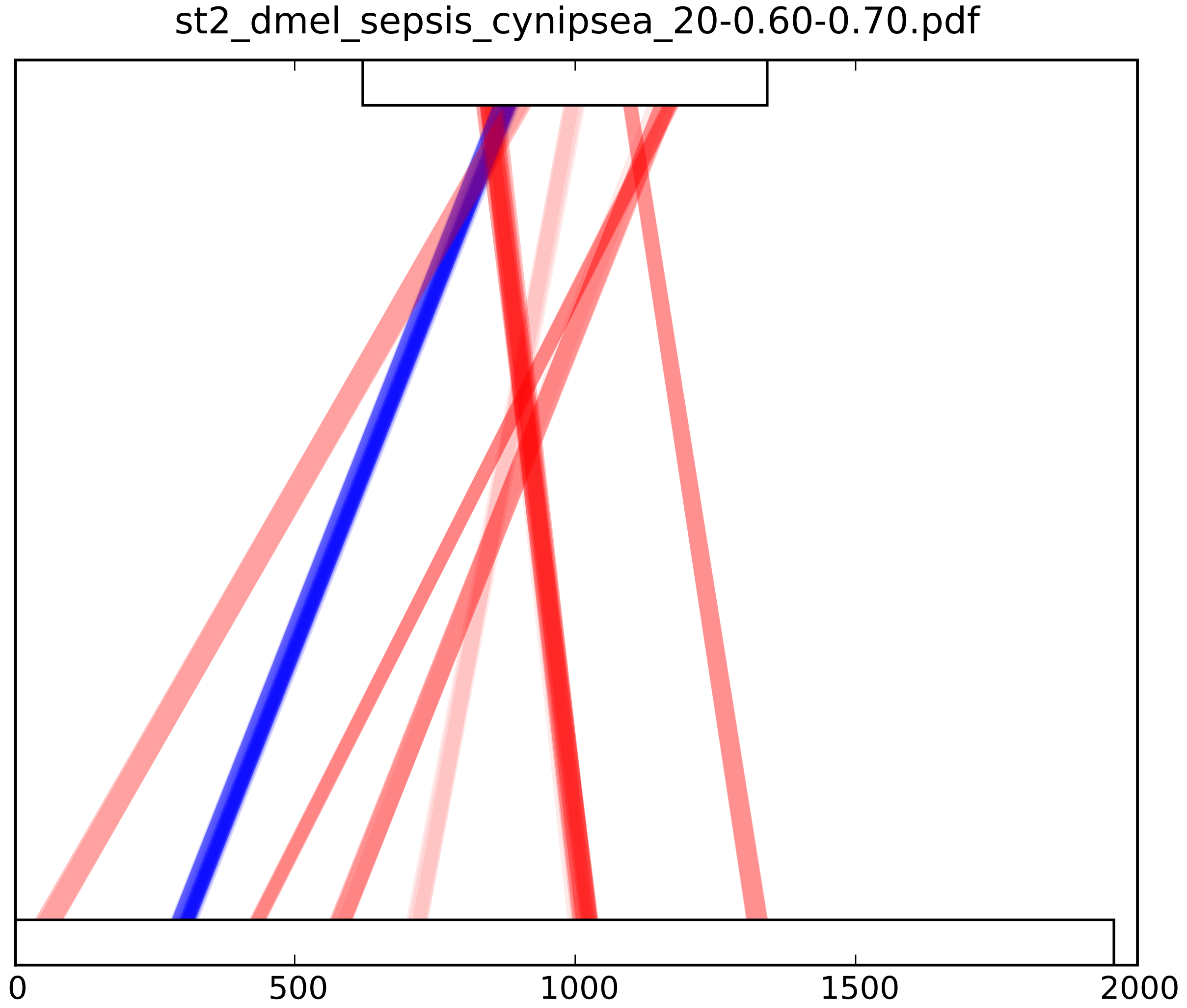
st2_dmel_sepsis_cynipsea_20-0.60-0.70.pdf
0
500
1000
1500
2000

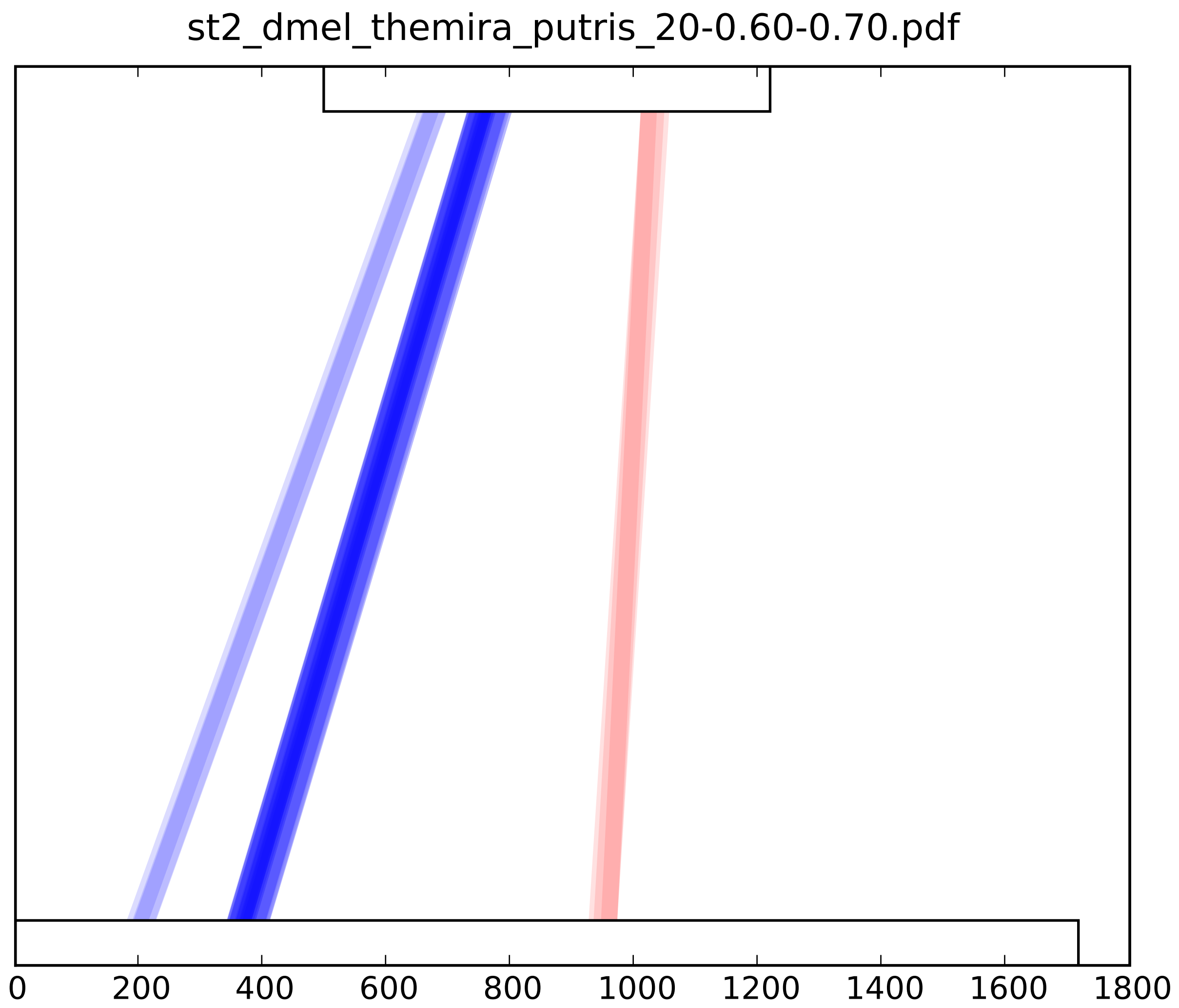
st2_dmel_themira_putris_20-0.60-0.70.pdf
0
200
400
600
800
1000
1200
1400
1600
1800

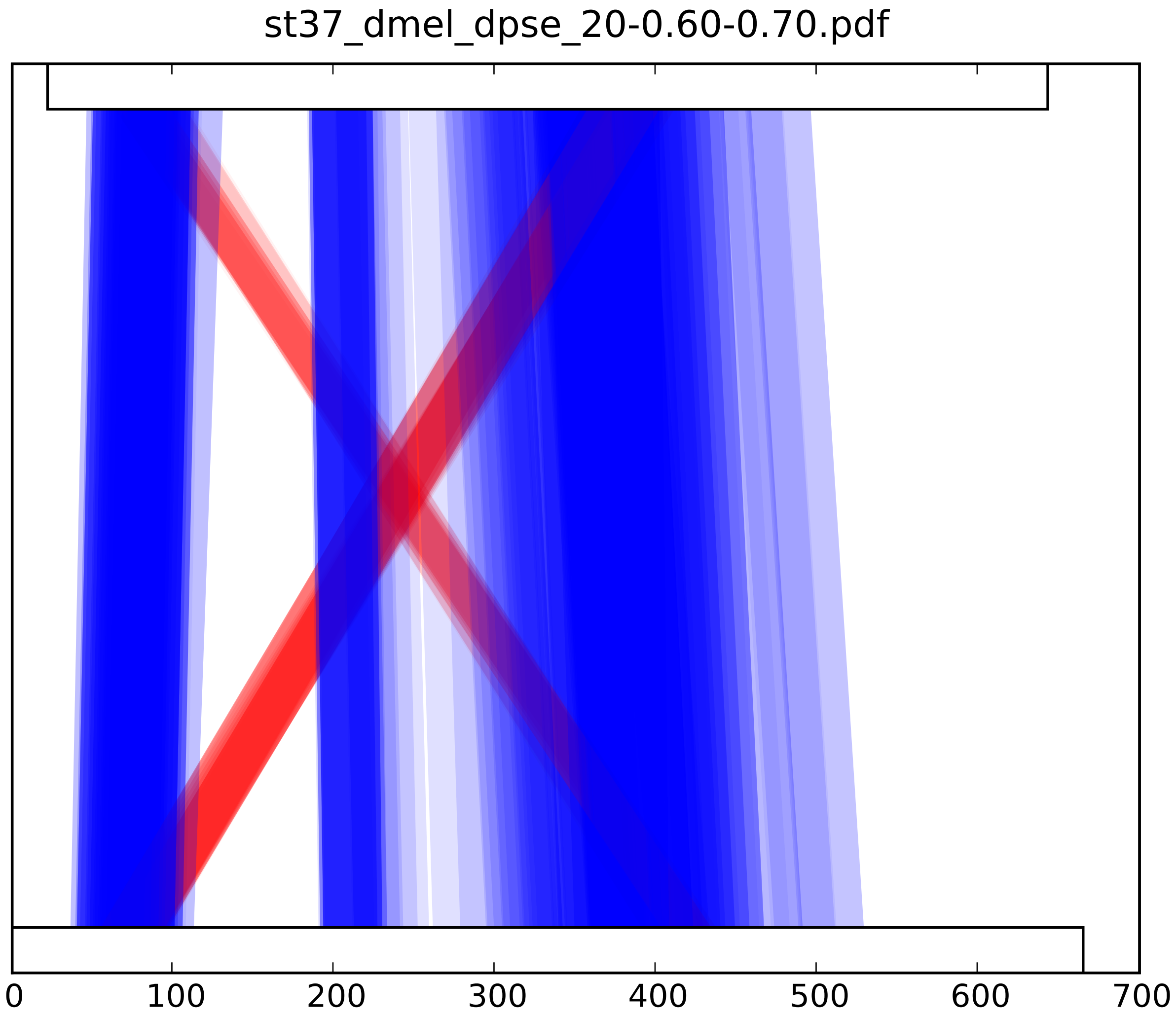
st37_dmel_dpse_20-0.60-0.70.pdf
0
100
200
300
400
500
600
700

st37_dmel_dvir_20-0.60-0.70.pdf

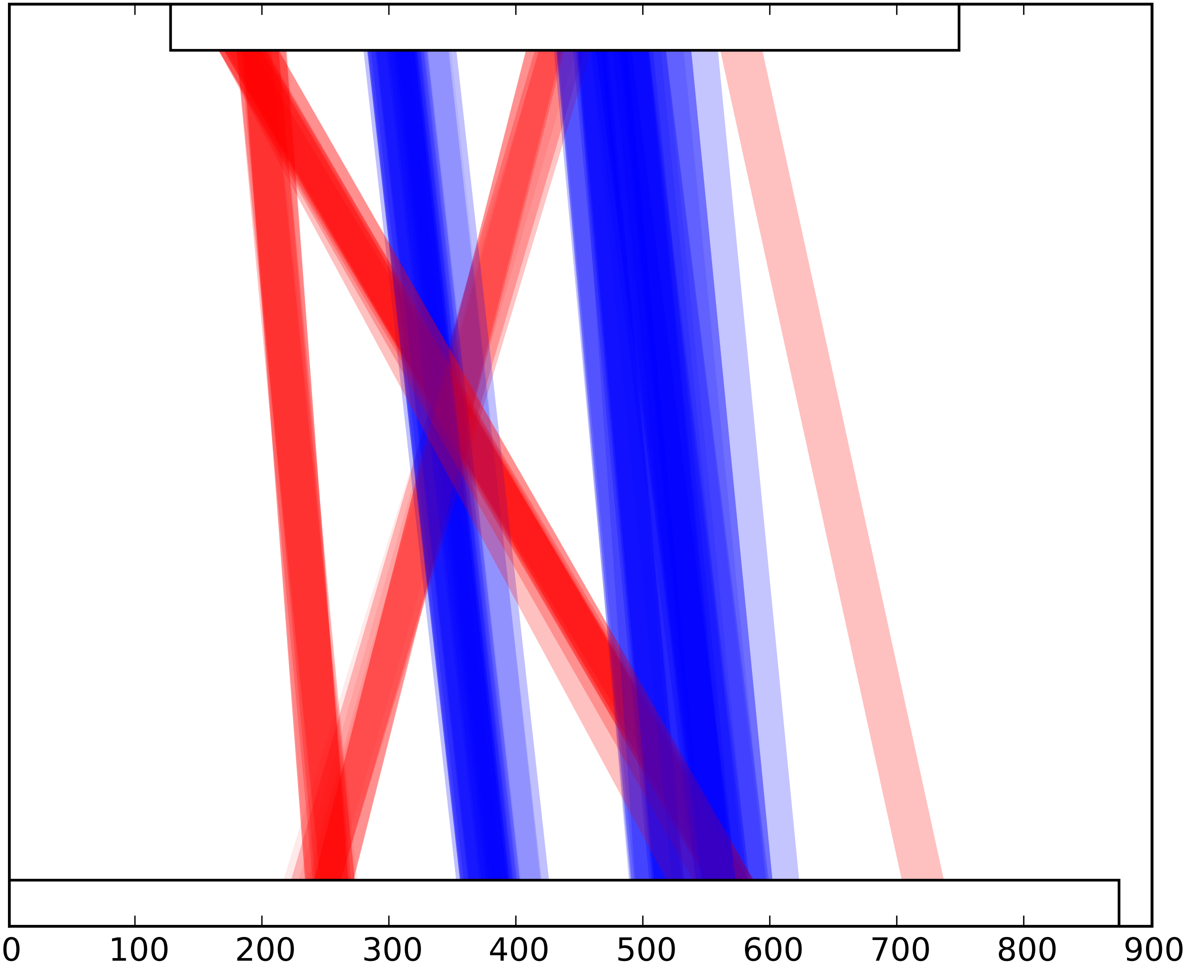

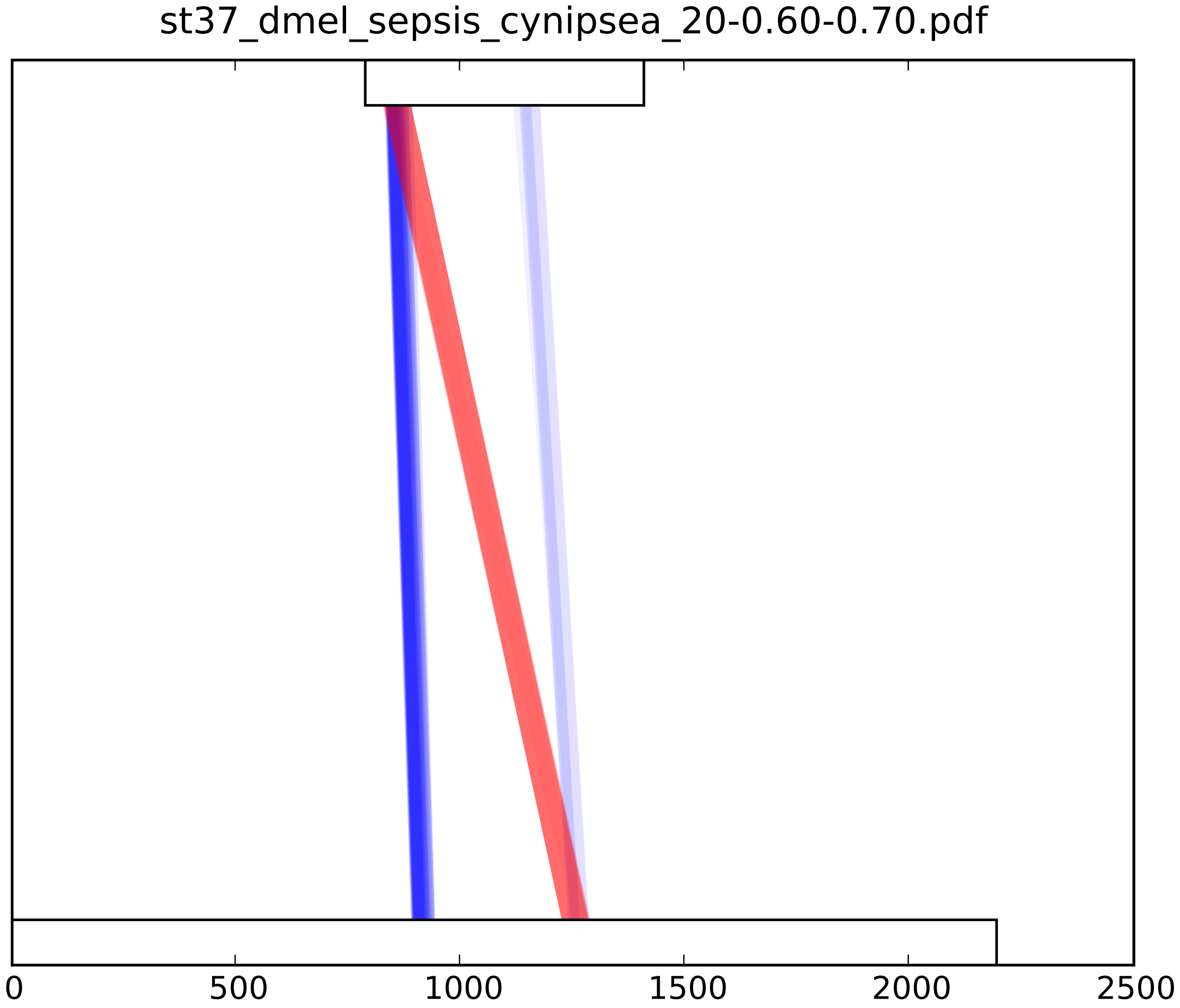

st37_dmel_sepsis_cynipsea_20-0.60-0.70.pdf
0
500
1000
1500
2000
2500

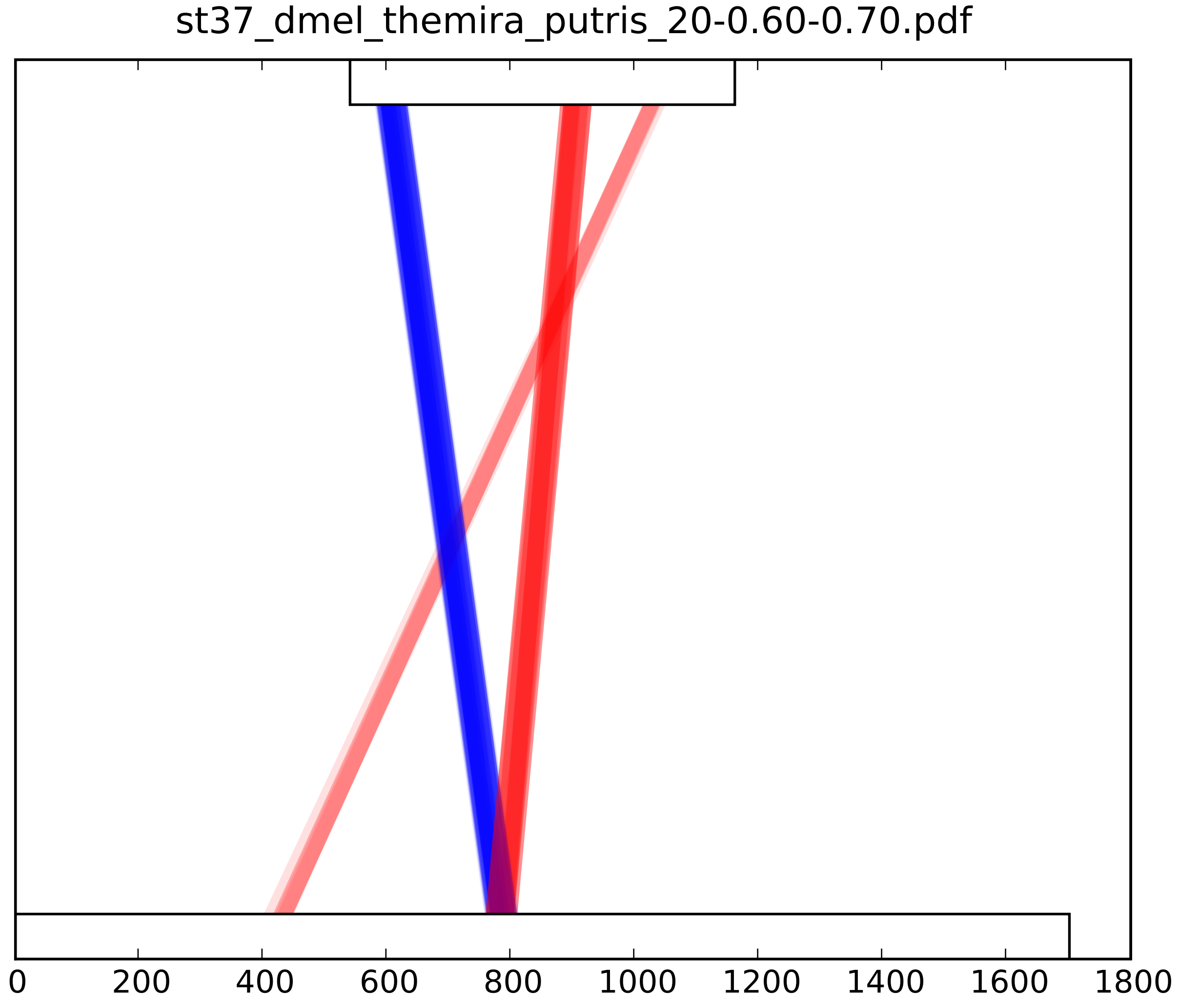
st37_dmel_themira_putris_20-0.60-0.70.pdf
0
200
400
600
800
1000
1200
1400
1600
1800

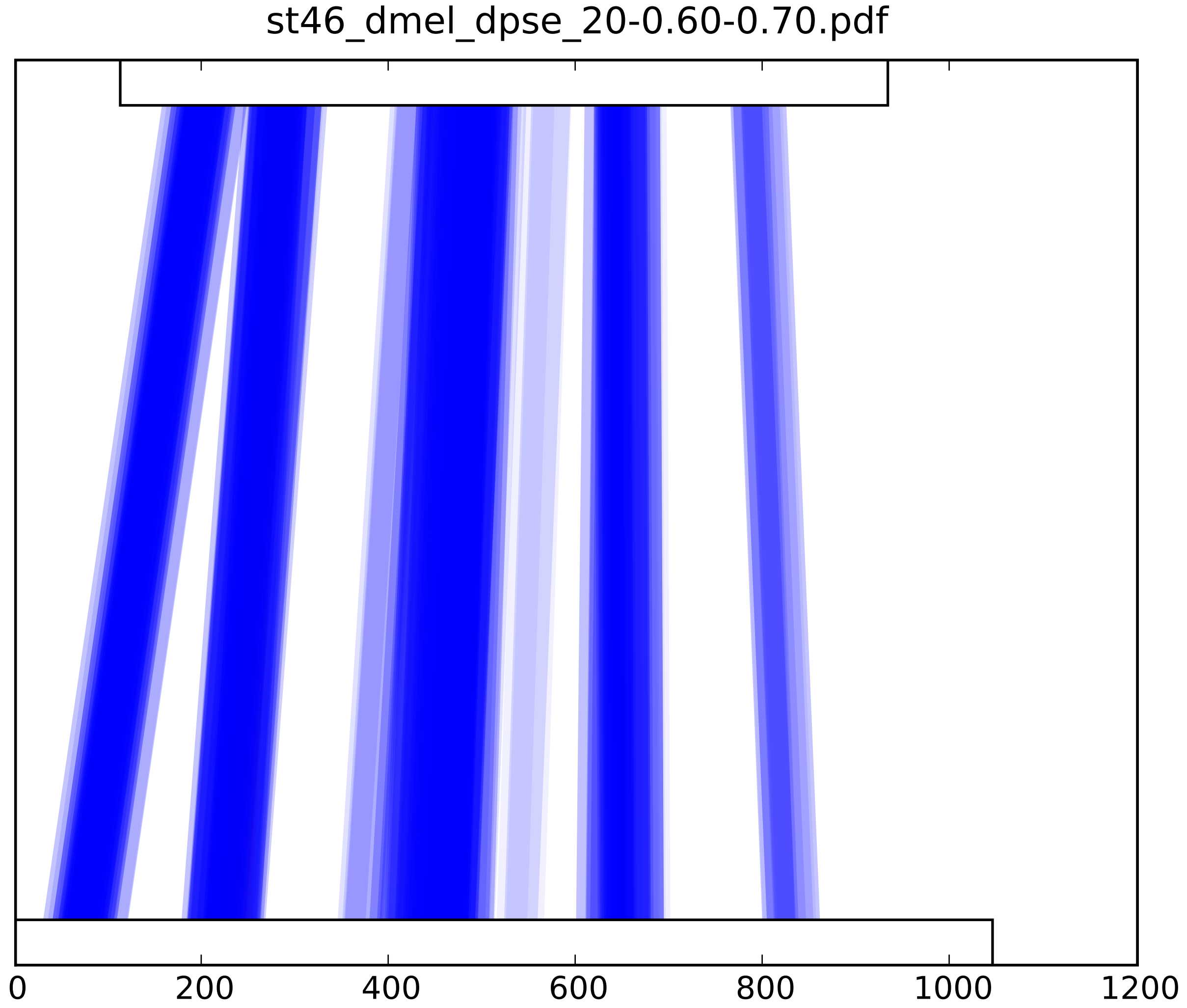
st46_dmel_dpse_20-0.60-0.70.pdf
0
200
400
600
800
1000
1200

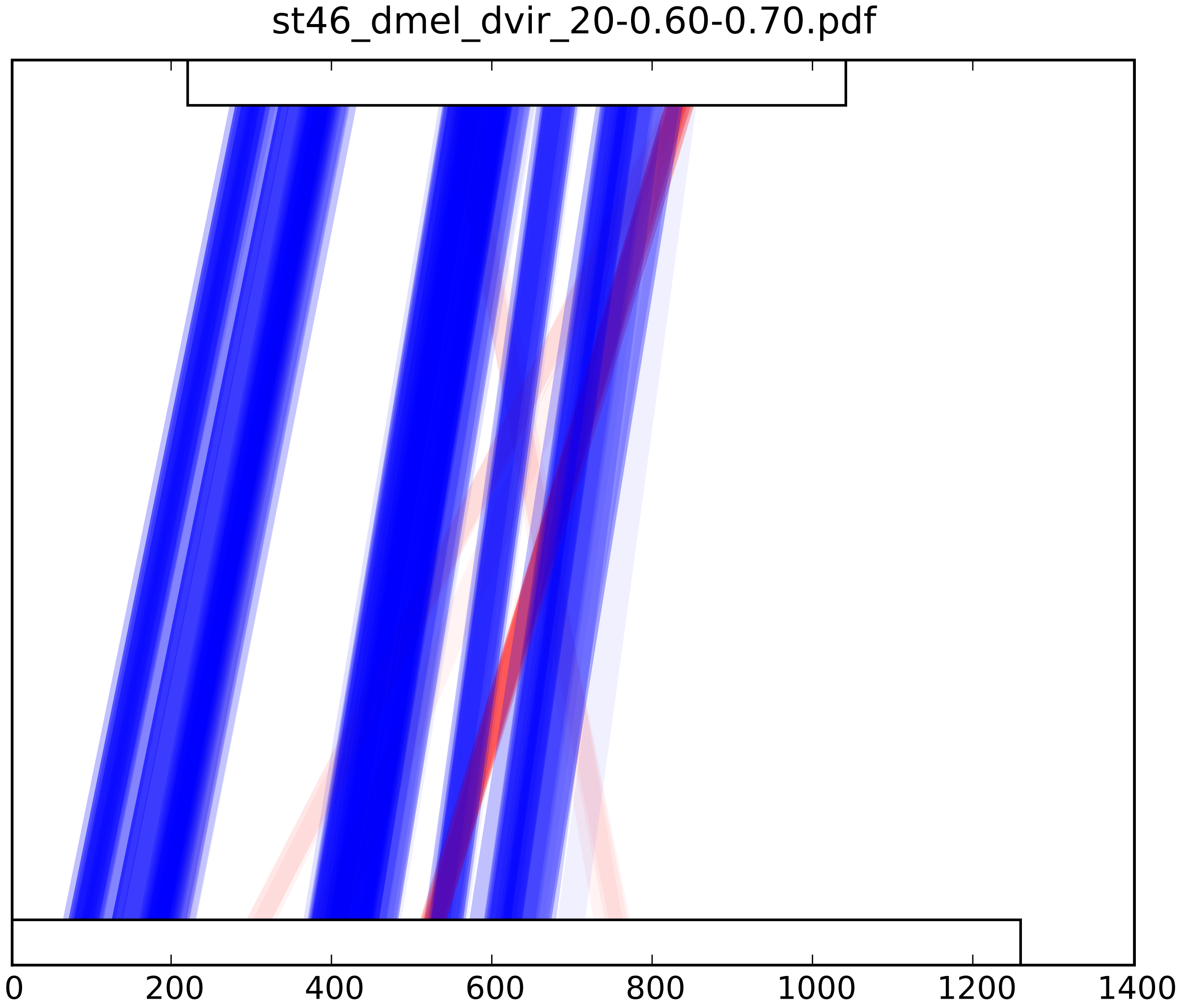
st46_dmel_dvir_20-0.60-0.70.pdf
0
200
400
600
800
1000
1200
1400

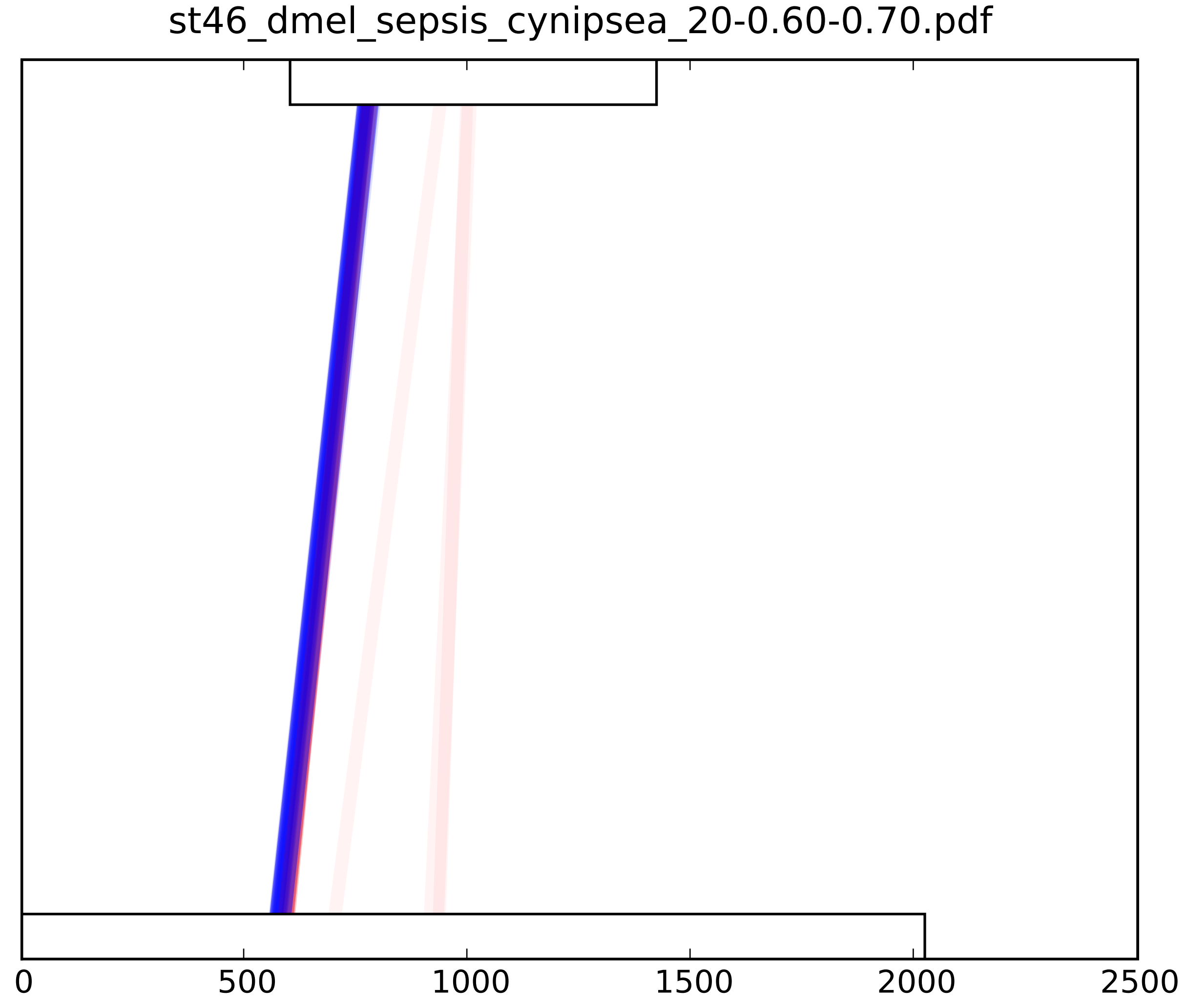
st46_dmel_sepsis_cynipsea_20-0.60-0.70.pdf
0
500
1000
1500
2000
2500

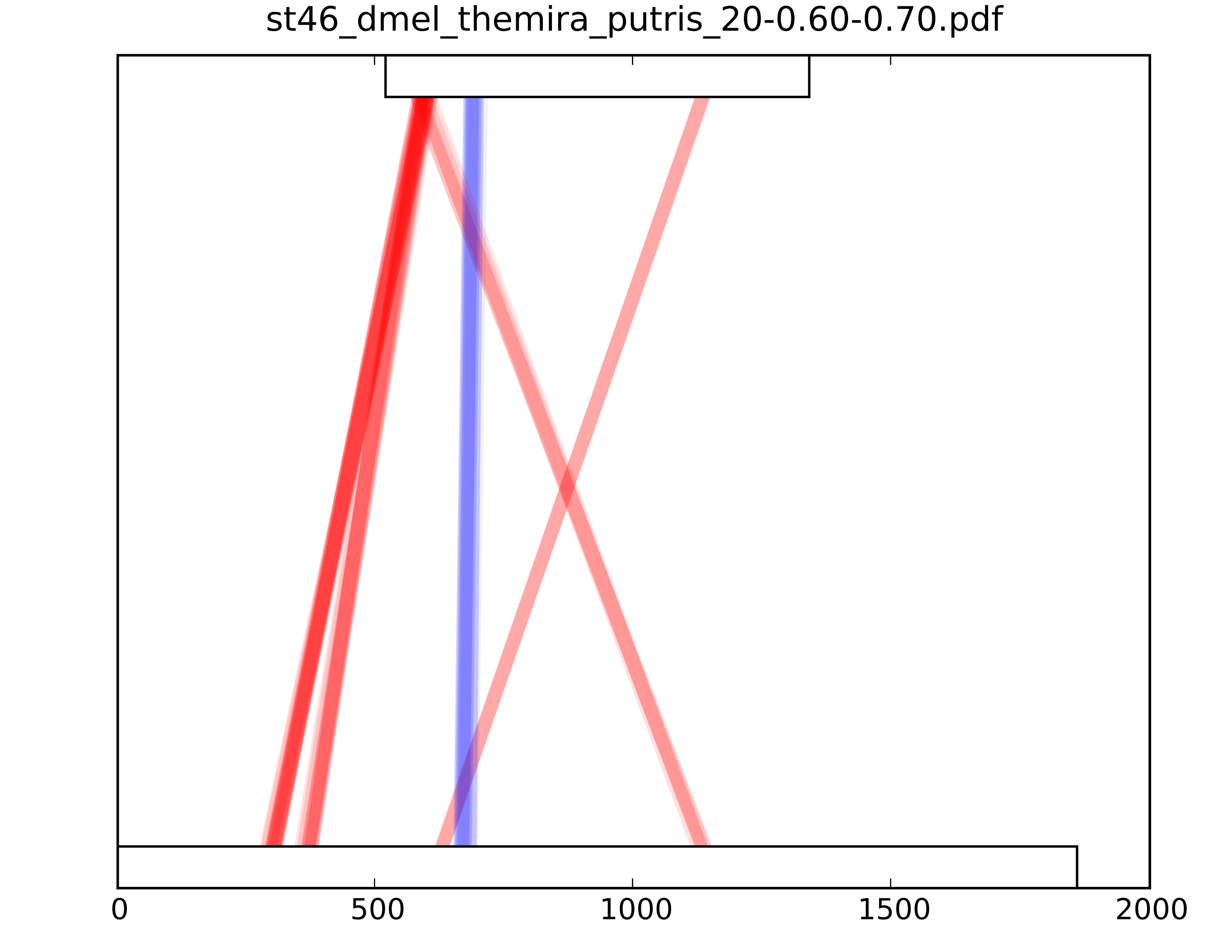
st46_dmel_themira_putris_20-0.60-0.70.pdf
0
500
1000
1500
2000

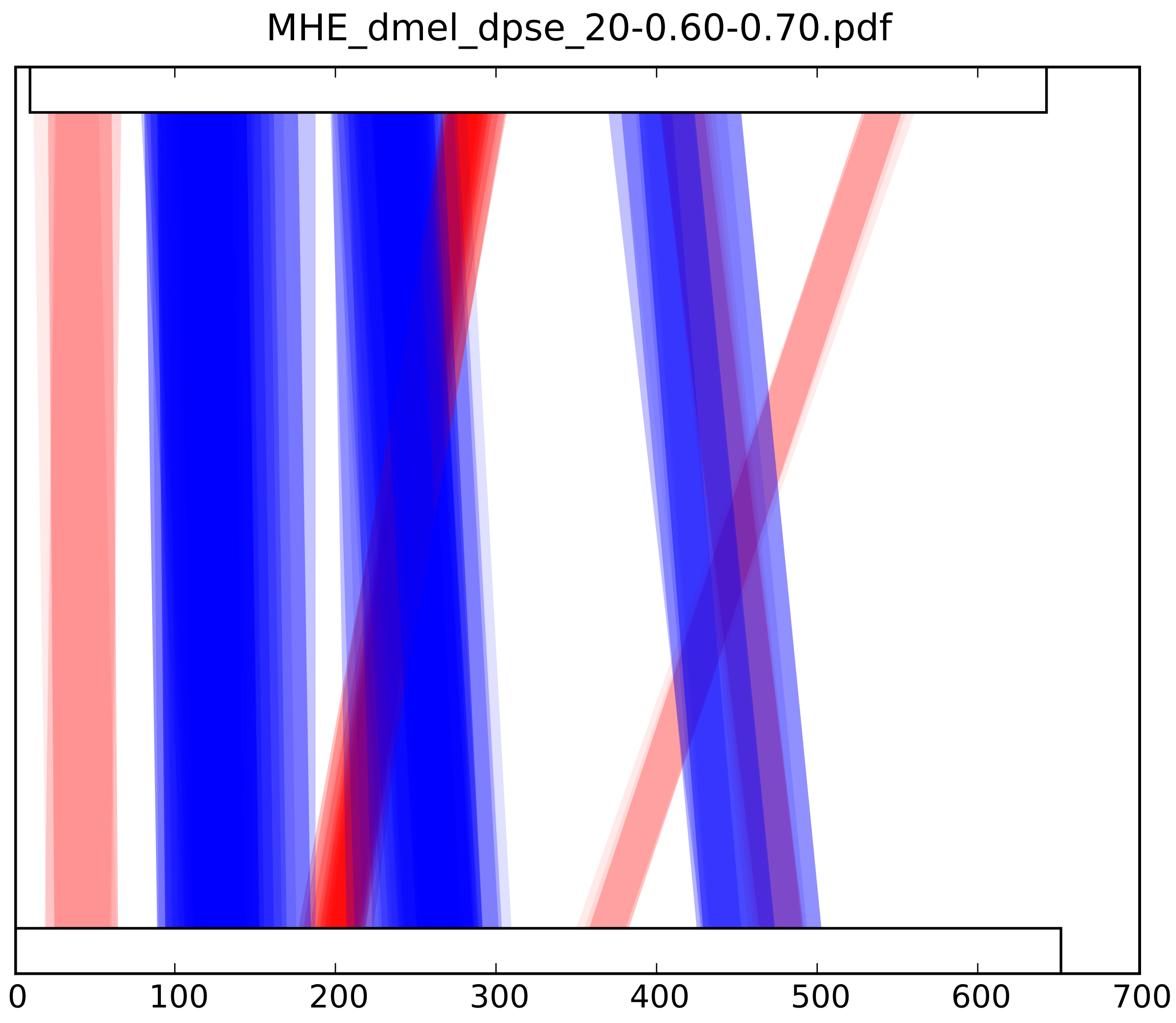
MHE_dmel_dpse_20-0.60-0.70.pdf
0
100
200
300
400
500
600
700

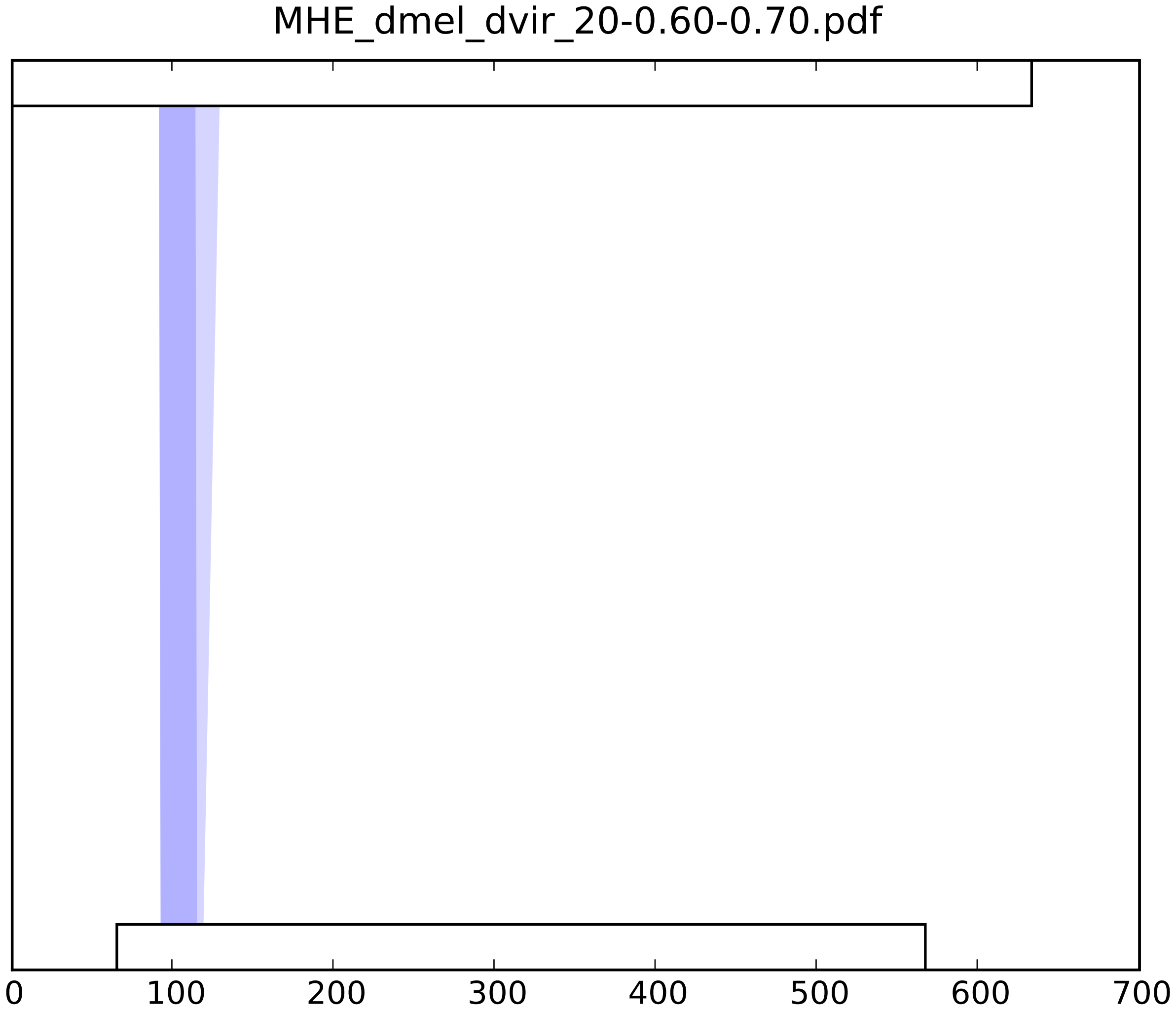
MHE_dmel_dvir_20-0.60-0.70.pdf
0
100
200
300
400
500
600
700

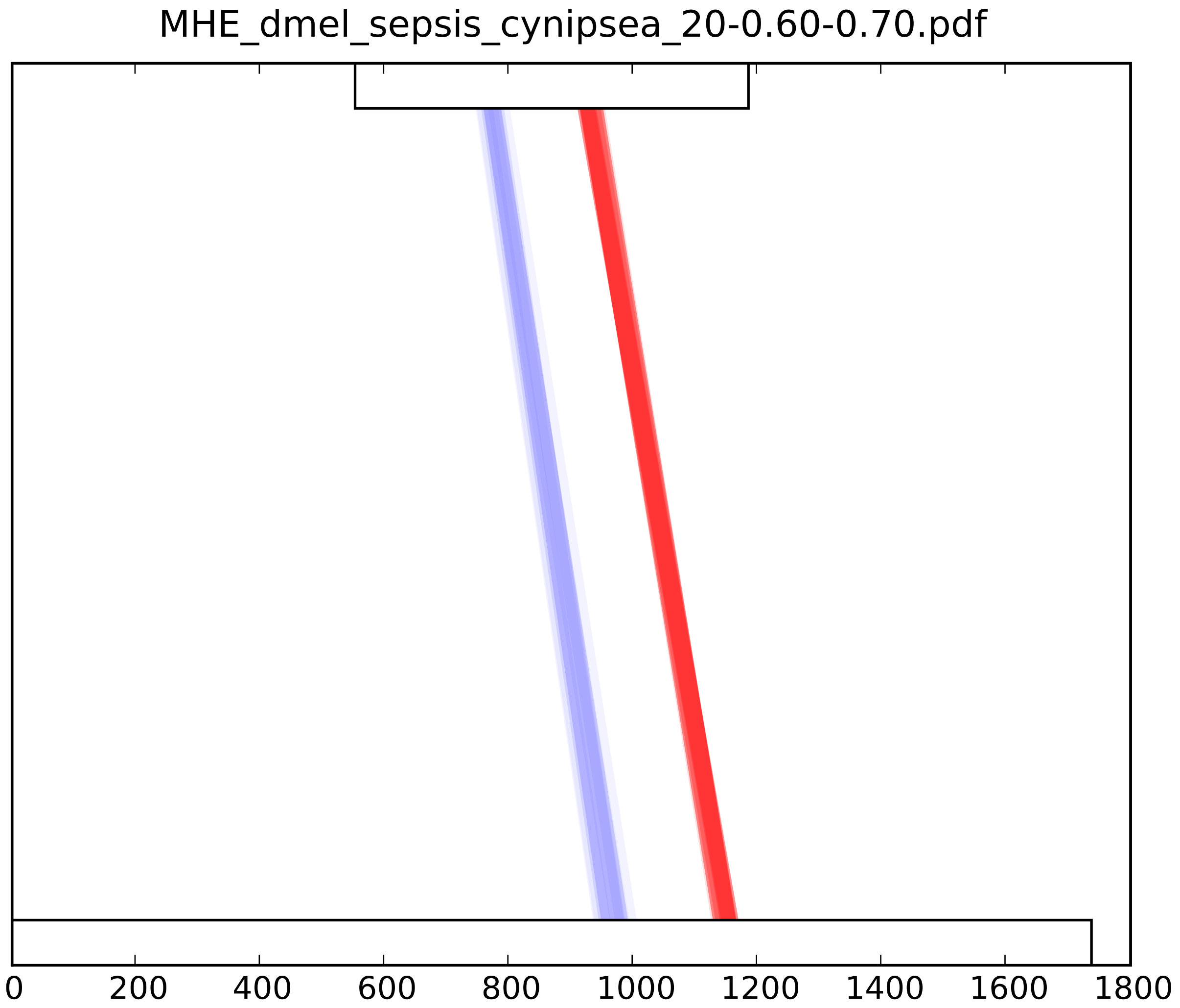
MHE_dmel_sepsis_cynipsea_20-0.60-0.70.pdf
0
200
400
600
800
1000
1200
1400
1600
1800

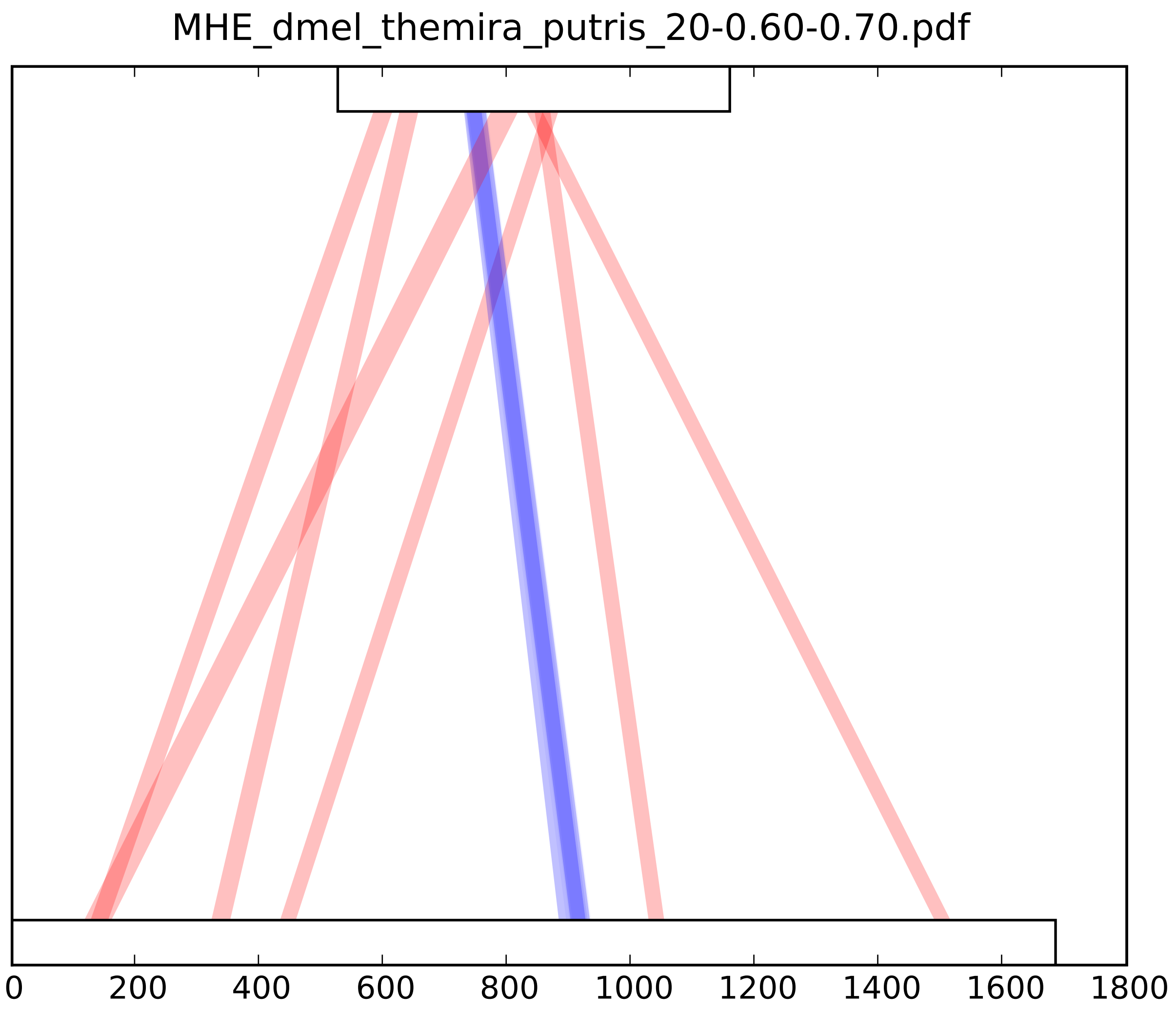

MHE_dmel_themira_putris_20-0.60-0.70.pdf
0
200
400
600
800
1000
1200
1400
1600
1800

st2_dmel_dpse_14-0.60-0.50.pdf

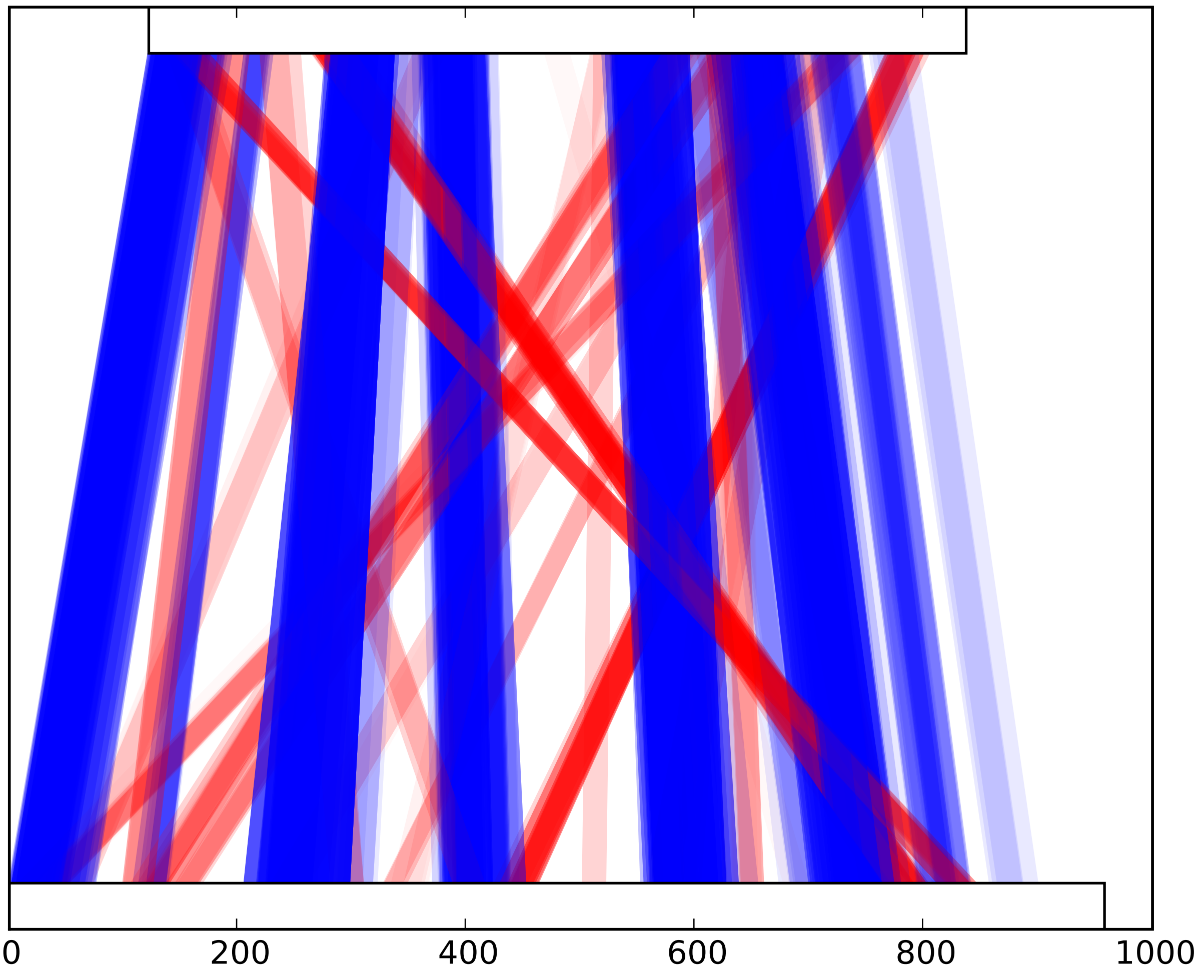

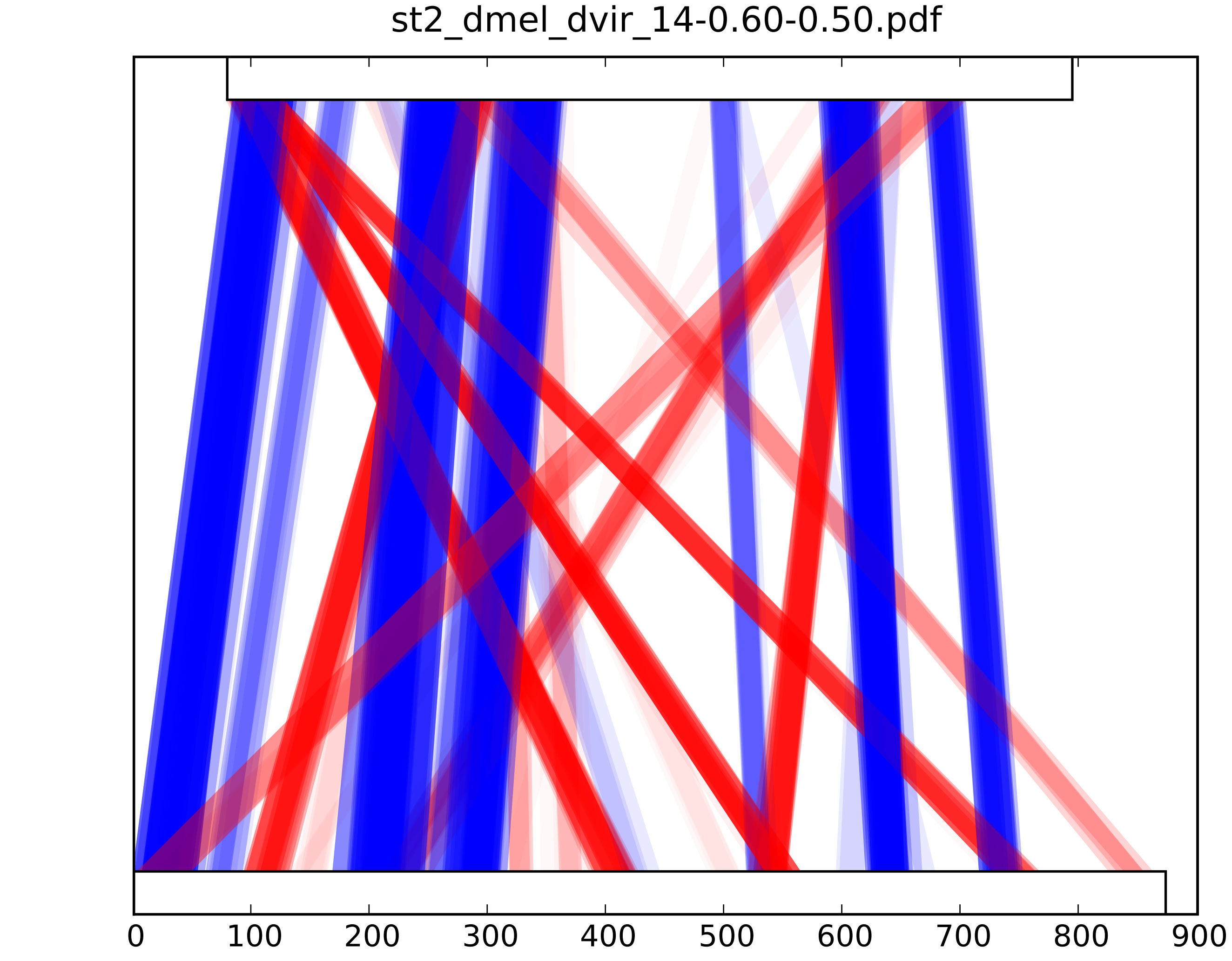
st2_dmel_dvir_14-0.60-0.50.pdf
0
100
200
300
400
500
600
700
800
900

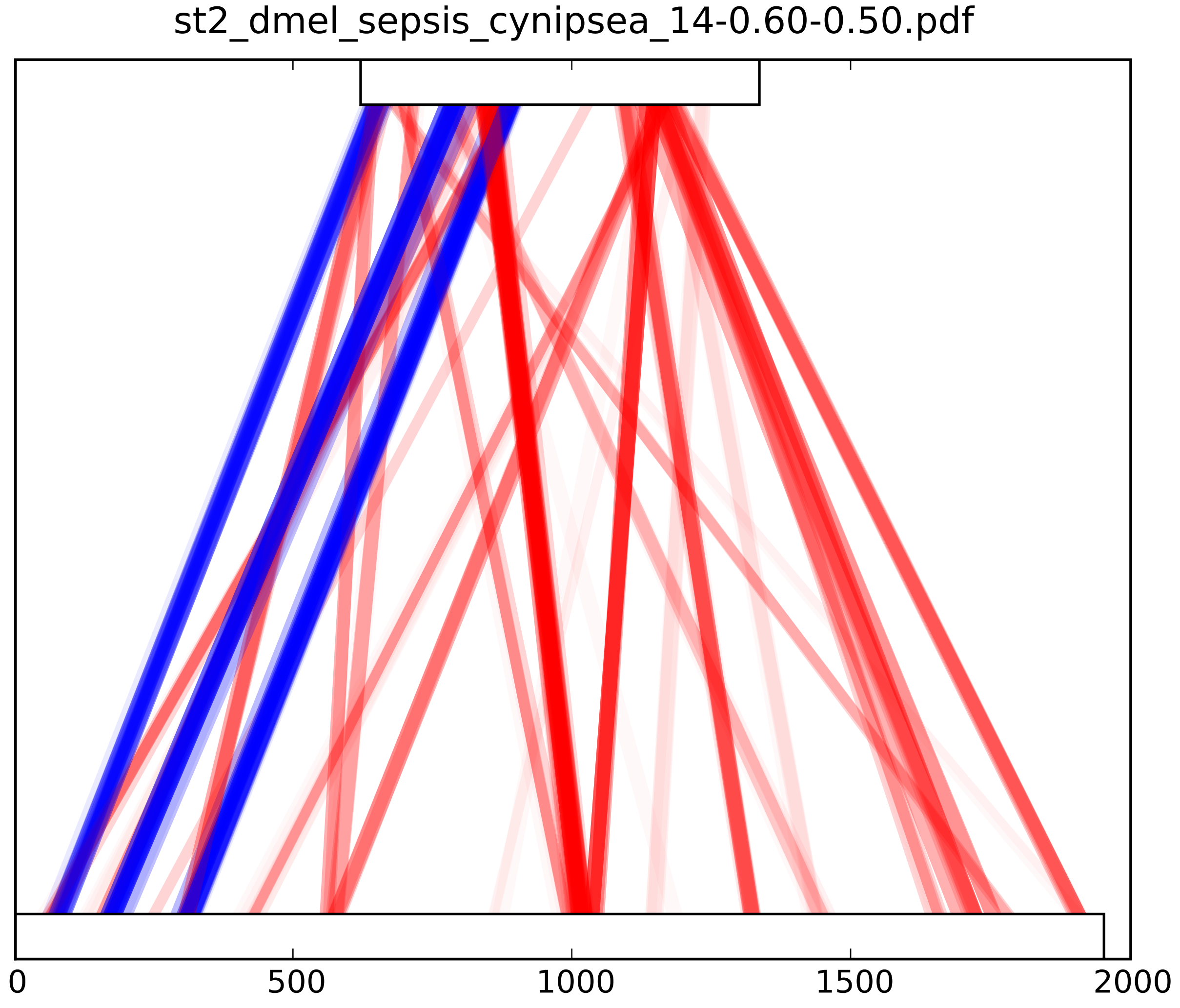
st2_dmel_sepsis_cynipsea_14-0.60-0.50.pdf
0
500
1000
1500
2000

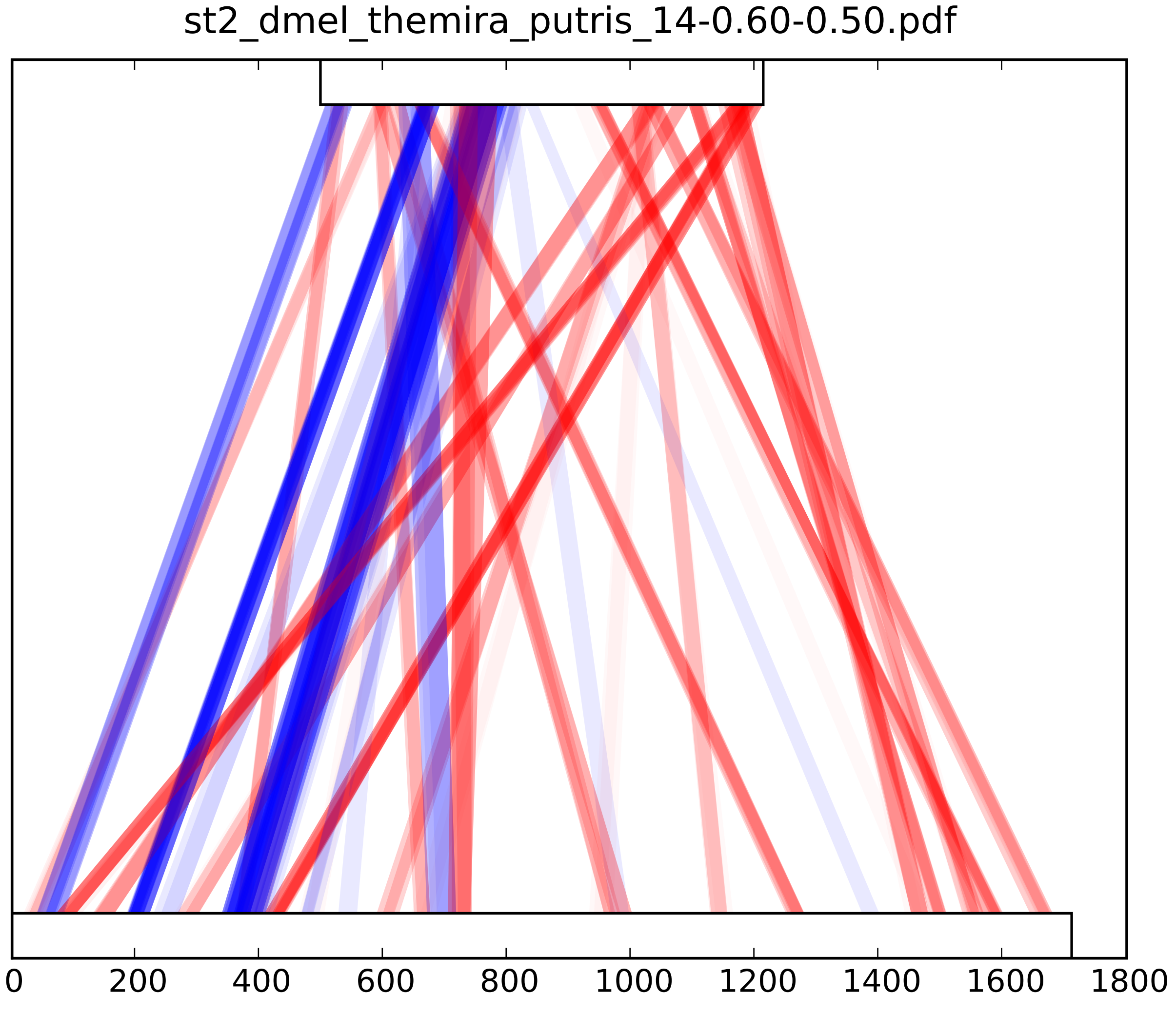

st2_dmel_themira_putris_14-0.60-0.50.pdf
0
200
400
600
800
1000
1200
1400
1600
1800

st37_dmel_dpse_14-0.60-0.50.pdf

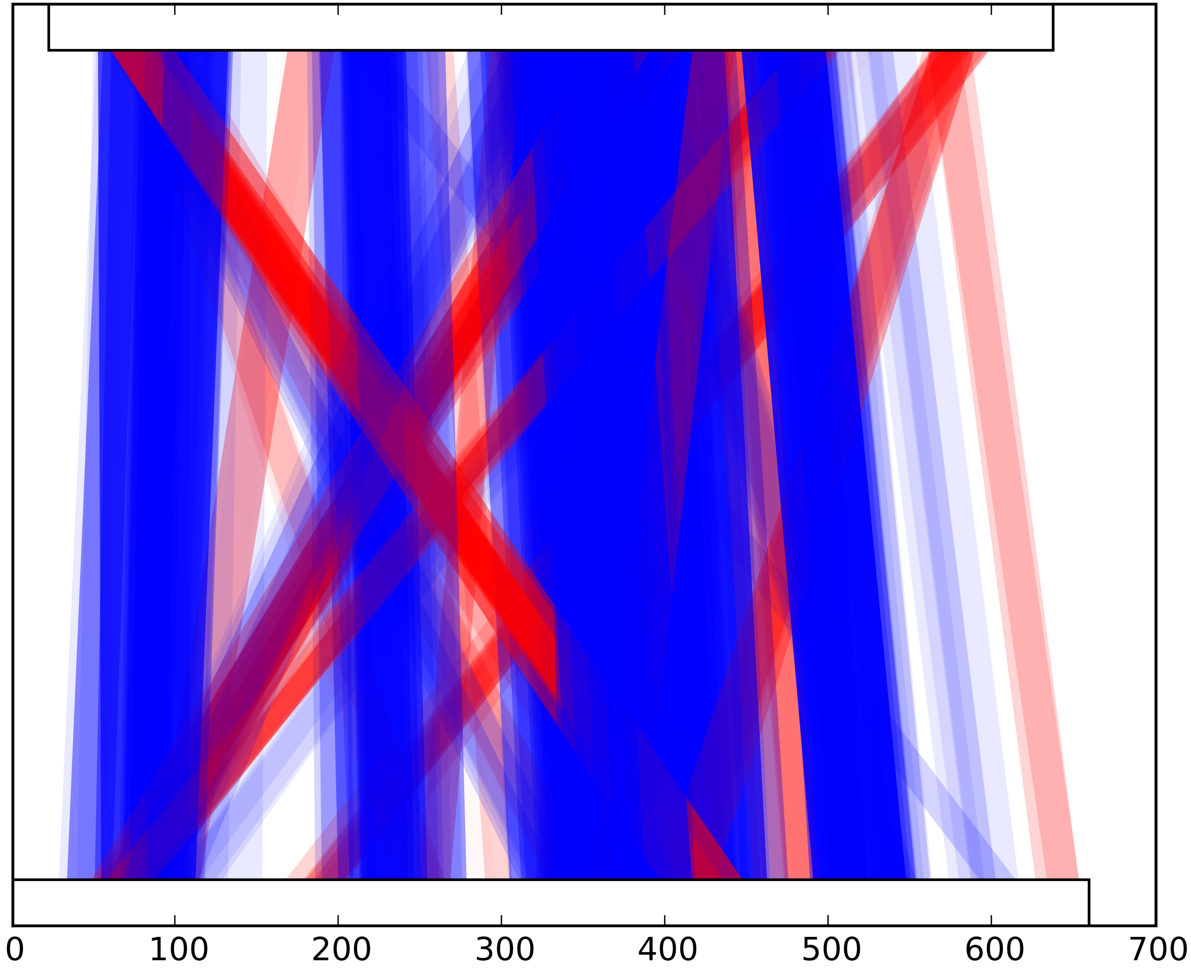

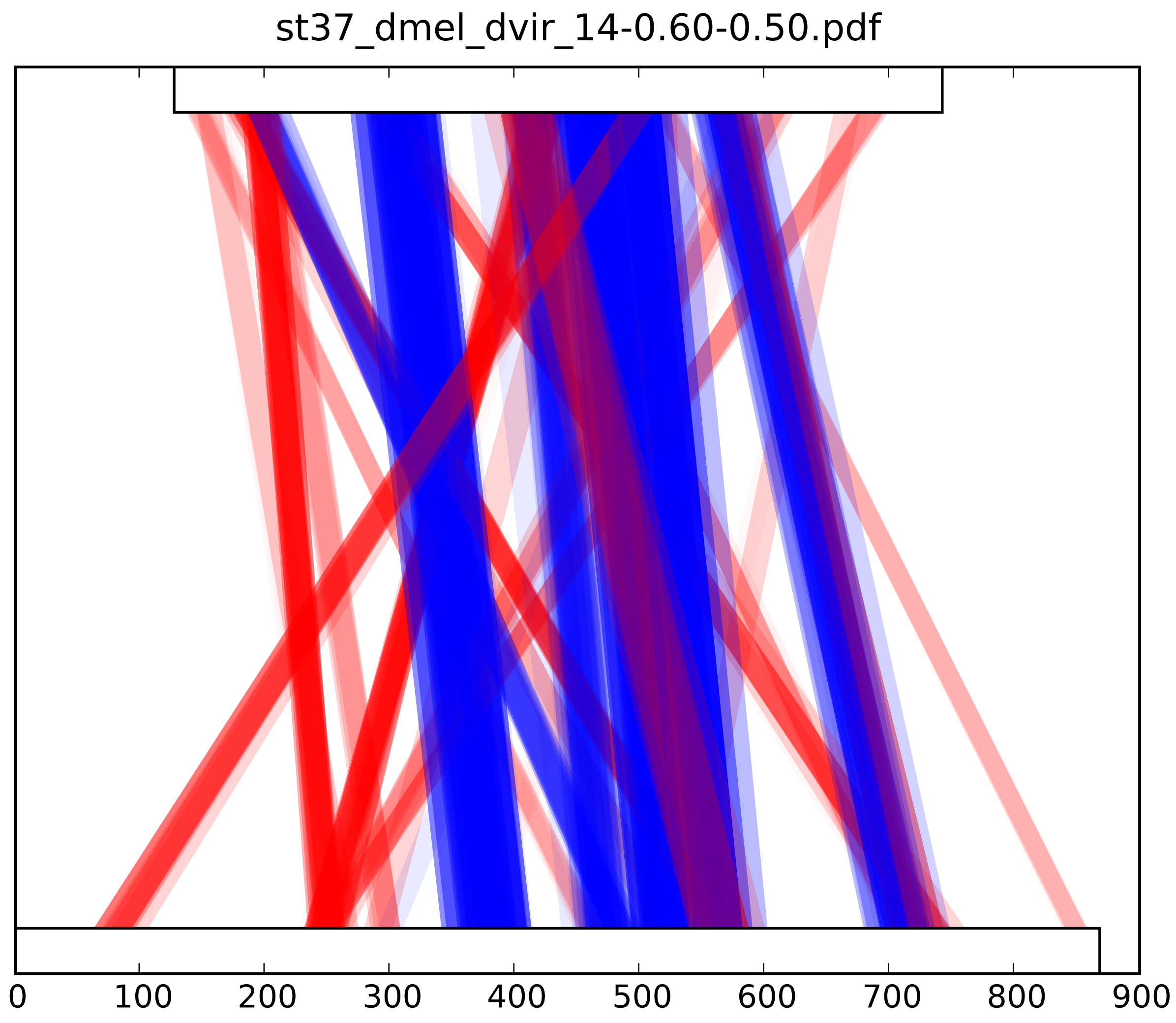
st37_dmel_dvir_14-0.60-0.50.pdf
0
100
200
300
400
500
600
700
800
900

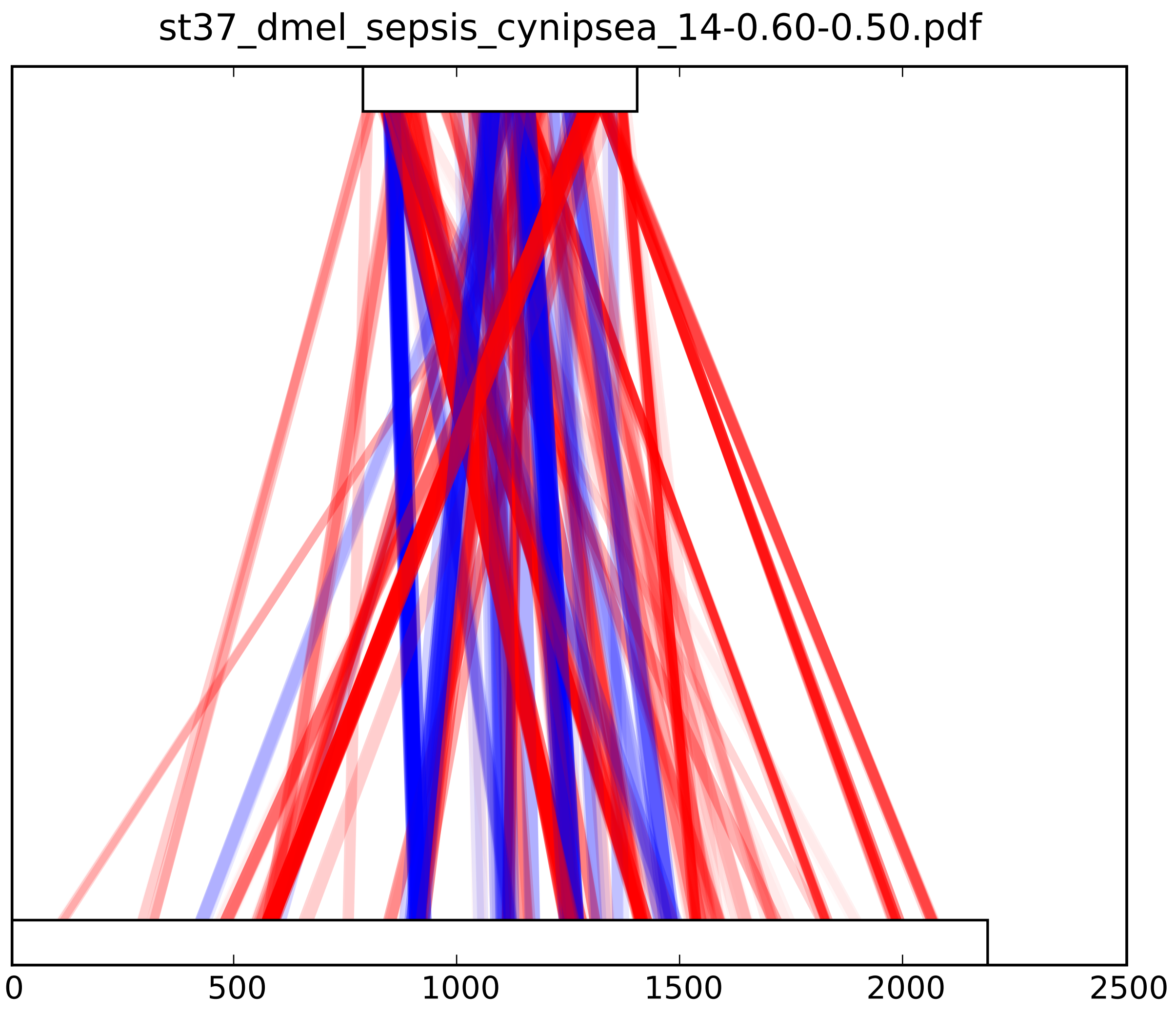

st37_dmel_sepsis_cynipsea_14-0.60-0.50.pdf
0
500
1000
1500
2000
2500

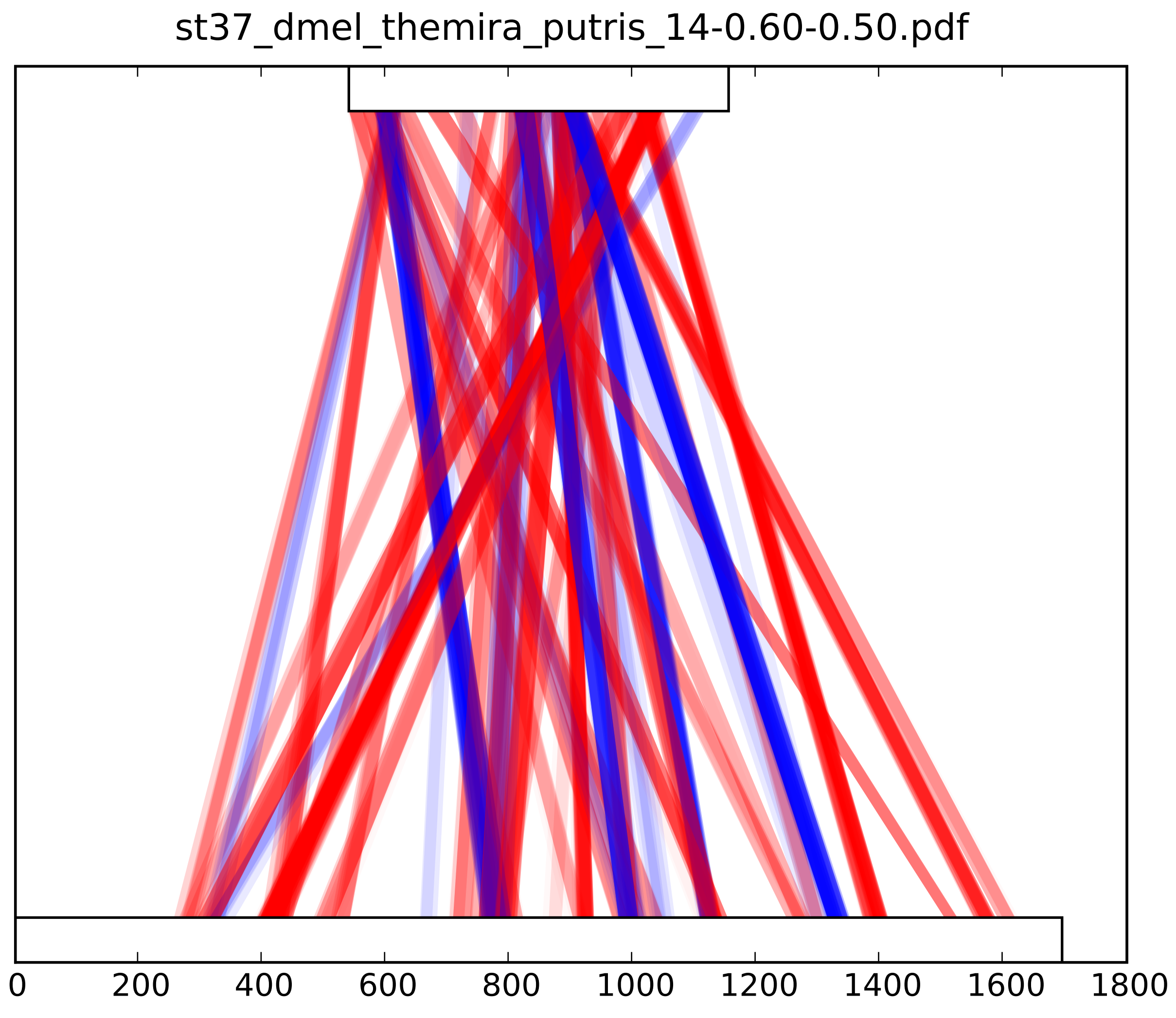
st37_dmel_themira_putris_14-0.60-0.50.pdf
0
200
400
600
800
1000
1200
1400
1600
1800

st46_dmel_dpse_14-0.60-0.50.pdf

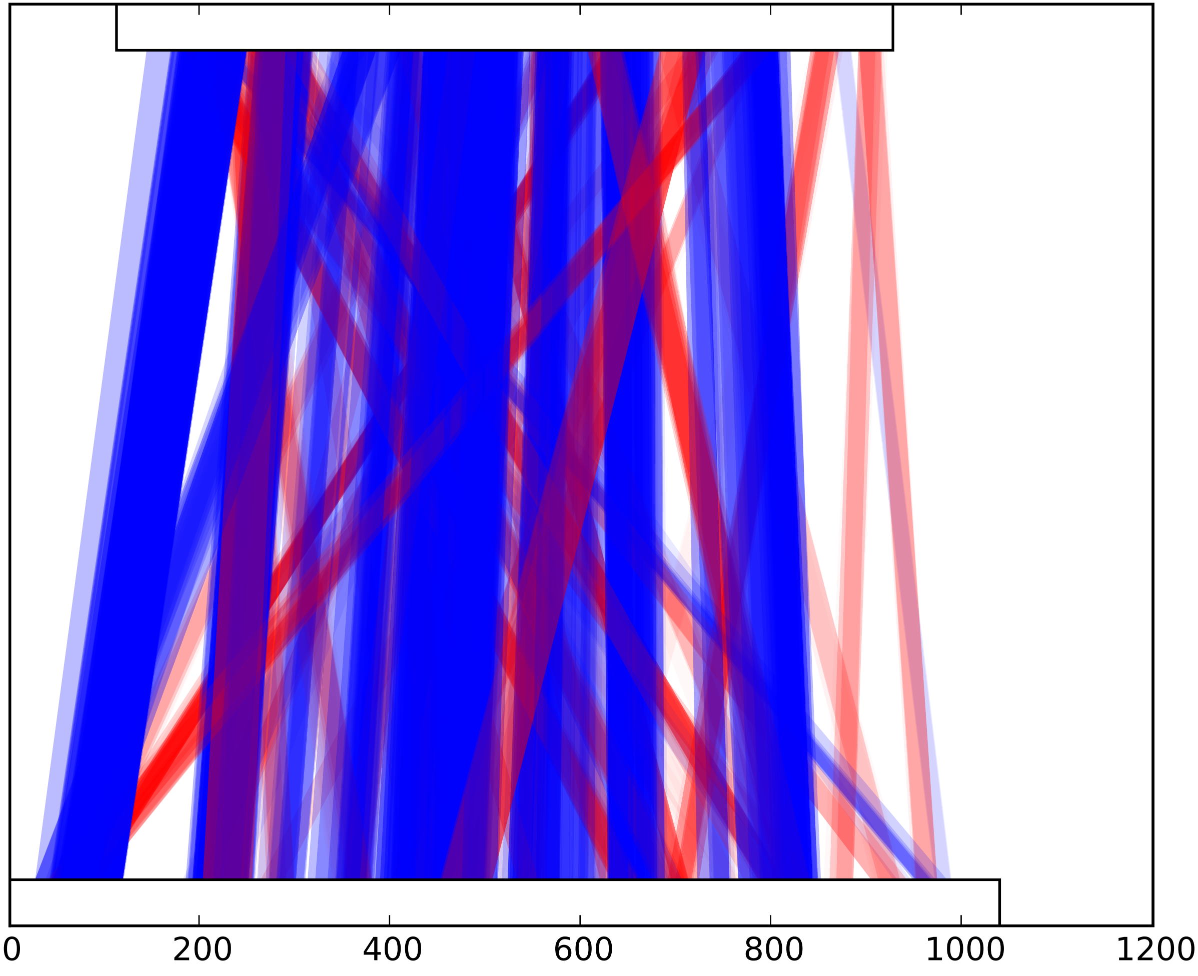

st46_dmel_dvir_14-0.60-0.50.pdf

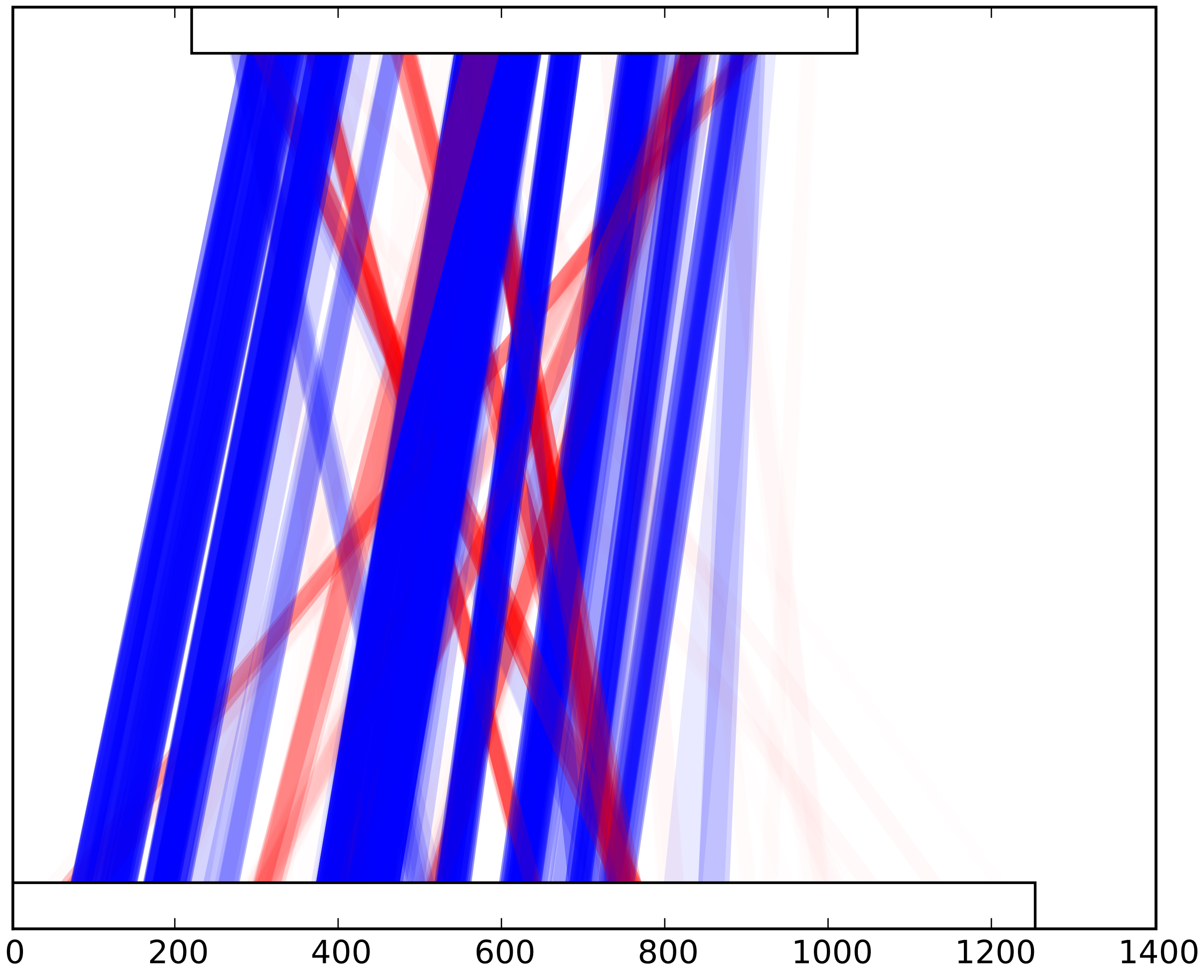

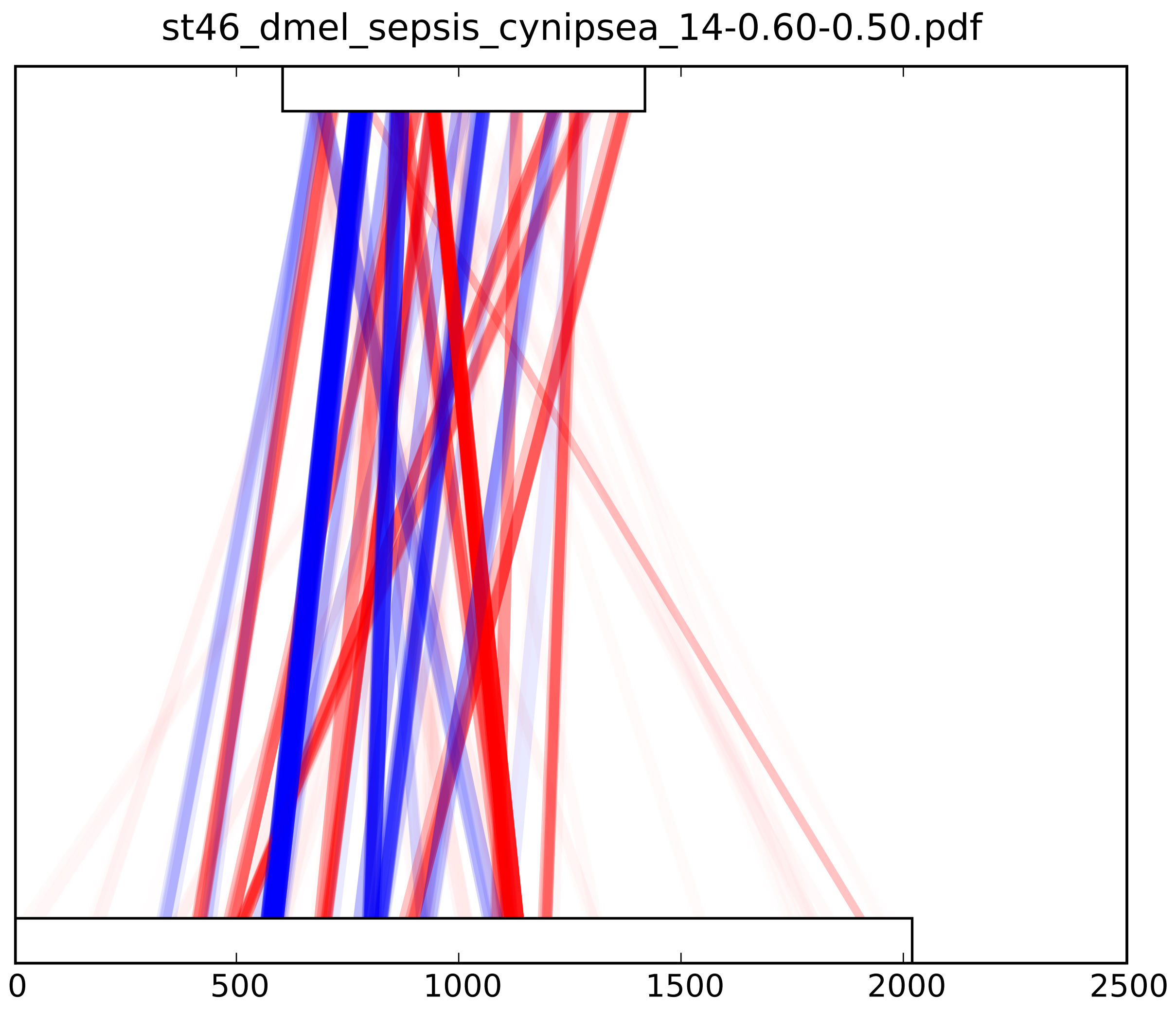

st46_dmel_sepsis_cynipsea_14-0.60-0.50.pdf
0
500
1000
1500
2000
2500

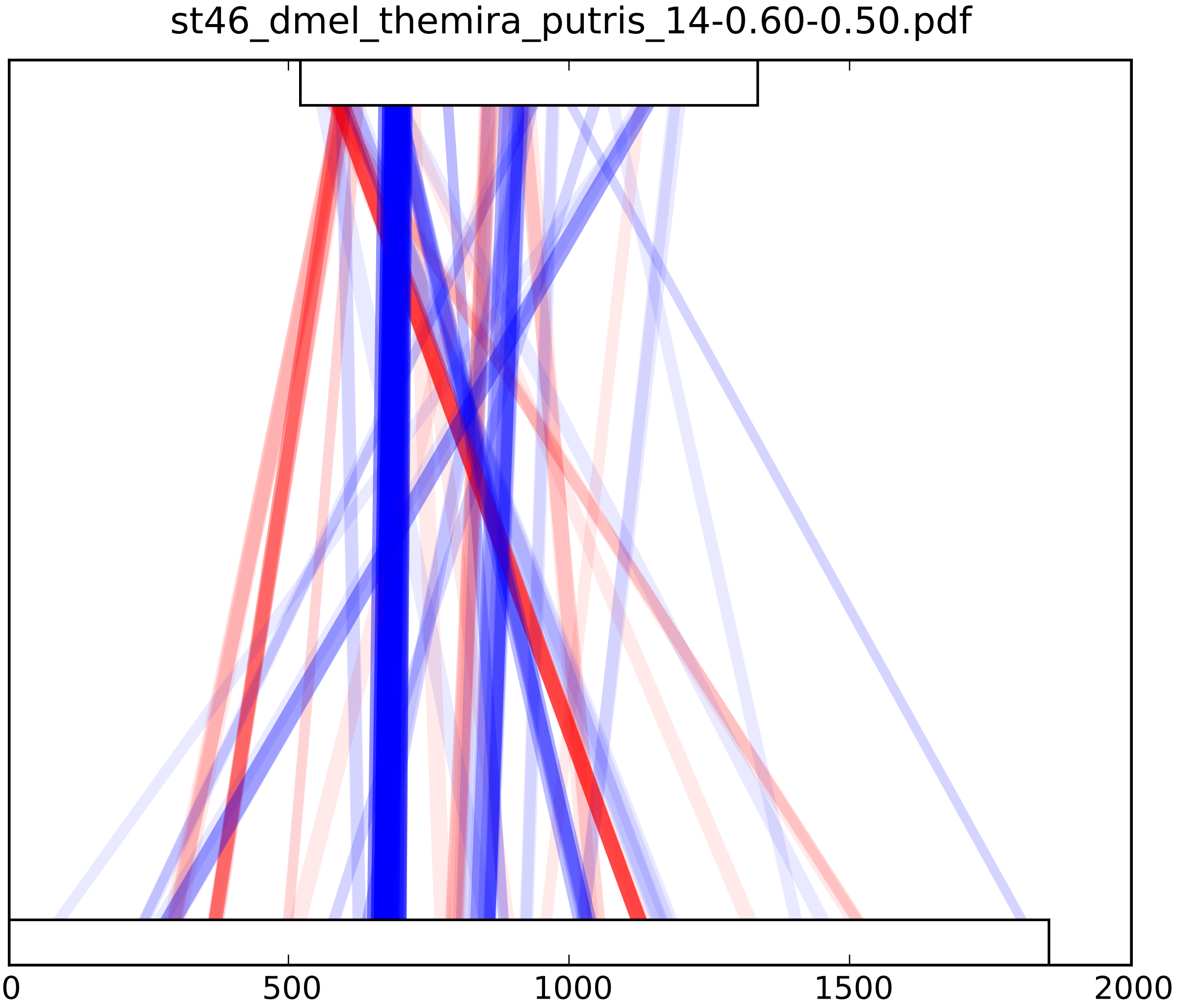
st46_dmel_themira_putris_14-0.60-0.50.pdf
0
500
1000
1500
2000

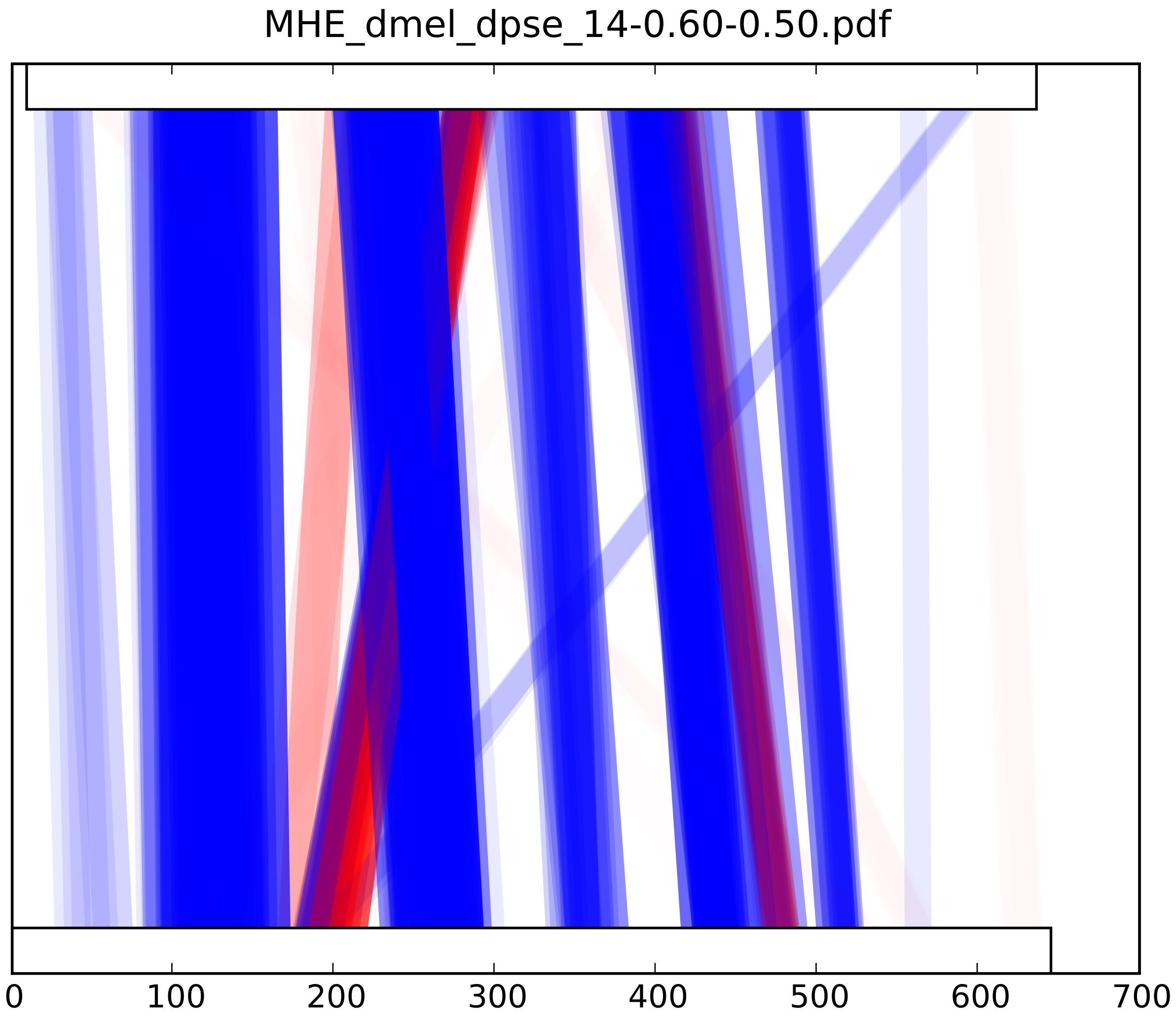

MHE_dmel_dpse_14-0.60-0.50.pdf
0
100
200
300
400
500
600
700

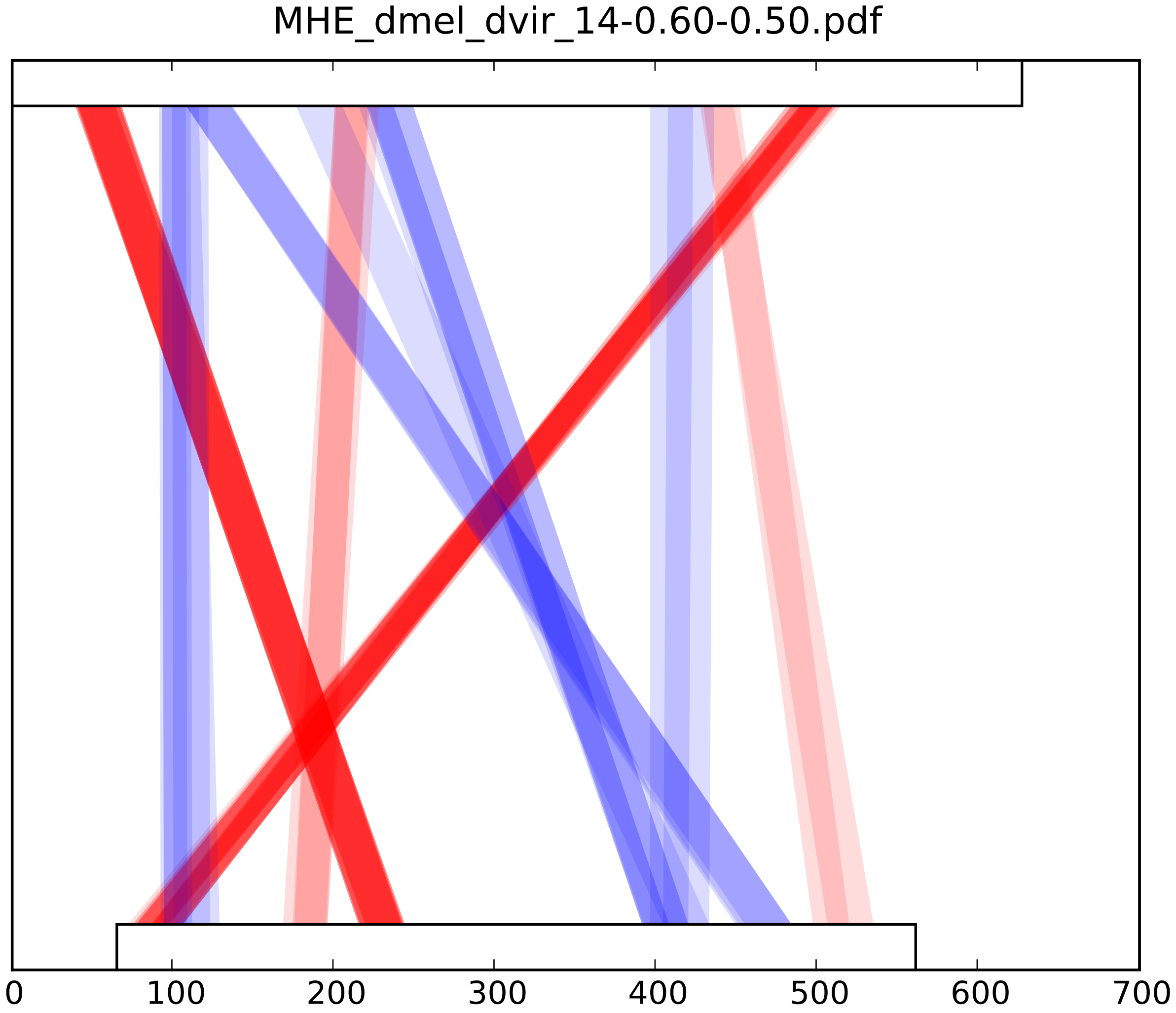
MHE_dmel_dvir_14-0.60-0.50.pdf
0
100
200
300
400
500
600
700

MHE_dmel_sepsis_cynipsea_14-0.60-0.50.pdf

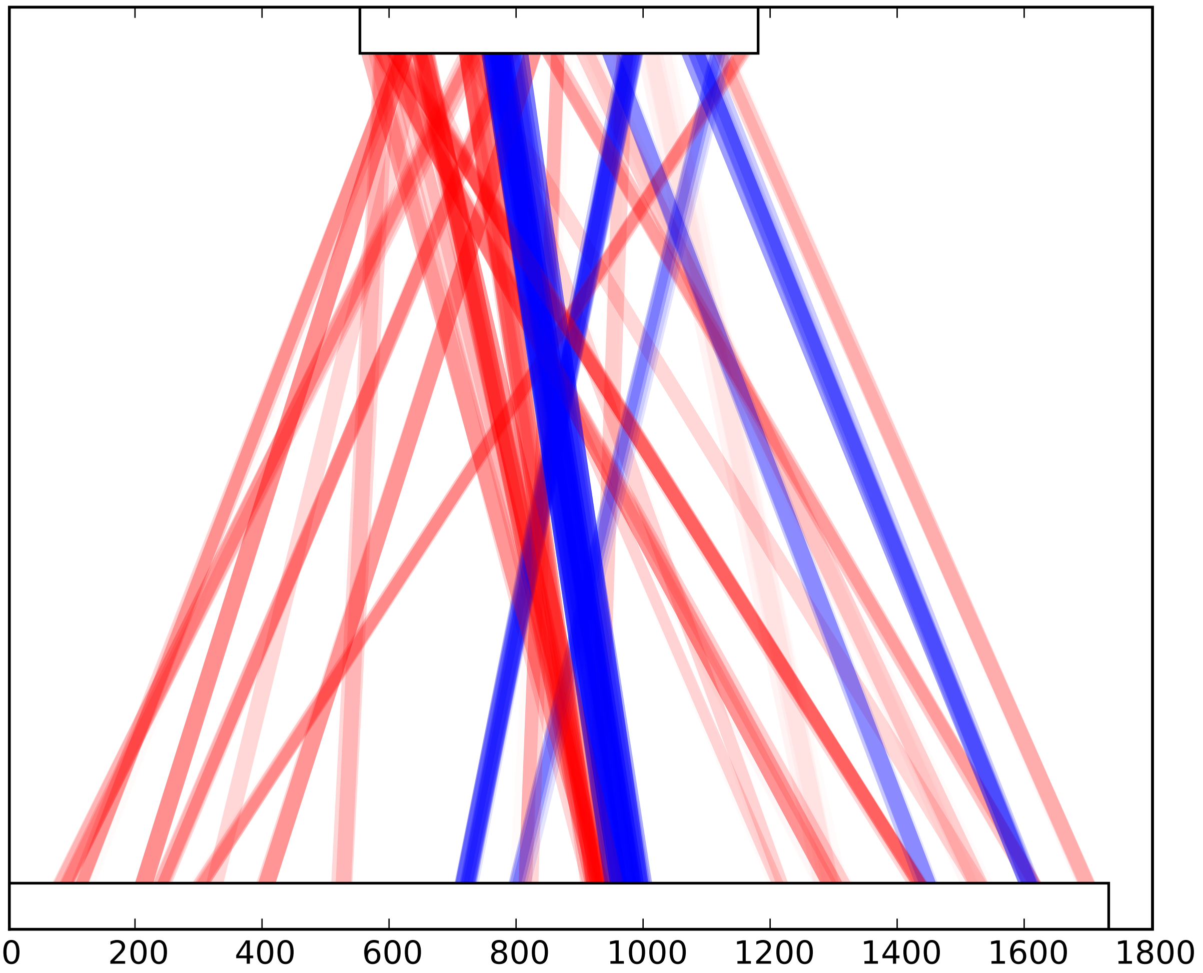

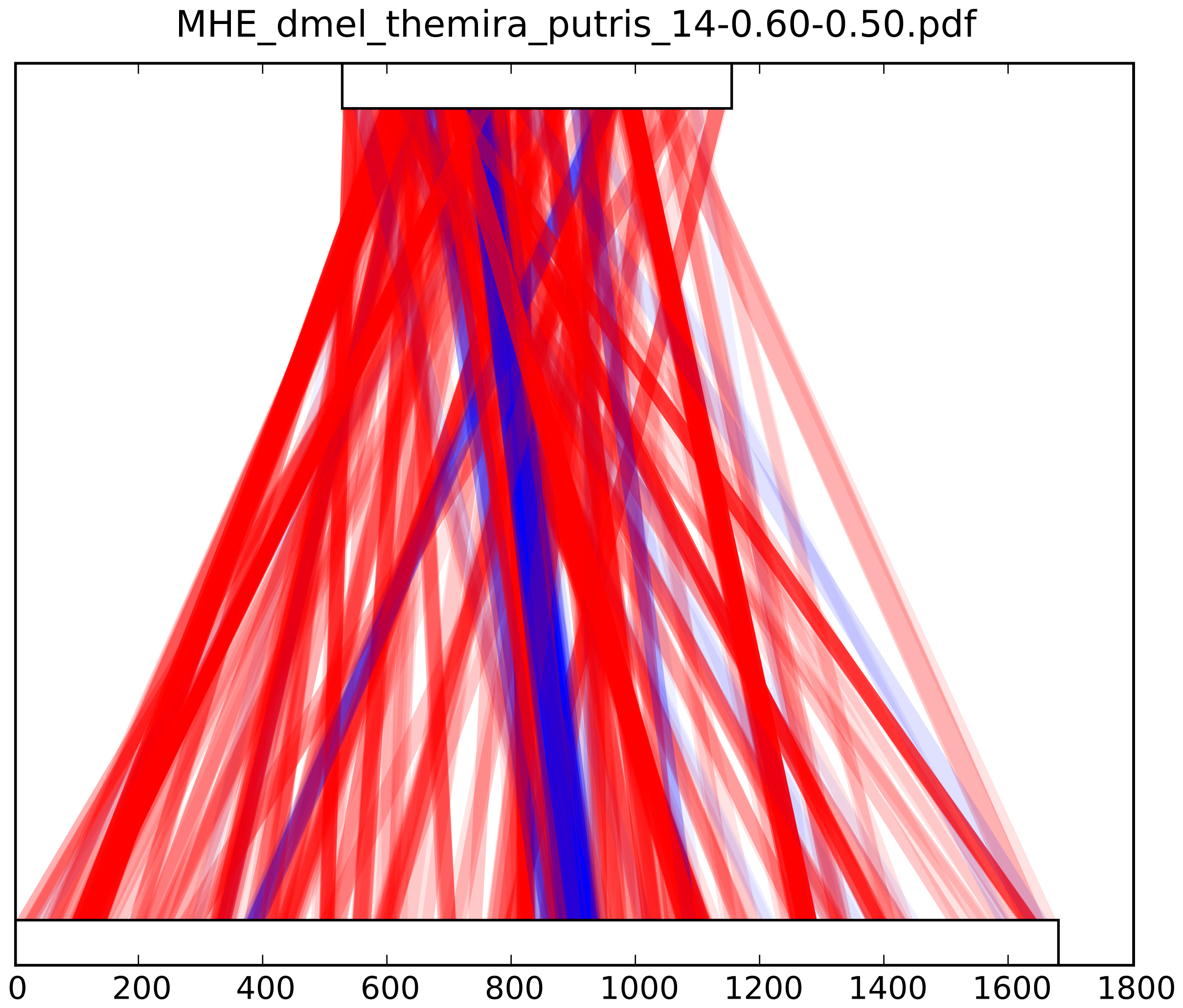
MHE_dmel_themira_putris_14-0.60-0.50.pdf
0
200
400
600
800
1000
1200
1400
1600
1800

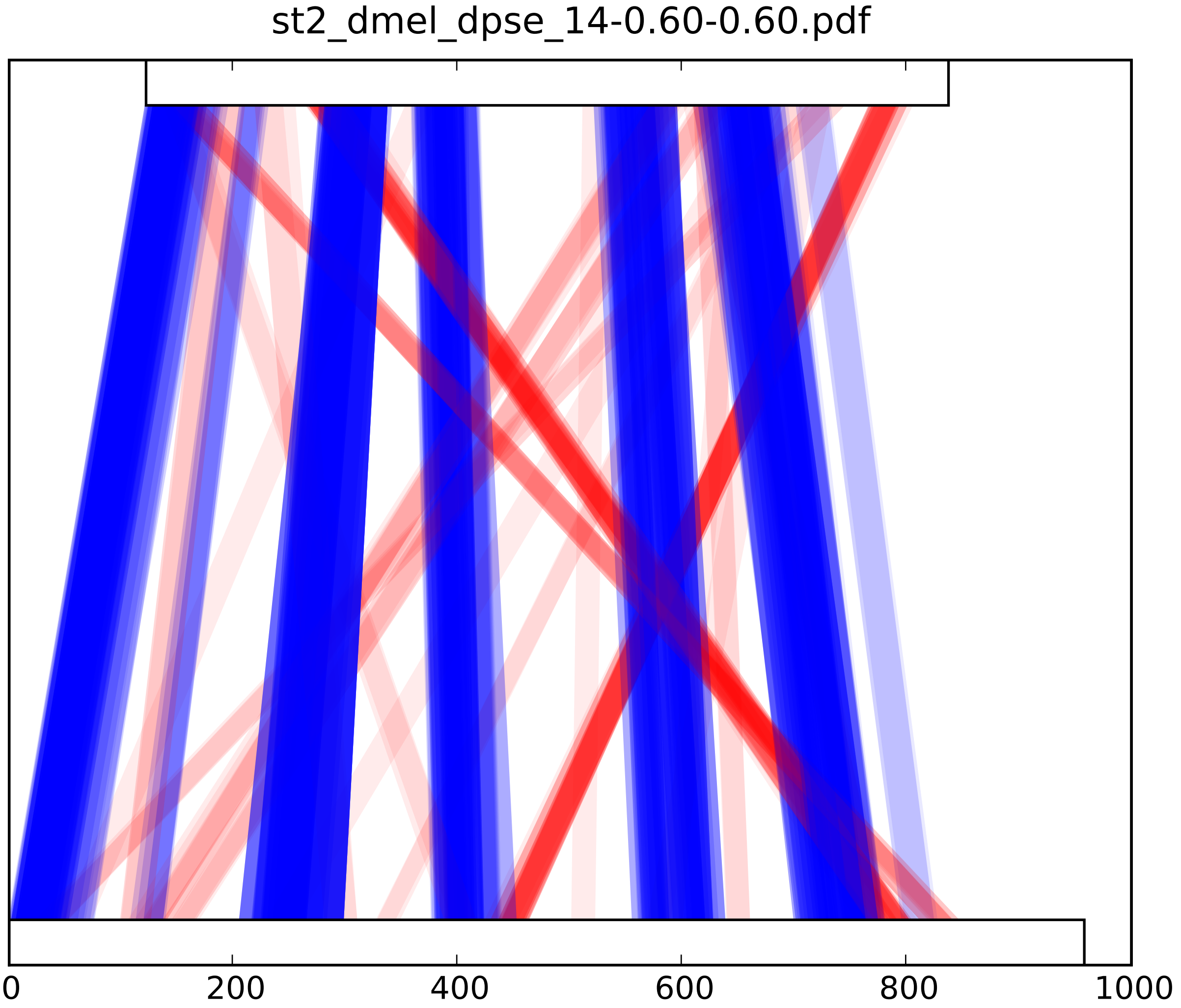
st2_dmel_dpse_14-0.60-0.60.pdf
0
200
400
600
800
1000

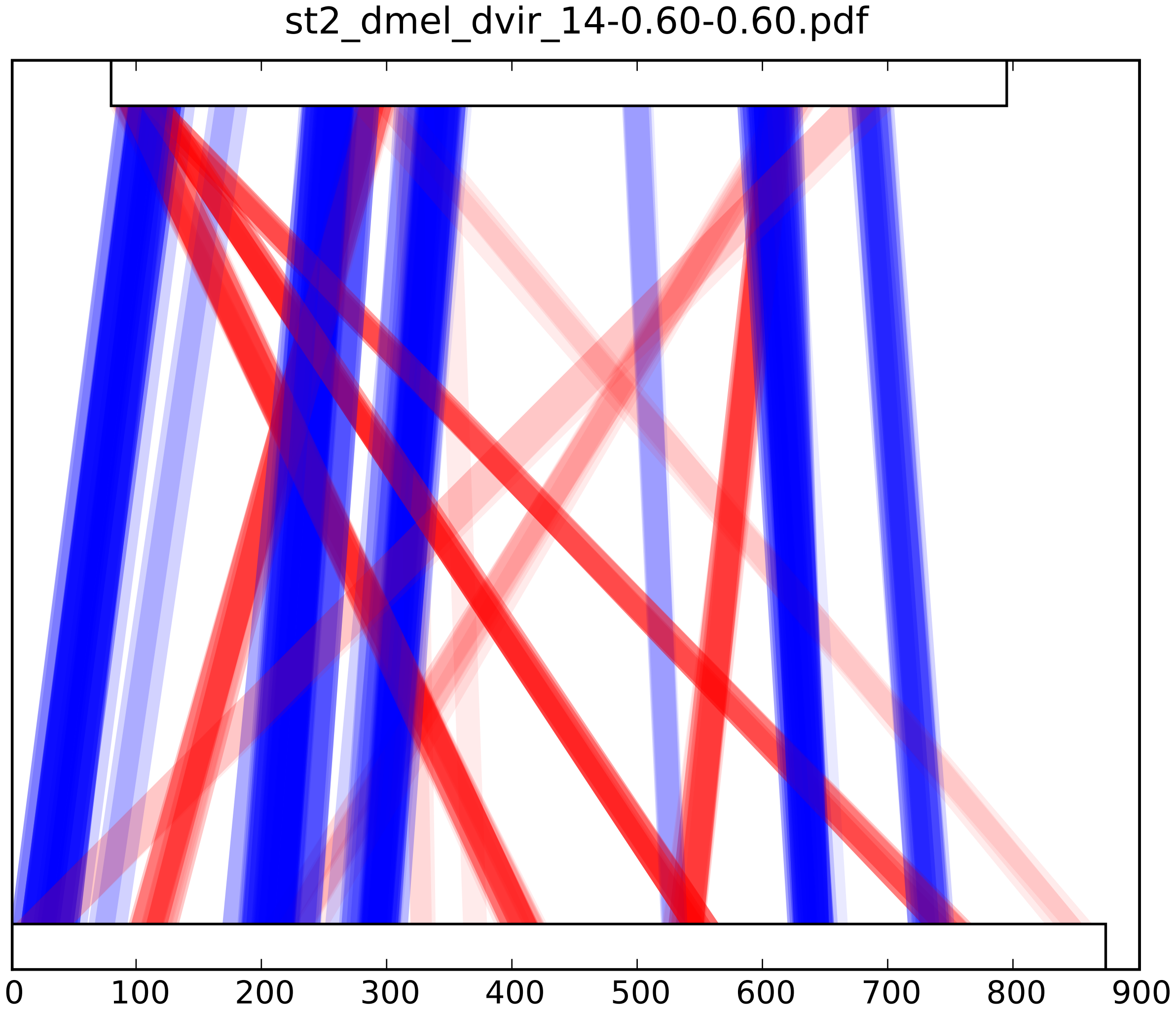

st2_dmel_dvir_14-0.60-0.60.pdf
0
100
200
300
400
500
600
700
800
900

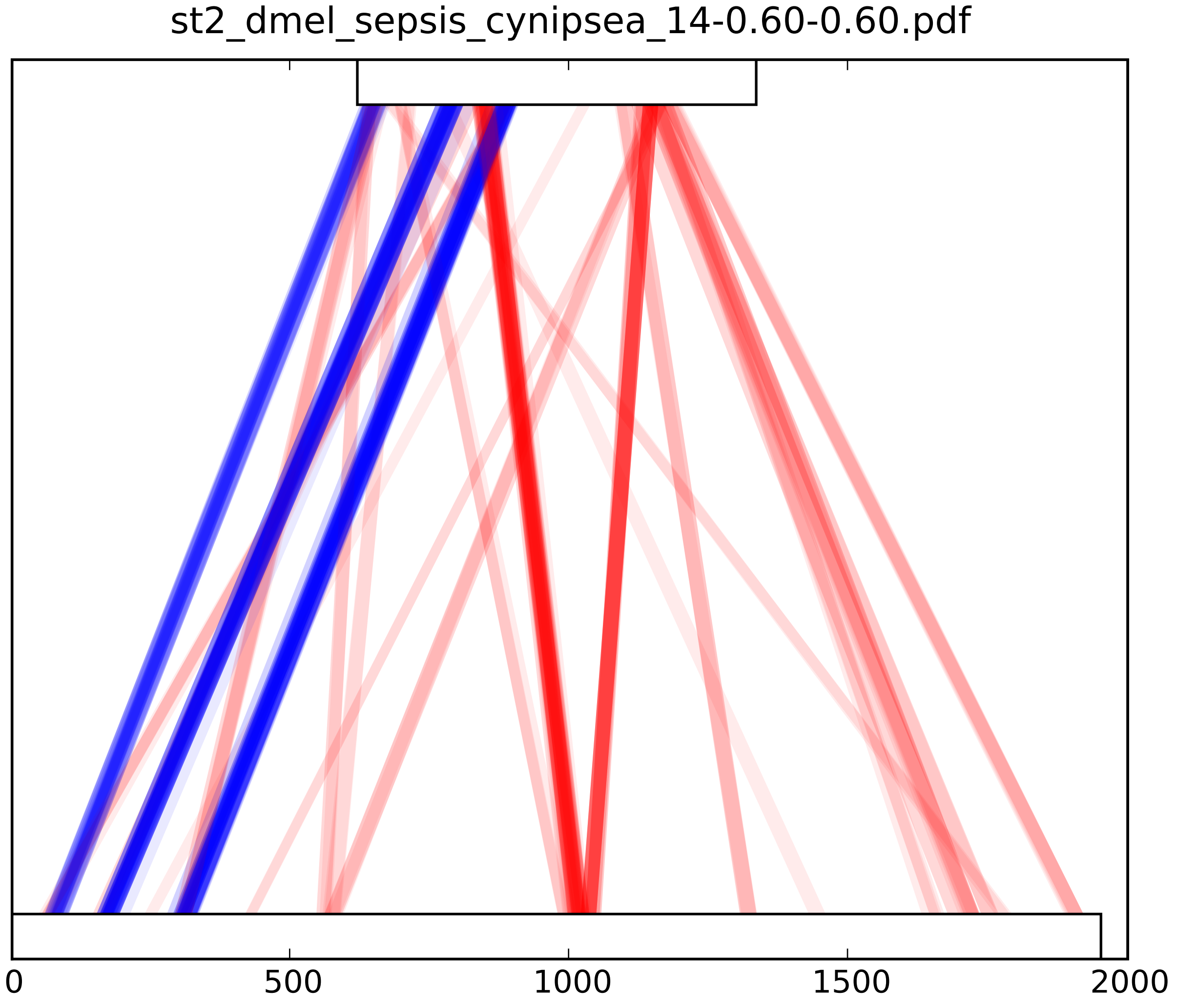
st2_dmel_sepsis_cynipsea_14-0.60-0.60.pdf
0
500
1000
1500
2000

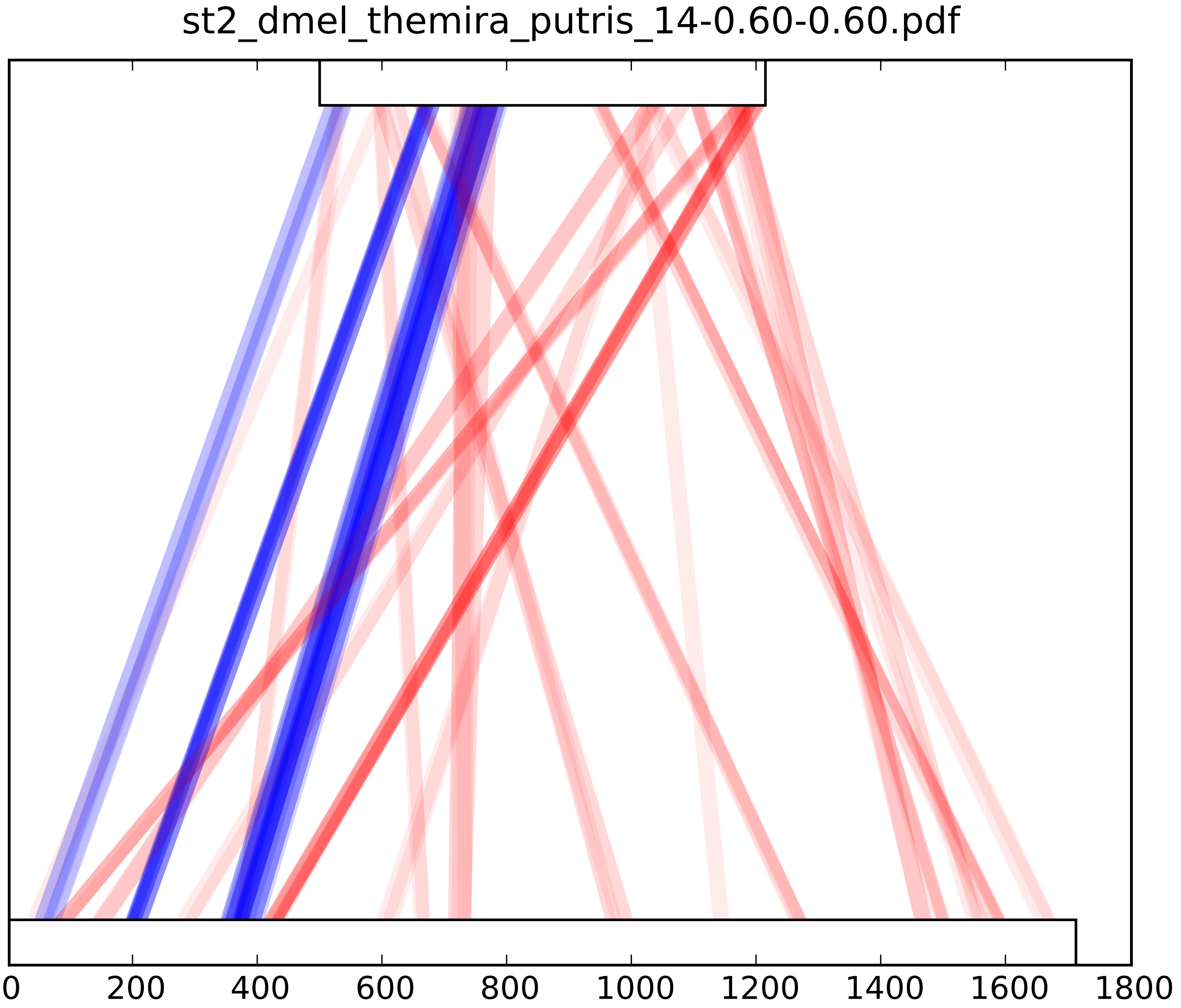
st2_dmel_themira_putris_14-0.60-0.60.pdf
0
200
400
600
800
1000
1200
1400
1600
1800

st37_dmel_dpse_14-0.60-0.60.pdf

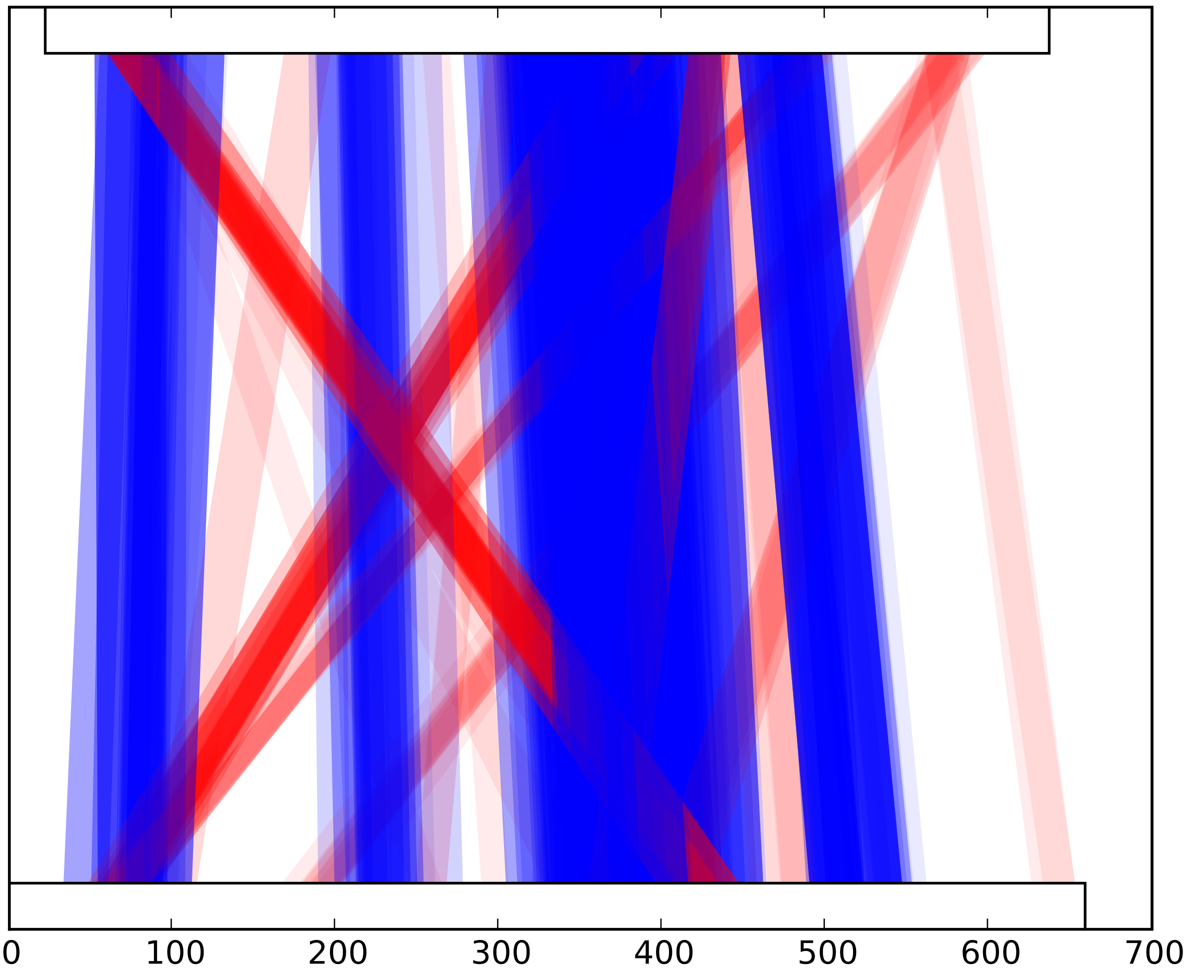

st37_dmel_dvir_14-0.60-0.60.pdf

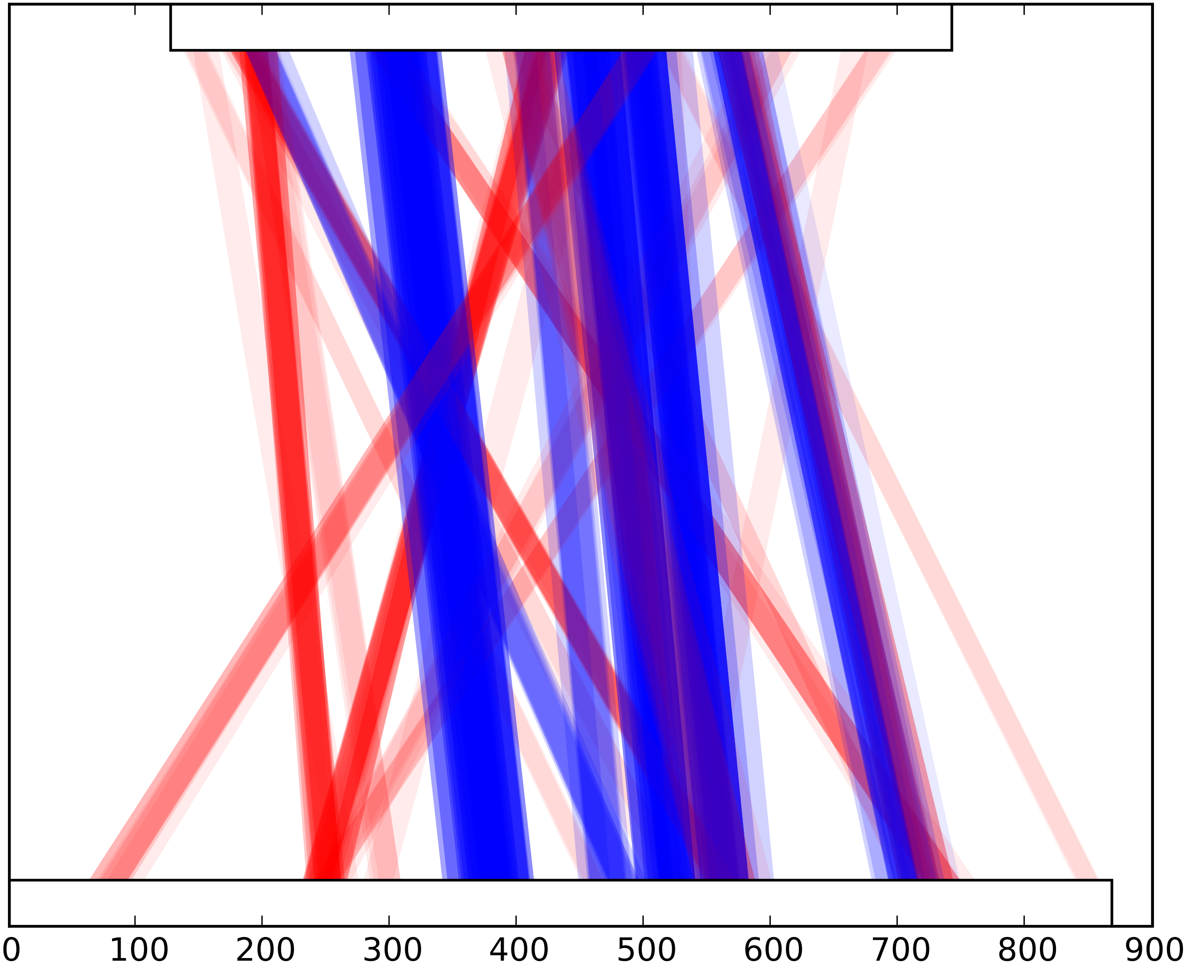

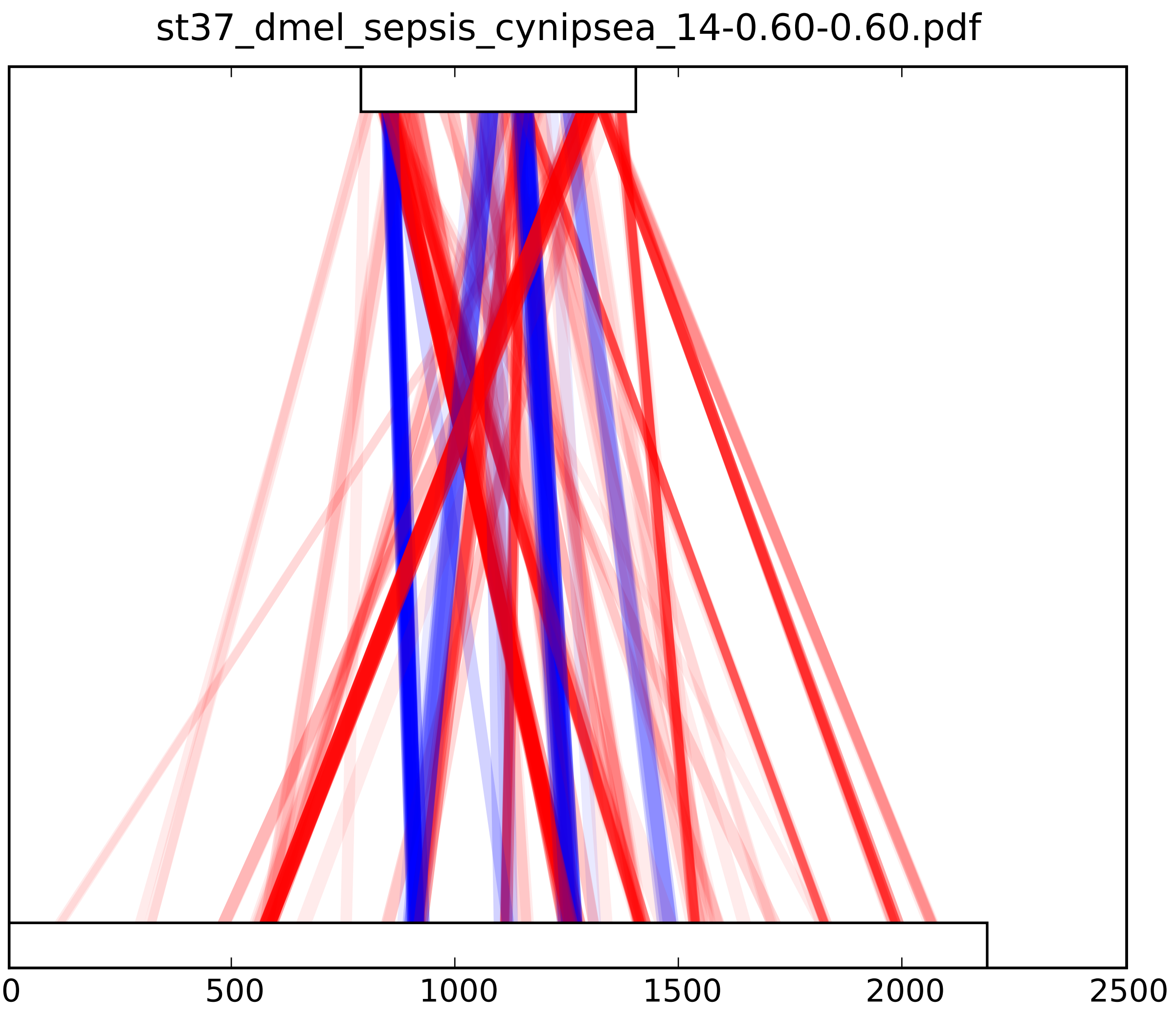
st37_dmel_sepsis_cynipsea_14-0.60-0.60.pdf
0
500
1000
1500
2000
2500

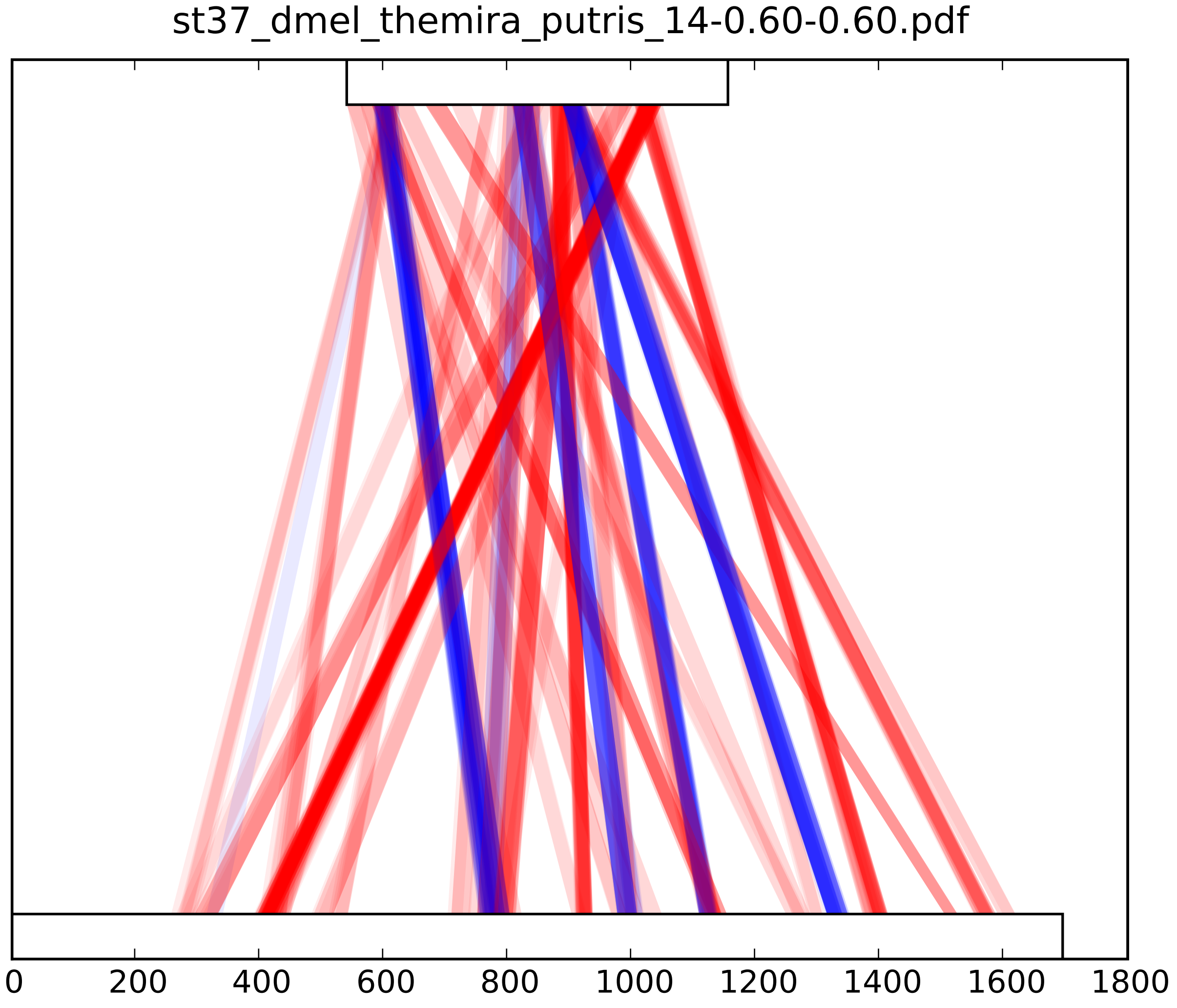

st37_dmel_themira_putris_14-0.60-0.60.pdf
0
200
400
600
800
1000
1200
1400
1600
1800

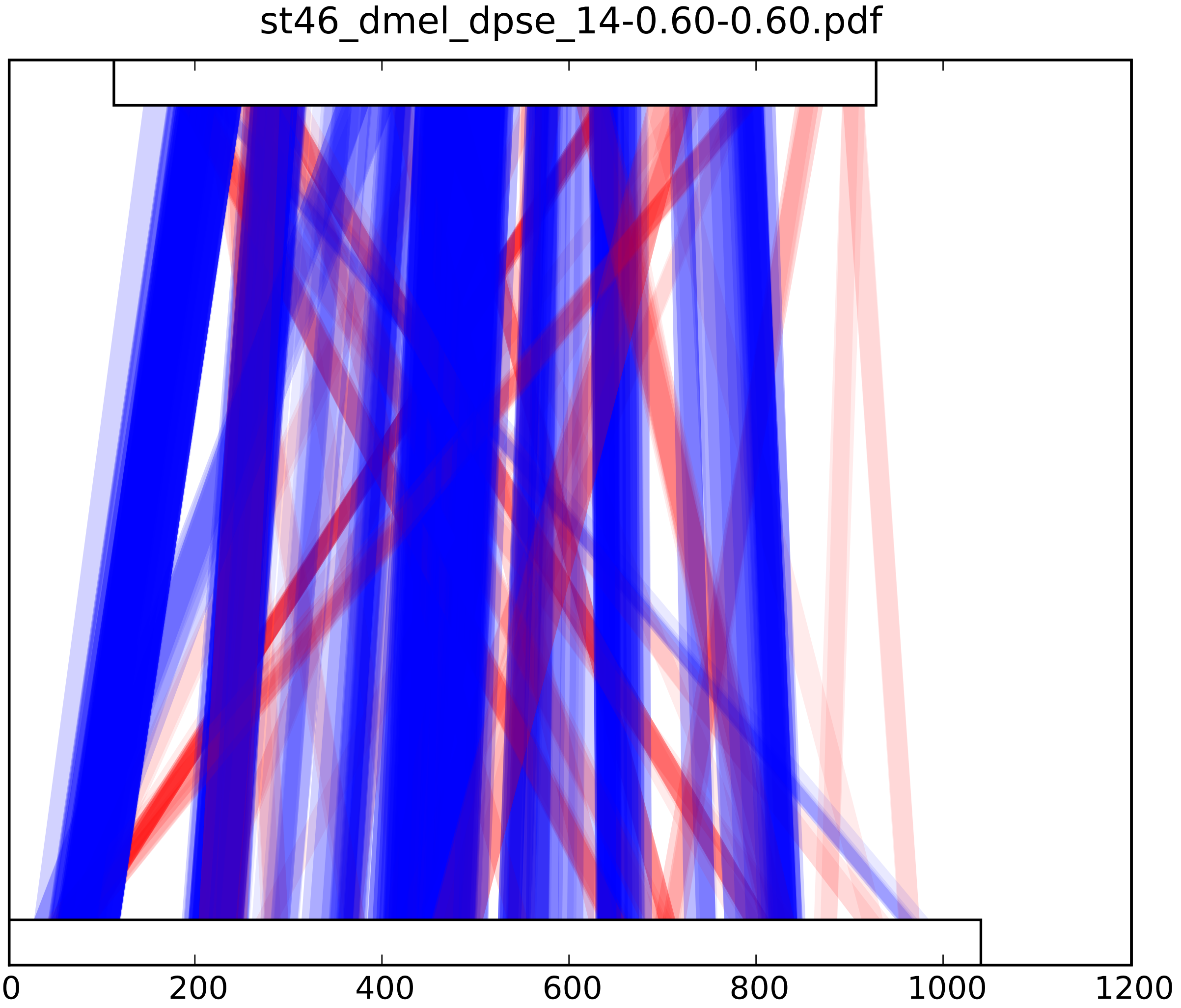

st46_dmel_dpse_14-0.60-0.60.pdf
0
200
400
600
800
1000
1200

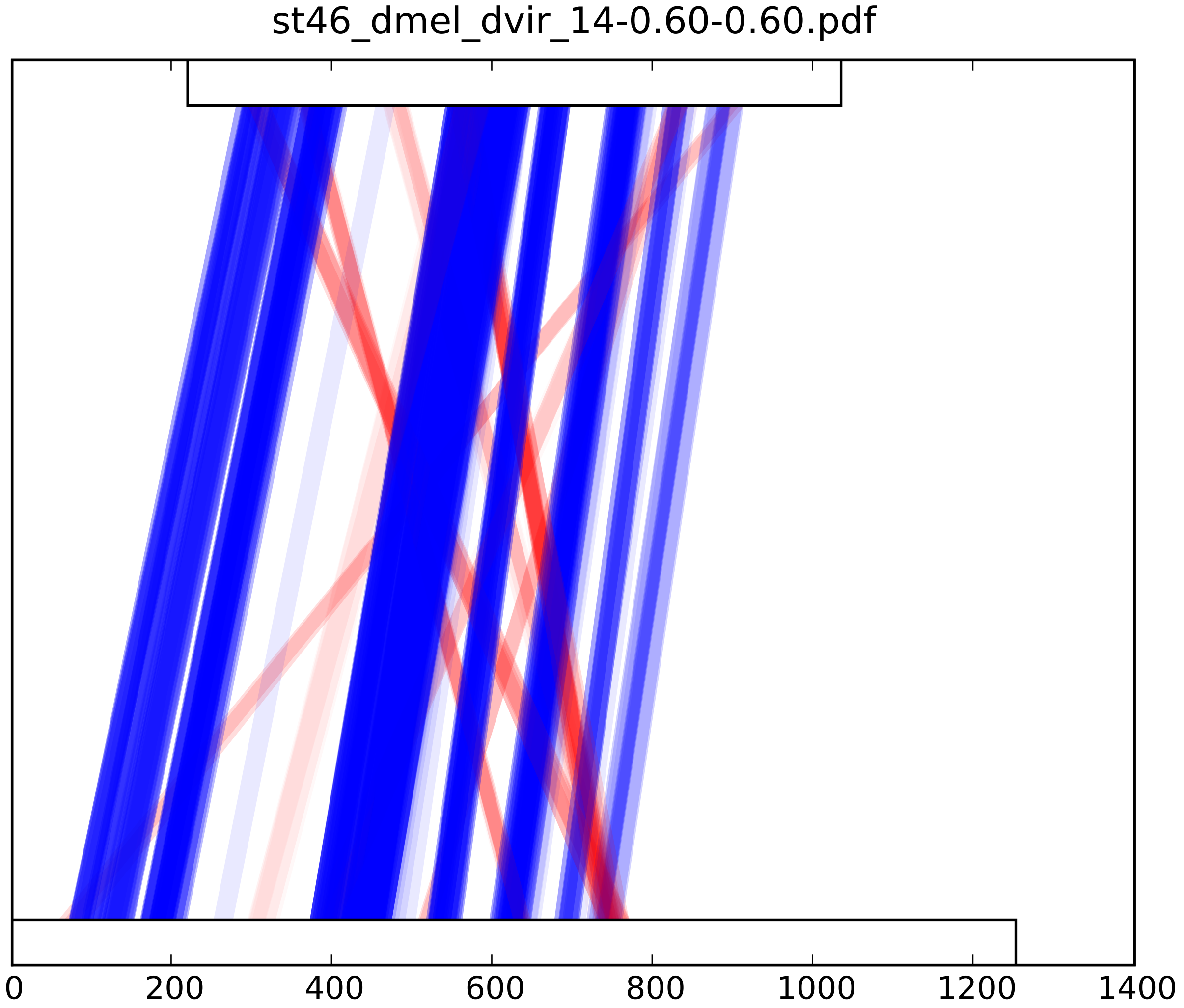
st46_dmel_dvir_14-0.60-0.60.pdf
0
200
400
600
800
1000
1200
1400

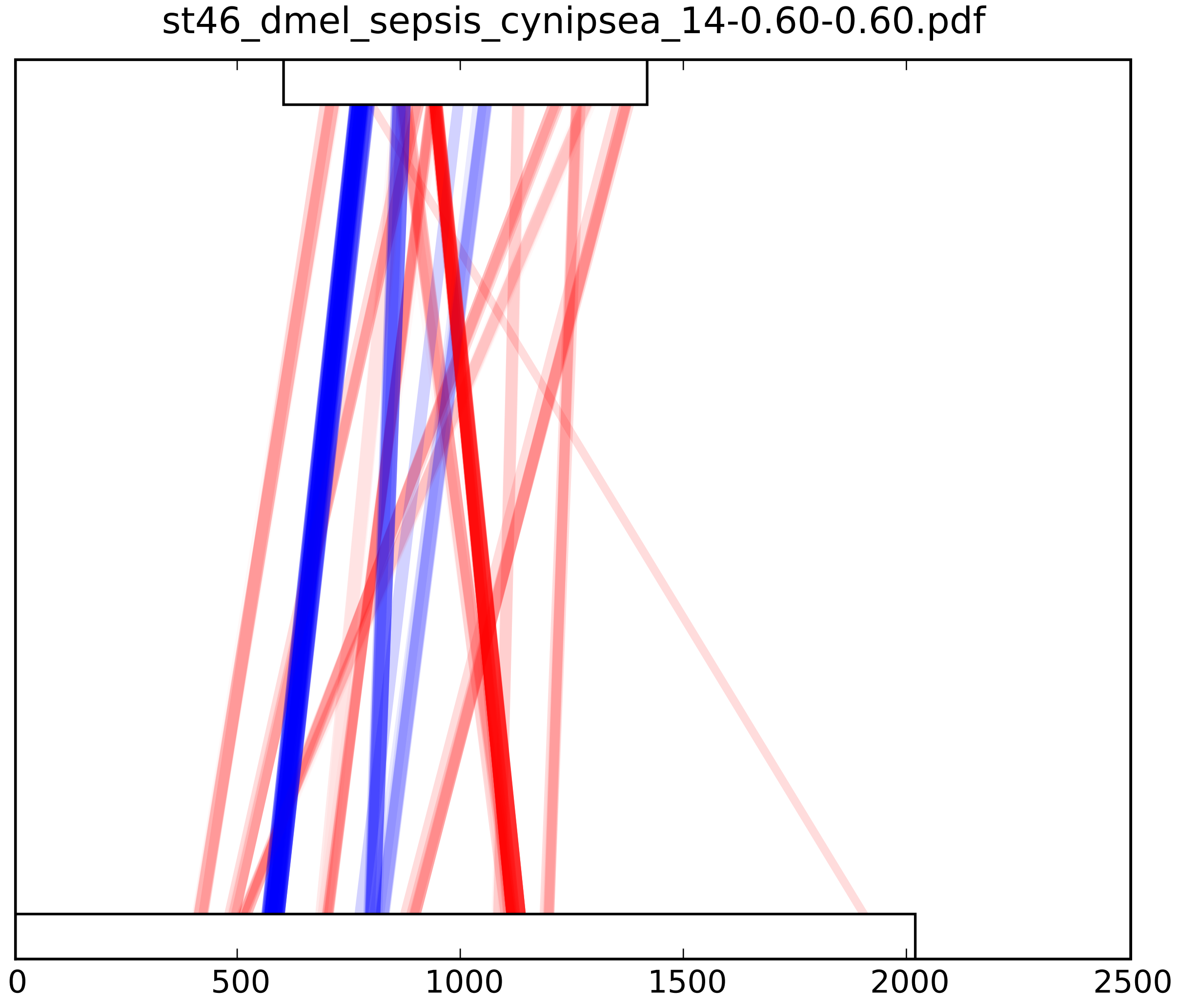
st46_dmel_sepsis_cynipsea_14-0.60-0.60.pdf
0
500
1000
1500
2000
2500

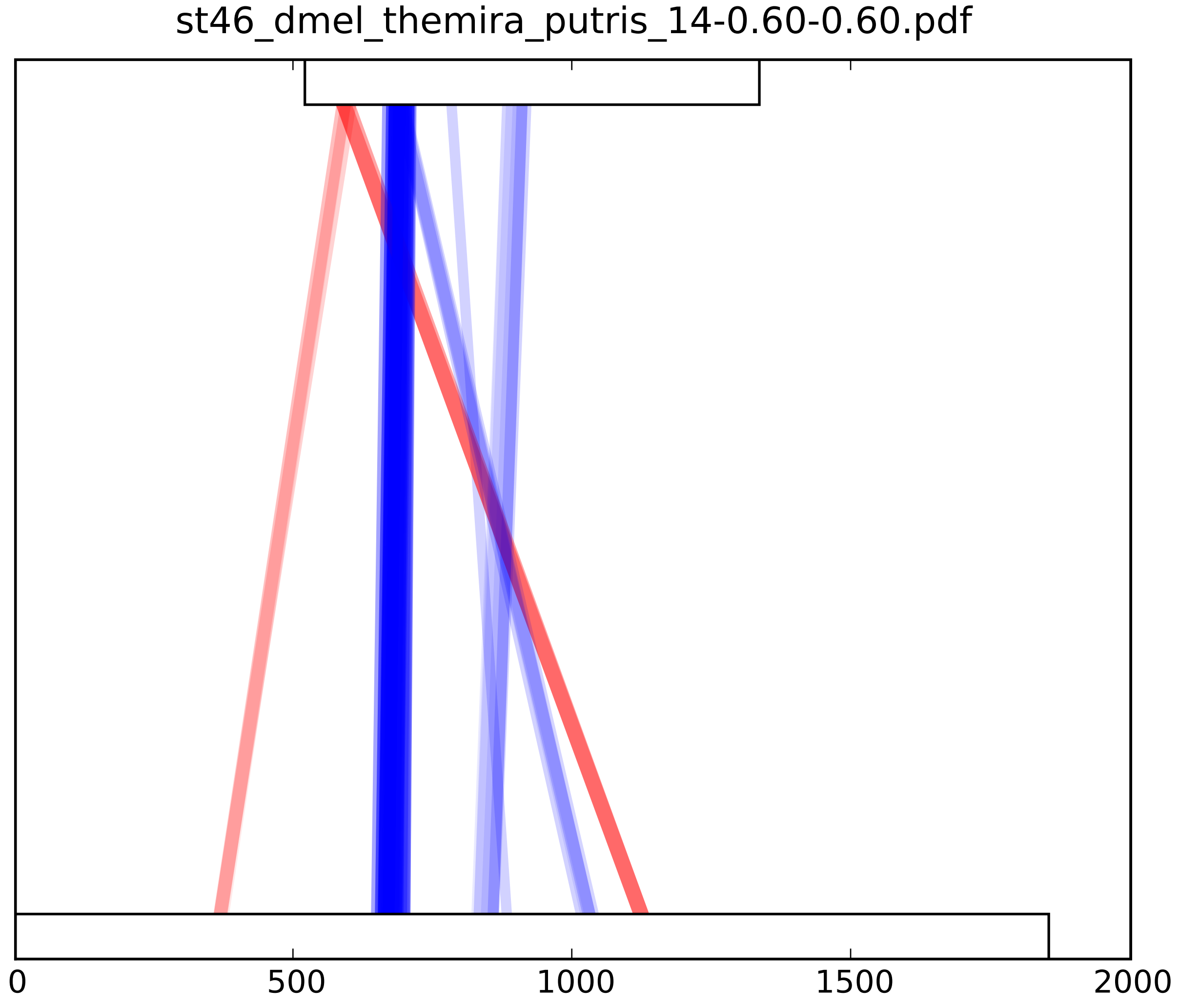

st46_dmel_themira_putris_14-0.60-0.60.pdf
0
500
1000
1500
2000

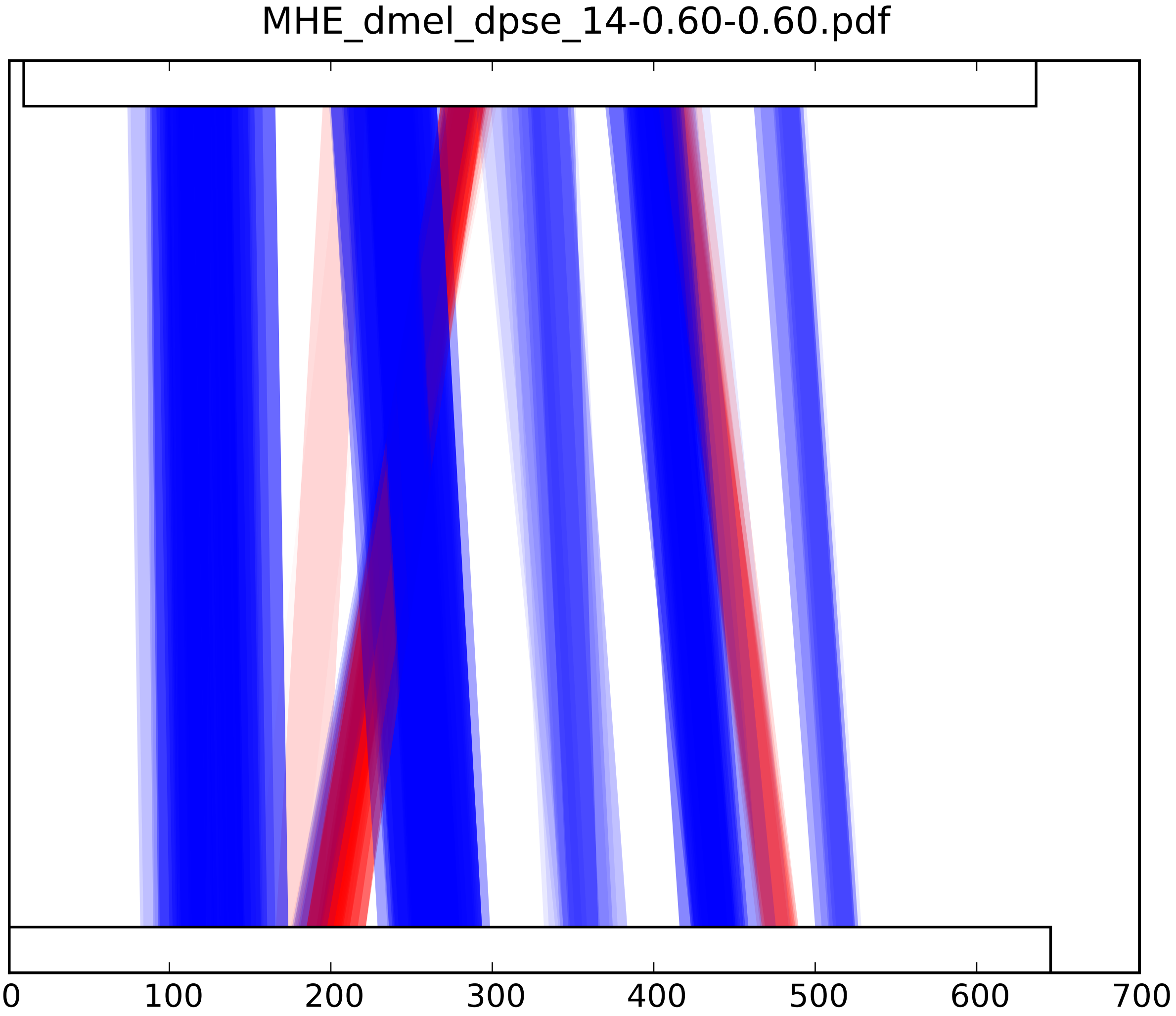
MHE_dmel_dpse_14-0.60-0.60.pdf
0
100
200
300
400
500
600
700

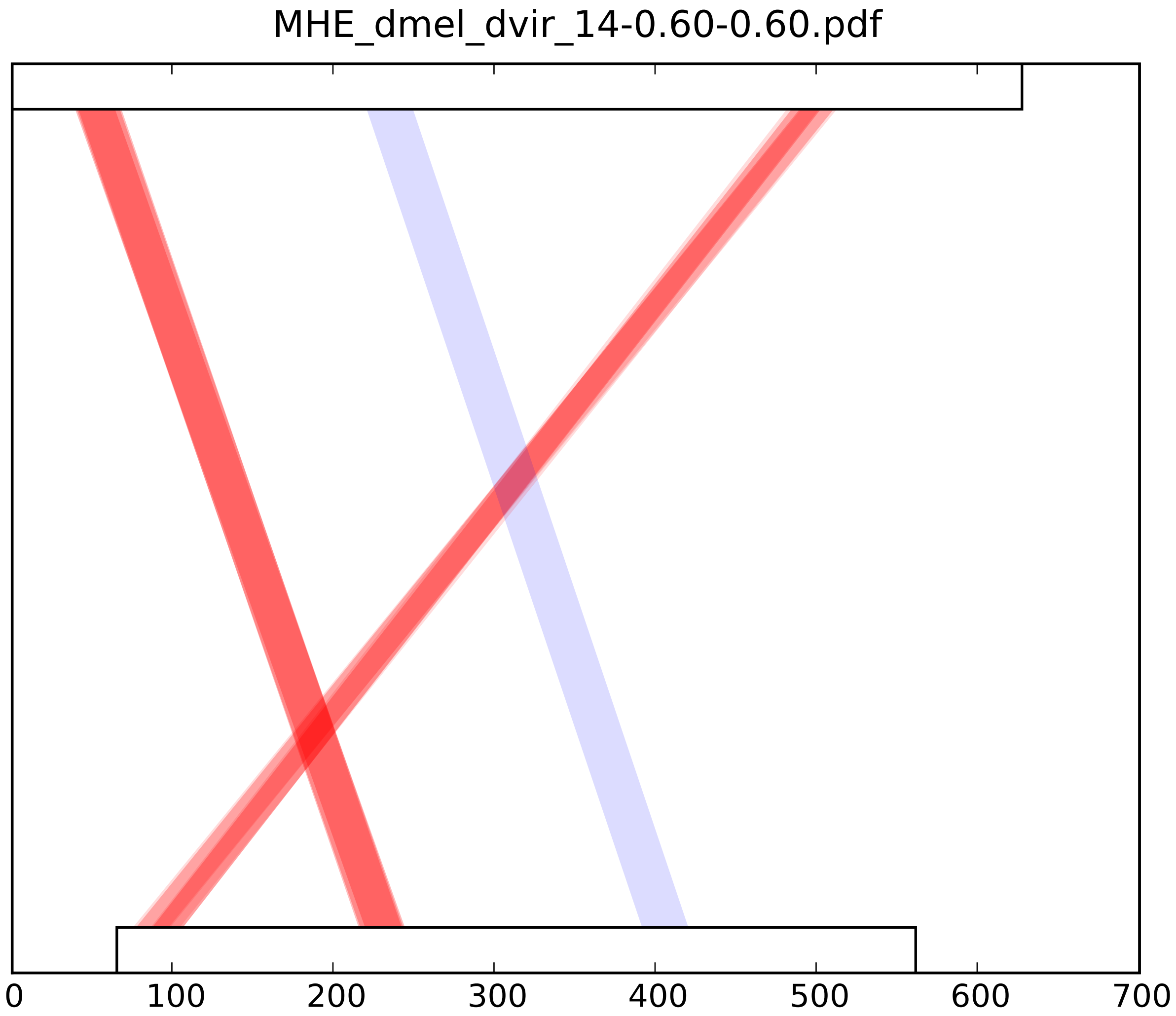
MHE_dmel_dvir_14-0.60-0.60.pdf
0
100
200
300
400
500
600
700

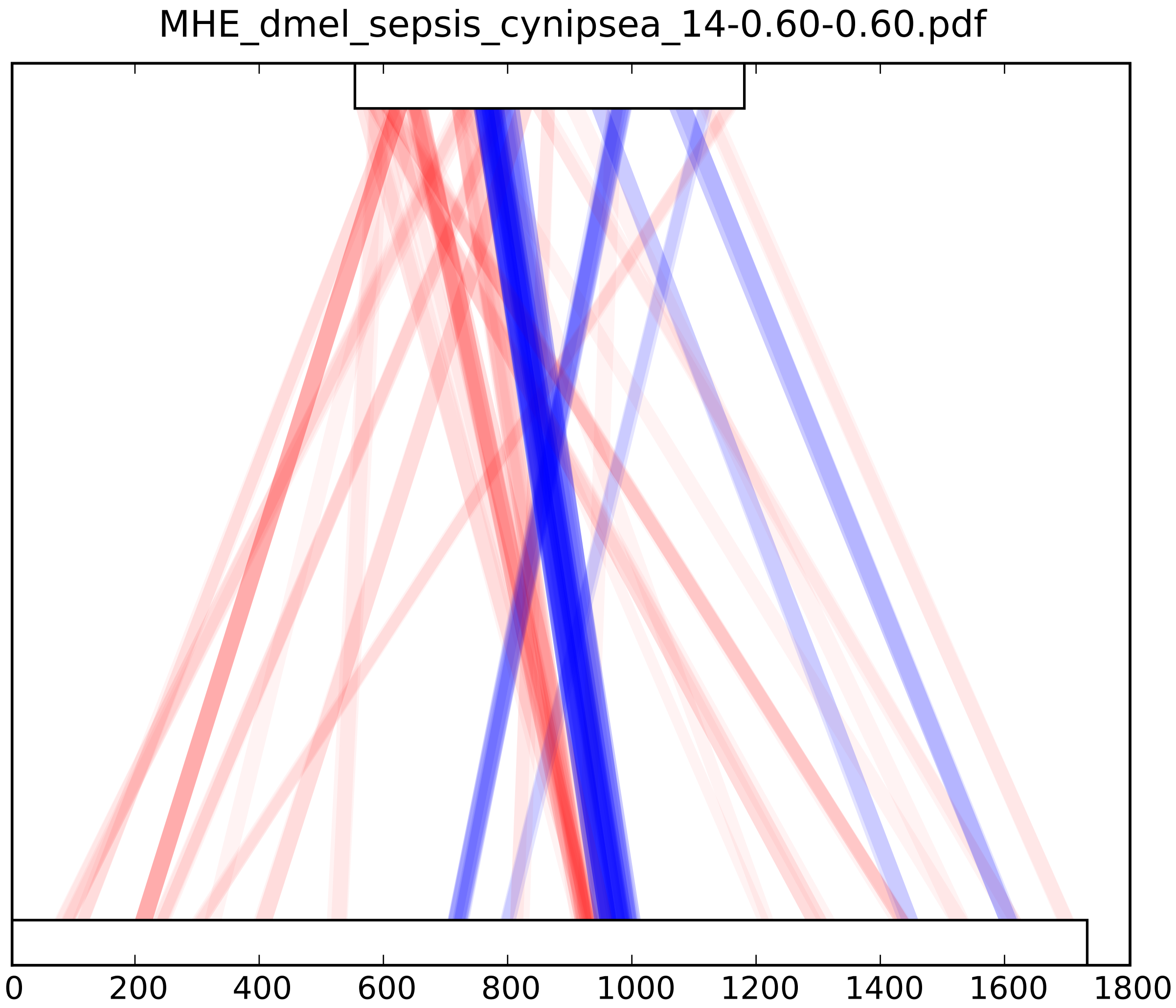
MHE_dmel_sepsis_cynipsea_14-0.60-0.60.pdf
0
200
400
600
800
1000
1200
1400
1600
1800

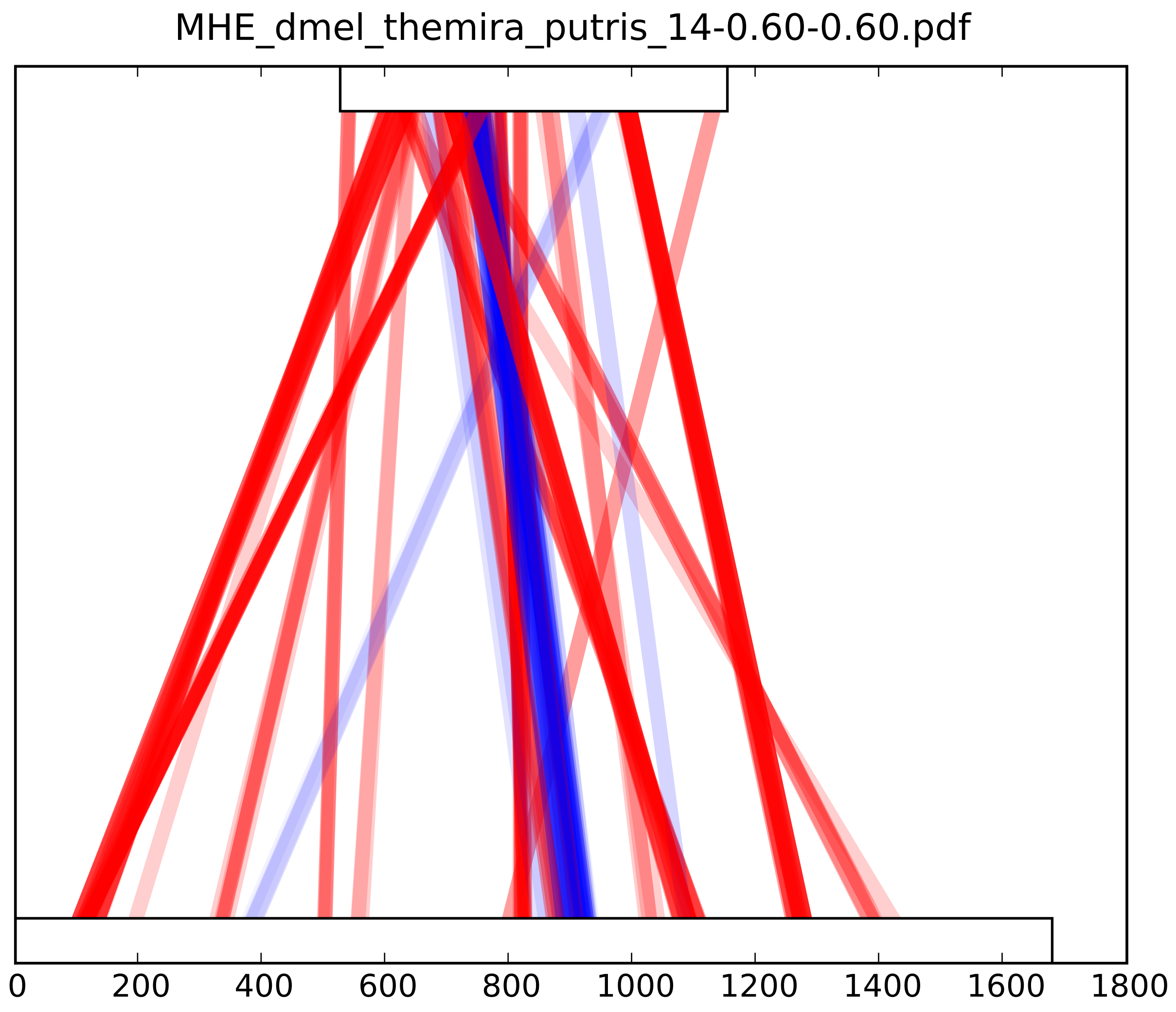
MHE_dmel_themira_putris_14-0.60-0.60.pdf
0
200
400
600
800
1000
1200
1400
1600
1800

st2_dmel_dpse_14-0.60-0.70.pdf

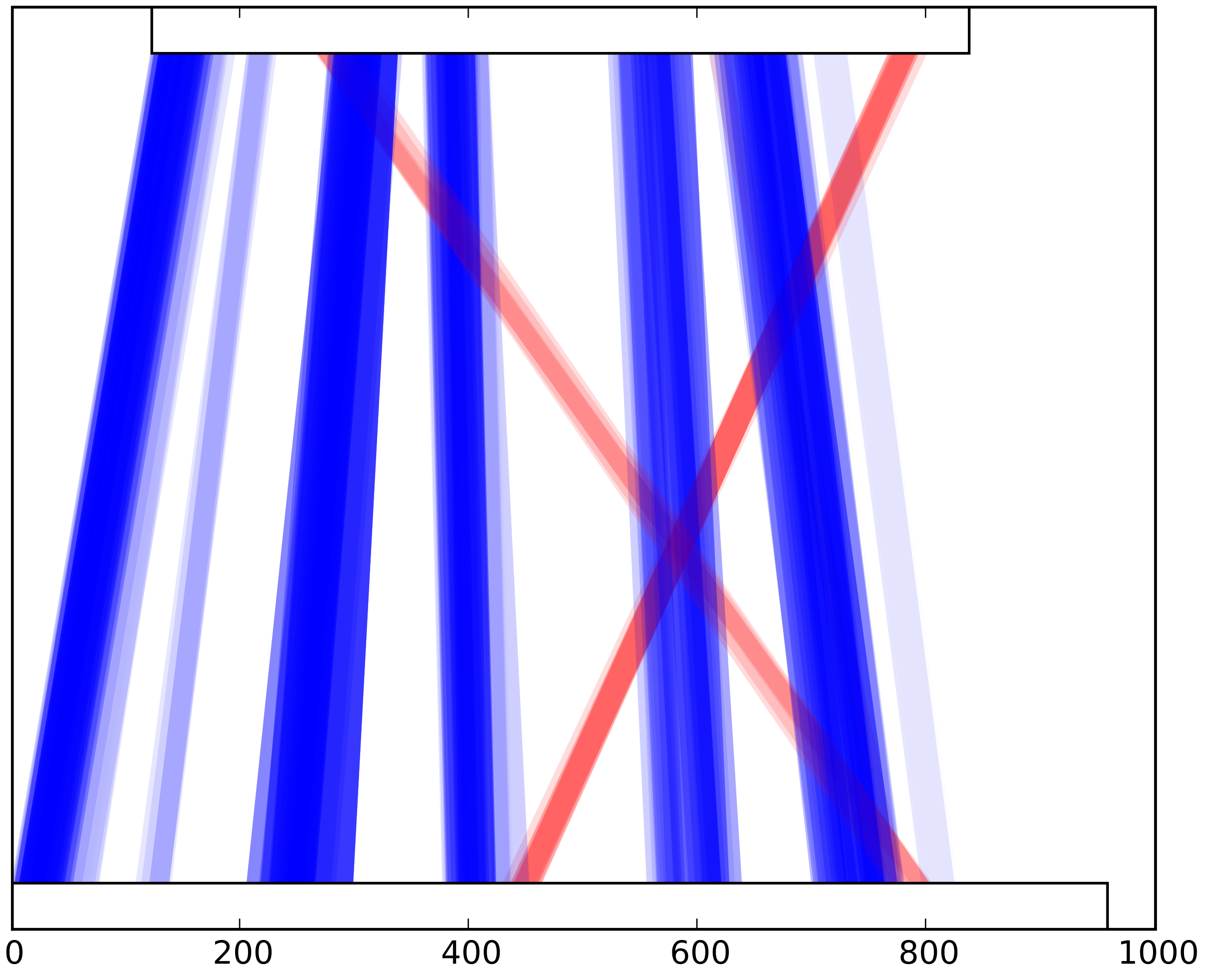

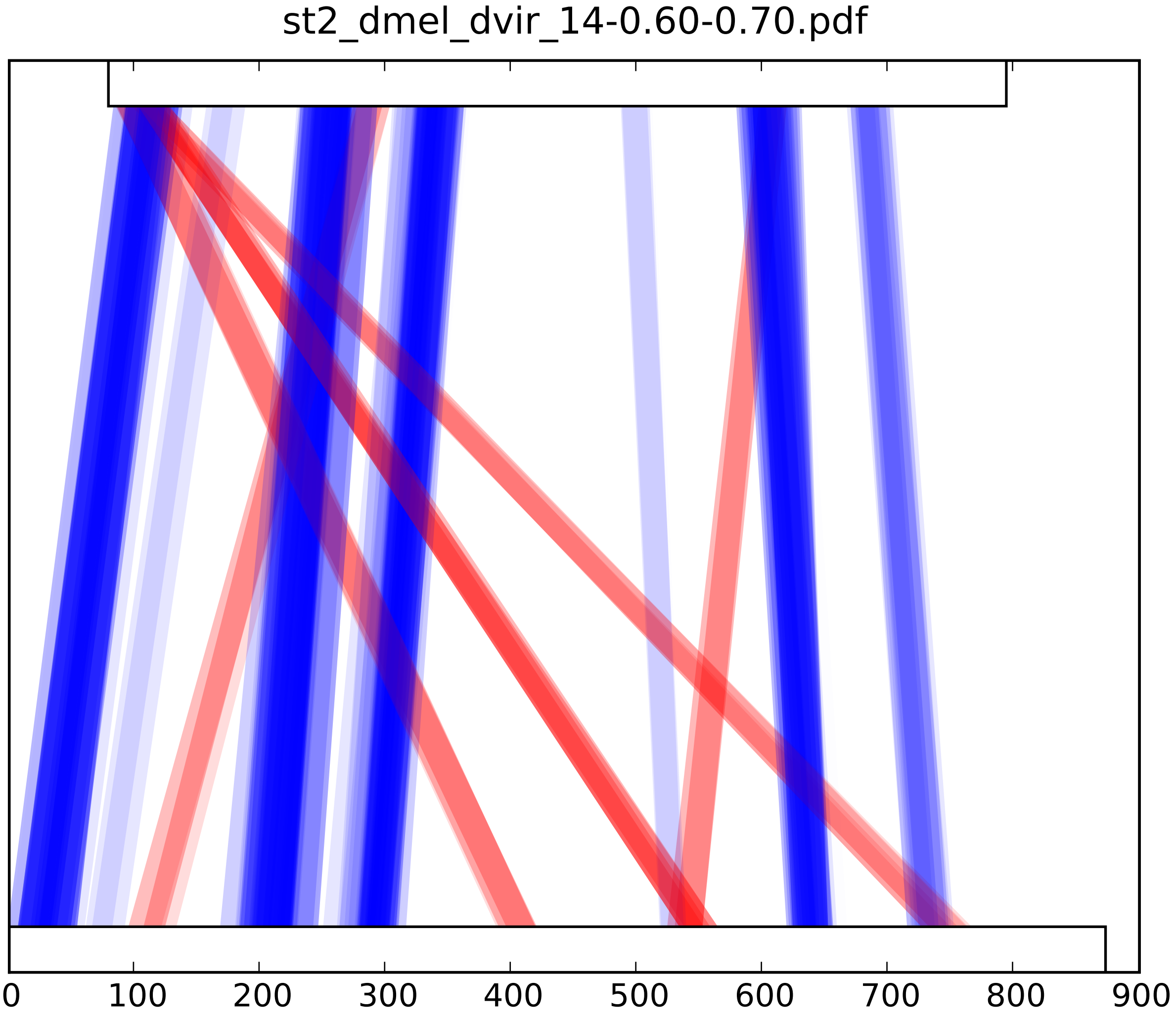
st2_dmel_dvir_14-0.60-0.70.pdf
0
100
200
300
400
500
600
700
800
900

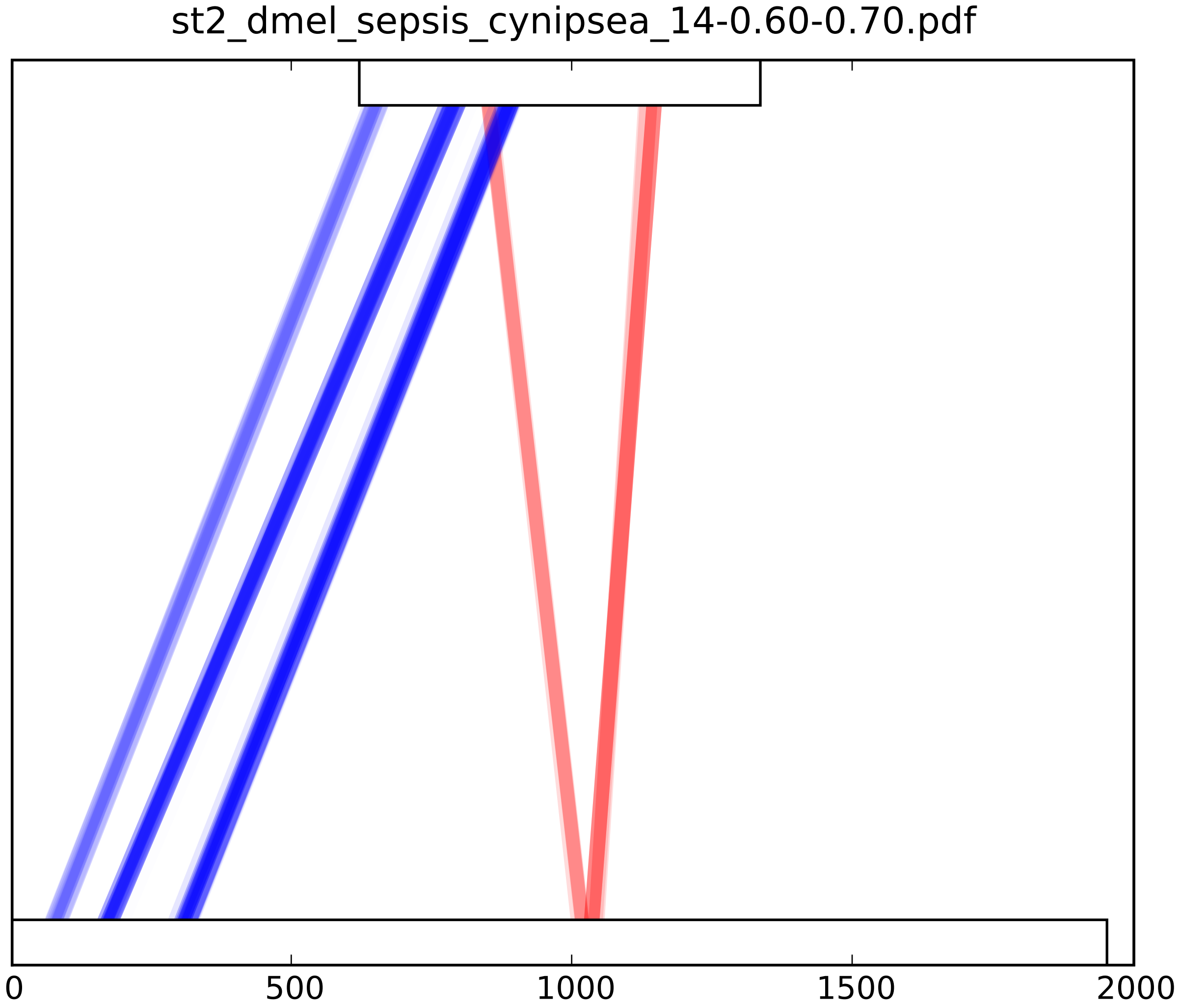
st2_dmel_sepsis_cynipsea_14-0.60-0.70.pdf
0
500
1000
1500
2000

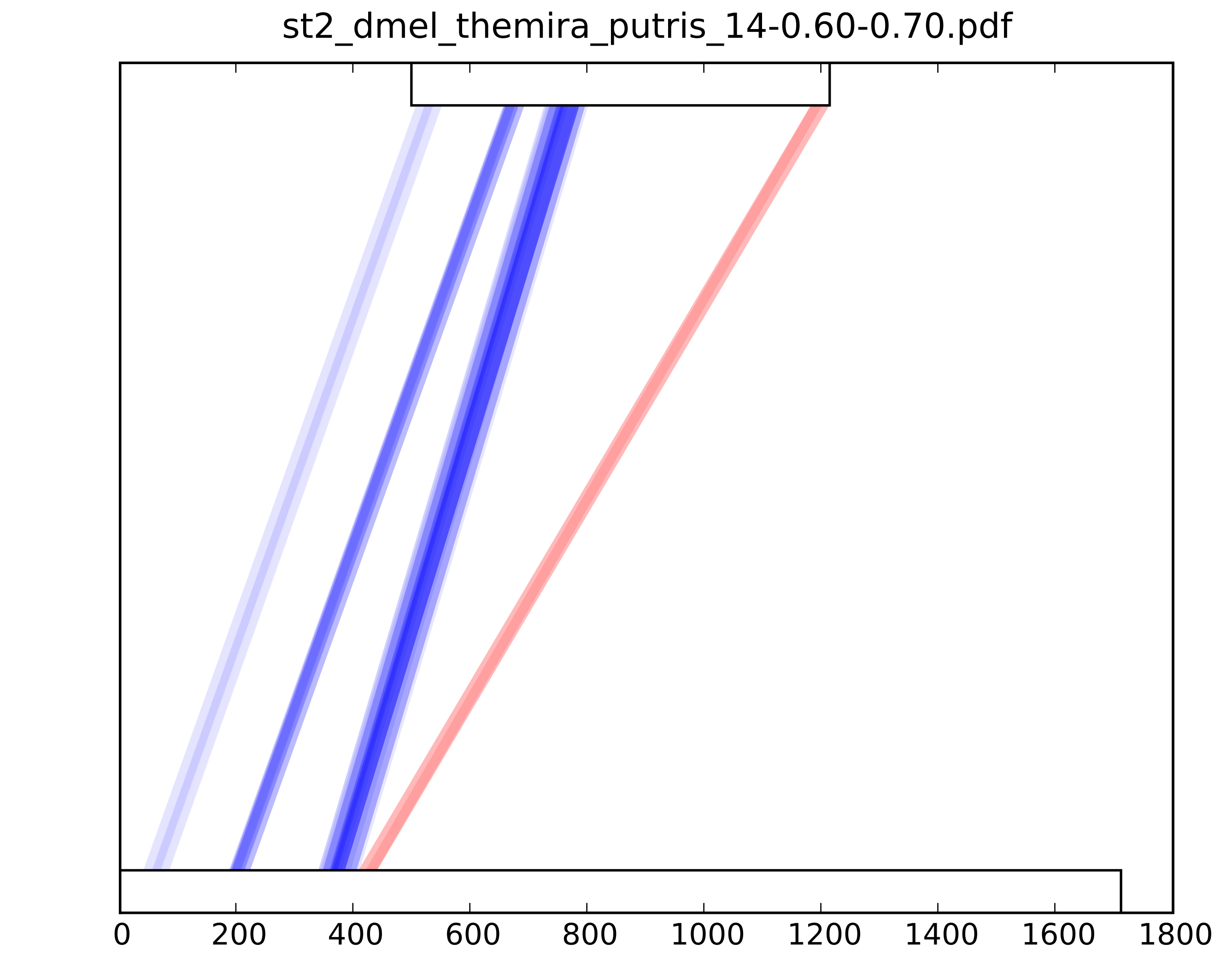
st2_dmel_themira_putris_14-0.60-0.70.pdf
0
200
400
600
800
1000
1200
1400
1600
1800

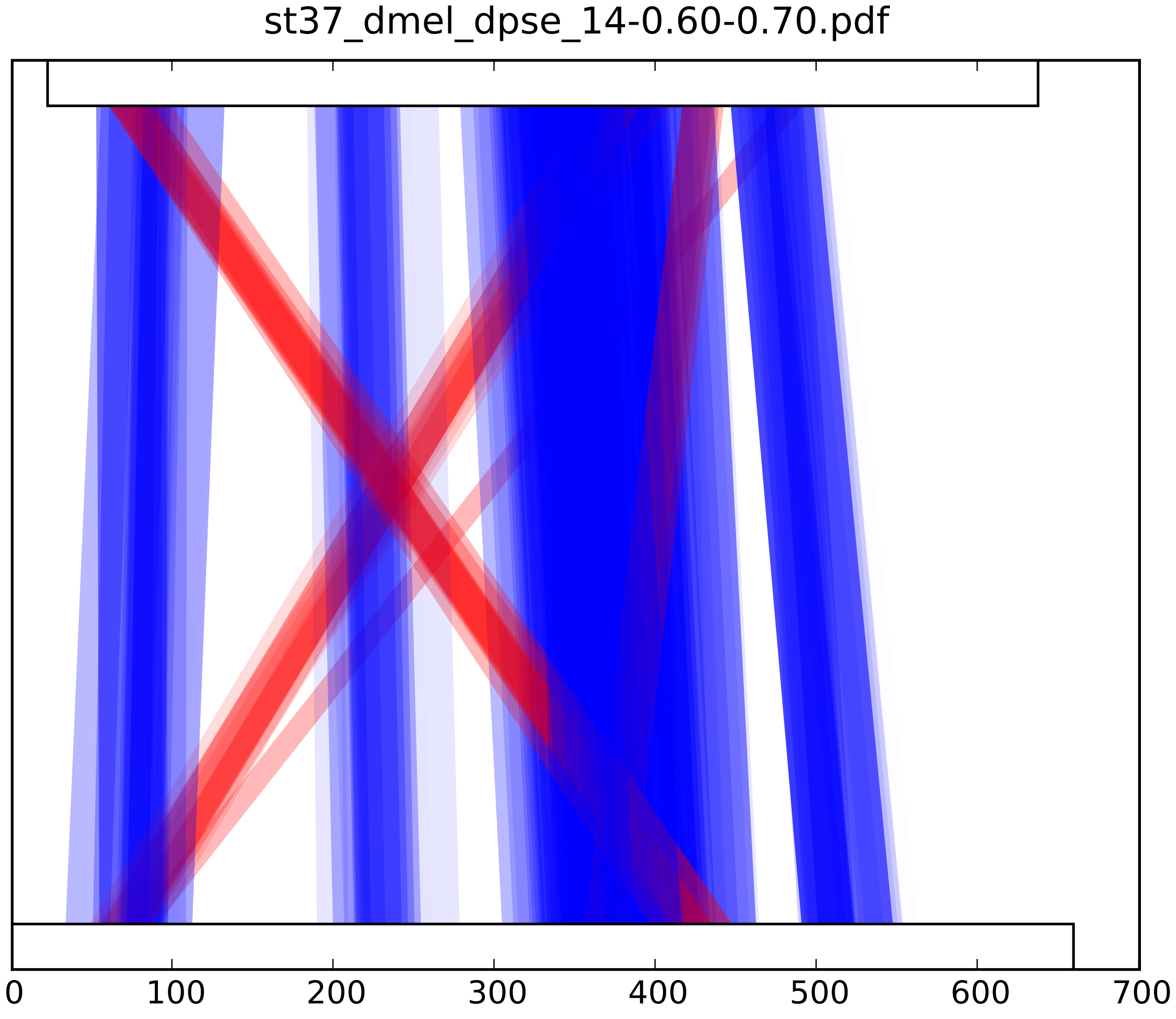
st37_dmel_dpse_14-0.60-0.70.pdf
0
100
200
300
400
500
600
700

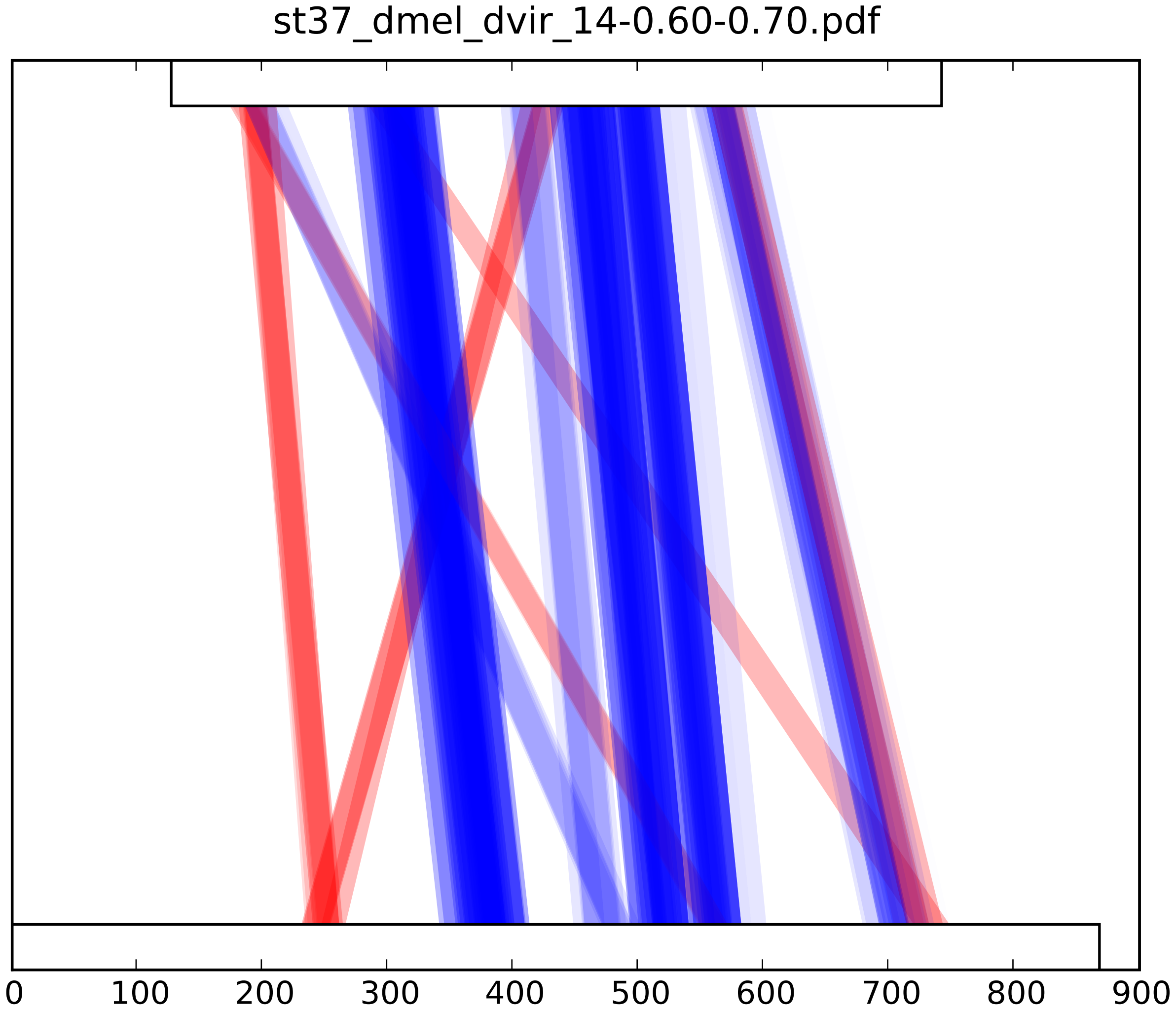
st37_dmel_dvir_14-0.60-0.70.pdf
0
100
200
300
400
500
600
700
800
900

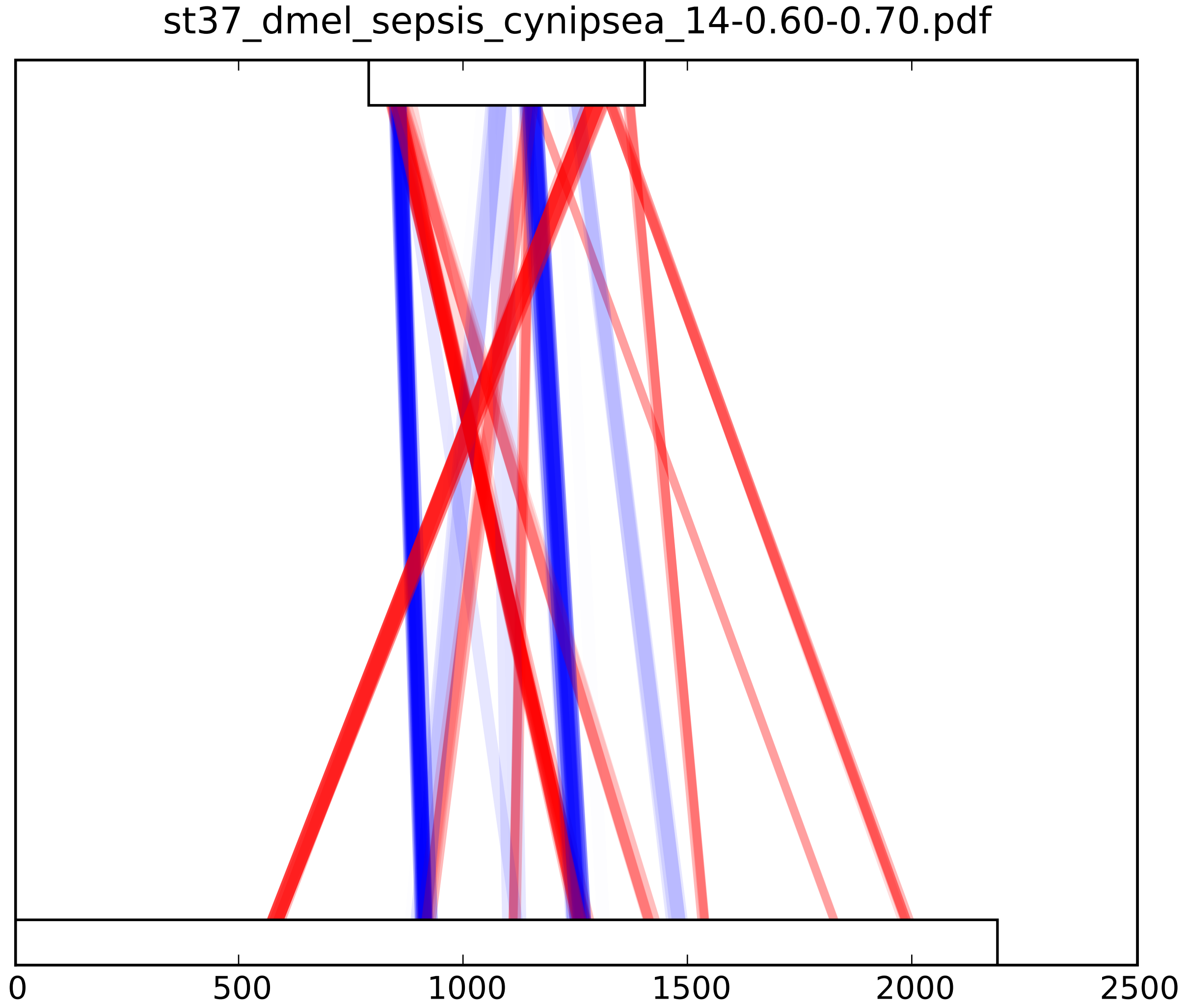
st37_dmel_sepsis_cynipsea_14-0.60-0.70.pdf
0
500
1000
1500
2000
2500

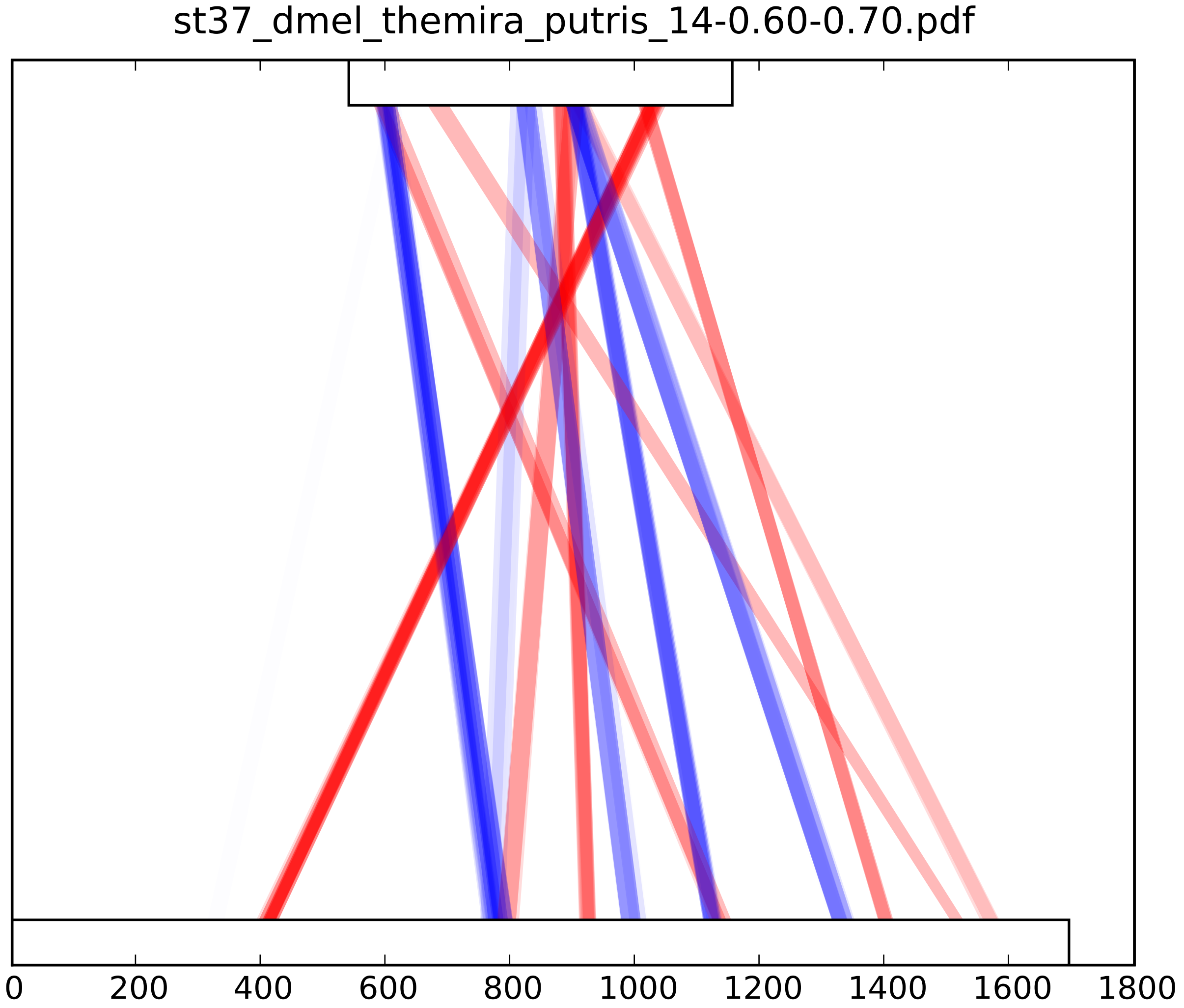
st37_dmel_themira_putris_14-0.60-0.70.pdf
0
200
400
600
800
1000
1200
1400
1600
1800

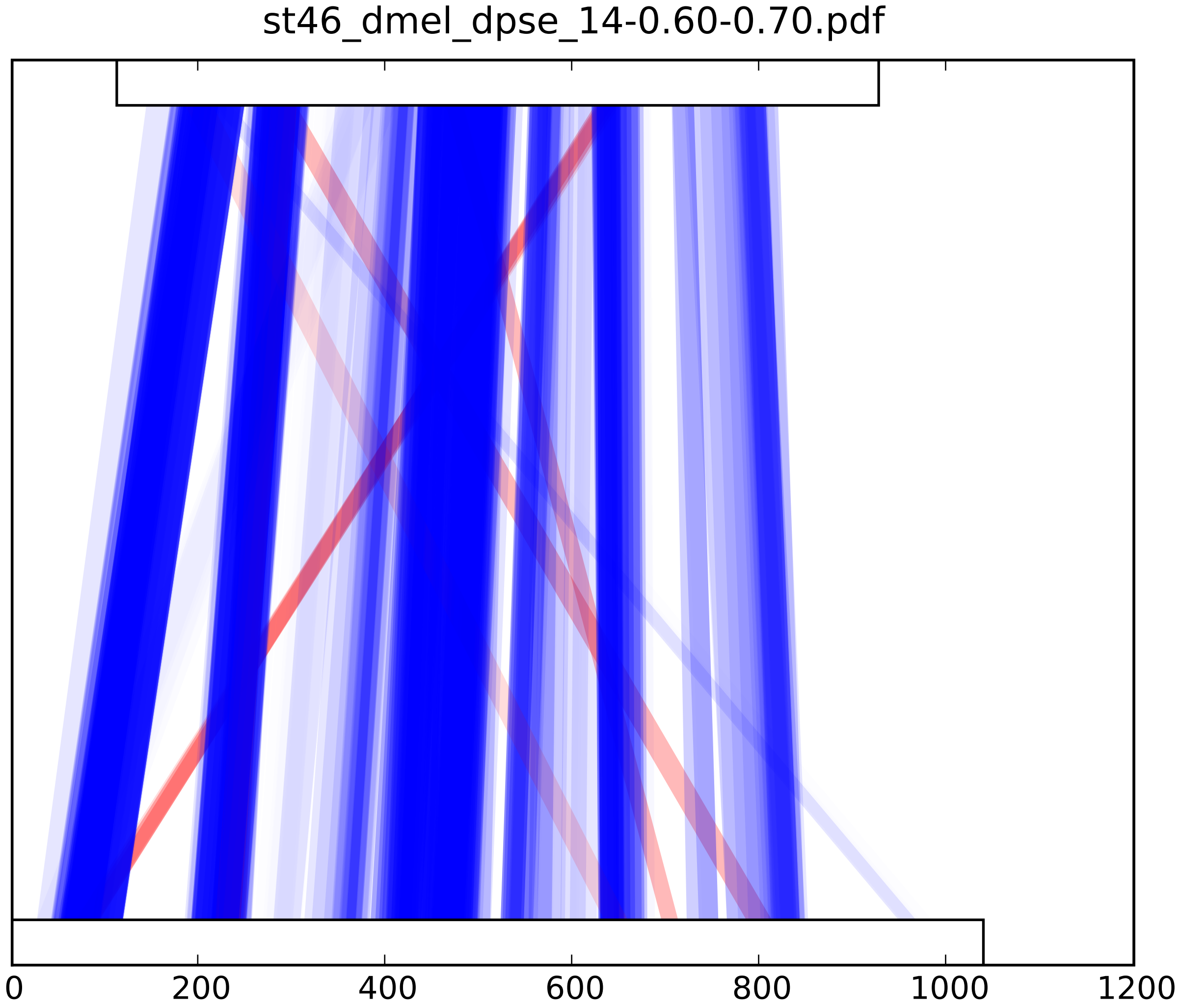
st46_dmel_dpse_14-0.60-0.70.pdf
0
200
400
600
800
1000
1200

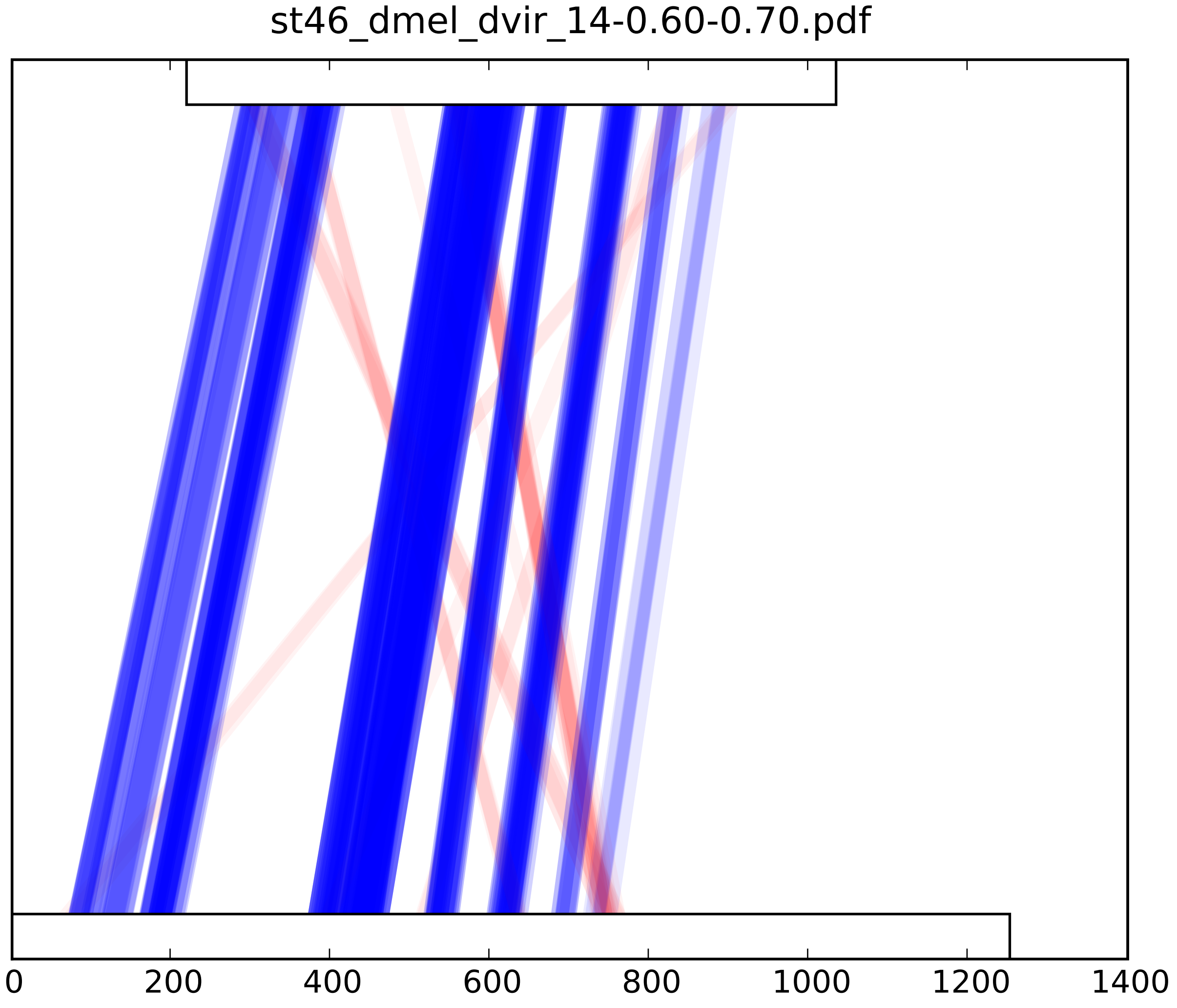
st46_dmel_dvir_14-0.60-0.70.pdf
0
200
400
600
800
1000
1200
1400

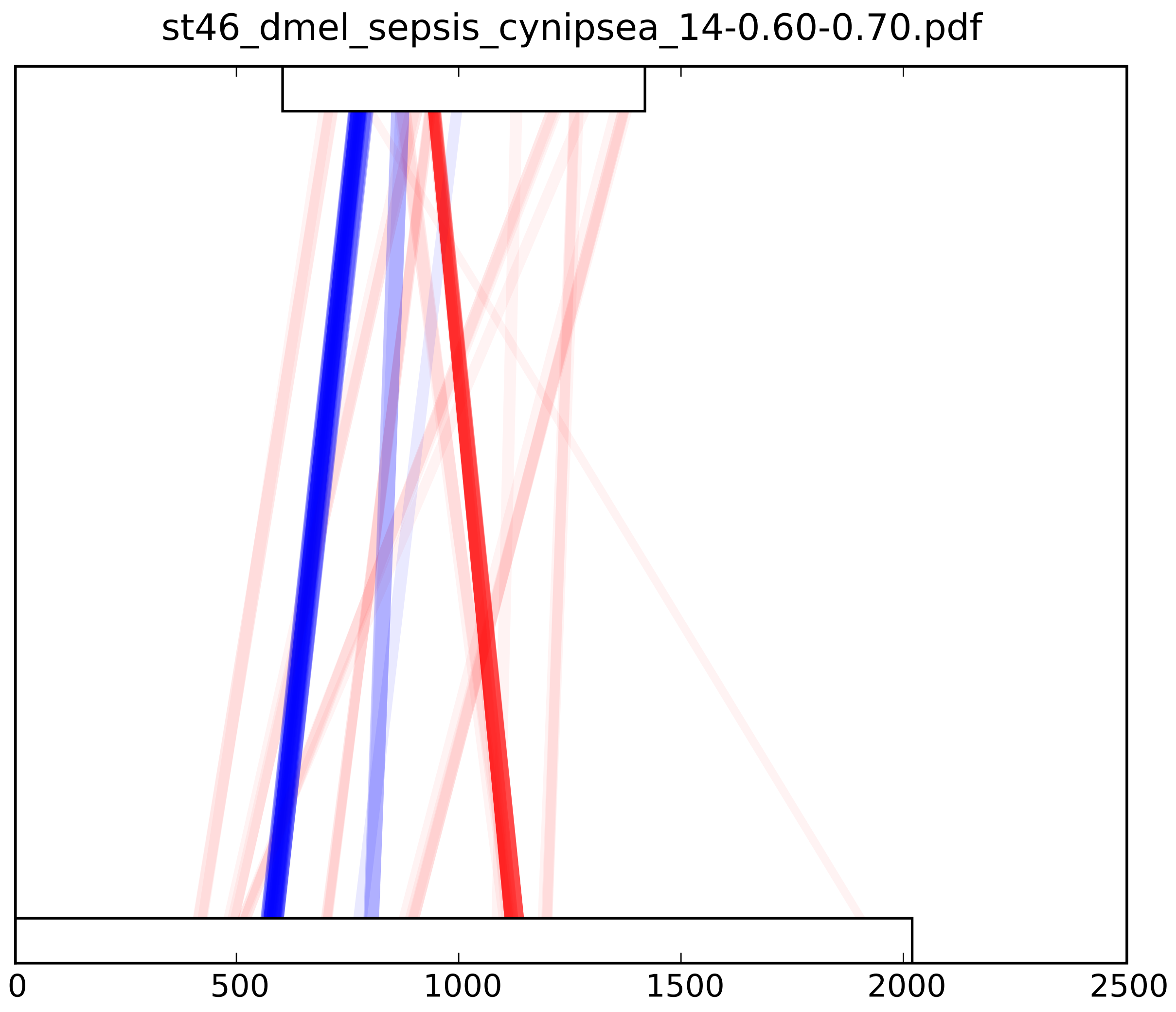
st46_dmel_sepsis_cynipsea_14-0.60-0.70.pdf
0
500
1000
1500
2000
2500

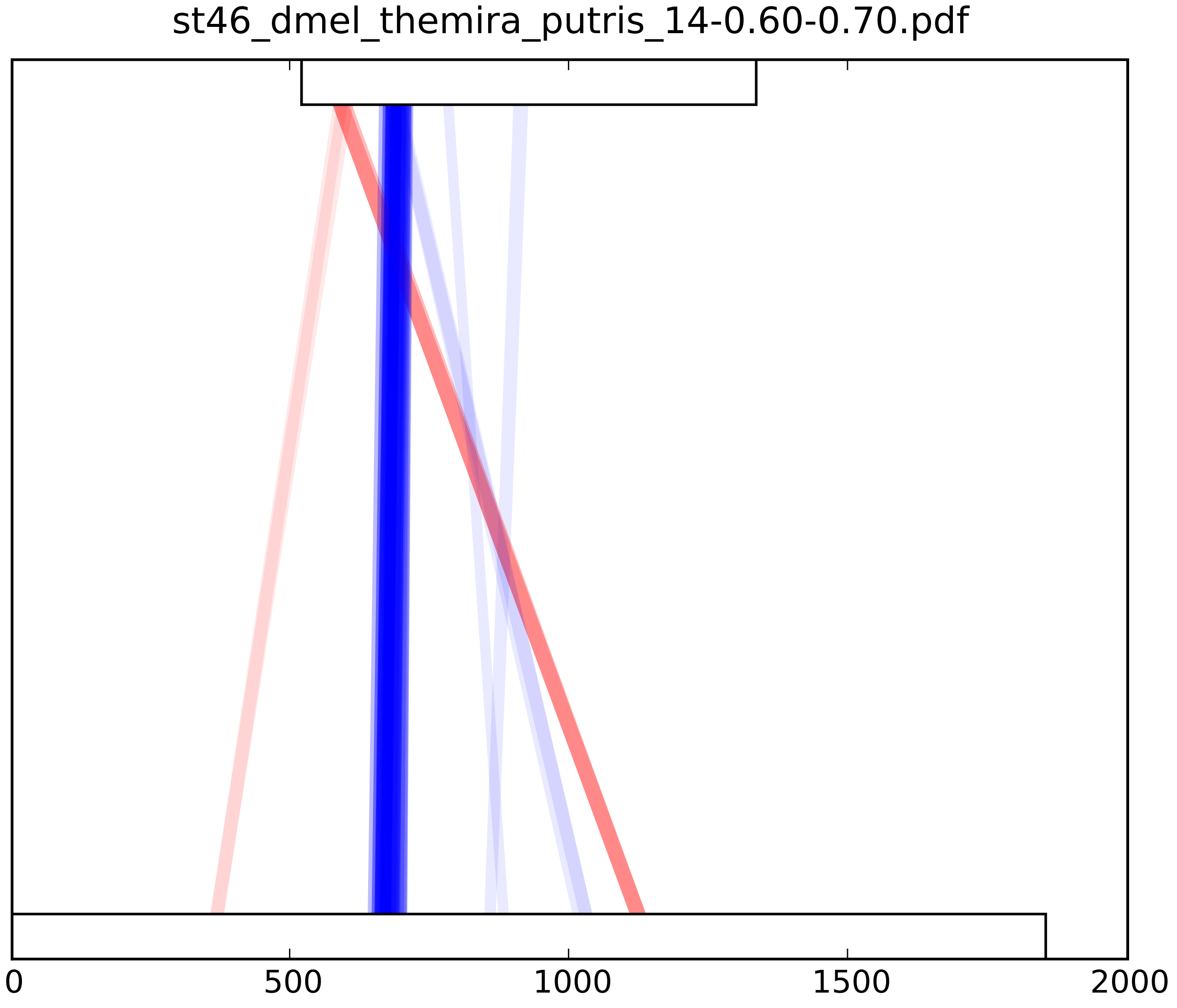
st46_dmel_themira_putris_14-0.60-0.70.pdf
0
500
1000
1500
2000

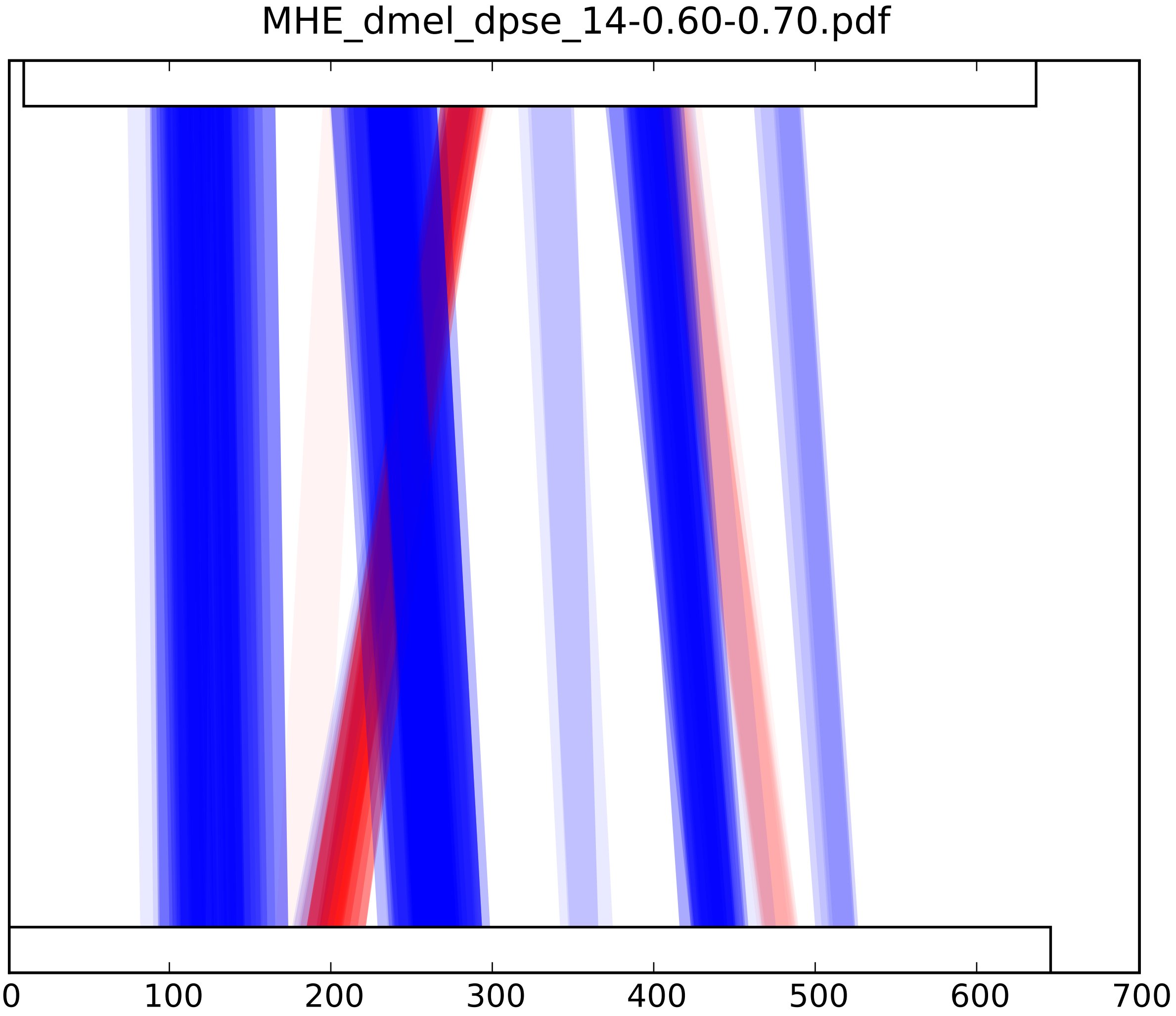
MHE_dmel_dpse_14-0.60-0.70.pdf
0
100
200
300
400
500
600
700

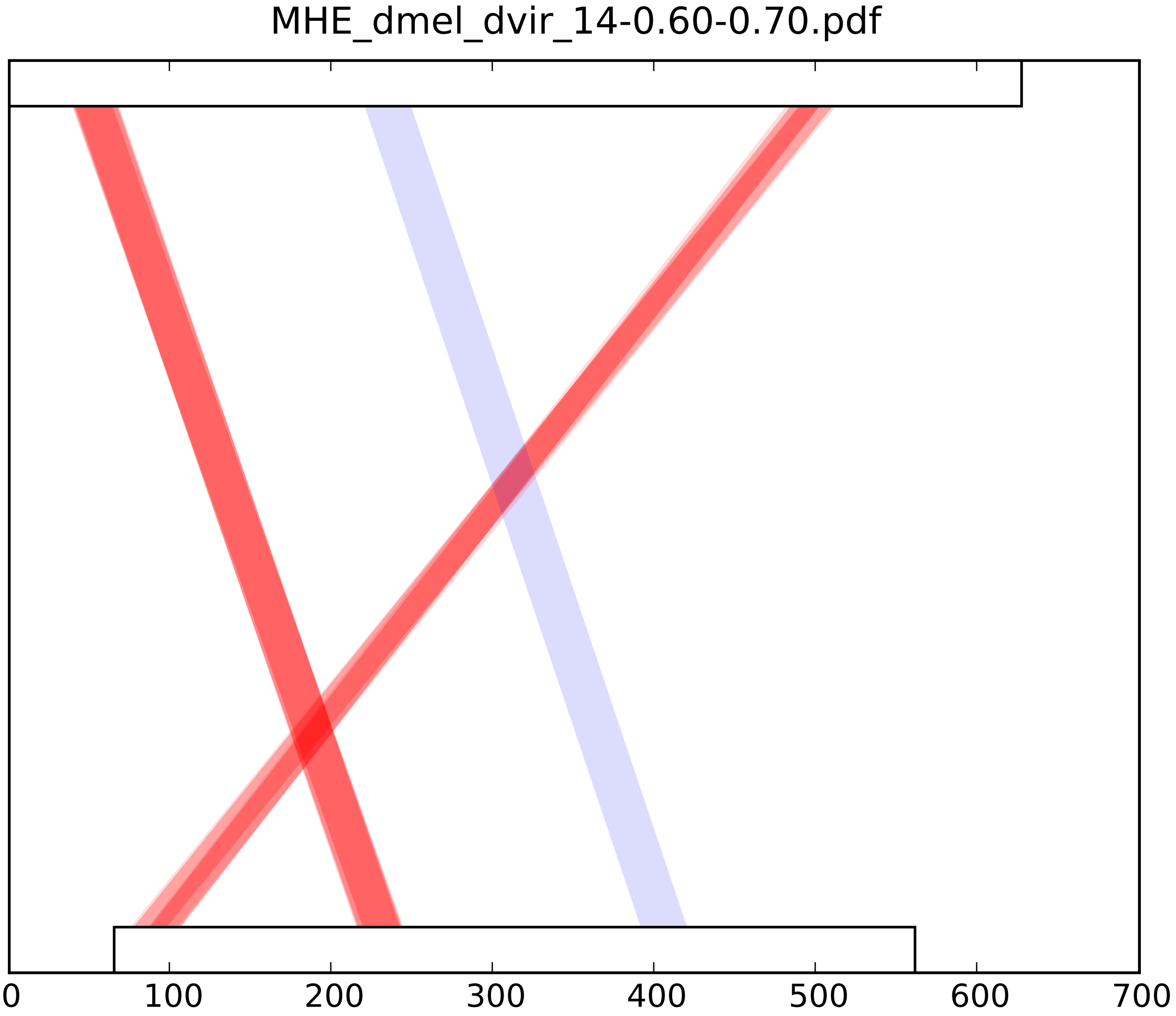
MHE_dmel_dvir_14-0.60-0.70.pdf
0
100
200
300
400
500
600
700

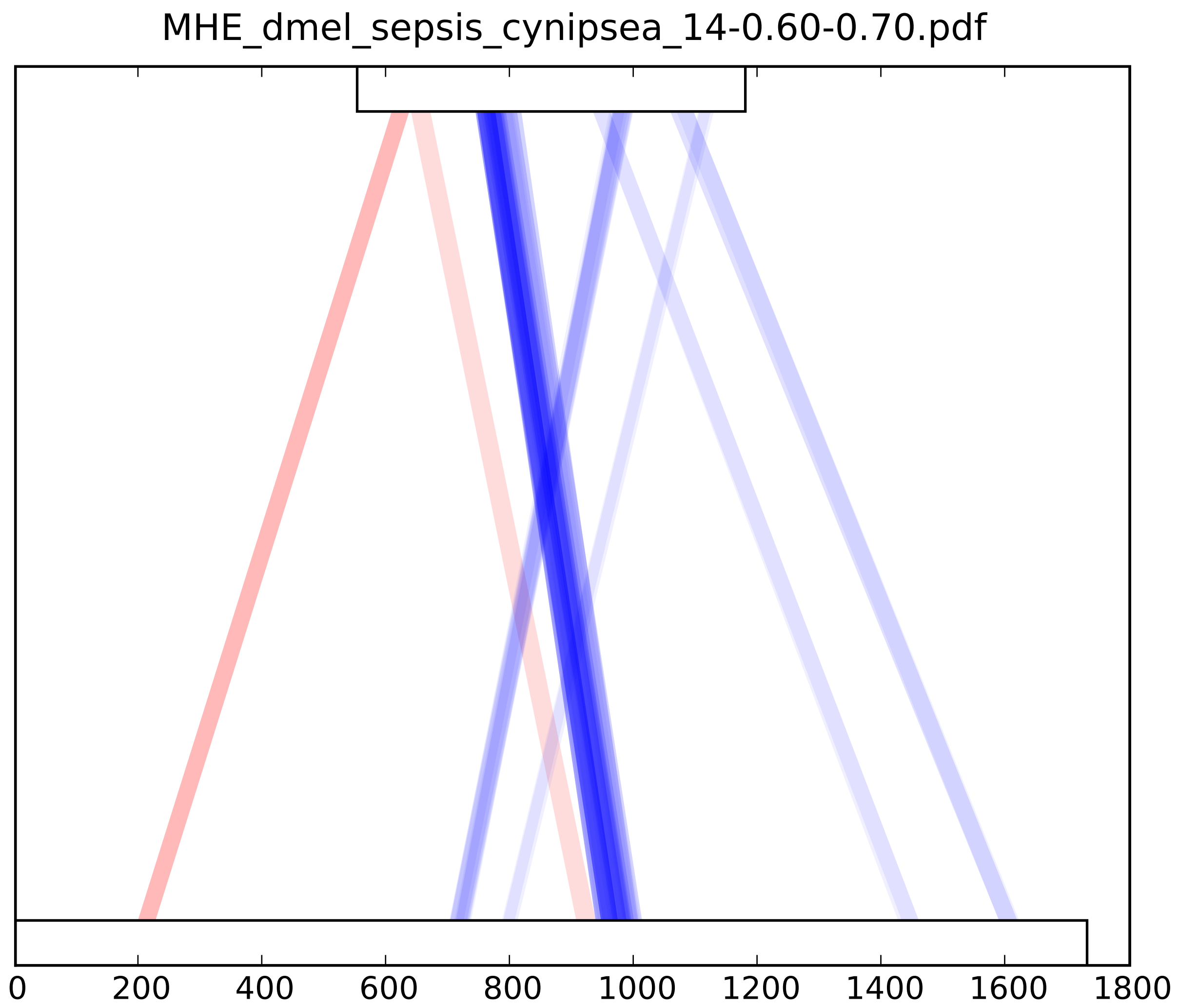
MHE_dmel_sepsis_cynipsea_14-0.60-0.70.pdf
0
200
400
600
800
1000
1200
1400
1600
1800

MHE_dmel_themira_putris_14-0.60-0.70.pdf

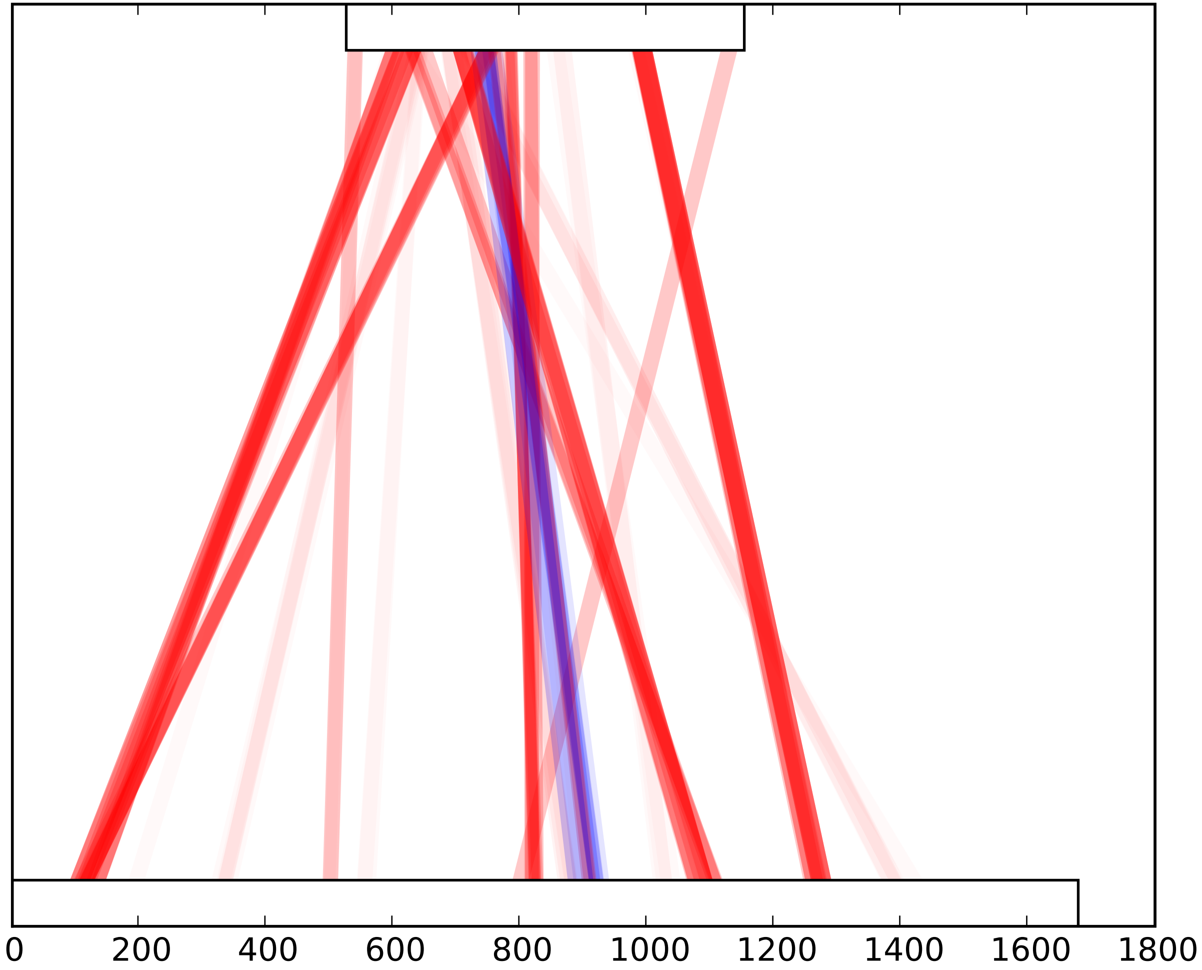

Supplement: Figure S2 — Similarity maps (derived from dot plots and BLAST-based similarity plots) for four even-skipped enhancers in multiple Drosophila and sepsid species. Similarity maps were computed for dotplots and BLAST based 2D similarity plots shown in Figure S1. For each set of dot plots, three maps are shown, each with a different threshold on which dotplot hits are shown: cutoffs of 0.50, 0.60, and 0.70 representing the position of the score for the hit between the highest and lowest scores (a cutoff of 0.60, for example, means that only hits in the top 40% of the range shown in the dot plot are mapped). For BLAST-based similarity maps, all HSPs in the similarity plots are shown. Blue boxes represent forward strand hits, red boxes indicate reverse strand hits. The opacity of the color was scaled so that the highest scoring hits had maximal opacity of 1.0 and the lowest scoring hit had opacity of 0.1. (1.37 MB PDF). [file pgen.1000268.s002.pdf]
